# Annual mass budget of Antarctic ice shelves from 1997 to 2021

**DOI:** 10.1126/sciadv.adi0186

**Published:** 2023-10-12

**Authors:** Benjamin J. Davison, Anna E. Hogg, Noel Gourmelen, Livia Jakob, Jan Wuite, Thomas Nagler, Chad A. Greene, Julia Andreasen, Marcus E. Engdahl

**Affiliations:** ^1^ School of Earth and Environment, University of Leeds, Leeds, UK.; ^2^ School of Geosciences, University of Edinburgh, Edinburgh, UK.; ^3^ Earthwave, Codebase, Office L2, 3 Lady Lawson St, Edinburgh, UK.; ^4^ ENVEO IT GmbH, Innsbruck 6020, Austria.; ^5^ Jet Propulsion Laboratory, California Institute of Technology, Pasadena, CA, USA.; ^6^ Department of Soil, Water, and Climate, University of Minnesota, St. Paul, MN, USA.; ^7^ ESA-ESRIN, Largo Galileo Galilei 1, 00044 Frascati, Italy.

## Abstract

Antarctic ice shelves moderate the contribution of the Antarctic Ice Sheet to global sea level rise; however, ice shelf health remains poorly constrained. Here, we present the annual mass budget of all Antarctic ice shelves from 1997 to 2021. Out of 162 ice shelves, 71 lost mass, 29 gained mass, and 62 did not change mass significantly. Of the shelves that lost mass, 68 had statistically significant negative mass trends, 48 lost more than 30% of their initial mass, and basal melting was the dominant contributor to that mass loss at a majority (68%). At many ice shelves, mass losses due to basal melting or iceberg calving were significantly positively correlated with grounding line discharge anomalies; however, the strength and form of this relationship varied substantially between ice shelves. Our results illustrate the utility of partitioning high-resolution ice shelf mass balance observations into its components to quantify the contributors to ice shelf mass change and the response of grounded ice.

## Teaser

Many Antarctic ice shelves are losing mass due to thinning and retreat, resulting in accelerated ice flow towards the ocean.

## INTRODUCTION

Ice shelves fringe the majority of the fast-flowing parts of the Antarctic Ice Sheet (AIS) (*1*) and exert a critical control on the rate of ice discharge into the ocean through a process known as “buttressing” (*2*, *3*). Ice shelf thinning (*4*, *5*) or retreat (*6*–*9*) can reduce the buttressing force provided by the ice shelf, leading to an increase in the speed of the upstream grounded ice (*10*) and an increase in the ice sheet contribution to global sea level rise. For example, in the Amundsen Sea Embayment of West Antarctica, decadal variations in ice shelf basal melt rates and consequent changes in ice shelf thickness have caused large increases in grounding line discharge (*11*–*15*), which has been exacerbated in recent years by a sequence of major calving events at Pine Island Ice Shelf (*8*). The disintegrations of the Larsen B, Larsen A, and Prince Gustav Channel ice shelves were followed by a multiyear acceleration and thinning of their tributary glaciers (*10*, *16*–*19*). At the ice sheet scale, the observed spatial patterns of grounded ice speed change in recent decades can be reproduced by ice flow models that are forced only by the observed change in ice shelf thickness (*4*, *5*). Modeling studies have also examined hypothetical scenarios including complete ice shelf loss, which results in large-scale ice sheet destabilization (*20*, *21*), confirming the importance of ice shelves for stabilizing large portions of the AIS.

Ice shelves are one of the most vulnerable parts of the AIS to changes in atmospheric and ocean conditions. They are low-elevation plains that experience widespread and often intense surface melting (*22*–*26*). Vertical drainage of ponded surface meltwater can cause ice shelf flexure (*27*, *28*) and drove, in combination with other factors, the rapid fragmentation and collapse of the Larsen B Ice Shelf in 2002 (*18*, *29*). Ice shelf surface melting is projected to intensify this century (*30*), which may lead to more widespread and more frequent meltwater ponding, potentially increasing the risk of ice shelf disintegration (*31*). Ice shelves also have large ice-ocean interfaces where basal melting and refreezing occur (*32*), which can affect ice shelf stability and enhance calving (*33*, *34*). Changes in sea ice conditions, combined with ocean swell, currents, tides, and ocean surface slope, can lead to calving from or disintegration of ice shelves (*35*, *36*).

The export of solid and liquid freshwater from ice shelves affects water column hydrography (*37*, *38*), sea ice extent (*39*), and bottom water formation (*40*, *41*), with feedback on the ice shelf (*42*). Some estimates of ice shelf freshwater export exist (*1*, *33*, *43*); however, these generally provide only temporal snapshots or short time series of freshwater export and have limited accounting of ice shelf area changes (*7*, *33*). Therefore the magnitude, timing, spatial distribution, and phase of these freshwater inputs are not known in detail, resulting in widely varying approaches to represent freshwater perturbations in ocean circulation models and consequently diverging conclusions regarding the effect of Antarctic meltwater on, for example, sea ice extent (*39*, *44*).

Each of the factors outlined above makes ice shelves a key pillar in ice sheet–climate interactions. In recognition of the importance of ice shelves, there is a burgeoning literature documenting and investigating ice shelf thickness changes (*15*, *45*–*49*), ice shelf area changes and calving (*7*, *50*–*52*), changes in grounding line discharge (*13*, *53*, *54*), or grounding line migration (*55*, *56*) at one or many ice shelves. Despite these efforts, the components of ice shelf change have rarely been viewed together to provide a clear and coherent picture of ice shelf mass changes during the satellite era (*7*, *46*), which hinders efforts to model the processes that drive ice shelf mass change and their impact on grounded ice (*20*, *57*, *58*). Therefore, it is essential to better quantify ice shelf freshwater export, ice shelf mass changes, and its components and importance for buttressing of grounded ice. Here, we make use of high-resolution satellite datasets to produce an annual record of ice shelf mass balance and its constituent components for all Antarctic ice shelves from 1997 to 2021.

## RESULTS

### Ice shelf freshwater export

**Fig. 1.**
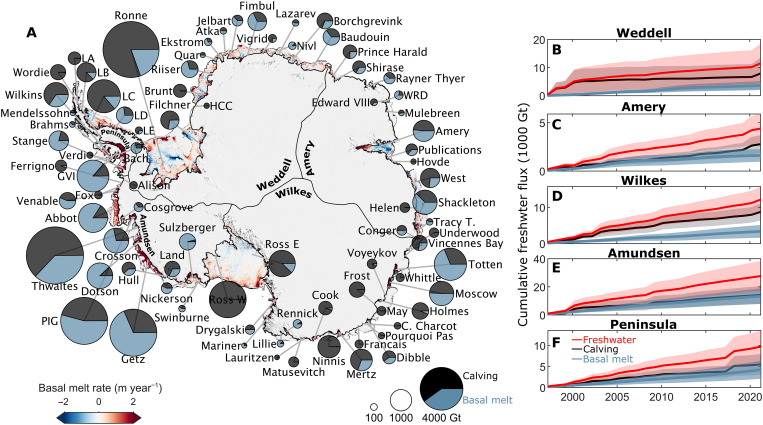
Pan-Antarctic ice shelf freshwater flux. (**A**) Cumulative ice shelf freshwater flux from 1997 to 2021 overlain on the 2010–2021 average ice shelf basal melt rates and a 750-m Moderate Resolution Imaging Spectroradiometer (MODIS) image mosaic (*102*). Only ice shelves with a freshwater flux greater than 50 Gt are plotted. (**B** to **F**) Regional ice shelf cumulative freshwater flux time series. LA, Larsen A; LB, Larsen B; LC, Larsen C; LD, Larsen D; LE, Larsen E; HCC, Hayes Coats Coast; WRD, Wilma Robert Downer; Tracy T., Tracy Tremenchus; C. Charcot, Commandant Charcot; PIG, Pine Island Glacier; GVI, George VI.

We provide new, annually resolved estimates of freshwater export from each of Antarctica’s ice shelves from 1997 to 2021 (Fig. 1). We draw on annual calving observations (*7*, *51*) and high-resolution satellite-derived estimates of ice shelf basal melt rates (*59*–*61*), integrated over time-varying ice shelf masks (see Materials and Methods). We note that we have not included subglacial melt fluxes or surface runoff in these freshwater flux estimates.

We find that Antarctic ice shelves exported 67,000 ± 3200 billion tonnes (Gt) of freshwater to the Southern Ocean from 1997 to 2021, or 2680 ± 580 Gt year^−1^ on average. Solid ice (calving) provided 60% of the pan-Antarctic freshwater export from 1997 to 2021 and over half the freshwater export for 72% of individual ice shelves. However, pan-Antarctic calving fluxes (1600 ± 520 Gt year^−1^ on average) were highly variable from year to year, with an SD of 1150 Gt. Therefore, although the liquid freshwater contribution was lower on average (1080 ± 210 Gt year^−1^), it provided the majority of the pan-Antarctic freshwater export during almost half our study period.

We find no significant trend in pan-Antarctic freshwater flux or its components. Overall, there is a weak (−50 Gt year^−1^) but insignificant negative trend in the pan-Antarctic freshwater flux from 1997 to 2021, which is almost entirely controlled by the calving of icebergs A38, A39, A43, and A44 from the Ronne Ice Shelf near the beginning of our time series. As these calving events are an expected phase of the cyclical advance and retreat of Ronne Ice Shelf, rather than a signal of sustained retreat, we do not expect this pan-Antarctic negative calving trend to be applicable over timescales spanning multiple cycles of ice shelf advance and retreat. We note that these large calving events are not masking calving trends at other smaller ice shelves: Only one ice shelf (Getz) has a significant (*P* < 0.05) positive calving trend through time, but it is modest (3% of its time-averaged calving flux).

### Pan-Antarctic ice shelf mass change

**Fig. 2.**
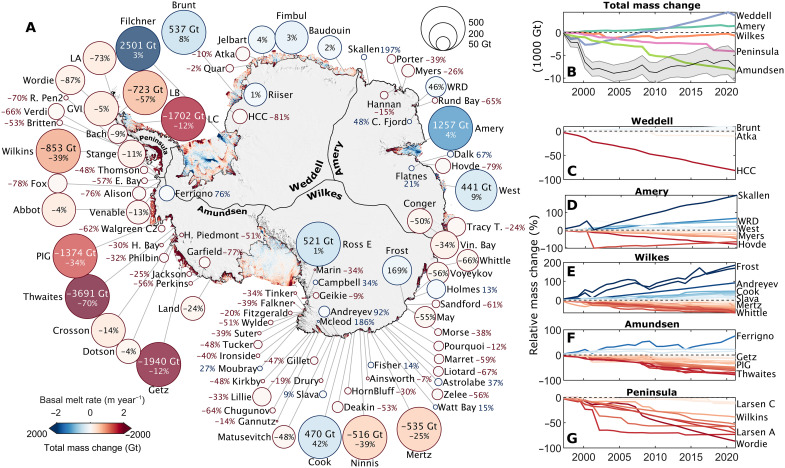
Pan-Antarctic ice shelf mass change. (**A**) Cumulative ice shelf mass change from 1997 to 2021 overlain on the 2010–2021 average ice shelf basal melt rates and a 750-m MODIS image mosaic (*102*). Circle area is capped at 500 Gt and only ice shelves with significant mass change are plotted. (**B**) Ice shelf mass change time series for each labeled region and for Antarctica (black). (**C** to **G**) Relative ice shelf mass change time series for individual ice shelves colored as in (A). LA, Larsen A; LB, Larsen B; LC, Larsen C; LD, Larsen D; LE, Larsen E; HCC, Hayes Coats Coast; WRD, Wilma Robert Downer; Tracy T., Tracy Tremenchus; C. Charcot, Commandant Charcot; PIG, Pine Island Glacier; GVI, George VI; Vin. Bay, Vincennes Bay; H. Piedmont, Hamilton Piedmont; H. Bay, Harmon Bay; Walgreen C2, Walgreen Coast 2; E. Bay, Eltanin Bay; R. Pen2, Rydberg Peninsula 2.

**Fig. 3.**
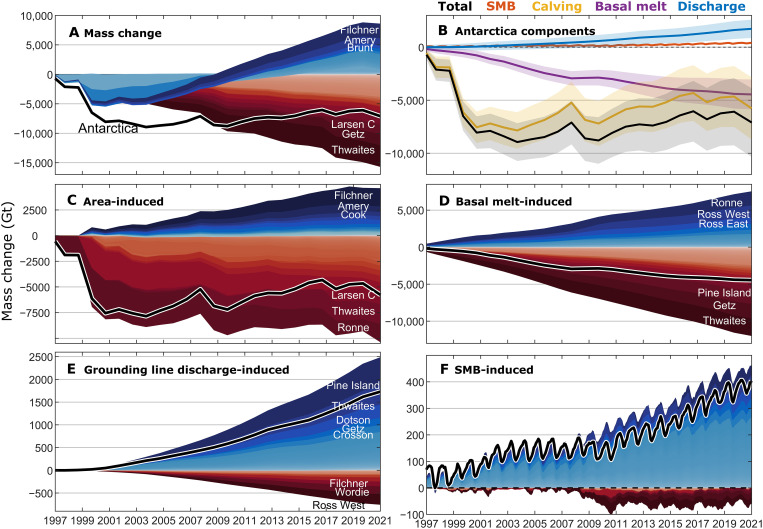
Partitioned ice shelf mass change time series. Stacked time series of cumulative ice shelf (**A**) total mass change, (**C**) mass change due to calving, (**D**) mass change due to basal melting, (**E**) grounding line discharge anomalies relative to 1997 values, and (**F**) SMB anomalies relative to the 1979–2008 climatological mean, with some of the dominant ice shelves for each component labeled. All ice shelves are shown, ordered by contribution to the total for each variable. Negative values (shown in red colors) indicate the overall mass loss or a contribution to mass loss, and the black line shows the pan-Antarctic total. (**B**) Pan-Antarctic time series for each of the budget components.

**Fig. 4.**
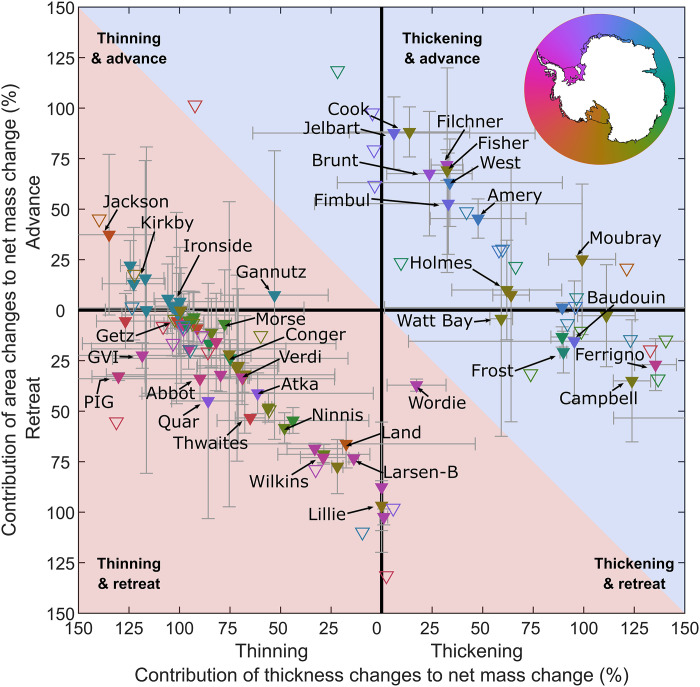
Ice shelf mass changes due to area and thickness changes. The contribution of time-integrated area change and basal melt–induced thickness change to the mass change of each ice shelf. Each point represents an ice shelf that has gained mass (background blue shading) or lost mass (background red shading) overall from 1997 to 2021. The symbol color indicates the ice shelf centroid longitude. Ice shelves with significant mass change are indicated by solid fill symbols and error bars (gray whiskers). Note that the percentage contributions do not sum to 100 because discharge, SMB anomalies, and grounding line migration also contribute to the overall mass change.

**Fig. 5.**
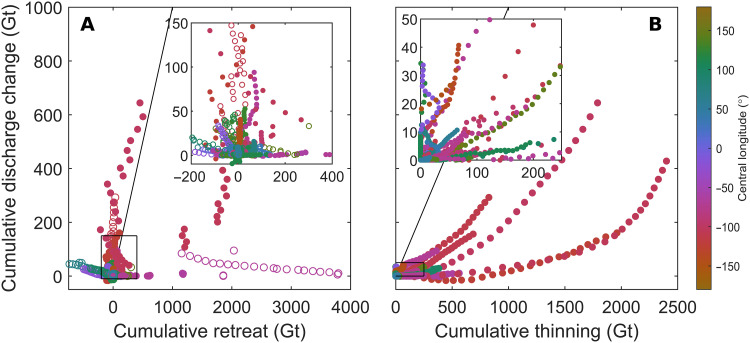
Relationships between cumulative retreat, thinning, and discharge change. (**A**) The relationship between time-dependent cumulative ice shelf retreat and cumulative grounding line discharge change from 1997 to 2021. (**B**) The equivalent of (A) but for basal melt–induced thinning. Each point represents the cumulative mass change up to each year in the time series (note that the points are not necessarily ordered chronologically). Only shelves with net discharge increases are plotted and shelves with significant (*P* < 0.05) positive (*R* > 0.25) relationships between cumulative retreat or thinning and discharge change are plotted with filled markers.

We quantify the mass change of all of Antarctica’s ice shelves annually and in total for the period 1997 to 2021 (Fig. 2) by combining the freshwater export time series described above with high-resolution satellite-derived estimates of grounding line discharge (see Materials and Methods), surface mass balance (SMB) from three regional climate models (*62*–*65*), and grounding line retreat in the Amundsen Sea Embayment (*56*) (see Materials and Methods). We explore absolute and relative ice shelf mass change (Fig. 2) before partitioning the mass budget into its constitutive components (see Materials and Methods, Figs. 3 and 4, and text S1) and examining the relationship between observed discharge changes with ice shelf area and thickness changes (Fig. 5).

Overall, we find that pan-Antarctic ice shelf mass decreased by 7500 ± 1500 Gt from 1997 to 2021 (Fig. 2). In terms of total mass change, much of this continent-wide signal is dominated by large reductions in the mass of Thwaites, Getz, Larsen C, and Pine Island ice shelves and large increases in the mass of Filchner, Amery, and Brunt ice shelves [note that our survey includes the calving of iceberg A74 on 26 February 2021 (*66*) but ends before the long-awaited calving of iceberg A81 from Brunt Ice Shelf on 22 January 2023]. Nevertheless, ice shelf mass loss was widespread around Antarctica: 71 out of 162 ice shelves lost mass. In keeping with a similar study (*45*), most of these ice shelves are located on the Antarctic Peninsula, in Victoria Land and Wilkes Land, and along the coastlines of the Amundsen and Bellingshausen Seas. Only 29 ice shelves gained mass from 1997 to 2021, and these are generally concentrated in Dronning Maud Land, the eastern Weddell Sea coastline, and around the Amery Ice Shelf. Many (*62*) ice shelves exhibited no measurable mass change (that is, their mass change was not significantly different from zero after accounting for errors), though many of these seemingly stable ice shelves have fluctuated in mass (see supplementary figures for each ice shelf).

Pan-Antarctic ice shelf mass decreased rapidly from 1997 to 2002 (Figs. 2B and 3), largely due to the calving of icebergs A38 and A39 in October 1998 and icebergs A43 and A44 in 2000 from Ronne Ice Shelf (*67*, *68*), followed by a retreat of Thwaites Ice Tongue and Mertz Ice Tongue in 2002 (*69*, *70*). The steady advance of Ronne, Filchner, Amery, and Cook ice shelves subsequently caused pan-Antarctic ice shelf mass gain from 2002 to 2021 (Figs. 2B and 3), interrupted only by large calving events from Thwaites in 2012 and 2017 (*69*), Larsen C in 2017 (*71*), and Ronne Ice Shelf in 2021 (*7*). The overall increase in ice shelf mass since 2002 should not be interpreted as a sign of widespread ice shelf recovery around Antarctica. In contrast, we find that 68 ice shelves have a significant (*P* < 0.05) negative mass trend from 1997 to 2021, of which 26 are greater than −2% per year, and that 47 ice shelves have lost more than 30% of their mass since 1997 (Fig. 2).

### Ice shelf mass budget partitioning

Pan-Antarctic ice shelf mass loss from 1997 to 2021 was due to both basal melt–induced thinning (−4480 ± 1420 Gt) and terminus retreat (−6200 ± 700 Gt), which were partially offset by positive SMB anomalies (340 ± 90 Gt), groundling line retreat (1070 ± 170 Gt), and grounding line discharge increase (1770 ± 870 Gt) (see and Materials and Methods, Fig. 3, and figs. S1 to S4). As with ice shelf mass changes, these pan-Antarctic totals for each mass budget component have substantial contributions from a small number of ice shelves. For example, the three ice shelves that retreated most (Ronne, Thwaites, and Larsen C) contributed −5170 ± 470 Gt to the pan-Antarctic terminus retreat. Similarly, the three ice shelves with the greatest basal melt–induced thinning (Thwaites, Getz, and Pine Island) contributed 6150 ± 420 Gt to pan-Antarctic basal melt–induced thinning (noting that some ice shelves underwent net basal melt–induced thickening).

To gain insight into the relative importance of basal melting and calving in driving ice shelf mass change, we examine the contributions of each component to the observed mass change of each ice shelf individually (Fig. 4). By normalizing by ice shelf mass change in this way, we find that basal melt–induced thinning was typically the dominant contributor to a mass loss for individual ice shelves. Of the 71 ice shelves that unambiguously lost mass, basal melt–induced thinning accounted for more than 50% of that mass loss for 37 to 52 ice shelves (with a central estimate of 48), with the range due to uncertainties in the mass budget partitioning (text S1). Cumulative grounding line discharge anomalies relative to 1997 values have contributed an additional 1770 ± 870 Gt of ice input to Antarctic ice shelves since 1997 (Fig. 3E and fig. S1). This is primarily due to large increases in the velocity of ice streams draining into the Amundsen Sea Embayment ice shelves (Pine Island, Thwaites, Crosson, Dotson, and Getz) but also due to discharge increases at 21 other ice shelves, including Ainsworth, Publications, Ninnis, Frost, Dibble, and Hull (fig. S1). Grounding line discharge decreased at nine ice shelves (Fig. 3E), resulting in 540 ± 310 Gt of reduced mass input to those ice shelves. In some cases, decreases in discharge were due to the deceleration of ice flow, such as at Ross West. For Larsen B and Wordie ice shelves, however, the discharge reduction was instead due to ice shelf collapse and a commensurate reduction in the length of the grounding line connected to the ice shelf (see Materials and Methods).

The contribution of SMB anomalies to pan-Antarctic ice shelf mass change was modest but nonnegligible (340 ± 90 Gt). For some ice shelves, SMB anomalies contributed notably to their mass change (fig. S4). For example, Nivl, Ekström, Baudouin, and Fimbul ice shelves in Dronning Maud Land gained mass, to which SMB anomalies contributed 18 ± 14%, 17 ± 7%, 14 ± 5%, and 12 ± 5%, respectively. Similarly, SMB made notable contributions to the mass loss of some ice shelves, including Moscow University (9 ± 6%) and Totten (10 ± 4%) ice shelves in East Antarctica.

## DISCUSSION

### Relationships between ice shelf mass change and grounding line discharge

We observe widespread decreases in Antarctic ice shelf mass (Fig. 2) and widespread increases in grounding line discharge since 1997 (fig. S1). Of the 71 ice shelves that lost mass overall during the study period, only a third (*26*) also experienced an overall, significant (defined as *R*^2^ > 0.5 and *P* < 0.05) increase in grounding line discharge. The lack of a significant grounding line discharge increase at the other 45 ice shelves suggests that the observed mass loss from those ice shelves has not caused a significant change in buttressing, possibly where mass losses are dominated by basal melt–induced thinning in locations that provide little buttressing (*5*), or by the calving of “passive ice” where ice flow is extensional (*4*, *6*, *72*).

Modeling studies (*6*, *7*, *73*) suggest that grounding line discharge can increase due to retreat or thinning, or both combined, which does not necessarily require an overall reduction in ice shelf mass. We therefore compare grounding line discharge anomalies relative to 1997 values to basal melt–induced thinning and retreat individually (Fig. 5). In the following, we compare cumulative anomalies in each budget component because they are more sensitive to small but sustained changes in mass flux. We note that if anomalies in any budget component are positive but constant in time, then the cumulative anomaly will increase linearly over time. Thus, if the anomalies increase linearly in time, then the cumulative anomaly will increase quadratically (or decrease quadratically in the case of negative anomalies).

In general, we find that both cumulative retreat and/or cumulative basal melt–induced thinning are associated with an increase in grounding line discharge (Fig 5, A and B). To illustrate, cumulative discharge anomaly time series are significantly positively correlated with retreat time series at 48 ice shelves (Fig. 5A) and with basal melt–induced thinning time series at 57 ice shelves (Fig. 5B), of which 25 also retreated. However, we emphasize that the strength and form of these relationships vary substantially between ice shelves, reflecting differing sensitivities of ice shelves to thinning and retreat as well as changes in that sensitivity over time due to, for example, detachment from pinning points. In addition, 18 ice shelves had significant positive cumulative discharge trends but did not have a positive relationship with either cumulative basal melt–induced thinning or retreat, which could indicate either uncertainty in the respective time series or a prolonged discharge response to a single calving or melt event (fig. S5).

The interpretation of the observed relationships between basal melt–induced thinning, retreat, and cumulative discharge change is more complicated than for similar relationships derived from diagnostic modeling experiments (*4*–*7*). These modeling experiments isolate the effect of calving and melting and typically ignore longer-term discharge changes due to ice mass redistribution (*74*), grounding line migration (*75*, *76*), or geometry-induced changes to ocean circulation in ice shelf cavities (*42*, *77*), which aids interpretation of model output and facilitates disentangling the effects of individual processes. Our observations permit a different perspective that integrates the effects of both the instantaneous and transient discharge response to a change in ice shelf buttressing plus any internal and ice-ocean feedbacks that operate over timescales of less than 25 years. To illustrate, there are several ice shelves, such as Andreyev, Clarke Bay, Dalk, and Hull, at which a single or series of calving events were immediately followed by sustained increases in grounding line discharge despite terminus readvance (fig. S5). This integrated response makes grounding line discharge observations at annual temporal resolution less useful for disentangling the contributions of thinning, retreat, and internal feedback to the observed grounded ice response or for informing physical representations of those relationships in models. Nevertheless, we suggest that this integrative quality makes them well-suited for informing simpler representations of the process- and time-integrated relationship between ice shelf deterioration and grounding line discharge, which may supplement high-fidelity but computationally expensive physically based ice-ocean models (*78*).

## Summary

We show that Antarctic ice shelves have exported 67,000 ± 3200 Gt of freshwater to the Southern Ocean from 1997 to 2021, of which solid ice (calving) provided 60% and which has been fairly constant on annual timescales since 1997 other than spikes in solid ice export from large calving events. These spatially and temporally resolved freshwater flux estimates could be used to inform modeling investigations seeking to constrain any emerging impacts of Antarctic freshwater export on Southern Ocean circulation, biogeochemical cycling, and ecological productivity, compared to other observationally constrained climate forcings, such as changes in sea ice extent and formation rates.

Building on previous studies (*7*, *45*), we show that many Antarctic ice shelves deteriorated significantly from 1997 to 2021 and that the vast majority of those have significant mass loss trends. Pan-Antarctic ice shelf mass decreased overall by 7500 ± 1500 Gt during the 1997–2021 period due to both terminus retreat (6200 ± 700 Gt) and basal melt–induced thinning (4480 ± 1420 Gt), which were partially offset by increases in grounding line discharge (1770 ± 870 Gt), SMB (340 ± 90 Gt), and grounding line retreat (1070 ± 170 Gt). Ice shelf mass loss was regionally concentrated along the Antarctic Peninsula, the Amundsen Sea, and Bellingshausen Sea coastlines in West Antarctica, and in Wilkes Land and Victoria Land in East Antarctica. Pan-Antarctic ice shelf mass has increased since 2002 due to the steady advance of a small number of large ice shelves, but this growth masks significant and sustained reductions in the mass of many Antarctic ice shelves. Basal melt–induced thinning was the dominant mass loss term for a slight majority (52 to 73%) of ice shelves that lost mass. Both basal melt–induced thinning and retreat were significantly positively correlated with grounding line discharge change at many ice shelves, but there was a wide range in the strength and form of the relationship between ice shelves. These new observations provide a more detailed picture of the health of Antarctic ice shelves and drivers of ice shelf mass change than was previously available and highlight the wide-ranging relationships between ice shelf mass change and grounding line discharge change.

## MATERIALS AND METHODS

### Ice shelf masks

We generate quasi-annual masks for all 162 ice shelves around Antarctica from 1997 to 2021. These 162 ice shelves are composed of up to 186 ice shelf units, but we amalgamate some shelf units into single ice shelves, as has been done previously (*7*). The number of ice shelf units changes over time due to ice shelf disintegration into multiple smaller units (for example, at Larsen B and Wordie). To generate these annual masks, we use a grounding line derived from BedMachine v2 (*79*) ice thickness and bed elevation or the MEaSUREs grounding line (*80*) where there is no clear difference between the two products. We combine this static grounding line with quasi-annual coastlines from 1997 to 2021 (*7*).

To create complete masks from these separate products, we combine them in the following way. For each ice shelf, first, we clip the BedMachine or MEaSUREs grounding line using previously published ice shelf masks (*1*) to create a separate grounding line for each ice shelf. Second, we modify the coastlines of (*7*) to follow the internal boundary of islands that intersect the edge of ice shelves. This becomes important when integrating SMB and basal melt rate estimates within each ice shelf mask and is particularly important for ice shelves such as Wilkins, which detach from coastal islands during the study period that would otherwise cause apparent changes in SMB or basal melt fluxes. For most ice shelves, we join the grounding line to the coastline either using the point of intersection with the coastline or, if there is no intersection, by joining the grounding line with the nearest coastline point each year. For some ice shelves that are connected to the coastline dataset via islands that are not included in the grounding line or coastline data, we instead use the MEaSUREs islands dataset as a bridge. Similarly, for Ross East and Ross West, we use an existing delineation (*1*) to connect our grounding line to the quasi-annual coastlines. Last, islands that are internal to each ice shelf are removed, again, to avoid integrating SMB and basal melt fluxes over areas that are irrelevant to the mass of the ice shelf.

We make some further modifications to the Thwaites Glacier Ice Shelf due to the difficulty of accurately delineating the calving front of this ice shelf in satellite imagery, resulting in large (tens of kilometers) differences between different datasets (*7*, *69*, *81*, *82*). From 2009 to 2019, we used higher-resolution delineations of the calving front derived from Moderate Resolution Imaging Spectroradiometer (MODIS) imagery at 500 × 500 m resolution (*51*). This resulted in a large (2777 km^2^) reduction in ice shelf area in 2009 caused by switching datasets; however, this is ice area that had already calved in previous years and the majority of the area reduction occurs because the coarser coastline dataset (used before 2009) simplifies the shear zone between the eastern and western ice shelves. Therefore, this dataset switch essentially amalgamates some of the true calving flux that had happened over the preceding years into 2009. It is therefore probable that we overestimate the calving flux in 2009, but that the overall calving flux during the study period is closer to the true calving flux.

### Grounding line discharge

We calculate ice discharge across the grounding line of each ice shelf from 1997 to 2021. We define each flux gate at 200-m increments on an Antarctic Polar Stereographic grid (EPSG 3031).

We estimate grounding line discharge, *D*, across each flux gate pixel as

D=VHwρ

where *V* is the gate-normal ice velocity, *H* is the ice equivalent thickness, *w* is the pixel width, and ρ is ice density (917 kg m^−3^).

The gate-normal ice velocity is given by

$$V=\sin(\theta )V_{x}-\cos(\theta )V_{y}$$

where *V_x_* and *V_y_* are the easting and northing components of the horizontal ice velocity, as defined by the South Polar Stereographic grid (EPSG3031), respectively, and θ is the angle of the flux gate relative to the same grid. Ice velocity data are compiled from multiple published and freely available sources. From 1997 to 2018, we used 240 × 240 m ITS_LIVE annual mosaics (*54*). From 2000 and 2005 to 2016, we used 1 × 1 km MEaSUREs annual velocity mosaics (*83*, *84*). We also used a MEaSUREs velocity mosaic incorporating velocity estimates between 1995 and 2001, at 450 × 450 m resolution (*85*). From 2015 to 2021, we used 200 × 200 m monthly velocity mosaics derived from intensity tracking of Sentinel-1 image pairs (*86*) (https://cryoportal.enveo.at/data/). In the Amundsen Sea Embayment, we additionally use velocity estimates in 1996 to constrain discharge at the beginning of our time series. The 1996 velocities are a combination of 450 × 450 m MEaSUREs InSAR-based estimates derived from 1-day repeat ERS-1 imagery (*13*, *87*), which covers the region spanning Cosgrove to Kohler Glacier, and 200 × 200 m velocities from ERS offset tracking over the Getz basin (https://cryoportal.enveo.at/data/), which have been filled using the optimized BISICLES ice sheet model (*88*). Each of these velocity products spans a time period; following (*89*), we treat each product as an instantaneous measurement with the time stamp given by the central date in the estimate. We extract easting and northing velocities at each gate pixel using nearest neighbor interpolation. Treating each gate pixel as a time series, we remove outliers in two stages. First, we remove global time series outliers, which we define as data points with more than five scaled median absolute deviations from the median, after detrending. Second, we remove local outliers, which we define as data points more than three SDs from the signal-to-noise ratio-weighted mean in a 1-year moving window. We fill temporal gaps using a linear interpolation except at the beginning and end of each time series, which are back- and forward-filled with the temporally nearest value for that pixel. For gate pixels with no data at any time, we assume the MEaSUREs reference velocity for epochs before 2015 and a 2015–2021 mean velocity calculated from the annual Sentinel-1 velocity mosaics for later epochs. Last, as in previous studies (*13*, *89*), we assume the depth-averaged velocity is the same as the measured surface velocity.

We create a baseline thickness estimate from a reference bed topography and ice surface dataset. To create our reference bed dataset, we primarily use BedMachine v2 bed topography (*79*, *90*). We replace the BedMachine bed with a more recent estimate in Princess Elizabeth Land (*91*) and with a dedicated bed topography dataset over the Antarctic Peninsula (*92*), after conversion to a common geoid (g104c). We combine these bed topography datasets with the 200-m Reference Elevation Model of Antarctica (REMA) digital elevation model (*93*) (DEM) to define our baseline thickness estimate.

Starting with this baseline thickness estimate, we estimate the time-varying ice thickness, and therefore discharge, at the time stamp of each velocity measurement using a fixed bed elevation and a time-varying ice surface. The assumption of a fixed bed means that we neglect any changes in bed elevation due to erosion, basal melt, or uplift, which we expect to be at least an order of magnitude smaller than the observed ice surface elevation changes. To vary the reference REMA ice surface, we use time-varying ice equivalent thinning rates from 1985 to 2020 derived from a constellation of satellite missions (*94*), smoothed with a 5-year boxcar filter, assuming that the REMA DEM is time-stamped to 9 May 2015 (*93*). Ice equivalent thickness is estimated at each velocity epoch by removing a time-varying firn air content, provided by the Institute for Marine and Atmospheric research Utrecht IMAU firn densification model (*95*, *96*), forced by RACMO2.3p2 (*62*). The total grounding line discharge at each ice shelf is estimated by integrating the pixel-based discharge estimate for all flux gate pixels for that ice shelf.

At the majority of ice shelves, we assume a static grounding line throughout our study period for this discharge calculation. At most ice shelves, this simplification is justifiable because the changes in grounding line position are sufficiently small, even at shelves with large grounding line retreats such as Pine Island Glacier, that the additional mass changes due to basal melting, SMB, and divergence between the chosen grounding line dataset and true grounding line are small compared to changes in discharge caused by thickness and velocity changes. That is, the use of a static grounding line does not inhibit the measurement of discharge changes across the true grounding line.

We do use a time-varying grounding line and flux gate at Wordie and Larsen B because these ice shelves have broken into smaller ice shelf units during the study period, so there are large sections of the original grounding line that no longer have an attached ice shelf. It would not therefore be appropriate to calculate the discharge across those segments of the grounding line for our calculations of ice shelf mass balance. We therefore modify the grounding line annually so that we only include portions of the grounding line that have a downstream intact ice shelf each year.

### Discharge error

We define our discharge error, *D*_σ_, following (*89*) as

$$D_{\sigma }=\sqrt[{2}]{V_{\sigma }^{2}+H_{\sigma }^{2}}$$

where *V*_σ_ is the velocity-induced discharge errors and *H*_σ_ is the thickness-induced discharge errors. Both sources of discharge error are time-stamped and calculated at each flux gate pixel. Where available, we use the easting and northing velocity errors provided in each velocity product. Where we have interpolated the velocity, we define the error as 10% of the estimated easting and northing velocity components in each time-stamped pixel. We combine the easting and northing velocity errors normal to the flux gate through quadrature. Similarly, the thickness errors are defined as the sum through quadrature of the reference bed elevation error and the error in the time-stamped surface elevation estimate. We assume a 1-m error in the baseline REMA 200-m DEM, which is equivalent to the 90th percentile of the errors in the mosaic (*93*) and a 0.1–m year^−1^ error in the applied surface elevation change (*97*).

### Surface mass balance

We estimate the SMB of each ice shelf at monthly time resolution using output from three regional climate models: the Regional Atmospheric Climate Model (RACMO2.3p2) at 27 × 27 km resolution, the Modèle Atmosphéric Régional (*63*, *64*) at 35 × 35 km resolution, and the Danish regional climate model HIRHAM5 (*65*) at 0.11° resolution. For each ice shelf, we calculate the area of each ice shelf, accounting for distortion induced by the Polar Stereographic grid, and integrate the modeled SMB from each climate model individually, before averaging them. For some small ice shelves, no regional climate model pixels intersect the shelf; for these ice shelves, we simply use the nearest available pixel to the centroid of that ice shelf. For the three-model mean SMB time series, we assume an error of 14.8% in the SMB everywhere, which is the upper limit for the SMB error suggested in (*98*).

### Basal melt

We estimate ice shelf basal melt fluxes using a combination of two basal melt rate datasets. Where observational coverage permits (82 ice shelves), we estimate basal melt fluxes from January 2010 to January 2021 at monthly temporal resolution using swath-mode CryoSat-2 observations (*60*, *61*). For the other ice shelves and for those 82 ice shelves during the remaining study period, we use quarterly basal melt rate estimates (*59*) posted at 1920 × 1920 m^2^ spatial resolution. We describe the former dataset briefly below, but refer the reader to (*60*, *61*) for details.

The method to derive time series of basal melt rates from CryoSat-2 from 2010 to 2020 follows the mass conservation approach described in (*32*). The monthly elevation change is generated from swath-processed CryoSat-2 radar altimetry (*60*, *61*), the ice shelf mask and ice shelf thickness are from BedMachine Antarctica (*79*), the ice velocity is an ITS_LIVE composite (*99*), and the SMB and firn air content are from RACMO2.3p2 (*62*) and the IMAU firn densification model (*95*, *96*), respectively. For the melt time series, we consider time evolution in ice shelf extent, ice shelf thickness, SMB, and firn air content; during the CryoSat period, changes in ice divergence are negligible. Other than changes in ice shelf extent, time-dependent variables are similar to those in (*59*) for consistency. The melt rate uncertainty accounts for each of the mass conservation terms as described in (*32*).

We fill gaps in the coarser resolution melt rates (*59*) using the following procedure. After stacking the melt rate grids over each ice shelf, we treat each pixel as a time series and linearly interpolate across gaps that are no more than 6 months (i.e., two consecutive missing values). Any remaining gaps are filled using the time-average melt rate for that pixel. For the 2018–2021 period, when no coarse melt rate data are available, we use the median of the 1997–2017 time series of ice shelf-integrated melt rates for each ice shelf. Some ice shelves have no basal melt data at any time in either product; for these ice shelves, we assume no basal melting and no thinning. These ice shelves are Alison, Andreyev, Astrolabe, Cirque Fjord, Commandant Charcot, Eltanin Bay, Falkner, Fox Ice Stream, Francais, Hamilton Piedmont, Hayes Coats Coast, Hovde, Liotard, Marret, Mcleod, Quatermain Point, Rose Point, Rund Bay, Rydberg Peninsula 1 and 2, Sandford, Skallen, Telen, Underwood, and Whittle.

We estimate the total basal melt flux and total melt flux error at each melt epoch by multiplying the average melt rate and melt rate error for each ice shelf by the area of each shelf, assuming an ice density of 917 kg m^−3^. We then smooth the time series of ice shelf-integrated basal melt flux and melt flux error with a 5-year moving mean filter. As with the SMB fluxes, our estimate of the area of each ice shelf accounts for distortion induced by the Polar Stereographic grid and varies quasi-annually using the ice shelf masks. In years without an ice shelf mask, we use the mask from the nearest available year.

### Iceberg calving flux

We make use of published calving flux measurements (*7*); for some ice shelves, we combine individual shelf units from (*7*) to determine the total calving flux for that ice shelf. The total calving flux from an ice shelf can be defined in terms of two components. The first is the steady-state calving flux, which is required to maintain a stationary terminus position. The steady-state calving flux is taken from (*7*) and is assumed to be static throughout our time series, though we note that the true steady-state calving flux will vary through time as the velocity, thickness, and length of the calving front change. The second is the calving flux due to changes in the position of the calving front. Throughout this paper, we present total calving flux, which is the sum of the steady-state calving flux and the calving flux due to changes in the position of the calving front.

For Thwaites Ice Shelf, we use a manual calving front delineation derived from 500 × 500 m MODIS imagery from 2009 to 2019 (*51*) and apply a similar approach for measuring calving flux as in (*7*). That is, we create a reference thickness dataset by combining BedMachine v2 ice thickness (*79*) and the REMA surface elevations (*93*) converted to ice thickness assuming hydrostatic equilibrium. Following (*7*), we extrude the ice thickness data along ice flow lines generated from the ITS_LIVE multiyear velocity mosaic (*99*) to create a gapless estimate of thickness that extends beyond the bounds of the ice shelf. In each year, we calculate the mass of the remaining ice shelf as its area multiplied by the ice thickness and the density of ice, 917 kg m^−3^. The annual mass change due to retreat or advance is then simply the change in ice mass between years. As in (*7*), we estimate the errors in the mass of the ice shelf at each measurement time as the root sum square of the mass error due to uncertainty in the coastline delineations (assumed to be ±1 pixel) and the mass error from uncertainties in the thickness estimate. The error in the calving flux due to area change between two delineations is then the root sum square of the errors in the mass of the shelf at each delineation.

Calving events associated with changes in terminus position are often large, stochastic events. We therefore report all calving flux measurements at the time the terminus position was observed, i.e., if the terminus retreated in one coastline compared to the previous, then we assign the associated calving flux to the time stamp of the latter observation. This means that we perform no interpolation or extrapolation of the calving estimates. In 1998 and 1999, there are no coastline observations—in those years, we assume that the true calving flux equals the steady-state calving flux, and therefore that all calving associated with changes in terminus position between 1997 and 2000 occurred in 2000.

### Grounding line migration

For Pine Island, Thwaites, Crosson, and Dotson ice shelves, we calculate the total ice shelf mass change from 1997 to 2021 due to grounding line migration. This region in West Antarctica was chosen as it represents the majority of known grounding line retreats in Antarctica during our study period. To do this, we calculate the ice shelf area change between the 1996 InSAR-derived grounding lines described in (*56*) and our reference grounding line described above. We convert this area change to a mass change using the area-averaged ice shelf thickness from our reference thickness dataset and an ice density of 917 kg m^−3^. We estimate the errors in this mass change assuming a 1-pixel digitization error and a 30-m error in the ice thickness estimate, which we combine through quadrature. Using this approach, we estimate that grounding line retreat has increased the mass of Pine Island, Thwaites, Crosson, and Dotson ice shelves by 220 ± 40 Gt, 230 ± 25 Gt, 200 ± 25 Gt, and 420 ± 80 Gt, respectively. We note that the mass change of Dotson Ice Shelf due to grounding line retreat will be highly sensitive to the location of the ice divide between Dotson and Crosson ice shelves, which intersects Smith Glacier where substantial grounding line retreat has been measured (*55*, *56*). We do not attempt to calculate this term annually; instead, we only apply the grounding line migration–induced mass change to the total mass change for each ice shelf. We assume that the grounding line of all other ice shelves is static throughout our study period.

### Ice shelf mass balance

To calculate ice shelf mass balance *M*, we combined our time series of grounding line discharge *Q*, basal melting *B*, SMB *S*, and calving *C* to a common time series. To avoid the need to interpolate our calving time series, we first linearly interpolate our discharge time series to the times of the terminus position observations and calculate the annual averages of the basal melt fluxes and SMB, centered on the time of the calving observations. The mass balance of each shelf is then given as

M=Q+S−C−B

which we calculate annually for each ice shelf from 1997 to 2021. Our mass balance estimates therefore represent annual averages, rather than snapshots in time.

Annual mass balance errors are estimated by combining the errors in each budget component through quadrature

$$M_{\mathrm{err}}=\mathrm{sqrt}(Q_{\mathrm{err}}^{2}+S_{\mathrm{err}}^{2}-C_{\mathrm{err}}^{2}-M_{\mathrm{err}}^{2})$$

As these are annual estimates in billion tonnes per year, the cumulative mass change is the simple cumulative sum of the annual values, and cumulative mass change errors for each ice shelf are defined as the root sum square of the annual errors (*88*, *100*). The pan-Antarctic totals of ice shelf mass changes are the sum of that from all individual shelves, and the total mass change errors are the root sum square of the errors from each ice shelf.

### Contributions of mass budget components to overall mass change

We estimate the contributions of changes in grounding line discharge, calving, SMB, and basal melting to the observed ice shelf mass changes. To do this, it is necessary to define so-called steady-state values of each budget component, deviations from which are assumed to cause mass change (*101*). The steady-state value for each component is defined as follows and described in more detail in text S1:

1) Grounding line discharge: the 1997 grounding line discharge and the associated error as described above.

2) Calving: the calving flux required to maintain a stationary terminus position and the associated error, as given in (*7*).

3) SMB: the 1979–2008 mean SMB, with the error given by the SD of 10 different 20-year reference periods in the period 1979–2008.

4) Basal melt: the basal melt flux required to cause zero mass change given the steady-state values defined above, with the errors defined as the sum through quadrature of the errors for the other steady-state components.

We chose to set the steady-state basal melt flux as a function of the other steady-state fluxes, rather than calculating it separately because calculation of the “true” steady-state basal melt flux (that is required to maintain ice shelf thickness) carries the most uncertainty and as otherwise the sum of the contributions would not equal the observed ice shelf mass changes (text S1).

Using these definitions, an increase in grounding line discharge or SMB above the steady-state value contributes an additional mass input to the ice shelf, whereas an increase in calving flux or basal melt flux relative to steady-state values contributes to ice shelf mass decrease (hence retreat or basal melt–induced thinning, respectively). The errors in the annual mass budget component anomalies are estimated by combining through quadrature the annual component mass flux and the error in the associated steady-state estimate. The cumulative anomaly errors are then the root sum square of the annual anomaly errors. The exception to this is the error in the total mass change due to terminus retreat; for which we calculate the total error directly from the errors in the mass of the ice shelf at the start and end of the study period, as given in (*7*). This significantly reduces the total error for this budget component because the errors are not accumulated over time. All contributions quoted in the main text are calculated using the time series of each budget component after interpolation to the calving time series. We compare our basal melt–induced thinning anomalies to previous similar studies in text S2.

We emphasize that these definitions are not necessarily equivalent to the true steady-state mass fluxes in each year (see text S1). For example, the true steady-state calving flux will vary year to year because of variations in the thickness and speed of ice at the calving front, as well as the length of the calving front itself. Similarly, the true steady-state basal melt rate will likely vary depending on the time-varying SMB and flux divergence across the ice shelf. While acknowledging these limitations, we argue that they are appropriate definitions given that our aim is to determine the contributions of each mass budget component to the mass change since 1997 (a time at which some ice shelves were not necessarily in balance). We also note that the calculation of these contributions is completely independent of the mass balance calculations required to calculate annual and total ice shelf mass changes, and as such, our choice of these definitions does not influence our estimates of ice shelf mass balance or overall mass change.

## Supplementary Material

20231012-1

## Notes

**Correction (5 December 2023):** Shortly after publication, the authors alerted the editorial office to a formatting error in table S2 that affected the display of data. The table has been updated and this correction has no effect on the results or conclusions of the paper.

## Supplementary Materials

### This PDF file includes:

Figs. S1 to S174 Table S1 Legend for table S2 Supplementary Text S1 and S2 References
