# Supplementary material for: Annual mass budget of Antarctic ice shelves from 1997 to 2021
Source: Sci Adv. 2023 Oct 12;9(41):eadi0186. doi: 10.1126/sciadv.adi0186 (PMC11650781; doi:10.1126/sciadv.adi0186)
Supplement: Supplementary file 2 — Figs. S1 to S174 Table S1 Legend for table S2 Supplementary Text S1 and S2 References [file sciadv.adi0186_sm.pdf]

CORRECTION POSTED 5 DECEMBER 2023

Science Advances
AAAS

Supplementary Materials for

Annual mass budget of Antarctic ice shelves from 1997 to 2021

Benjamin J. Davison *et al.*

Corresponding author: Benjamin J. Davison, b.davison@leeds.ac.uk

Sci. Adv. **9**, eadi0186 (2023)
DOI: 10.1126/sciadv.adi0186

The PDF file includes:

Figs. S1 to S174
Table S1
Legend for table S2
Supplementary Text S1 and S2
References

Other Supplementary Material for this manuscript includes the following:

Table S2

Supplementary figures

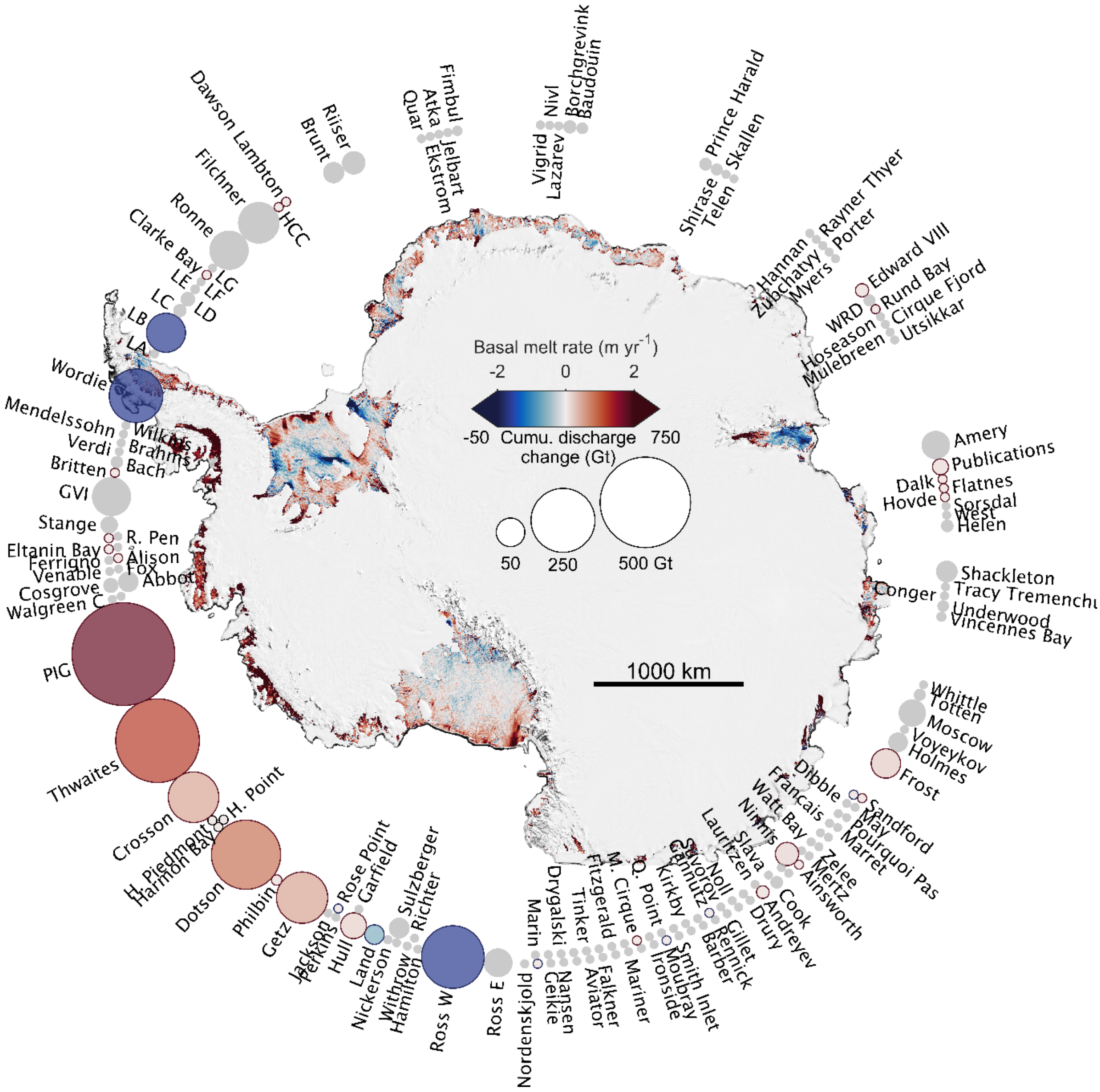

Supplementary Figure 1. Cumulative changes in grounding line discharge. Red circles indicate ice shelves where grounding line discharge has increased from 1997 to 2021, whilst blue circles indicate a decrease in grounding line discharge and grey circles indicate ice shelves with no significant change in grounding line discharge.

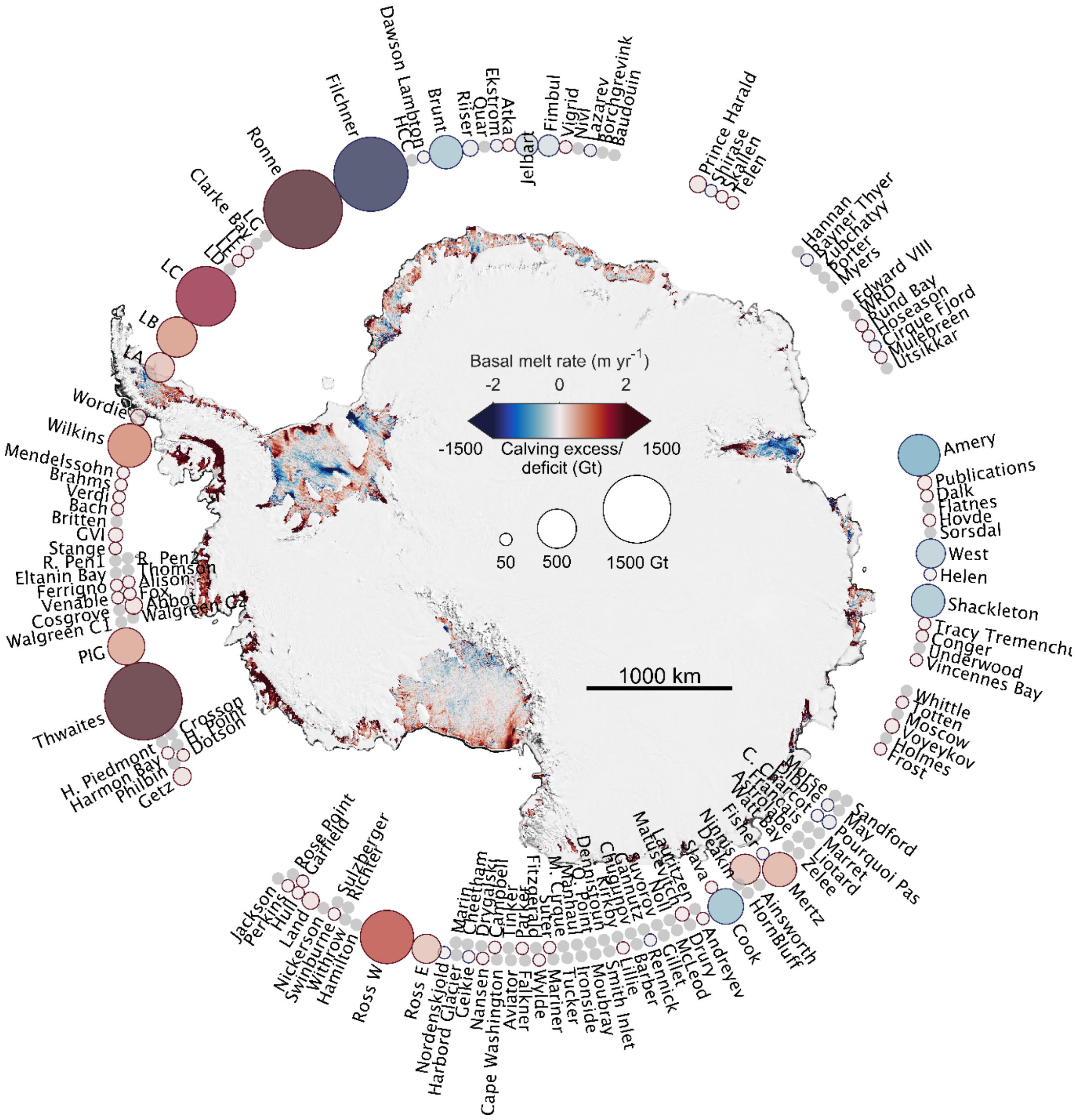

Supplementary Figure 2. Cumulative ice shelf calving flux. Red circles indicate ice shelves with cumulative calving excesses from 1997 to 2021, blue circles indicate shelves with cumulative ice shelf calving deficits and grey circles indicate shelves where cumulative calving fluxes were not significantly different from the steady-state value.

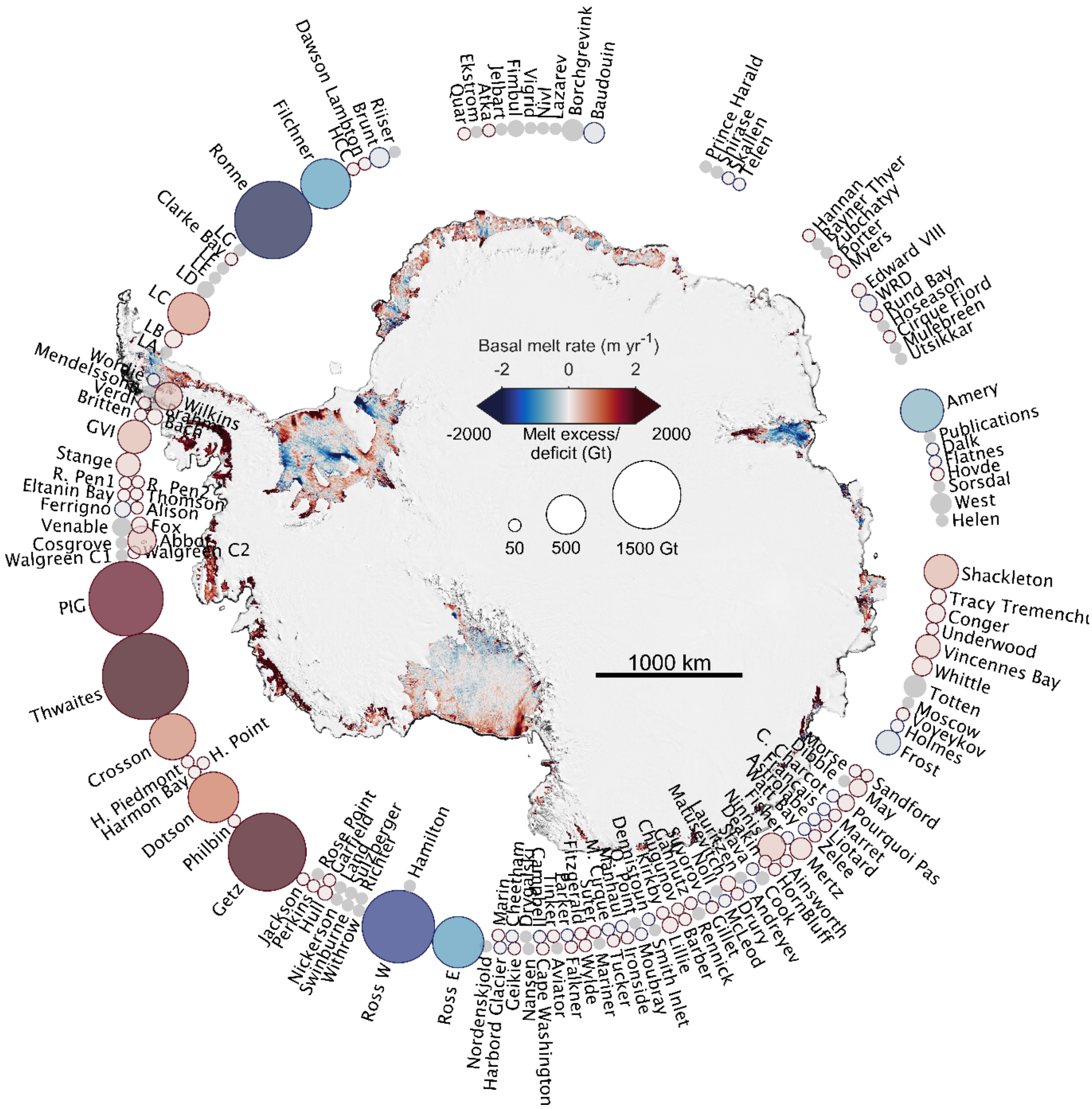

Supplementary Figure 3. Cumulative ice shelf basal melt flux. Red circles indicate ice shelves with cumulative basal melt excesses from 1997 to 2021, blue circles indicate ice shelves with cumulative basal melt deficits and grey circles indicate ice shelves where cumulative basal melt fluxes were not significantly different from the steady-state value.

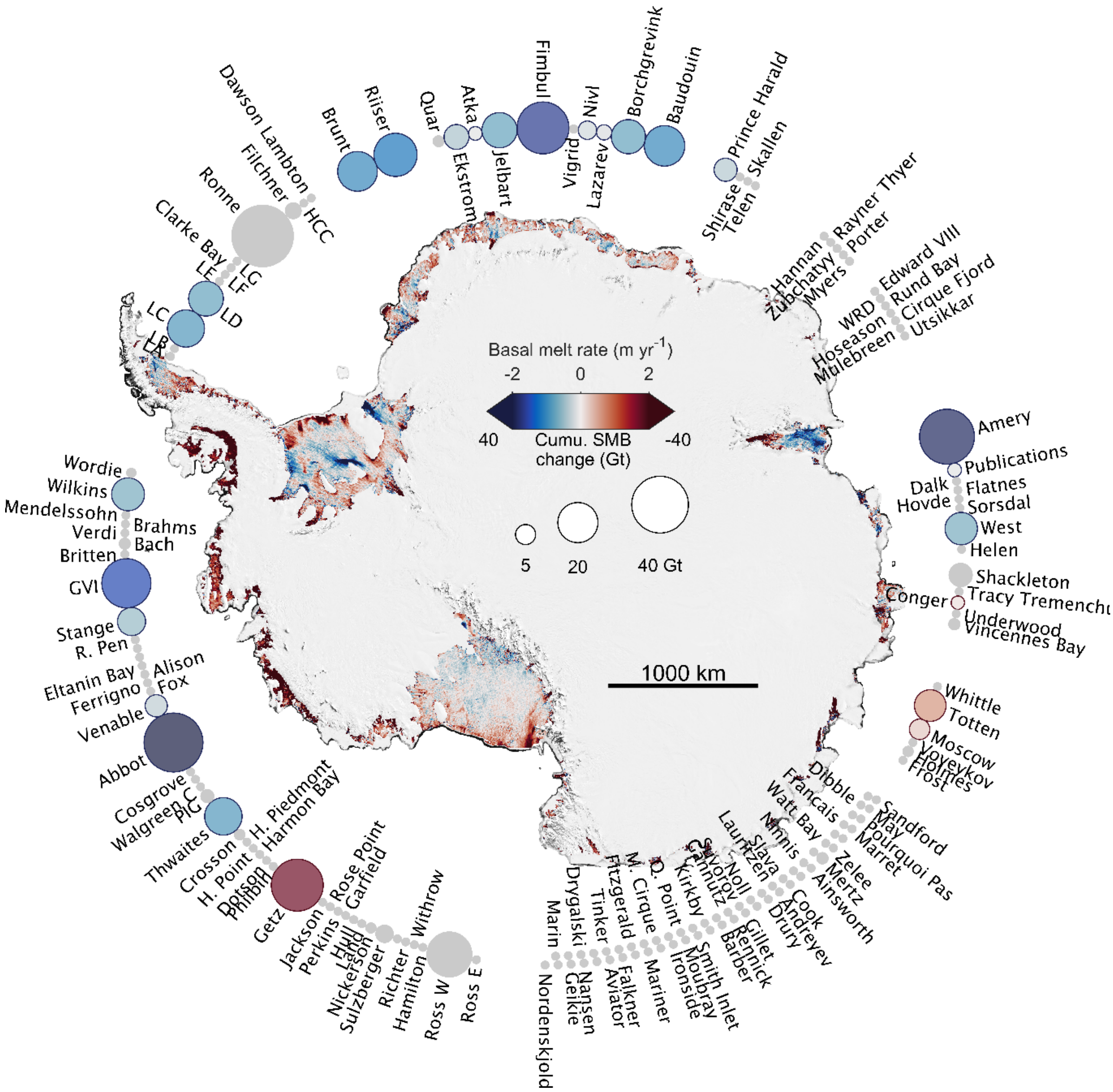

Supplementary Figure 4. Cumulative ice shelf surface mass balance flux. Red circles indicate ice shelves with cumulative surface mass balance excesses from 1997 to 2021, blue circles indicate ice shelves with cumulative surface mass balance deficits and grey circles indicate ice shelves where cumulative surface mass balance anomalies were not significantly different from zero.

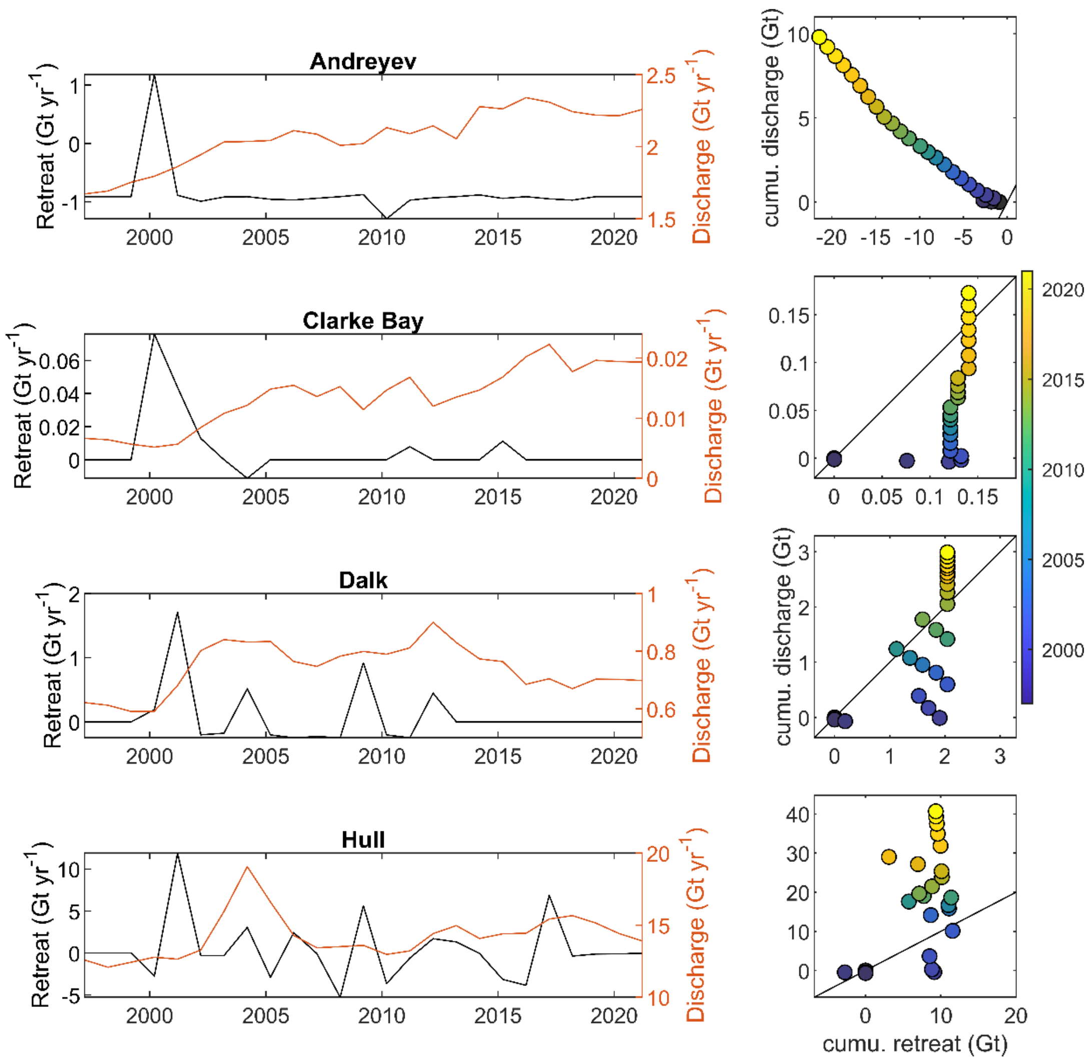

Supplementary Figure 5. The transient discharge response to multiple calving events at selected ice shelves. Each ice shelf experienced one or more major retreat events that was followed by a sustained increase in grounding line discharge.

Supplementary text 1: ice shelf mass budget partitioning

The aim here is to quantify the contributions of basal melting *B*, calving *C*, grounding line discharge *D* and surface mass balance *SMB* to ice shelf mass change. To do this, we calculate time-integrated anomalies of each budget component with respect to some reference state (following ref. (*101*)):

$$\delta M = \int dt\,(D + SMB - C - B) = t(D_0 + SMB_0 - C_0 - B_0) + \int dt\,(\delta \mathrm{D} + \delta \mathrm{SMB} - \delta \mathrm{C} - \delta \mathrm{B})$$

where $\delta M = M - M_0$, with M_0 the unknown reference ice shelf mass and D_0, SMB_0, C_0 and B_0 the reference (or steady-state) discharge, SMB, calving and basal melting respectively (all in Gt yr^{-1}), which together describe the fluxes required to maintain the mass of an ice shelf in steady-state. By definition, $D_0 + SMB_0 = C_0 + B_0$, so the first term on right hand side vanishes; however, D_0, SMB_0, C_0, B_0 are required to calculate δD, δSMB, δC and δB – the anomalies of each budget component, which are time-integrated to estimate the total contribution of each budget component to the overall mass change of each shelf.

Supplementary text 1.1: definition of the reference fluxes

The most intuitive way is to define the reference flux for each component is as follows:

- D_0: the climatological SMB over the grounded ice sheet basin that feeds the ice shelf. A 30-year average (1979-2008 in this case) is generally considered a meaningful climatology for atmospheric variables. This definition is typically used for grounded ice sheet mass budget partitioning, because sustained discharge deviations from this reference discharge imply continuous basin mass gain or loss. Therefore, using this definition assumes that there is some hypothetical steady-state ice shelf associated with the steady-state parent basin.
- SMB_0: the climatological (1979-2008) SMB integrated over the ice shelf itself.
- C_0: the calving flux required to maintain a stationary calving front position, which are taken from ref. (*7*).
- B_0: the basal melt flux required to maintain the ice shelf thickness

Ideally C_0 and B_0 should be defined using thickness and velocity data from the time period in which the ice shelves were in steady-state, so that they are consistent with the definitions of D_0 and SMB_0. In addition, the area of integration for calculating SMB_0 should be the area of the hypothetical steady-state ice shelf.

Supplementary text 1.1.1: reference grounding line discharge

To define D_0, a grounded ice sheet basin for each ice shelf is required. For most ice shelves, it is sufficient to use the NSIDC0709 basins (*1*). However, some of these basins contain several small ice shelves, so we define new basins (Supplementary Figure 6) for these ice shelves using a flow direction mosaic from the error-weighted mean of the ITS-LIVE (*54*) and MEaSUREs (*103*) multi-year velocity mosaics, which contain velocity estimates from 1985 to 2016.

Within each grounded ice sheet basin, we spatially integrate the 1979-2008 mean SMB from RACMO2.3p2 (*62*), MAR (*63, 64*) and HIRHAM5 (*65*), accounting for distortion induced by the polar

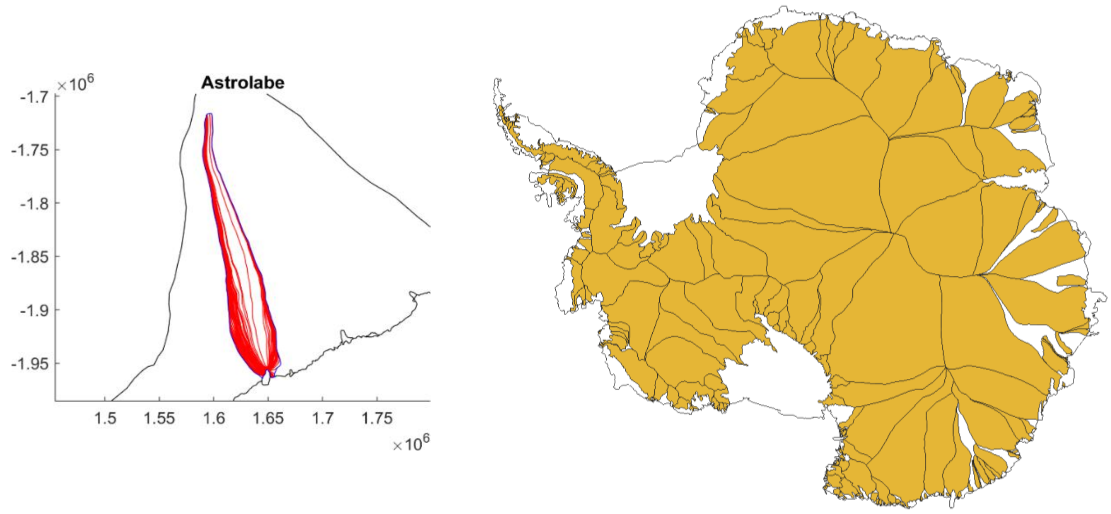

Supplementary Figure 6. Grounded ice sheet basins associated with each ice shelf.

stereographic grid. The uncertainty in D_0 arising from the choice of reference period is quantified using the standard deviation of D_0 from ten rolling 20-year reference periods during the 1979-2008 period (i.e. 1979-1999, 1980-2000 etc).

Supplementary text 1.1.2: reference SMB

To define SMB_0, we integrate SMB estimates from RACMO2.3p2, HIRHAM5 and MAR over a union of our quasi-annual 1997-2021 ice masks (Supplementary Figure 7), accounting for polar stereographic distortion. This union mask is therefore the largest observed ice shelf area during the study period and represents the hypothetical steady-state ice shelf area. We note that some of the small ice shelves are not covered by one or more regional climate models; for those ice shelves, we use the nearest available surface mass balance estimate from each model. The uncertainty in SMB_0 is estimated in the same manner as that for D_0.

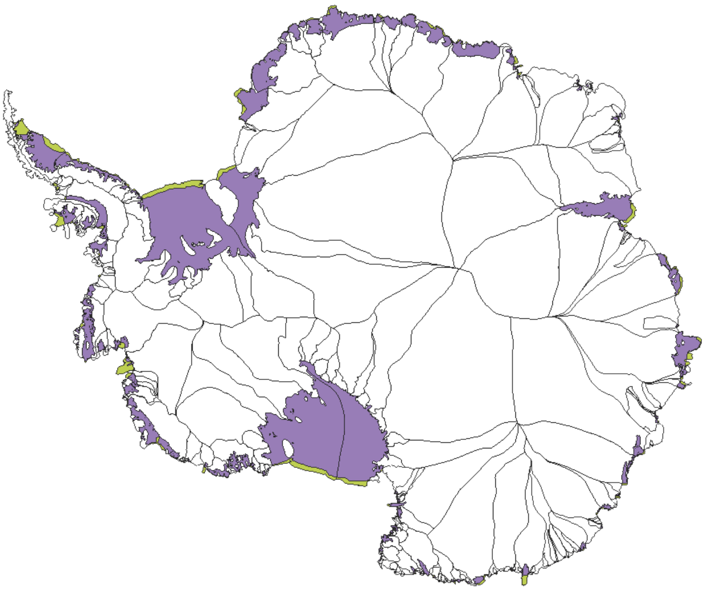

Supplementary Figure 7. Ice shelf masks. The ice shelf union mask (green) with the intersection mask (purple) overlaid for comparison and the grounded basins for each ice shelf in grey.

Supplementary text 1.1.3: reference basal melt flux

The ice shelf mass balance equation is

$$\frac{\partial H}{\partial t} + \nabla.(\boldsymbol{u}H) = a - m$$

So, in principle, we can derive the ice melt rate m at a given time if we know the accumulation a, thinning $\partial H/\partial t$, and flux divergence $\nabla.(\boldsymbol{u}H)$

$$m = a - \frac{\partial H}{\partial t} - \nabla.(\boldsymbol{u}H)$$

For an ice shelf in steady-state in terms of thickness (i.e. one that is not thinning or thickening), then $\partial H/\partial t$=0 and this reduces to:

$$\nabla.(\boldsymbol{u}H) = a - m$$

Thus, we can define a hypothetical 'balance melt rate', m_b, which is the melt rate required to maintain ice shelf thickness (for a given accumulation and ice flux divergence), as:

$$m_b = a - \nabla.(\boldsymbol{u}H)$$

To do this, we need an estimate of flux divergence (velocity u and thickness H) and accumulation that is representative of the time in which the ice shelf was in steady-state. We therefore create a gapless estimate of thickness and velocity by extruding a velocity and thickness mosaic along flowlines (as in ref (*7*)). We begin with a velocity mosaic derived from Amundsen Sea velocities from 1996 and from all MEaSUREs annual mosaics from 2000-2016 (*84*), with small gaps filled using the MEaSUREs multi-year mosaic (*103*), all at 1x1 km posting. We use thickness mosaics over the ice shelf from ref.

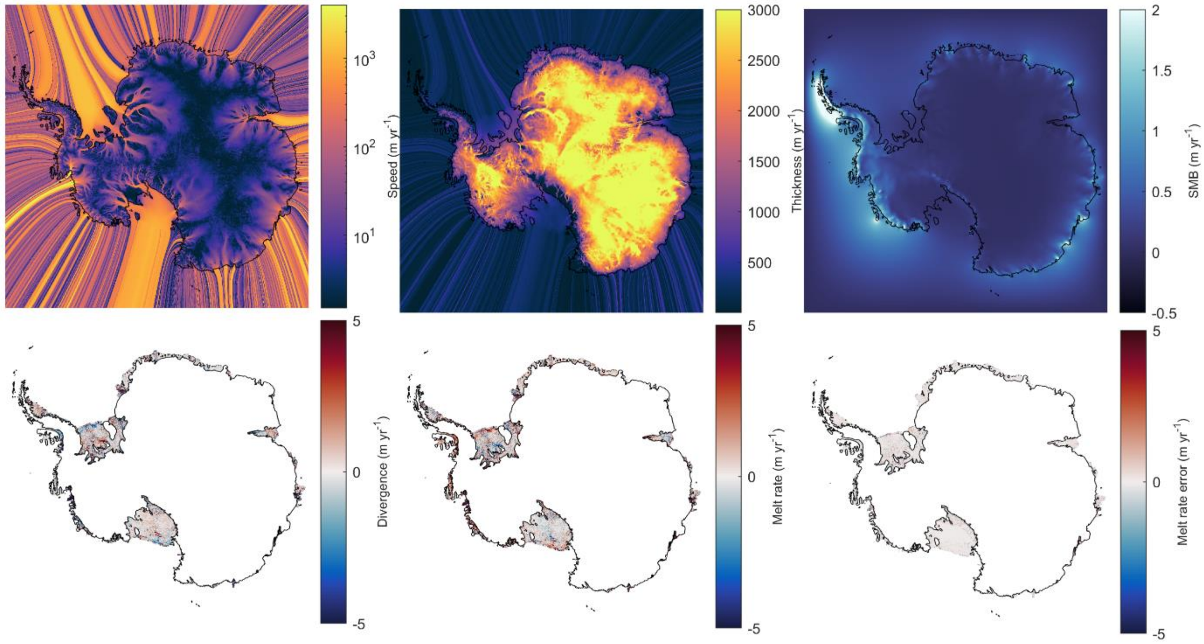

Supplementary Figure 8. Reference ice velocity, ice thickness, accumulation, divergence, balance basal melt rate and balance melt rate error estimates.

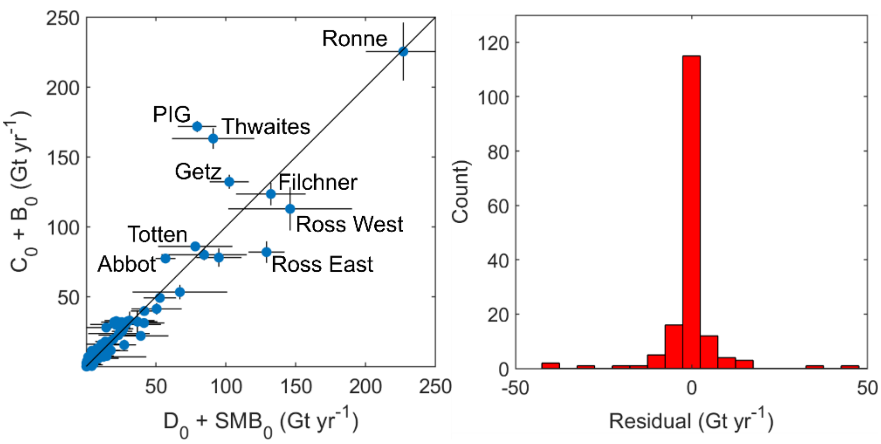

Supplementary Figure 9. Overview of reference fluxes for each ice shelf. Left panel shows the sum of reference mass inputs (D_0 and SMB_0) and reference mass outputs (B_0 and C_0) for each ice shelf, where the black line represents the 1:1 line and a hypothetical balanced ice shelf. The middle panel shows the distribution of apparent mass imbalances (or residuals from zero) implied by the references fluxes.

(46) and over the grounded ice sheet ref. *(94).* Accumulation is obtained from the reference SMB grid defined above and converted to metres of ice equivalent. These inputs, and the resulting balance melt rate grid, are shown in Supplementary Figure 8.

As with SMB_0, we integrate the balance melt rate over the union mask for each ice shelf (Supplementary Figure 8), accounting for polar stereographic distortion to obtain a reference melt flux in Gt/yr. Reference balance melt flux errors are estimated by recalculating the flux divergence with the easting and northing velocity errors included and the thickness error included (assumed to be uniformly 30 m). Uncertainties in the area of integration are not accounted for. We use a static reference melt flux, which means we do not account for the effect of changing flux divergence on the balance melt rate or changes in ice shelf area through time.

Supplementary text 1.1.4: combining the reference fluxes

If the reference fluxes are calculated appropriately, then by definition $D_0+SMB_0=C_0+B_0$. In practice, uncertainties in the input data for each component and inconsistencies in their definition (or the data used to represent them) mean that some residuals remain. Using the definitions and data described

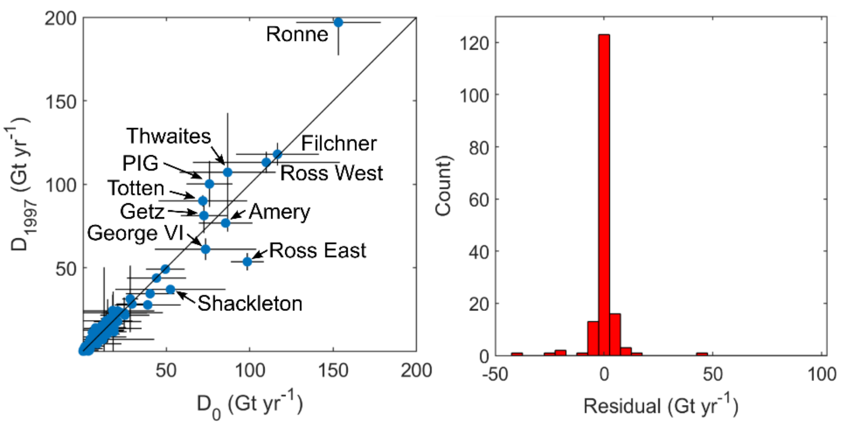

Supplementary Figure 10. The left panel compares the reference discharge, D_0, with the observed discharge in 1997, D_{1997}, for each ice shelf. Deviations from the 1:1 line imply ice shelves that have grounding line discharges greater than or less than the balance discharge estimated from surface mass balance totals over the ice shelf parent basin. The right panel shows the distribution of the differences between those discharge estimates.

above, almost all ice shelves balance within their errors (Supplementary Figure 9). Furthermore, we find that D_0 compares well with our estimated discharge in 1997 for most ice shelves, which is expected because (to the best of our knowledge) most ice shelves have not experienced increases in grounding line discharge (Supplementary Figure 10).

Using these initial estimates for D_0, SMB_0, C_0, B_0, we calculate time-series of annual δD, δSMB, δC and δB. Their time-integrated totals, which are assumed to represent the overall mass change due to each budget component are:

- $\int dt\, \delta\text{D}$ = 1,970±1,390 Gt
- $\int dt\, \delta\text{SMB}$ = 340±90 Gt
- $\int dt\, \delta\text{C}$ = -5,820±700 Gt
- $\int dt\, \delta\text{B}$ = -1,330±1,250 Gt

Combined, these equate to an overall ice shelf mass change of -4,840±2,000 Gt, which differs from our observed ice shelf mass change of -7,500 ± 1,500 Gt.

The differences arise because $D_0+SMB_0=C_0+B_0$ does not equal zero using the estimates above and those residuals accumulate by a factor of 24 over our study period. We therefore modify the reference fluxes to reduce the residuals to zero. First, we set D_0 to the observed 1997 discharge estimate, D_{1997}; overall, D_0 and D_{1997} compare well (Supplementary Figure 10), but the change in definition reduces $\int dt\, \delta D_{1997}$ to 1,770±870 and $\int dt\, \delta\text{M}$ to -5,030±1,680, which is closer to (but still very different from) our observed mass change.

The residuals remaining from $D_{1997}+SMB_0=C_0+B_0$ are shown in Supplementary Figure 11. We assume that all of these residuals arise from B_0, and therefore modify B_0 (forming B_{mod}) so that $\int dt\, \delta\text{M}$ equals our observed mass change. This is a simplistic assumption, but we justify it on the grounds that we know that the velocity and thickness data used to define B_0 were acquired recently and therefore are unlikely to representative of steady-state ice shelves in many cases. A comparison of B_0 and B_{mod} are shown in supplementary Figure 12.

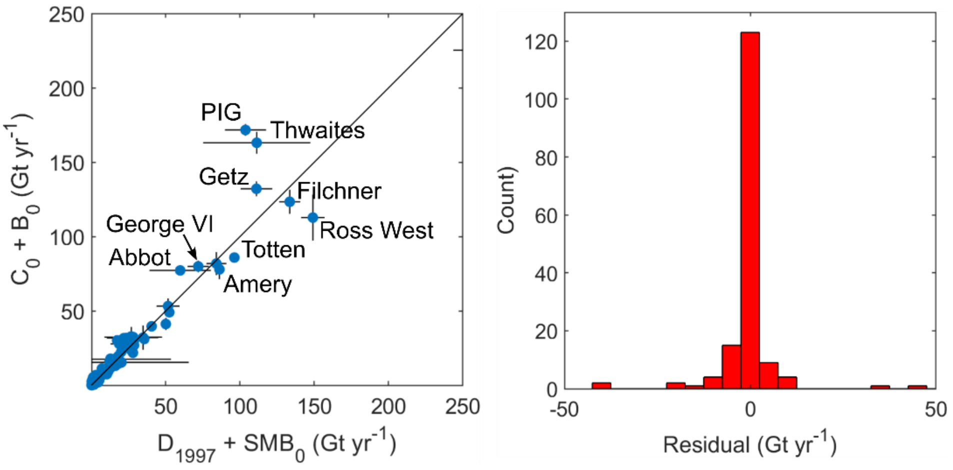

Supplementary Figure 11. As for Supplementary Fig. 9, but using the observed discharge in 1997 as the reference discharge.

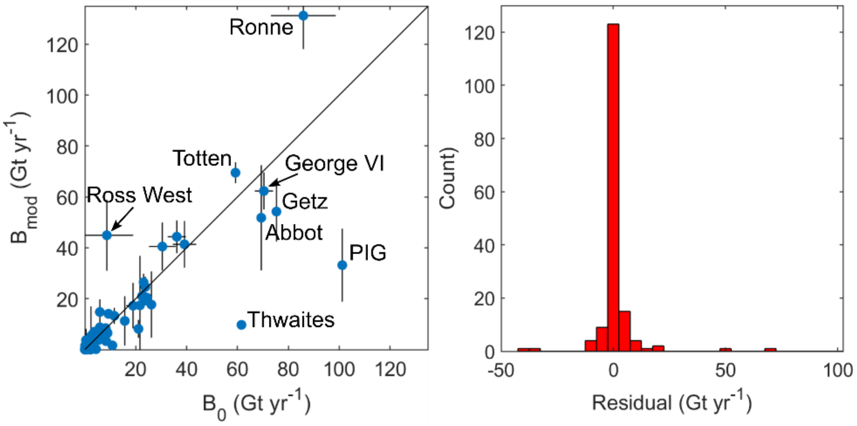

Supplementary Figure 12. Comparison of reference melt flux with the modified melt flux.

Supplementary text 2: comparison with previous studies

Supplementary Table 1 compares our integrated basal melt anomalies with thickness changes from previous studies (refs (*7*), (*45*), (*104*), (*105*), (*106*)). The differences between studies arise for several reasons. (i) Changes in ice shelf thinning rates over time. (ii) Because of sometimes large differences in ice shelf masks between studies or, in the case of our study, the use of time-varying ice shelf masks. (iii) Because we accumulate basal melt anomalies annually whereas other studies examine net thickness changes directly. (iv) Because of differences in elevation measurement density and therefore thickness change spatial resolution, which allow us to sample closer to the grounding line than in previous studies, which increases thinning rates on Pine Island and Thwaites, for example.

Supplementary Table 1. Comparison of our cumulative basal melt anomalies with *Greene2022* (ref *7*), *Paolo2015* (ref *45*), *Pritchard2012* (ref *104*), *Shepherd2004* (ref *105*) and *Shepherd2010* (ref *106*). All values are m/year and rounded to facilitate the comparison. Values not significantly different from zero were set to 0.0. The values for this study and *Greene2022* are converted to m/year using our intersection mask (Supplementary Fig. 8). Other values are taken from *Paolo2015* (ref *45*).

Ice shelf	This study 25 years (1997-2021)	*Greene2022* 25 years (1997-2021)	*Paolo2015* 18 years (1994-2012)	*Pritchard2012* 5 years (2003-2008)	*Shepherd2004* 9 years (1992-2001)	*Shepherd2010* 14 years (1994-2008)
Sulzberger	-0.2	0.1	0.0	0.3		
Nickerson	0.1	0.3	0.0	0.0		
Getz	-2.6	-0.9	-1.6	-1.7	-1.6	-1.8
Dotson	-6.1	-3.2	-2.6	-5.2	-3.3	
Crosson	-9.1	-4.8	-3.1	-3.3	-4.5	
Thwaites	-13.1	-6.6	-2.8	-5.6	-5.5	-8.3
Pine Island	-15.2	-3.6	-2.3	-4.9	-3.9	-6.0
Cosgrove	-0.3	-0.3	-0.2	-0.6	-0.7	
Abbot	-0.3	0.0	-0.2	0.4	-0.6	
Venable	-1.7	-2.8	-3.6	-2.5		-16.0
Stange	-1.0	-1.0	-0.8	-0.6		
Bach	-0.8	-0.3	-0.9	-0.7		8.8
Wilkins	-1.0	0.2	-0.6	-0.6		
George VI	-0.7	-0.7	-1.1	-0.9		-0.8
Larsen-B	-1.7	-0.8	-0.4	-2.3		
Larsen-C	-0.6	-0.5	-0.5	-0.9		-0.8
Larsen-D	-0.2	-0.1	-0.2	0.4		
Brunt	0.2	-0.1	0.3	0.3		0.6
Riiser	0.0	-0.1	0.1	0.3		
Fimbul	0.1	0.2	0.3	0.0		-0.5
Lazarev	0.0	0.1	0.0	-0.6		
Amery	0.4	-0.1	0.2	-0.6		0.9
West	0.4	-0.2	0.0	-1.1		
Shackleton	-0.6	-0.9	0.0	-1.1		
Totten	1.2	0.6	0.0	-3.8		
Moscow	0.0	-0.5	0.0	-1.0		5.4
Holmes	1.5	-1.1	0.0	-2.8		
Dibble	-0.2	0.5	-1.0	-2.2		
Mertz	-2.3	0.2	0.0	0.3		
Cook	1.0	0.4	0.0	1.1		
Rennick	-0.8	-0.5	-0.5	-1.2		
Mariner	-0.3	-0.2	0.0	0.2		
Drygalski	0.0	-0.2	0.0	-0.3		
Ross	0.1	0.0	-0.2	0.1		0.2
Filchner-Ronne	0.1	0.0	0.0	0.2		0.5

Summary figures 13-174 for individual ice shelves

In each figure, the bottom left panel shows an overview of the ice shelf, with quasi-annual coastlines (scaling from blue to yellow chronologically) overlain on a 150x150 m MODIS mosaic (*102*) and with the 2010-2021 average basal melt rate (Methods) and MEaSUREs velocity vectors overlain (*103*). This panel was produced with the aid of the Antarctic Mapping Tools for MATLAB(*107*). The top right panel shows the grounding line discharge observations (black dots) and errors (grey lines). The middle right panel shows the mass balance components as coloured bars (orange for SMB, blue for discharge, yellow for calving and purple for basal melt) with their errors as whiskers and the annual mass balance as a black line with errors shaded grey. The bottom right panel shows the cumulative mass changes due to each mass budget component, using the same colour scheme as in the middle right panel.

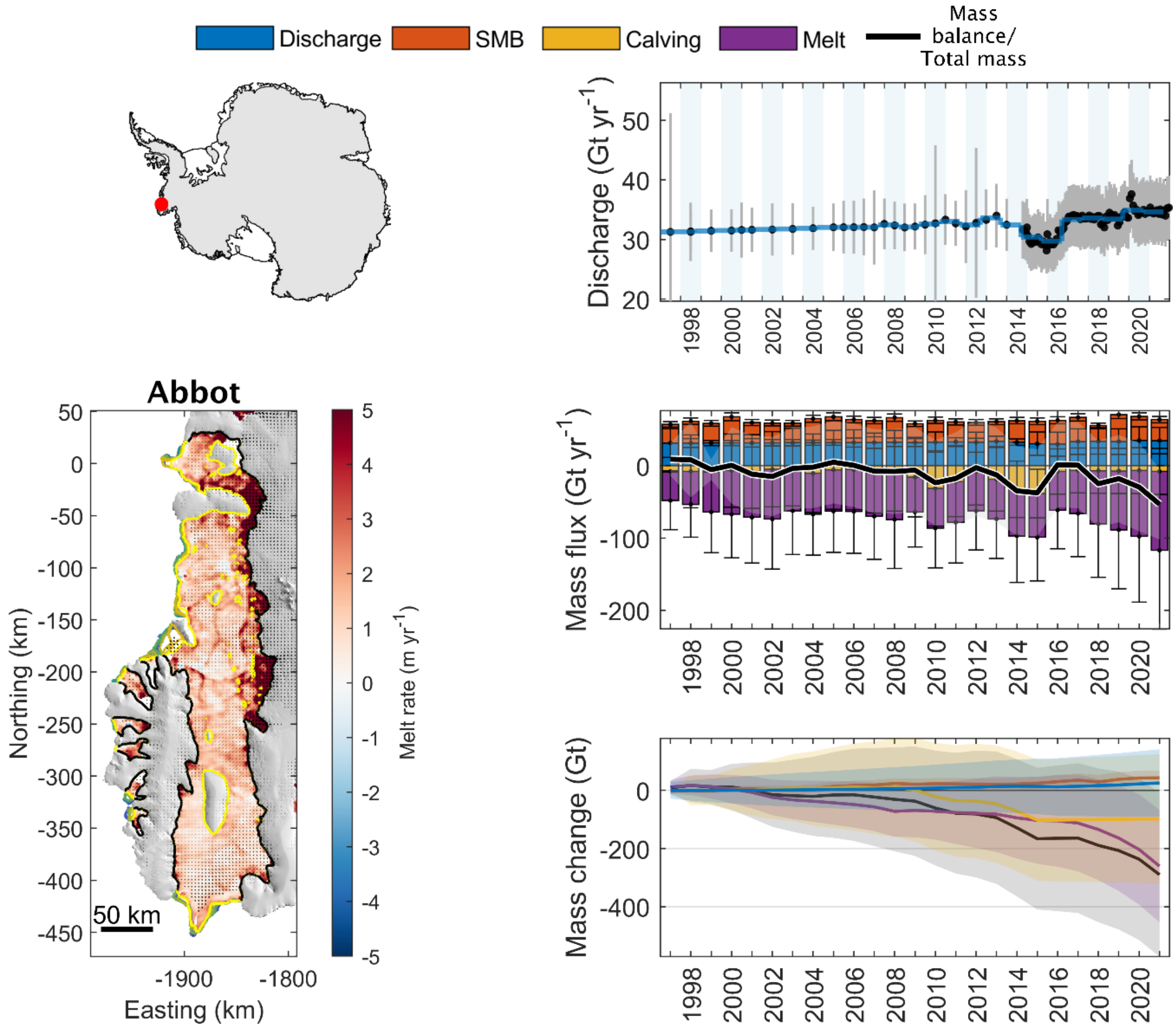

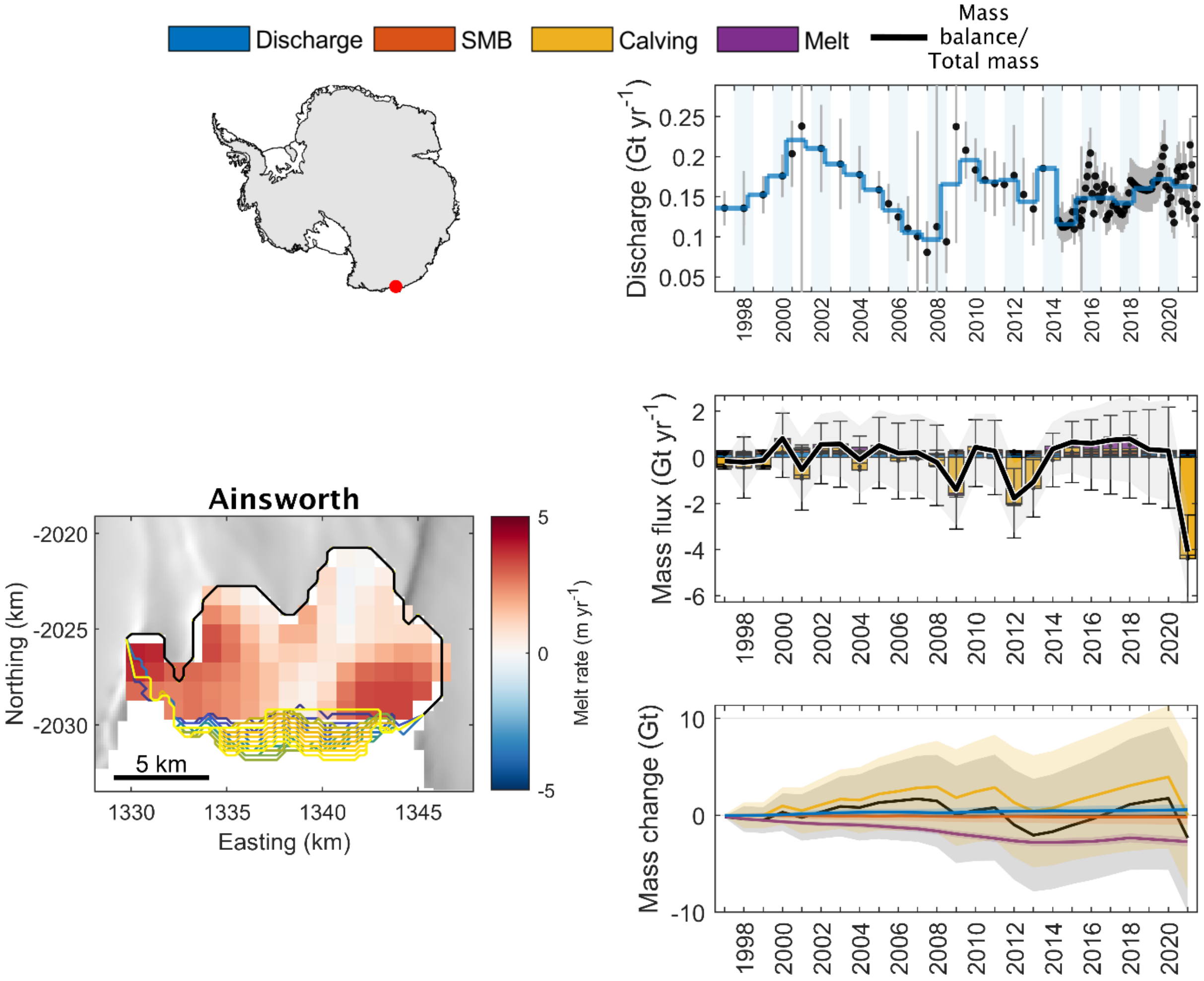
Discharge
SMB
Calving
Melt
Mass balance/ Total mass
Ainsworth
Northing (km)
Easting (km)
5 km
Melt rate (m yr-1)
Discharge (Gt yr-1)
Mass flux (Gt yr-1)
Mass change (Gt)

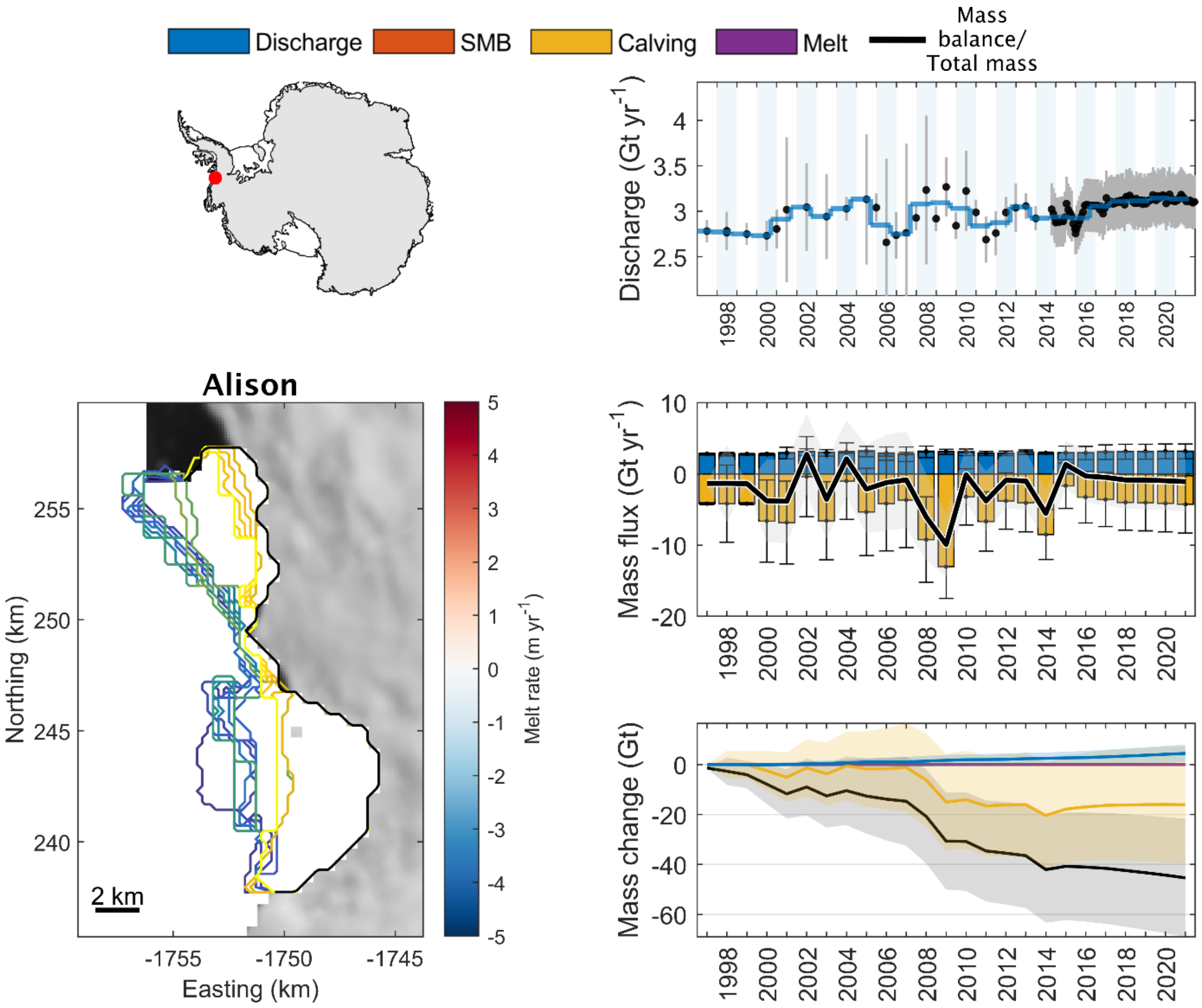
Discharge
SMB
Calving
Melt
Mass balance/ Total mass
Alison
Northing (km)
Easting (km)
2 km
Melt rate (m yr^{-1})
Discharge (Gt yr^{-1})
Mass flux (Gt yr^{-1})
Mass change (Gt)

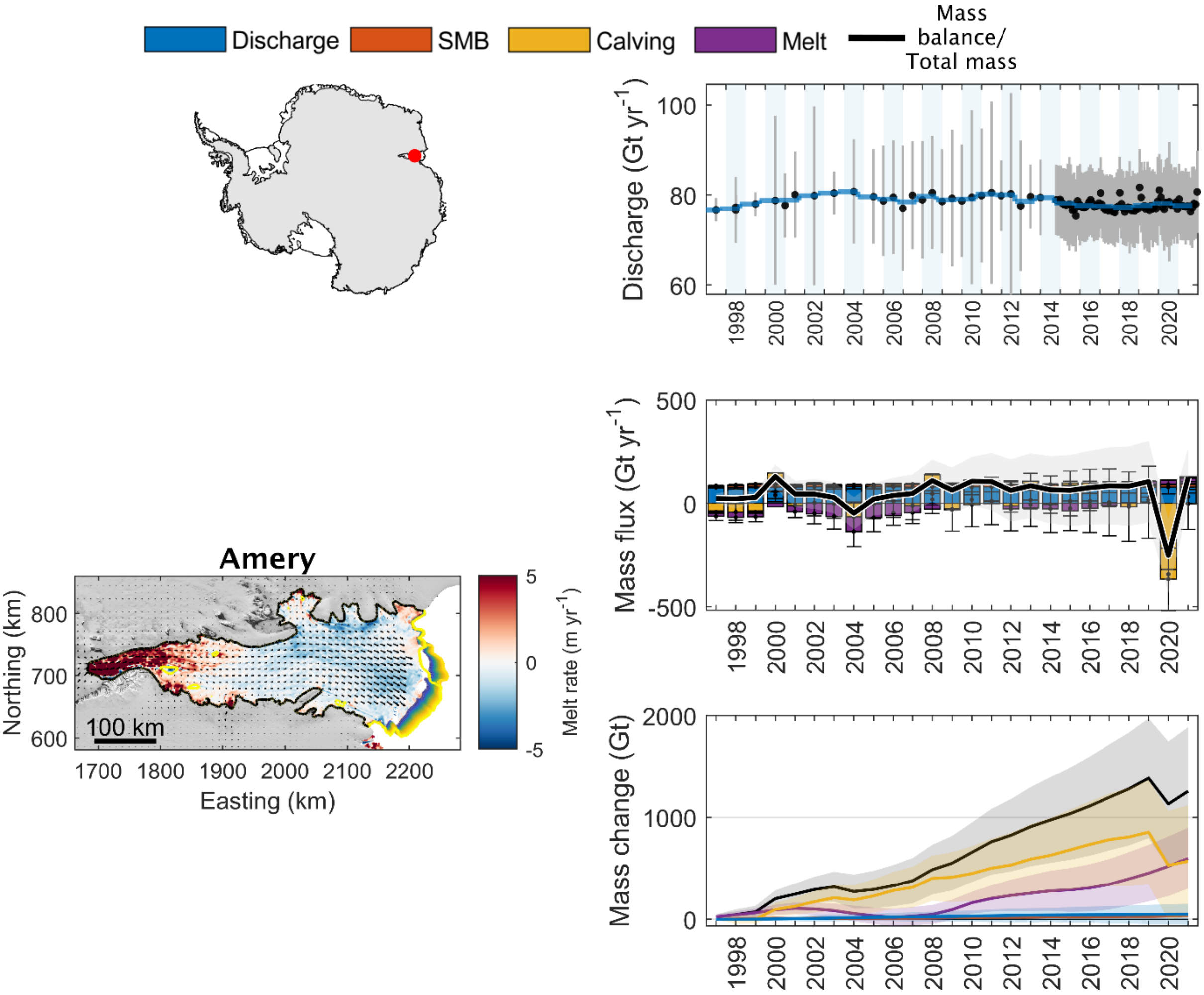

Discharge
SMB
Calving
Melt
Mass balance/ Total mass
Discharge (Gt yr-1)
Mass flux (Gt yr-1)
Mass change (Gt)
Amery
100 km
Northing (km)
Easting (km)
Melt rate (m yr-1)

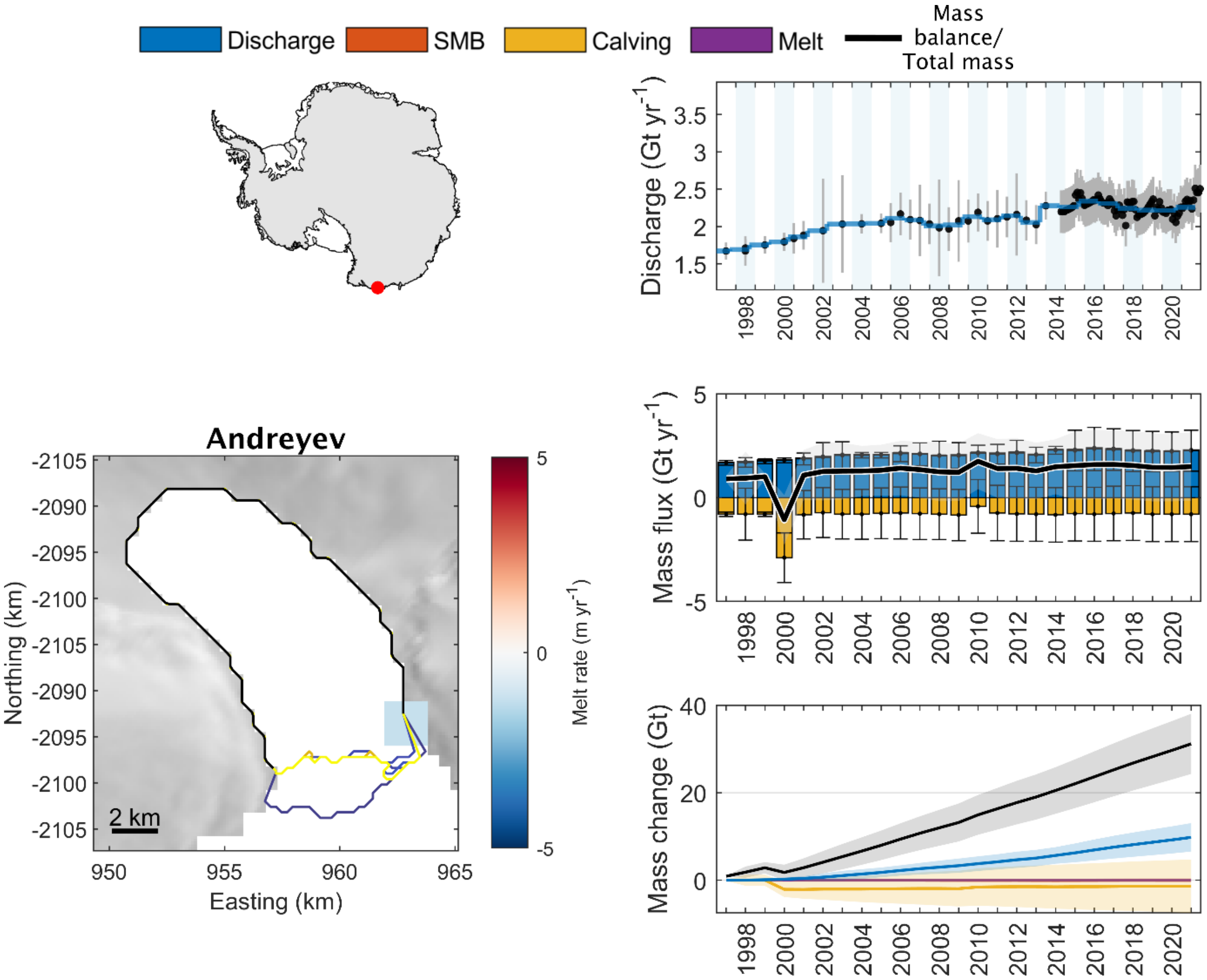
Discharge
SMB
Calving
Melt
Mass balance/ Total mass
Andreyev
Northing (km)
Easting (km)
2 km
Melt rate (m yr^-1)
Discharge (Gt yr^-1)
Mass flux (Gt yr^-1)
Mass change (Gt)

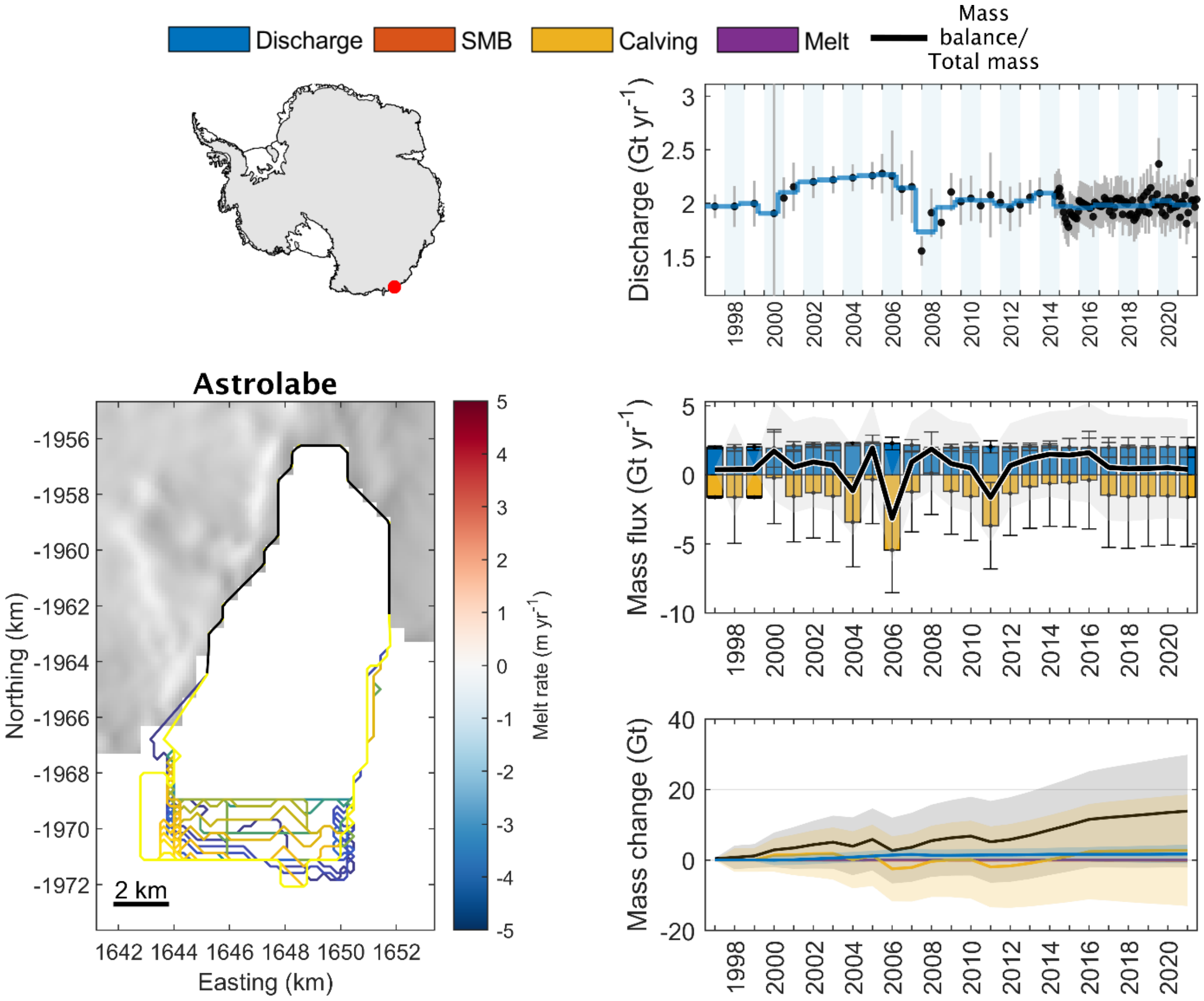

Discharge
SMB
Calving
Melt
Mass balance/ Total mass
Astrolabe
Northing (km)
Easting (km)
2 km
Melt rate (m yr-1)
Discharge (Gt yr-1)
Mass flux (Gt yr-1)
Mass change (Gt)

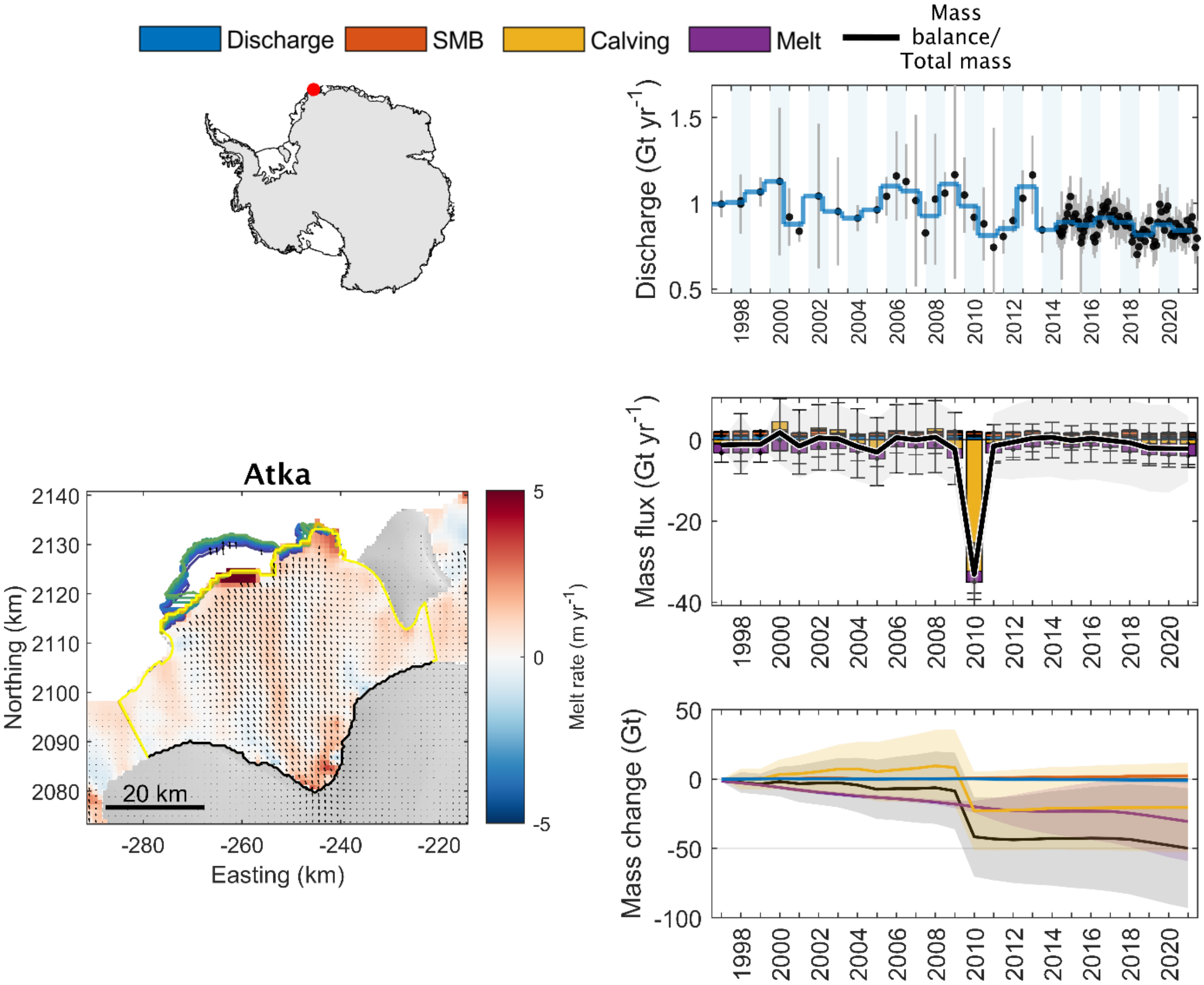

Discharge
SMB
Calving
Melt
Mass balance/ Total mass
Atka
Northing (km)
Easting (km)
20 km
Melt rate (m yr^{-1})
Discharge (Gt yr^{-1})
Mass flux (Gt yr^{-1})
Mass change (Gt)

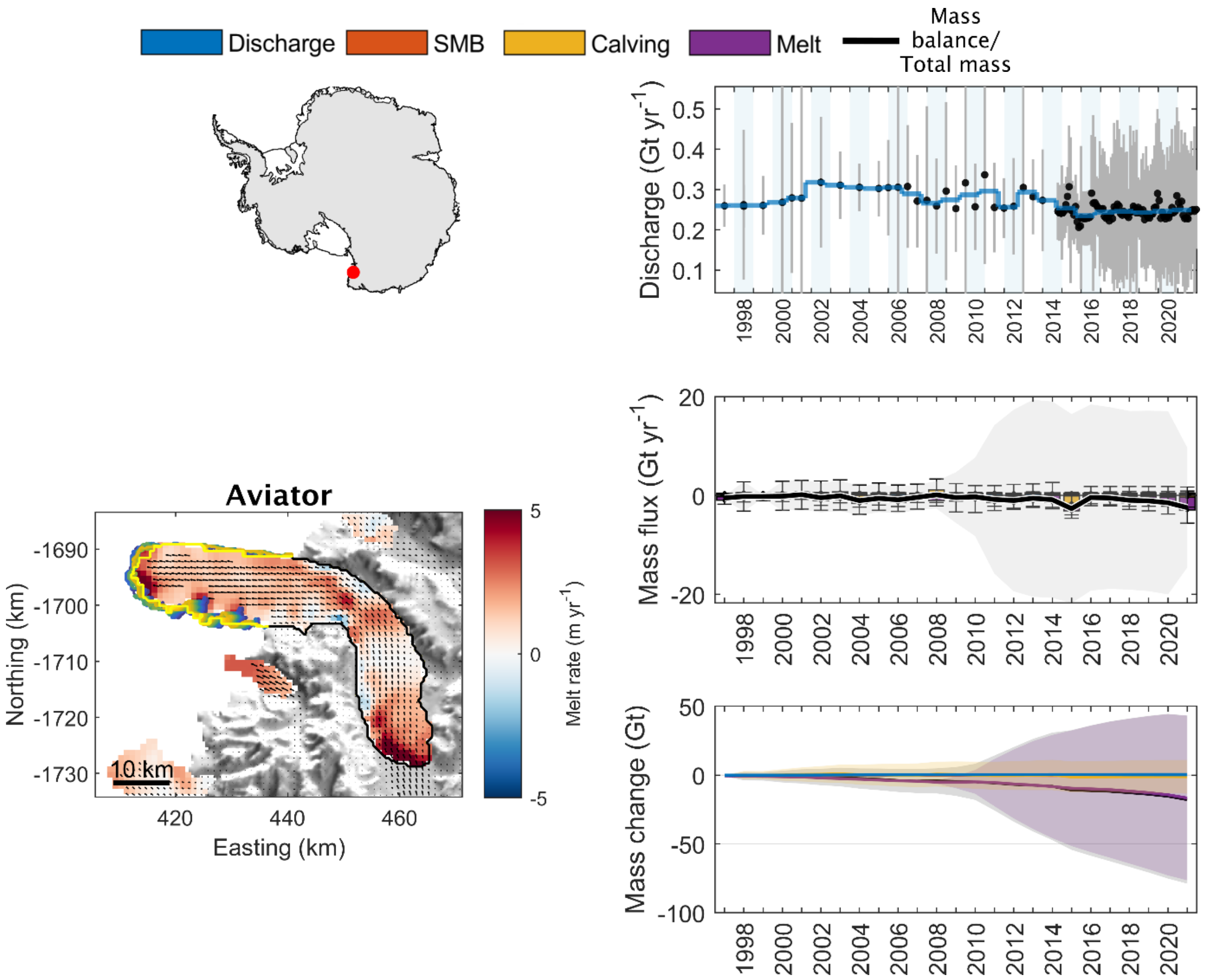

Discharge
SMB
Calving
Melt
Mass balance/ Total mass
Aviator
Northing (km)
Easting (km)
10 km
Melt rate (m yr-1)
Discharge (Gt yr-1)
Mass flux (Gt yr-1)
Mass change (Gt)

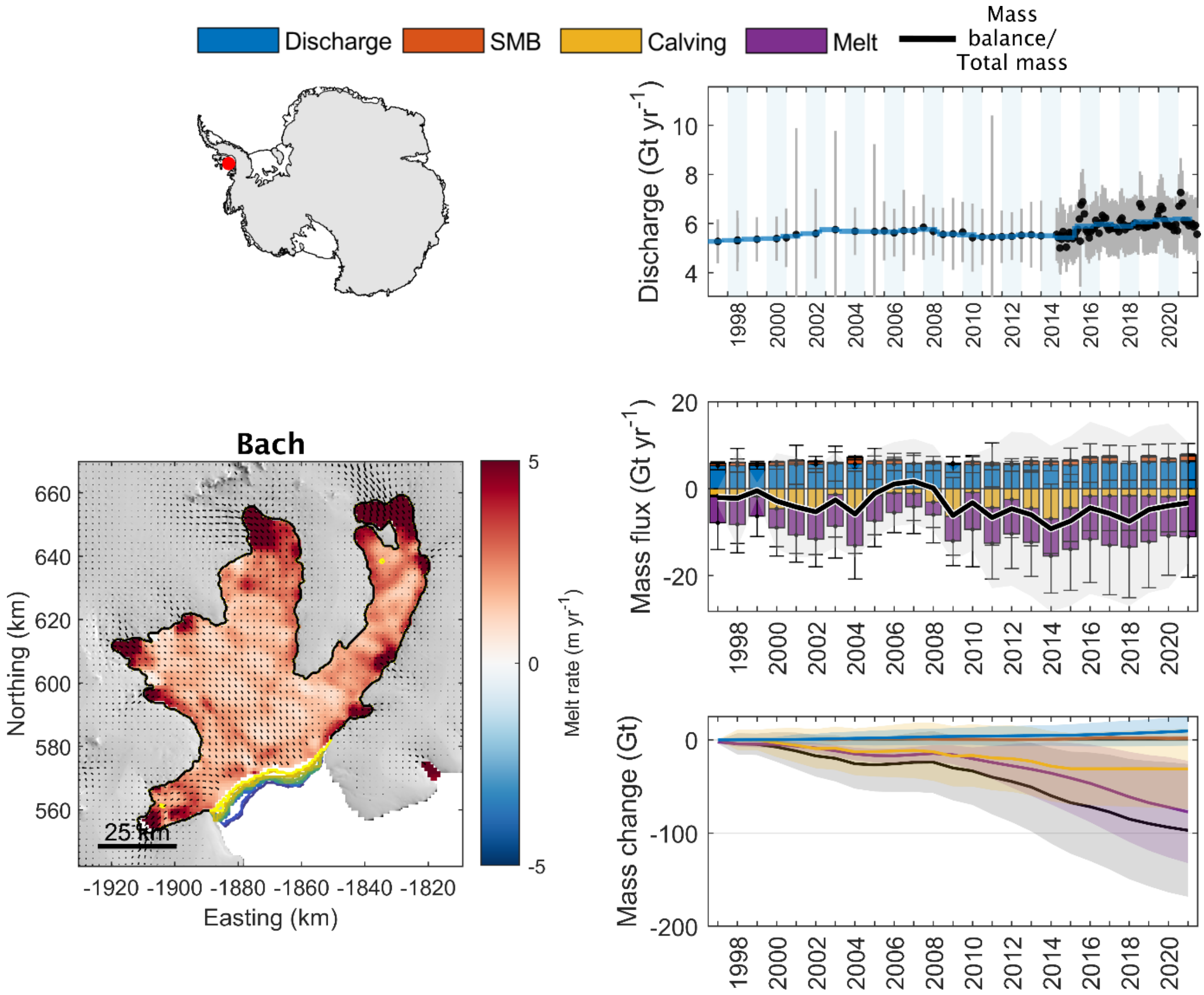
Discharge
SMB
Calving
Melt
Mass balance/ Total mass
Discharge (Gt yr-1)
Bach
Northing (km)
Easting (km)
25 km
Melt rate (m yr-1)
Mass flux (Gt yr-1)
Mass change (Gt)

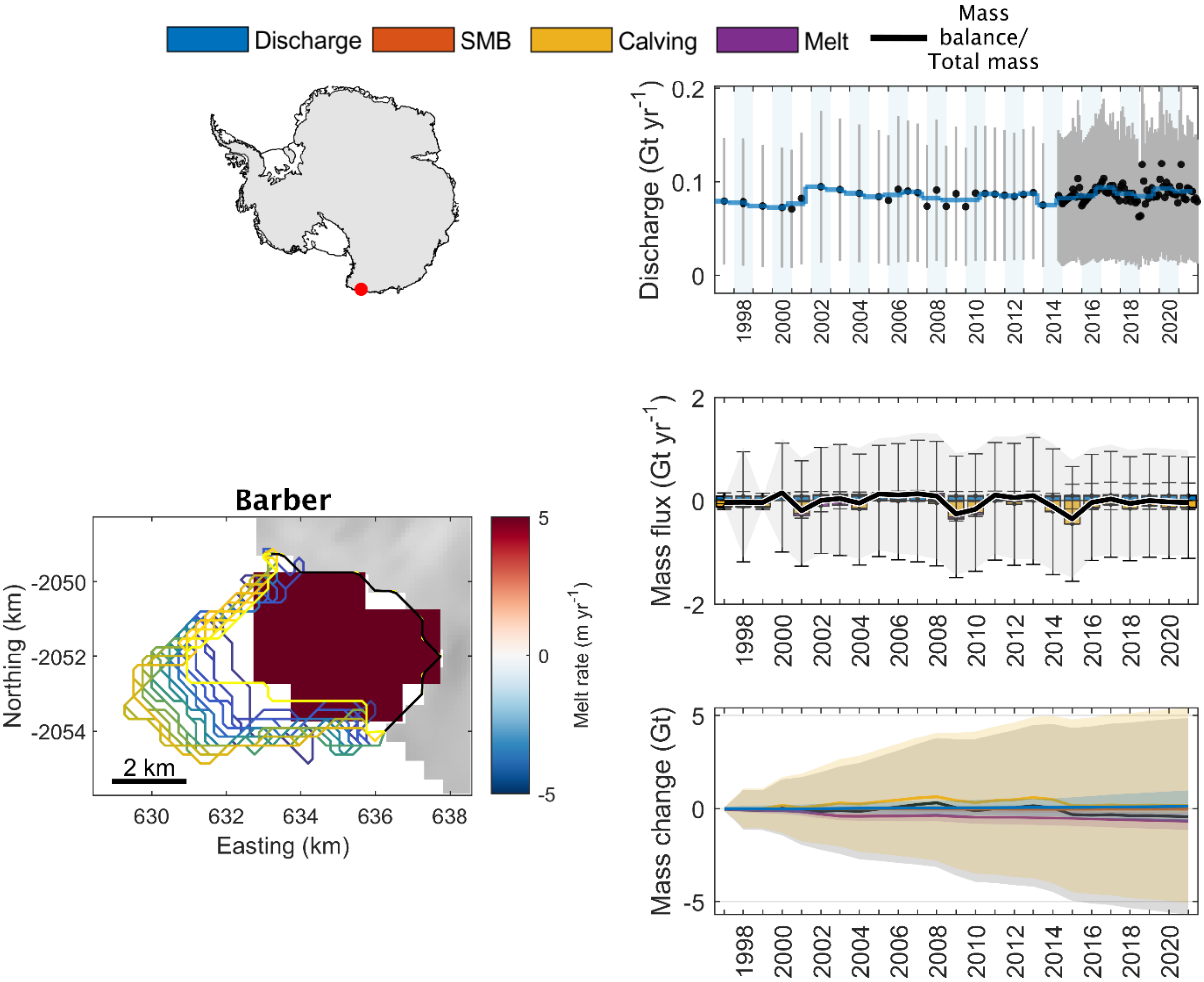
Discharge
SMB
Calving
Melt
Mass balance/ Total mass
Barber
Northing (km)
-2050
-2052
-2054
2 km
630
632
634
636
638
Easting (km)
5
0
-5
Melt rate (m yr⁻¹)
Discharge (Gt yr⁻¹)
0.2
0.1
0
Mass flux (Gt yr⁻¹)
2
0
-2
Mass change (Gt)
5
0
-5
1998
2000
2002
2004
2006
2008
2010
2012
2014
2016
2018
2020

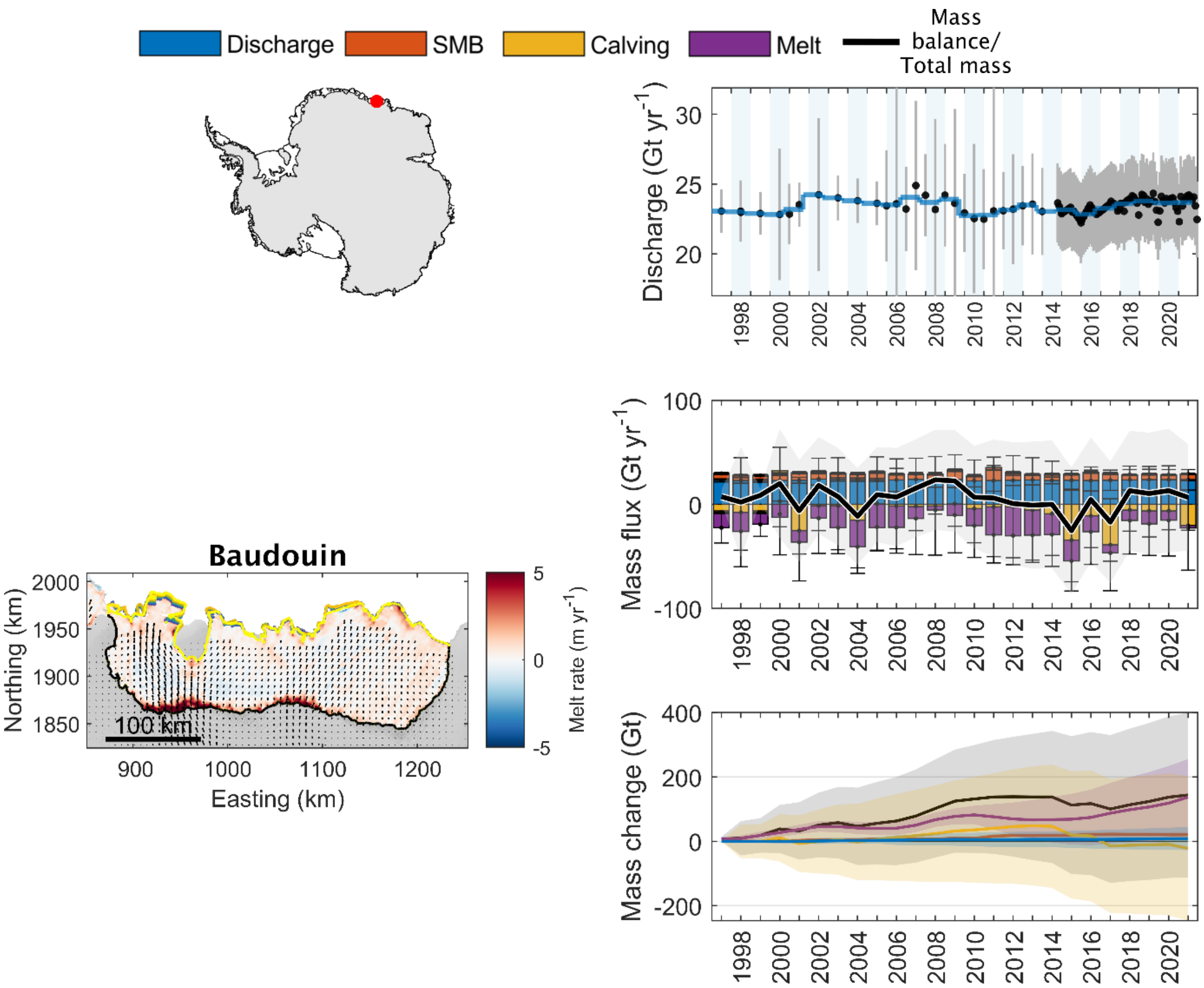

Discharge
SMB
Calving
Melt
Mass balance/ Total mass
Baudouin
Northing (km)
Easting (km)
100 km
Melt rate (m yr-1)
Discharge (Gt yr-1)
Mass flux (Gt yr-1)
Mass change (Gt)

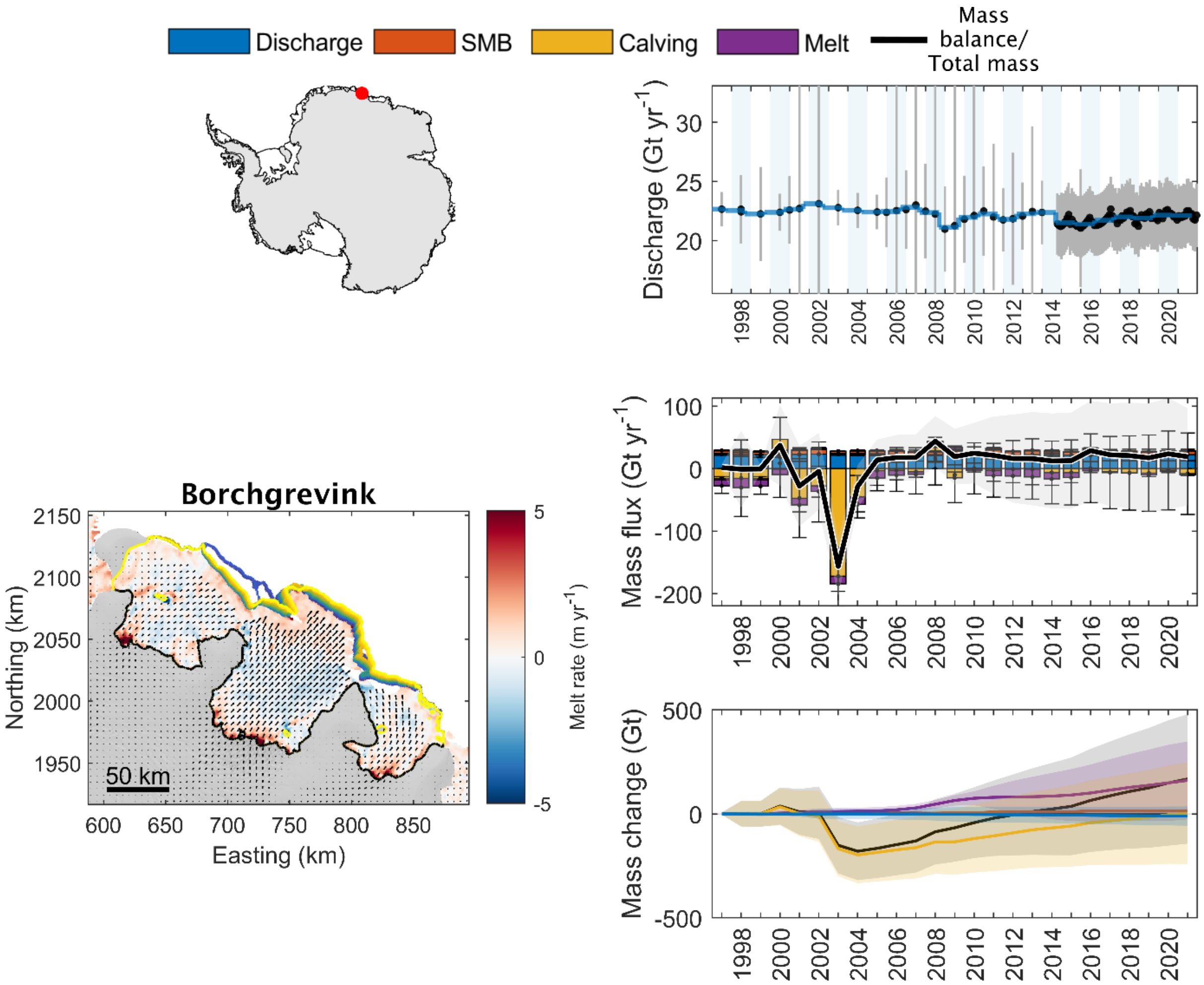

Discharge
SMB
Calving
Melt
Mass balance/ Total mass
Borchgrevink
Northing (km)
Easting (km)
50 km
Melt rate (m yr-1)
Discharge (Gt yr-1)
Mass flux (Gt yr-1)
Mass change (Gt)

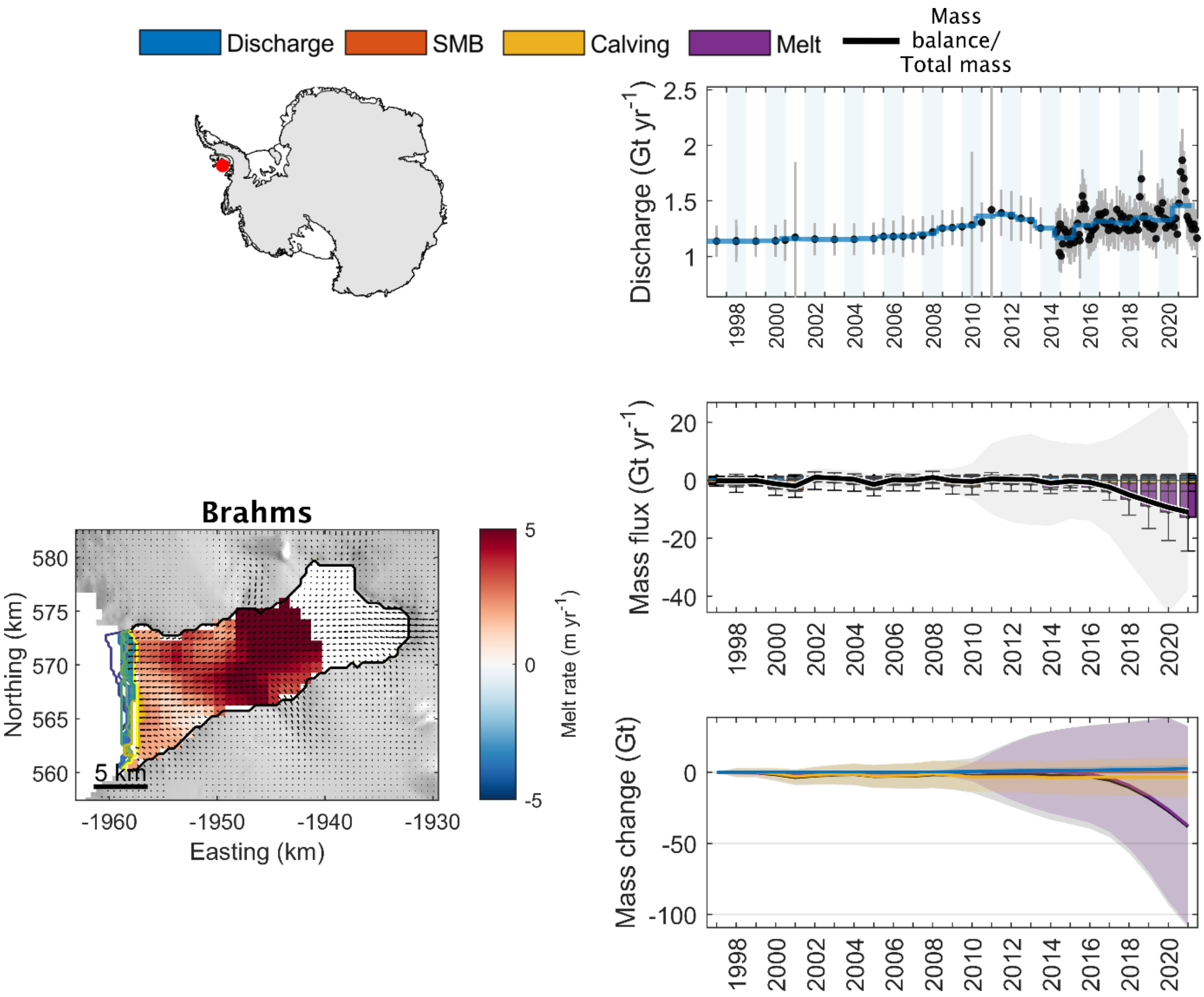

Discharge
SMB
Calving
Melt
Mass balance/ Total mass
Brahms
Northing (km)
Easting (km)
5 km
Melt rate (m yr^{-1})
Discharge (Gt yr^{-1})
Mass flux (Gt yr^{-1})
Mass change (Gt)

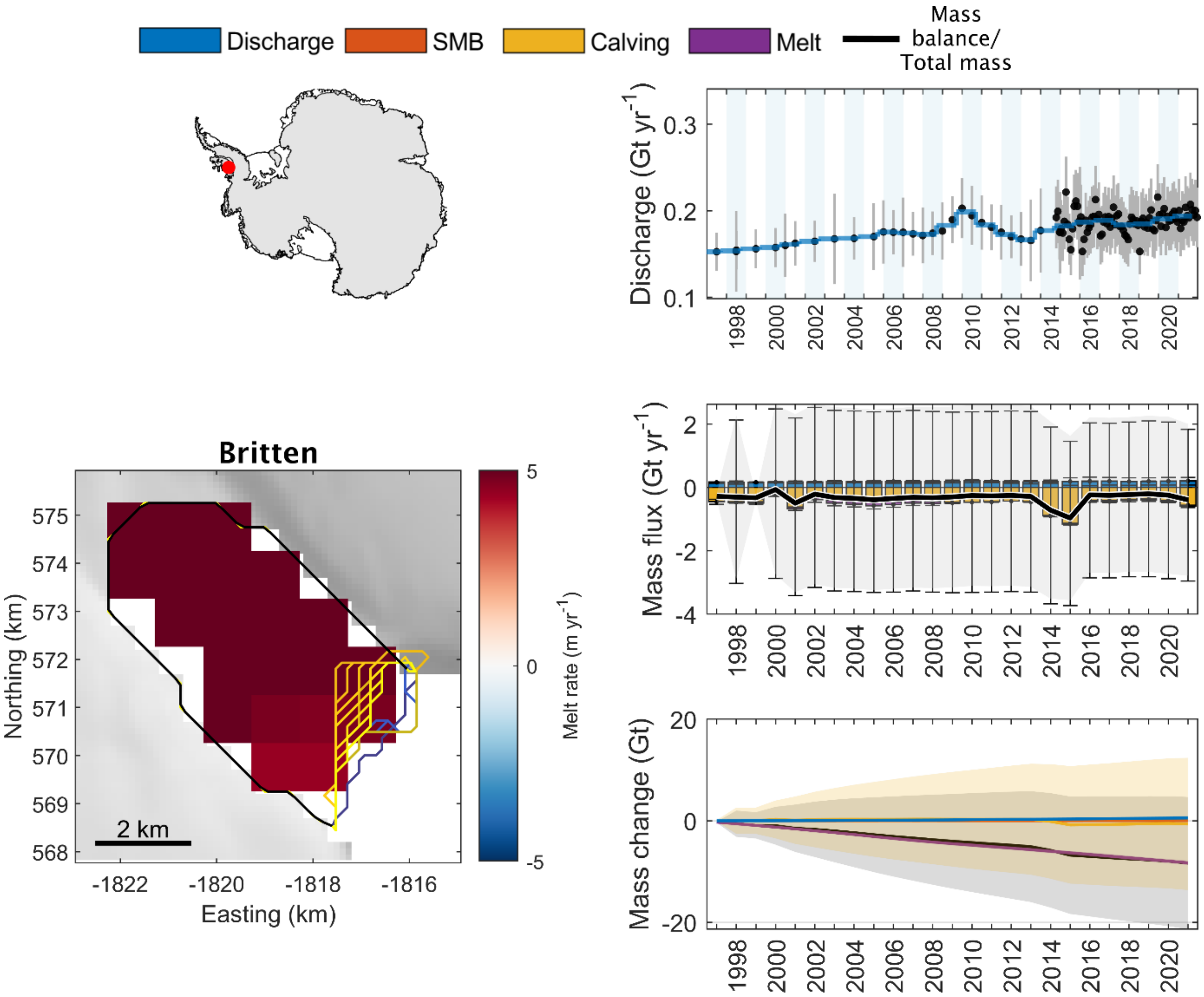
Discharge
SMB
Calving
Melt
Mass balance/ Total mass
Discharge (Gt yr-1)
Britten
Northing (km)
Easting (km)
2 km
Melt rate (m yr-1)
Mass flux (Gt yr-1)
Mass change (Gt)

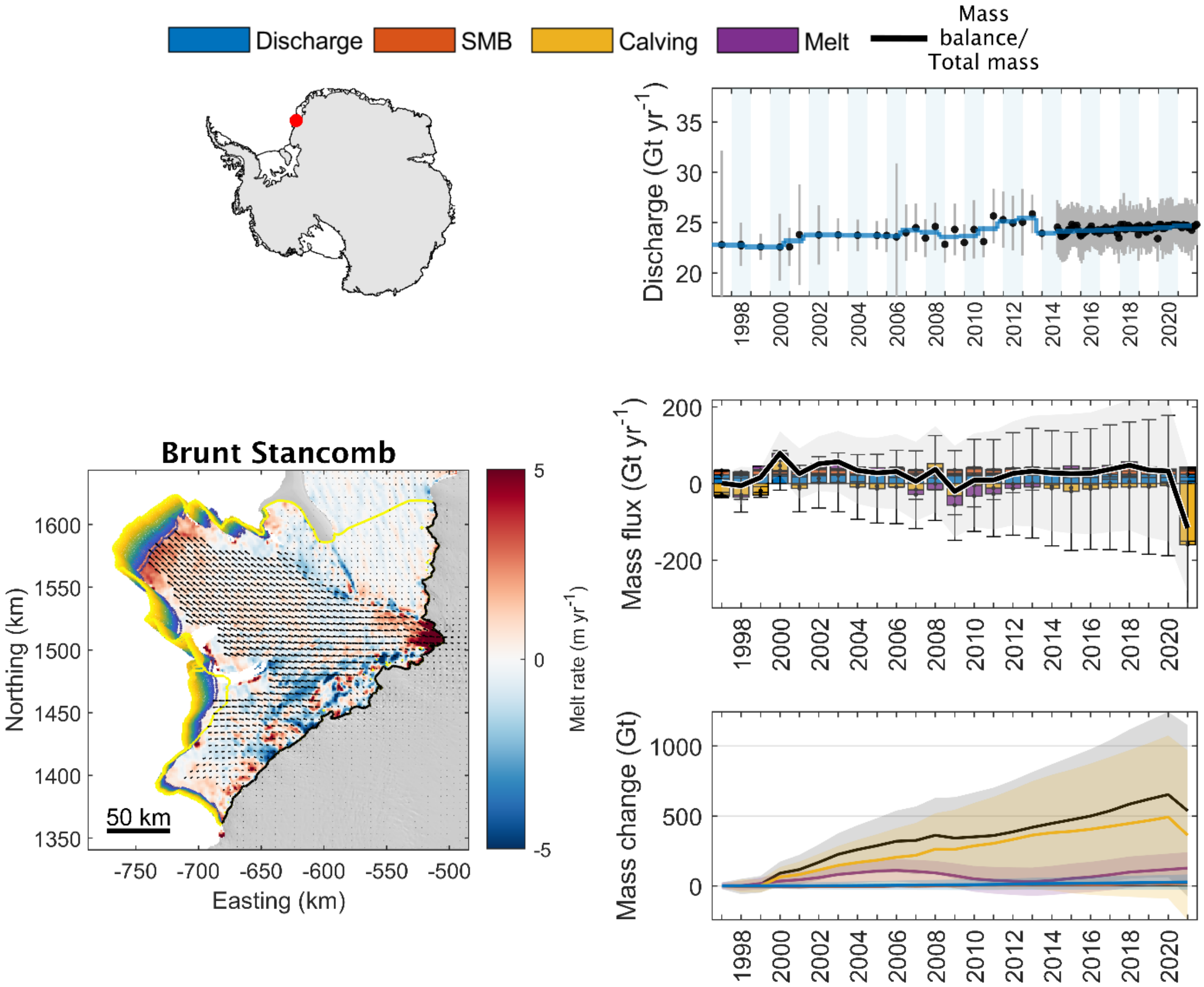
Discharge
SMB
Calving
Melt
Mass balance/ Total mass
Brunt Stancomb
Northing (km)
Easting (km)
50 km
Melt rate (m yr-1)
Discharge (Gt yr-1)
Mass flux (Gt yr-1)
Mass change (Gt)

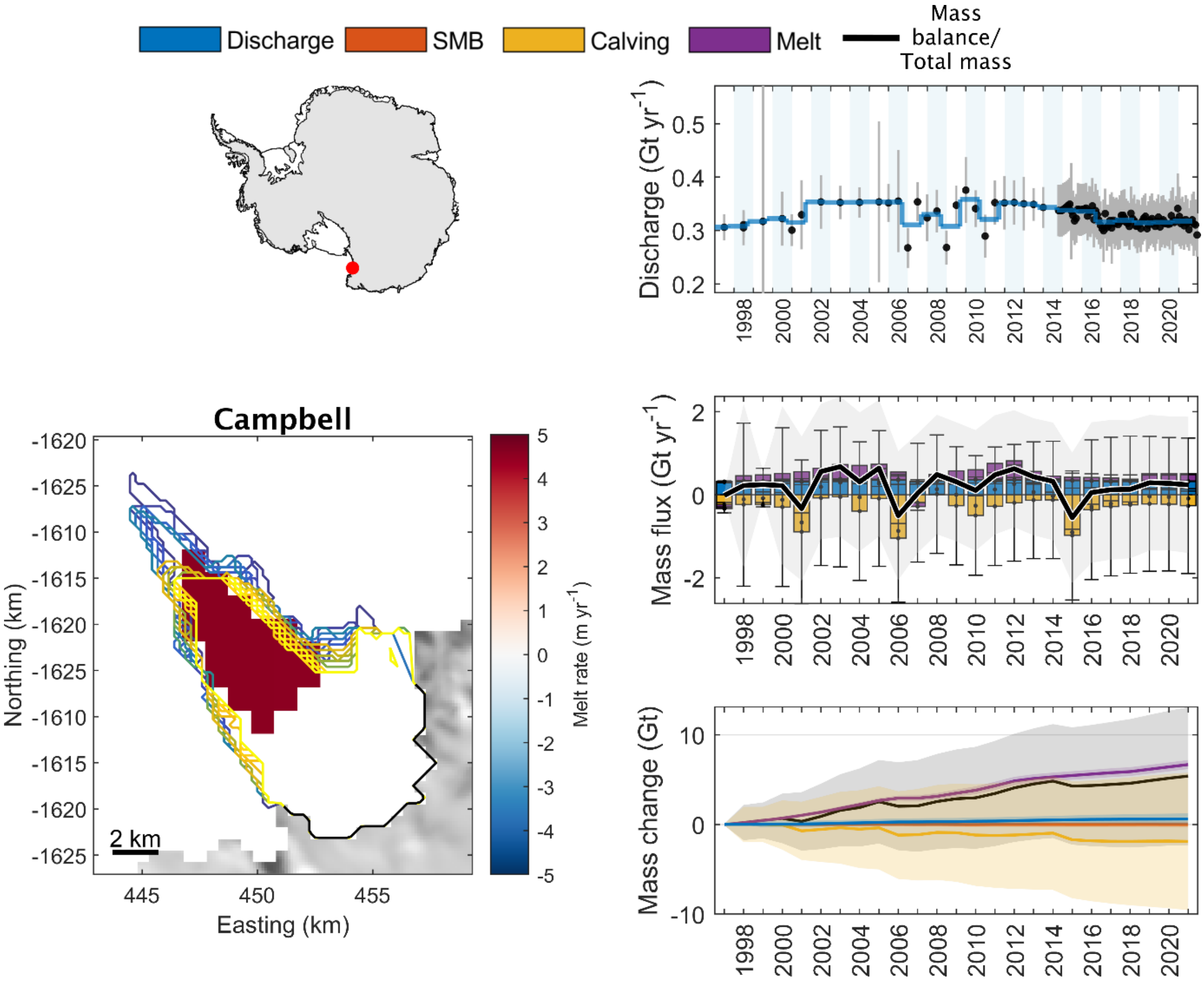

Discharge
SMB
Calving
Melt
Mass balance/ Total mass
Campbell
Northing (km)
Easting (km)
2 km
Melt rate (m yr^{-1})
Discharge (Gt yr^{-1})
Mass flux (Gt yr^{-1})
Mass change (Gt)

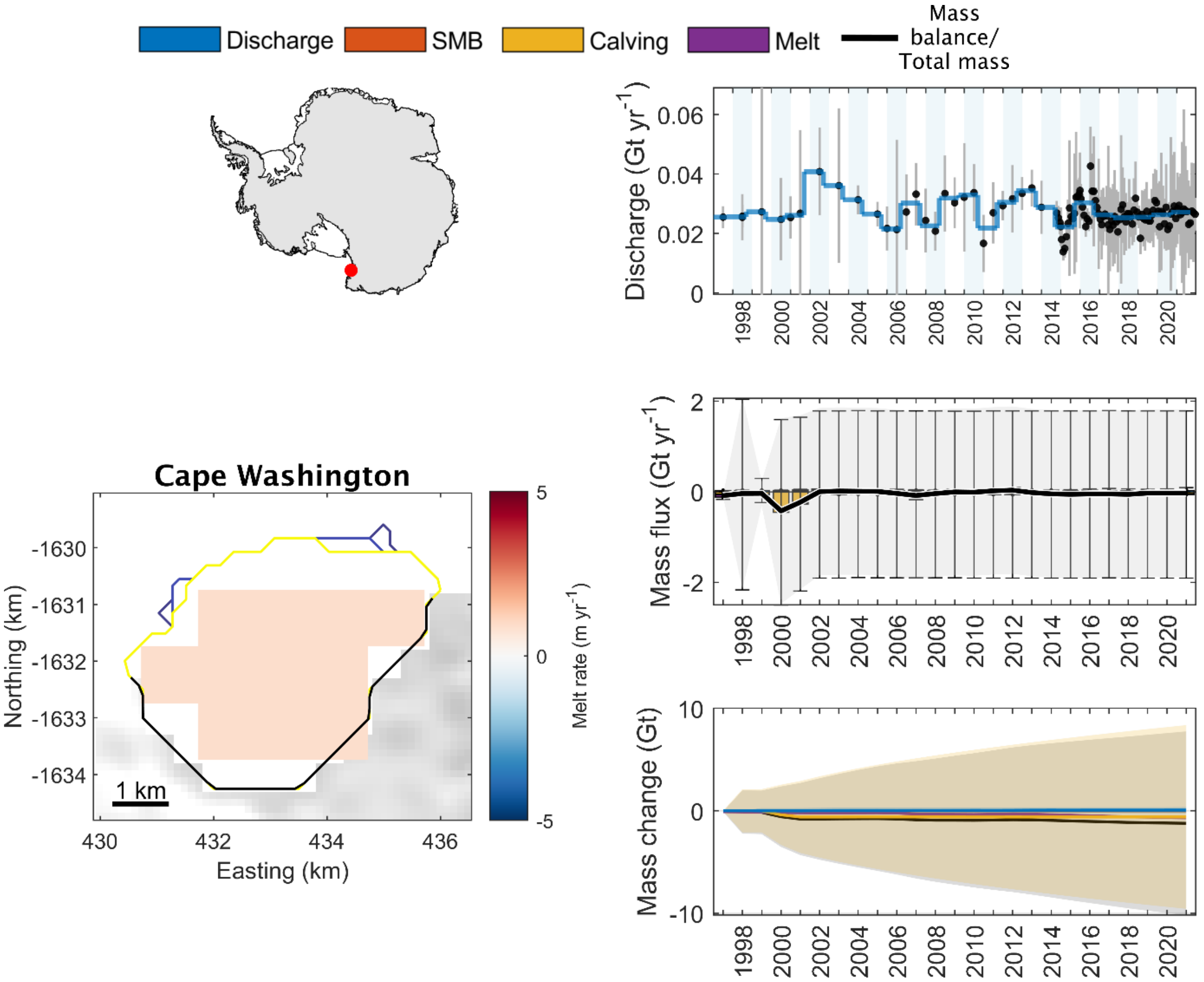
Discharge
SMB
Calving
Melt
Mass balance/ Total mass
Cape Washington
Northing (km)
Easting (km)
1 km
Melt rate (m yr^{-1})
Discharge (Gt yr^{-1})
Mass flux (Gt yr^{-1})
Mass change (Gt)

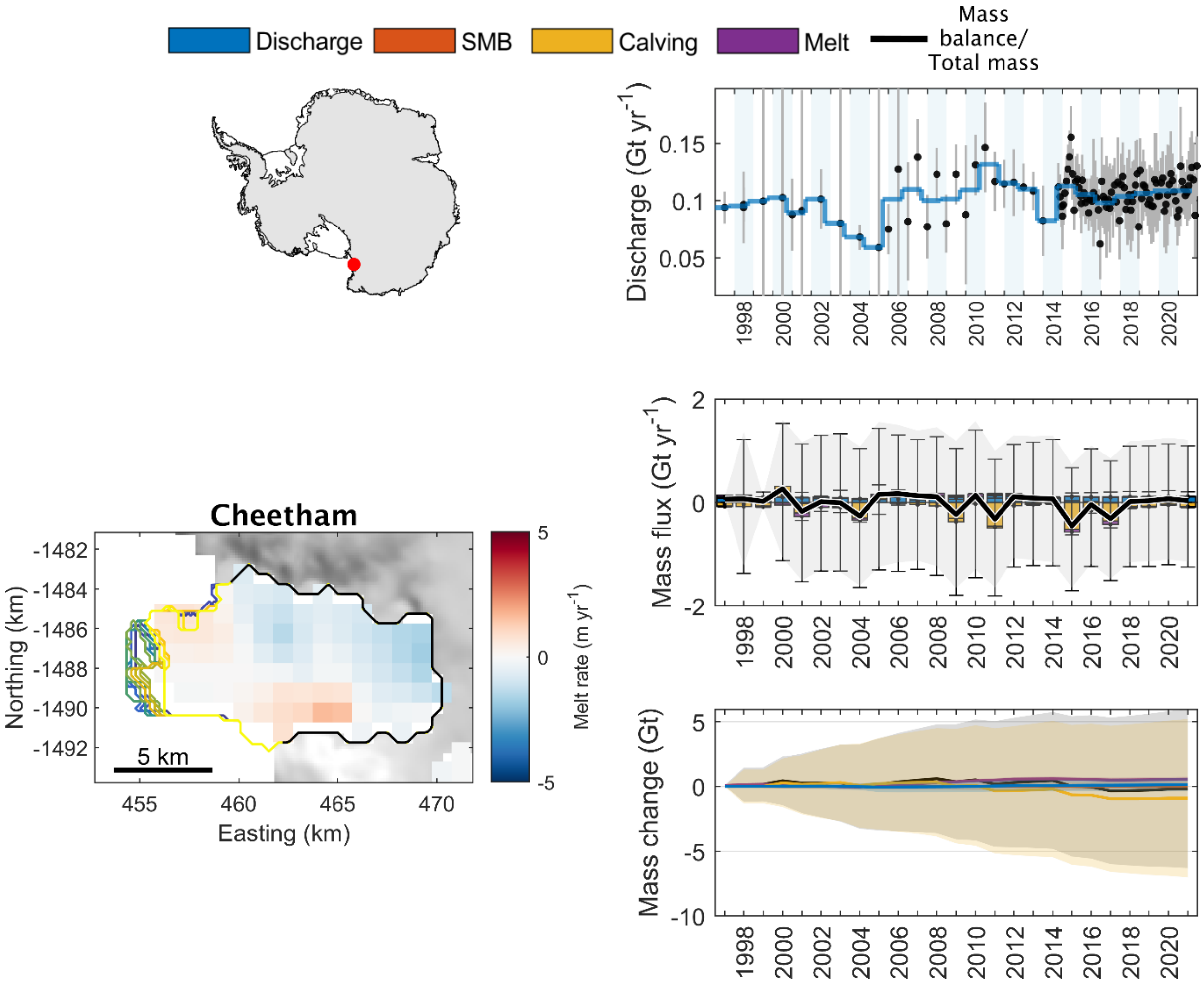
Discharge
SMB
Calving
Melt
Mass balance/ Total mass
Discharge (Gt yr-1)
0.15
0.1
0.05
1998
2000
2002
2004
2006
2008
2010
2012
2014
2016
2018
2020
Cheetham
Northing (km)
-1482
-1484
-1486
-1488
-1490
-1492
5 km
455
460
465
470
Easting (km)
5
0
-5
Melt rate (m yr-1)
Mass flux (Gt yr-1)
2
0
-2
Mass change (Gt)
5
0
-5
-10

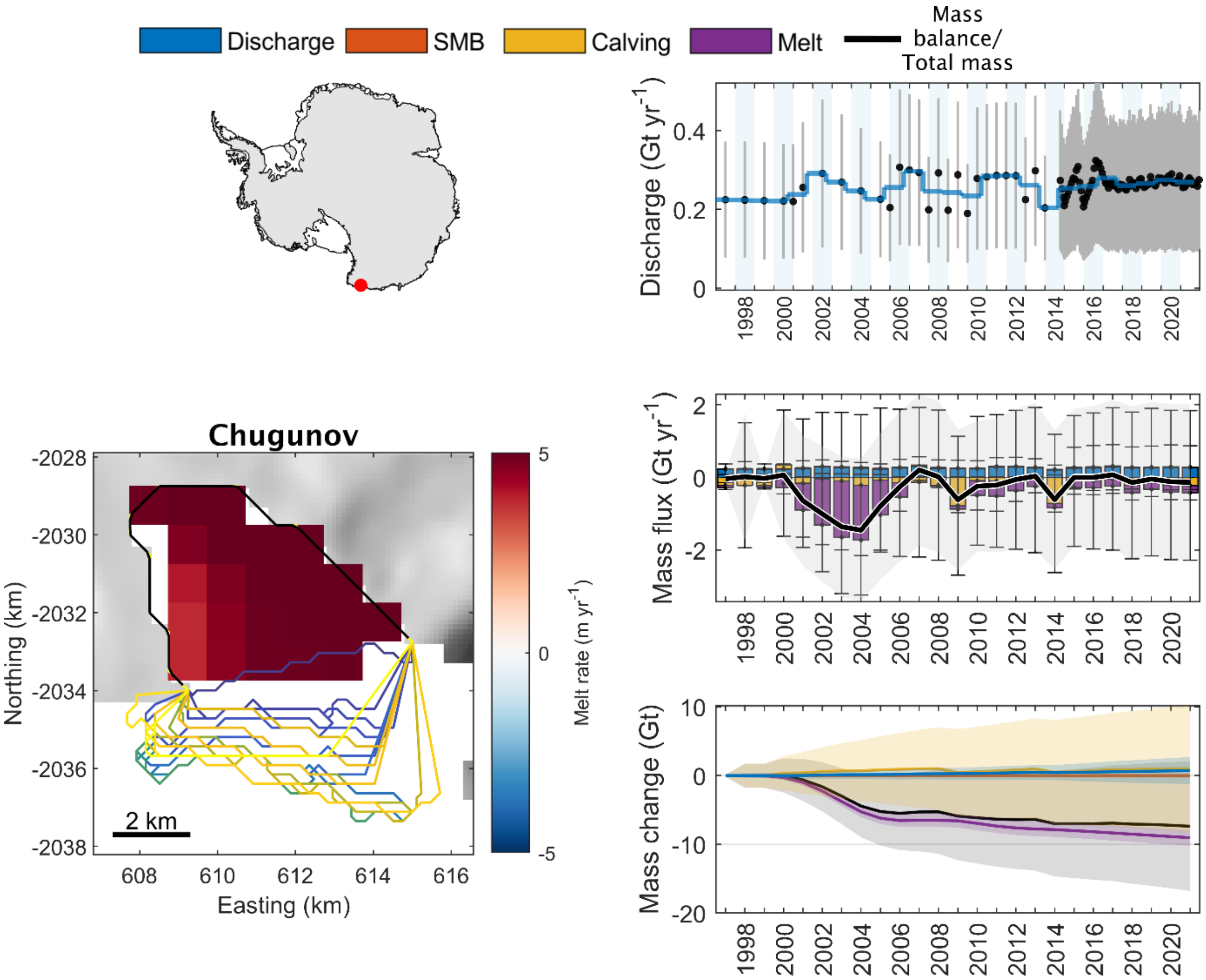

Discharge
SMB
Calving
Melt
Mass balance/ Total mass
Chugunov
Northing (km)
Easting (km)
Melt rate (m yr-1)
2 km
Discharge (Gt yr-1)
Mass flux (Gt yr-1)
Mass change (Gt)

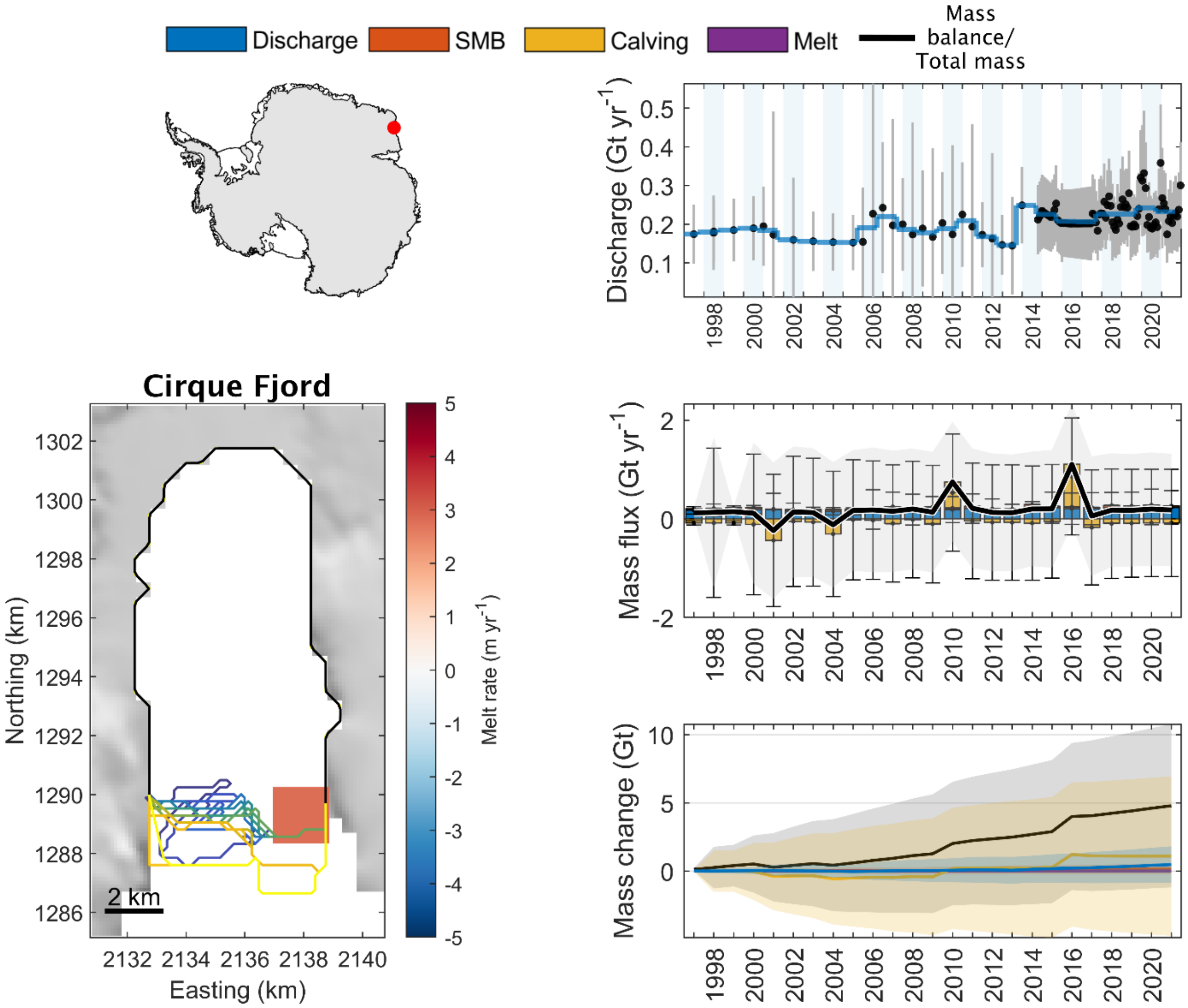

Discharge
SMB
Calving
Melt
Mass balance/ Total mass
Cirque Fjord
Northing (km)
Easting (km)
2 km
Melt rate (m yr-1)
Discharge (Gt yr-1)
Mass flux (Gt yr-1)
Mass change (Gt)

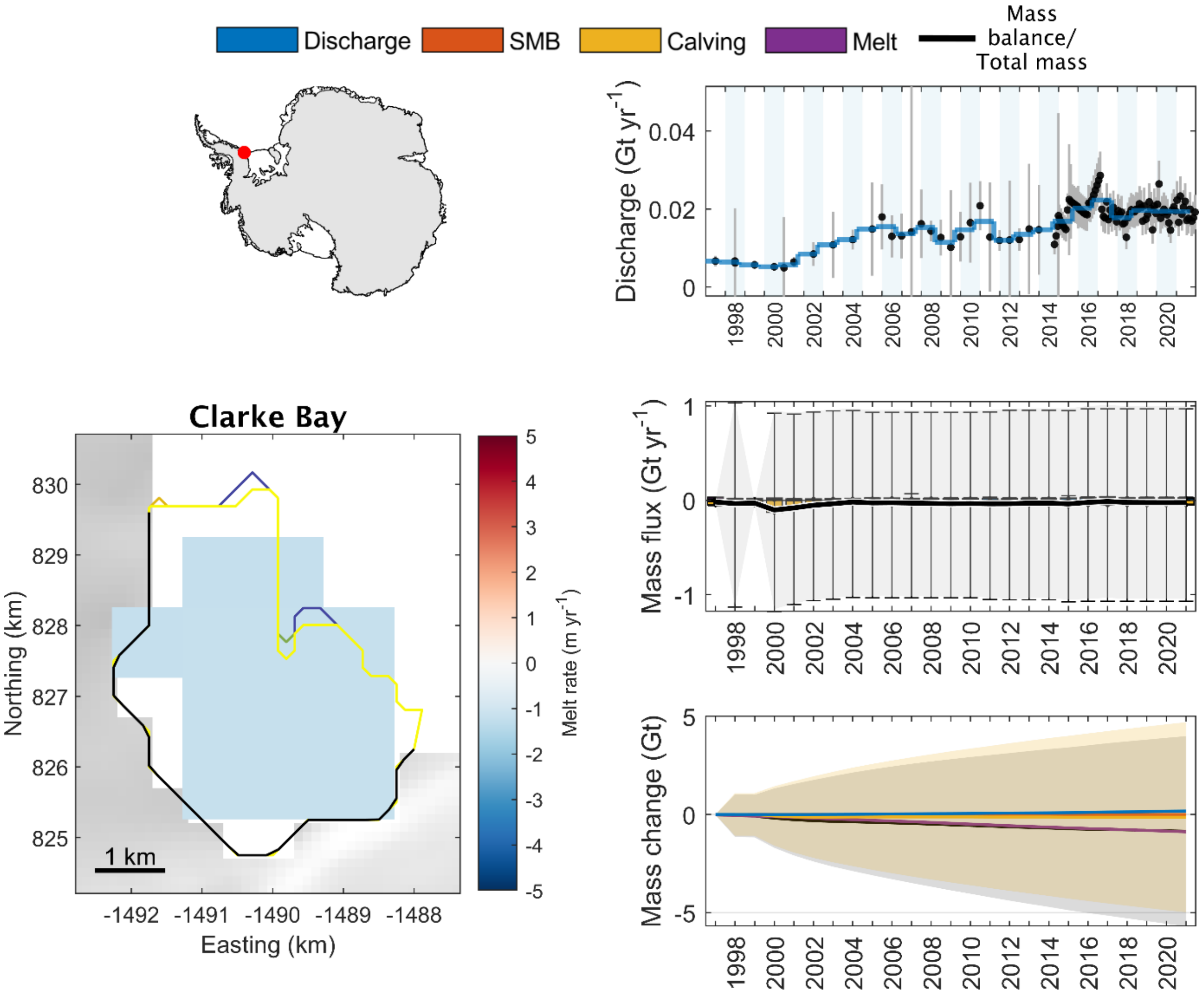
Discharge
SMB
Calving
Melt
Mass balance/ Total mass
Clarke Bay
Northing (km)
Easting (km)
1 km
Melt rate (m yr^{-1})
Discharge (Gt yr^{-1})
Mass flux (Gt yr^{-1})
Mass change (Gt)

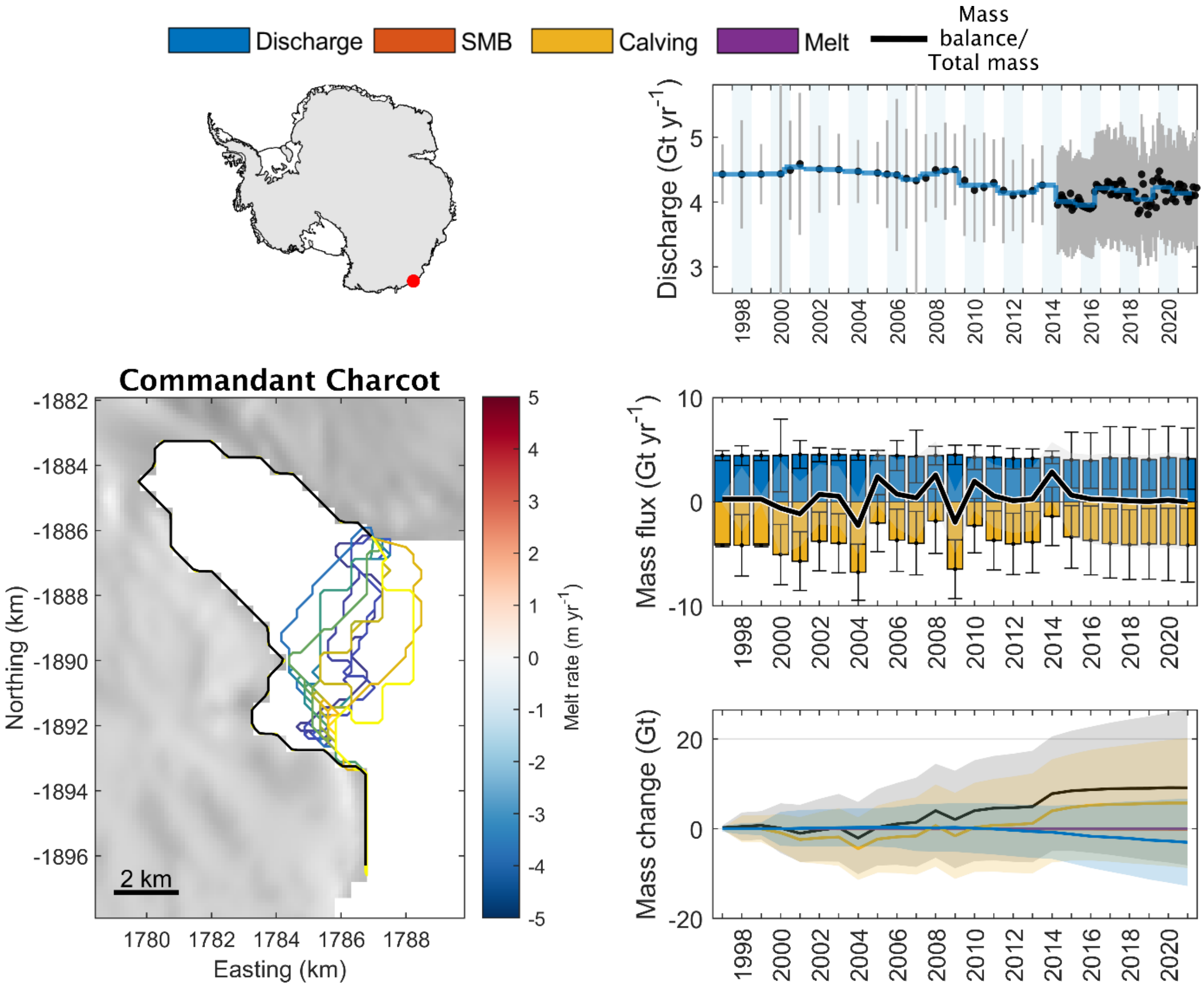
Discharge
SMB
Calving
Melt
Mass balance/ Total mass
Commandant Charcot
Northing (km)
Easting (km)
2 km
Melt rate (m yr-1)
Discharge (Gt yr-1)
Mass flux (Gt yr-1)
Mass change (Gt)

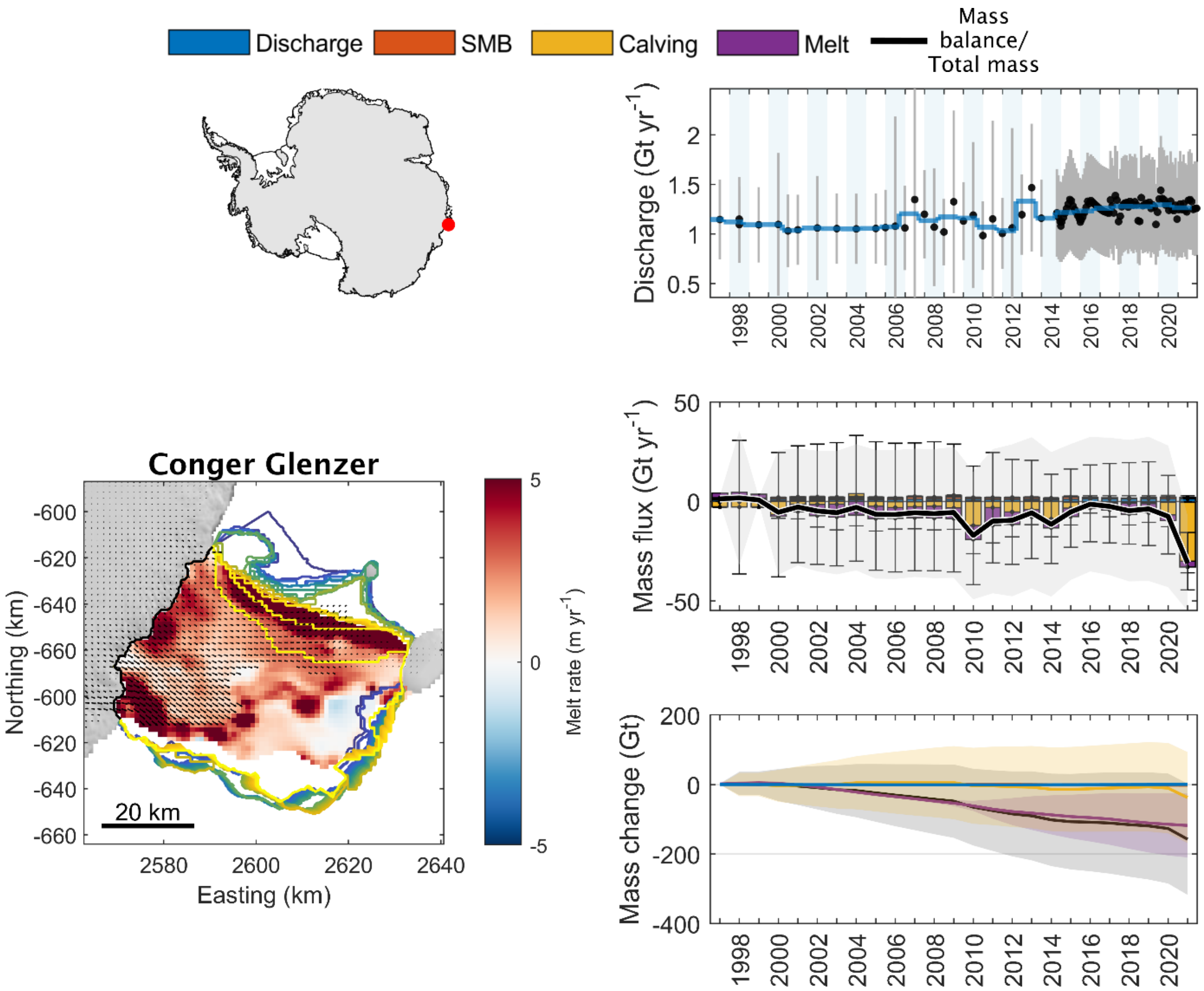

Discharge
SMB
Calving
Melt
Mass balance/ Total mass
Discharge (Gt yr^{-1})
Mass flux (Gt yr^{-1})
Mass change (Gt)
Conger Glenzer
Northing (km)
Easting (km)
Melt rate (m yr^{-1})
20 km

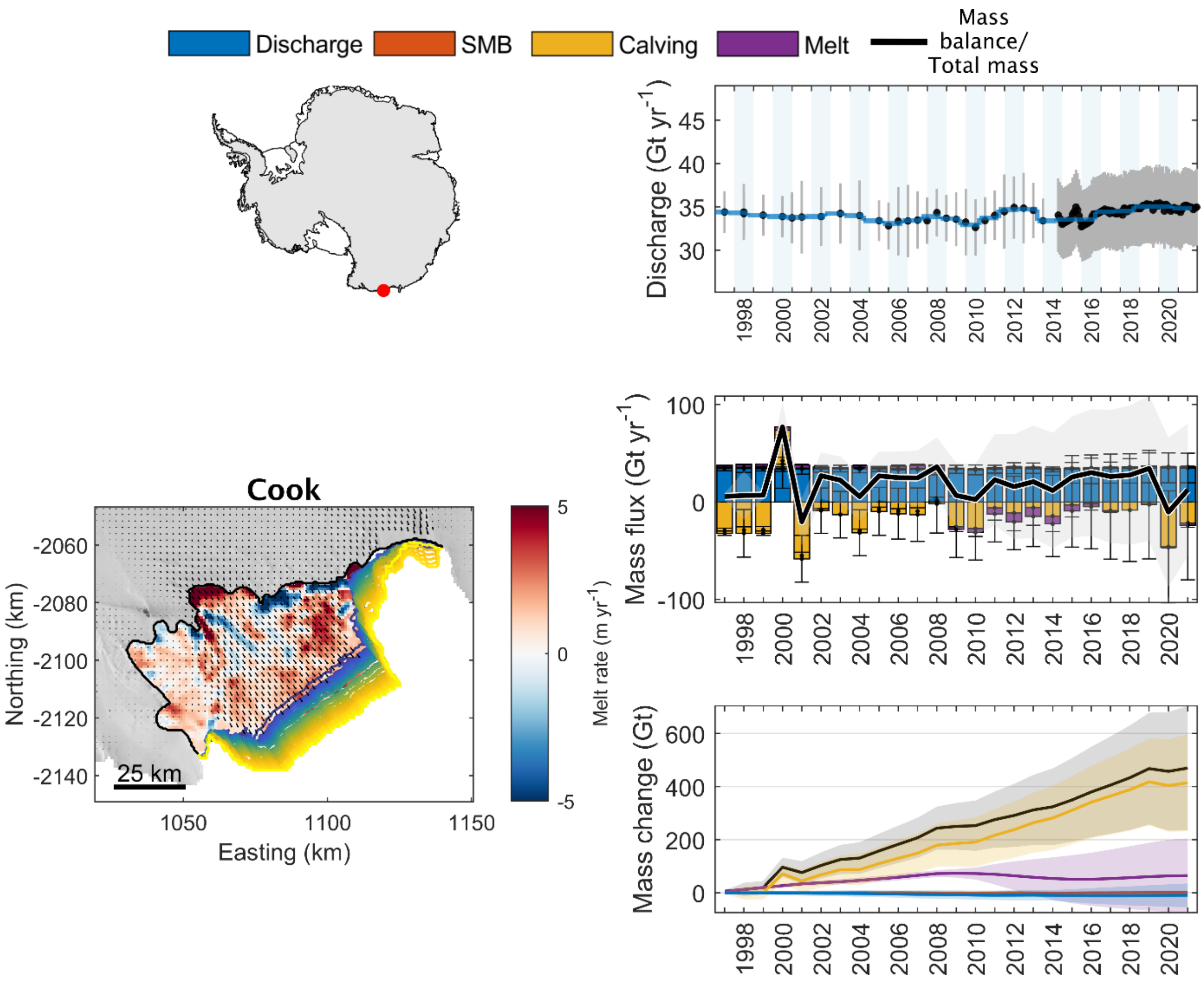

Discharge
SMB
Calving
Melt
Mass balance/ Total mass
Cook
Northing (km)
-2060
-2080
-2100
-2120
-2140
25 km
1050
1100
1150
Easting (km)
5
0
-5
Melt rate (m yr-1)
Discharge (Gt yr-1)
45
40
35
30
Mass flux (Gt yr-1)
100
0
-100
Mass change (Gt)
600
400
200
0
1998
2000
2002
2004
2006
2008
2010
2012
2014
2016
2018
2020

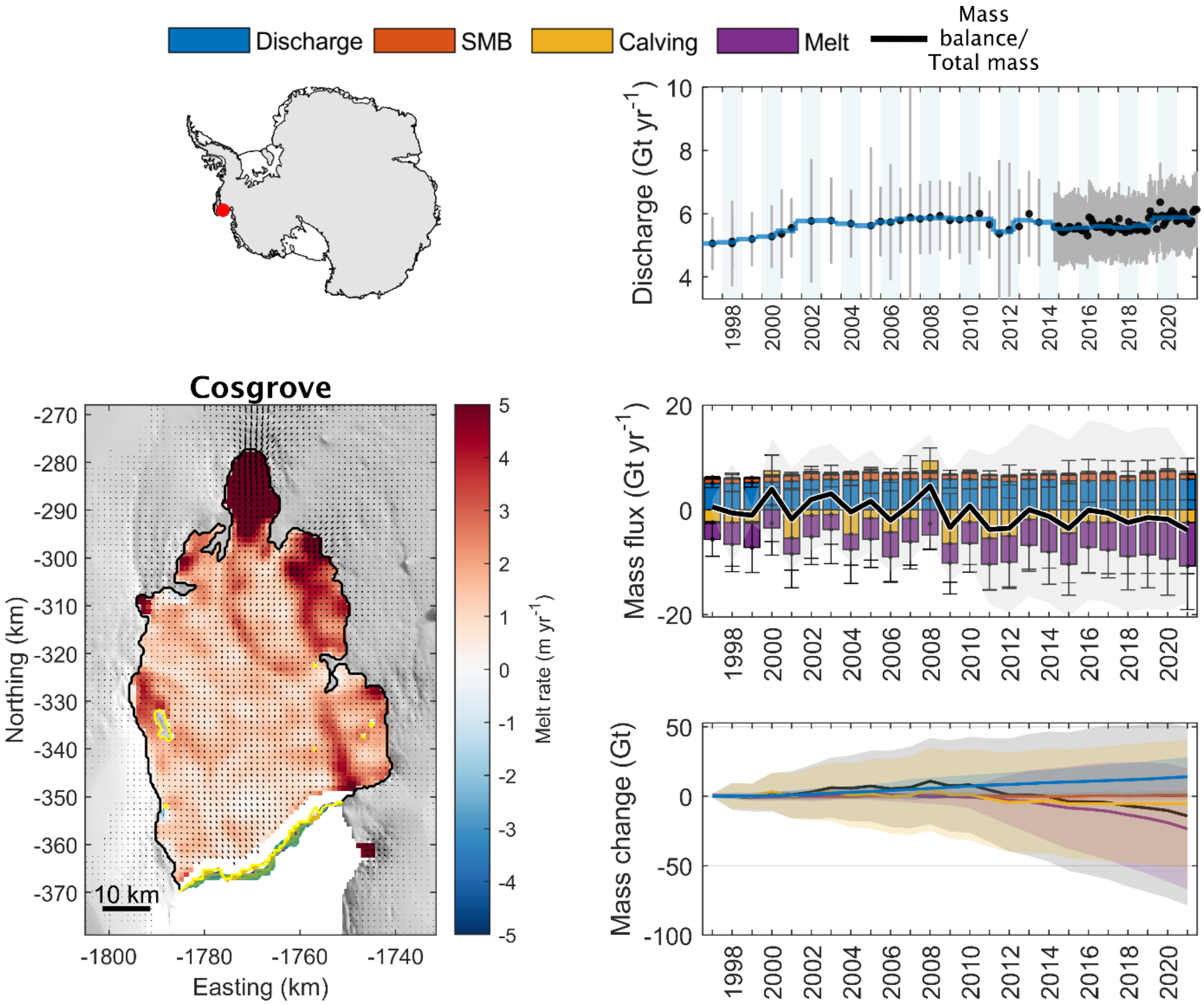
Discharge
SMB
Calving
Melt
Mass balance/ Total mass
Discharge (Gt yr^{-1})
Cosgrove
Northing (km)
Easting (km)
10 km
Melt rate (m yr^{-1})
Mass flux (Gt yr^{-1})
Mass change (Gt)

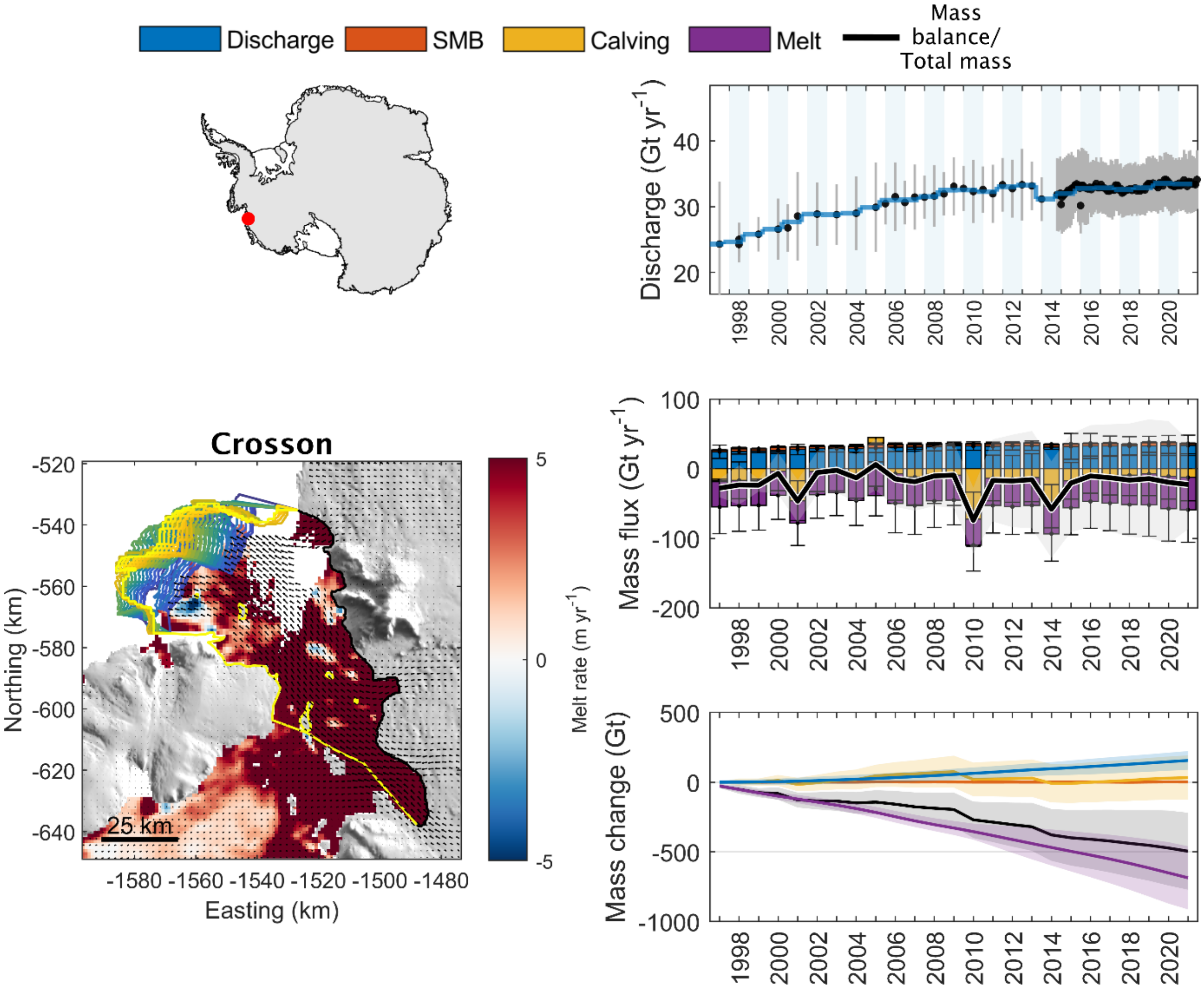
Discharge
SMB
Calving
Melt
Mass balance/ Total mass
Discharge (Gt yr-1)
Mass flux (Gt yr-1)
Mass change (Gt)
Crosson
Northing (km)
Easting (km)
25 km
Melt rate (m yr-1)
1998
2000
2002
2004
2006
2008
2010
2012
2014
2016
2018
2020
-1580 -1560 -1540 -1520 -1500 -1480
-520
-540
-560
-580
-600
-620
-640
5
0
-5
40
30
20
100
0
-100
-200
500
0
-500
-1000

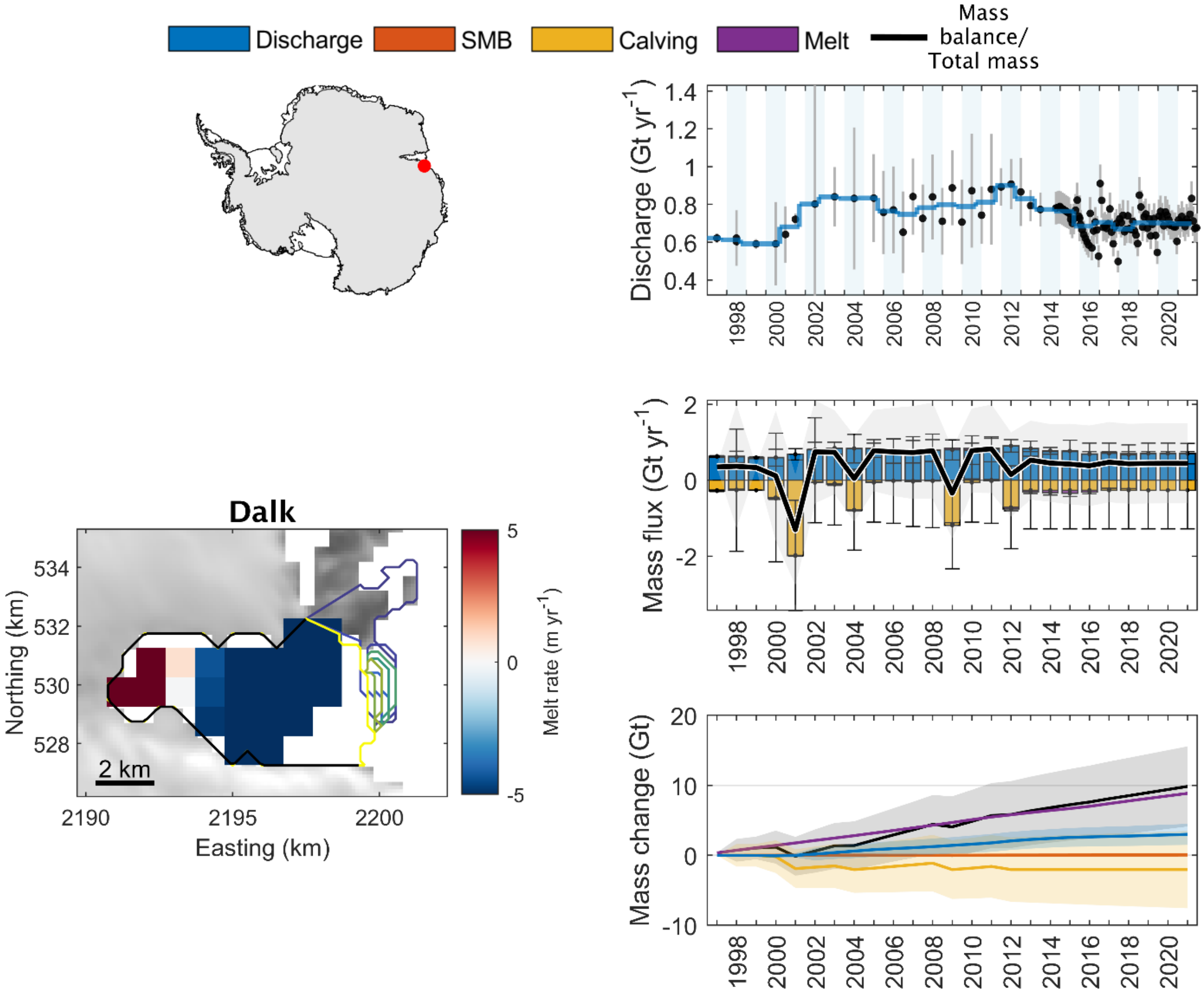

Discharge
SMB
Calving
Melt
Mass balance/ Total mass
Discharge (Gt yr-1)
Dalk
Northing (km)
Easting (km)
2 km
Melt rate (m yr-1)
Mass flux (Gt yr-1)
Mass change (Gt)

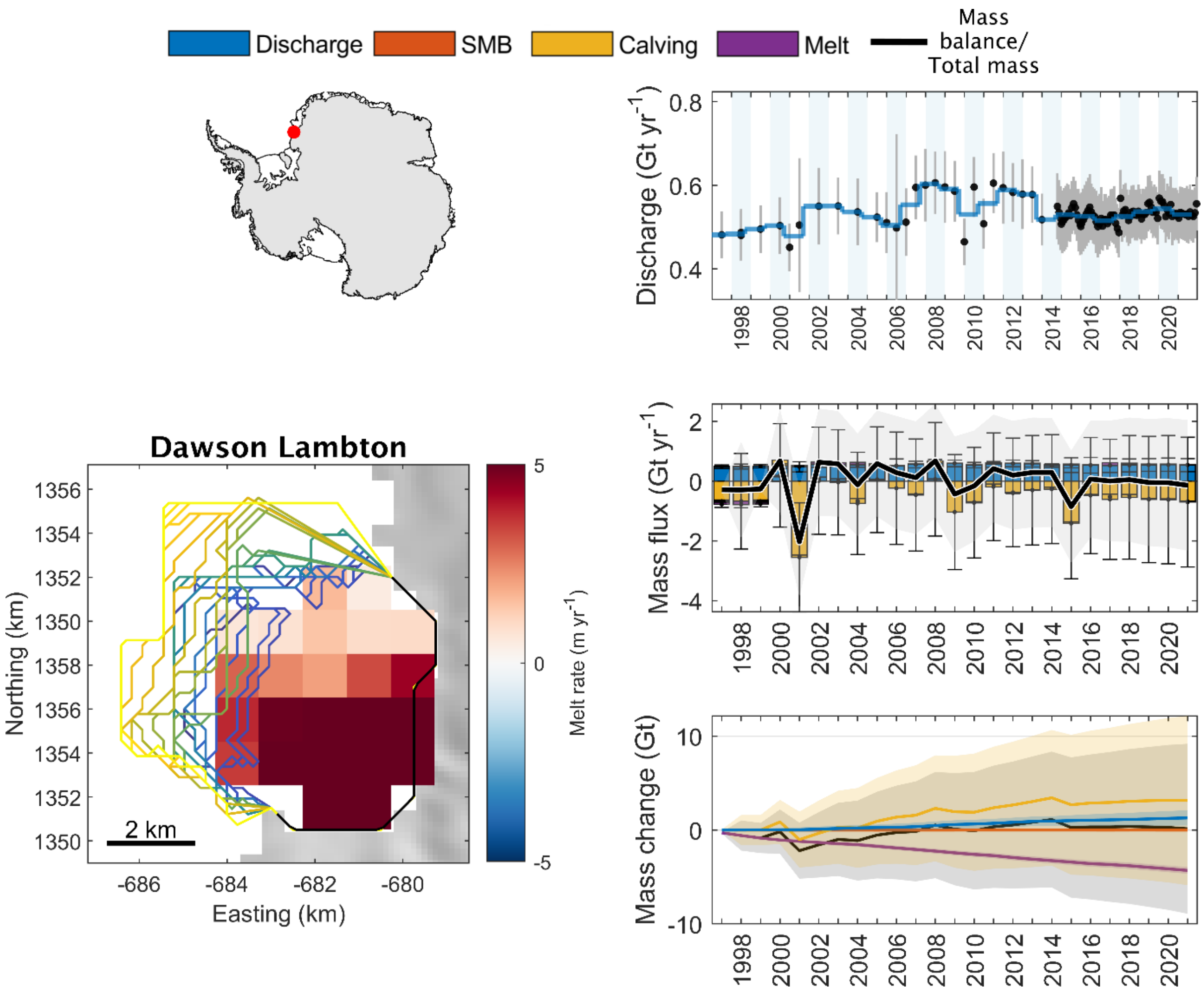
Discharge
SMB
Calving
Melt
Mass balance/ Total mass
Dawson Lambton
Northing (km)
Easting (km)
2 km
Melt rate (m yr^{-1})
Discharge (Gt yr^{-1})
Mass flux (Gt yr^{-1})
Mass change (Gt)

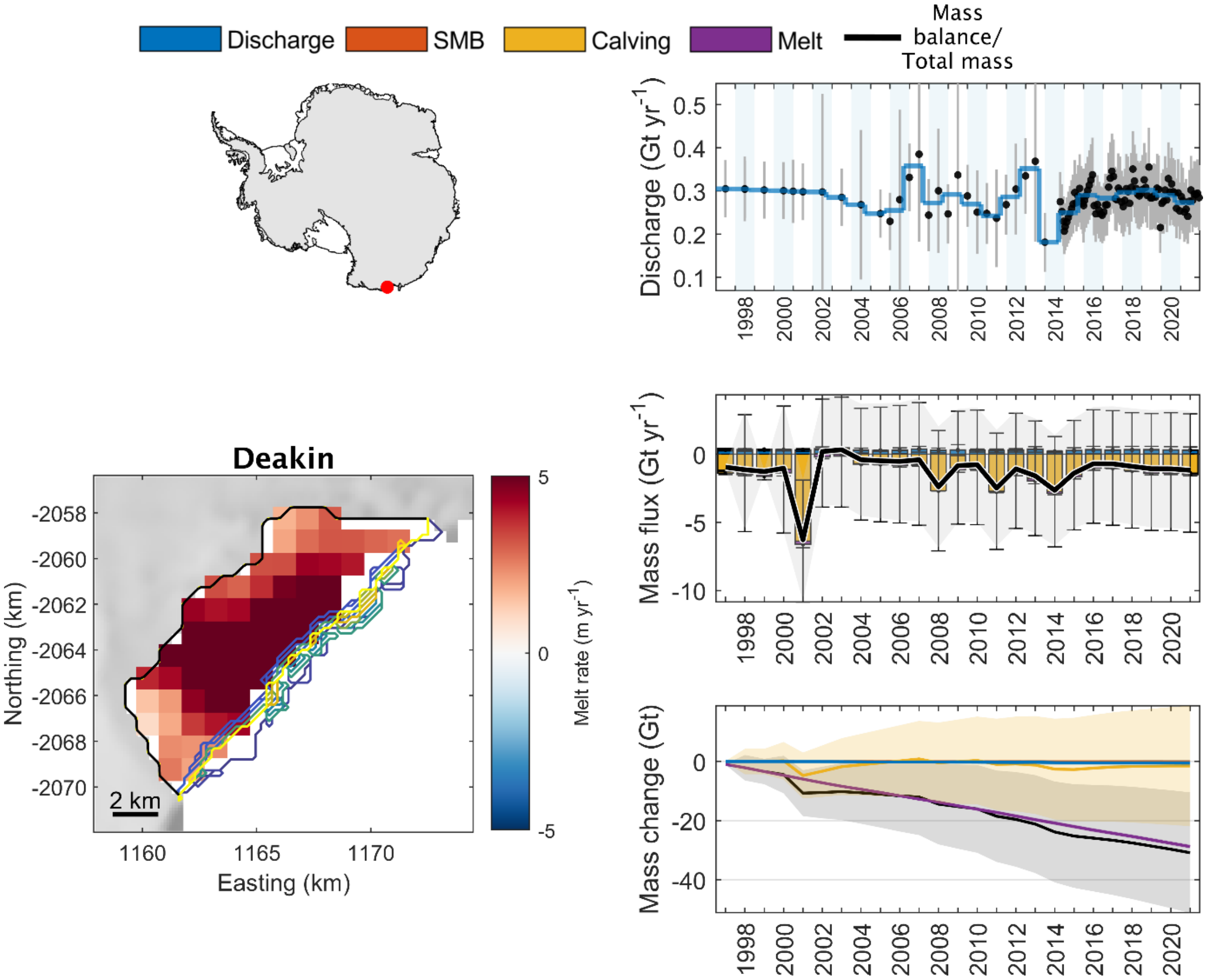

Discharge
SMB
Calving
Melt
Mass balance/ Total mass
Deakin
Northing (km)
Easting (km)
2 km
Melt rate (m yr-1)
Discharge (Gt yr-1)
Mass flux (Gt yr-1)
Mass change (Gt)

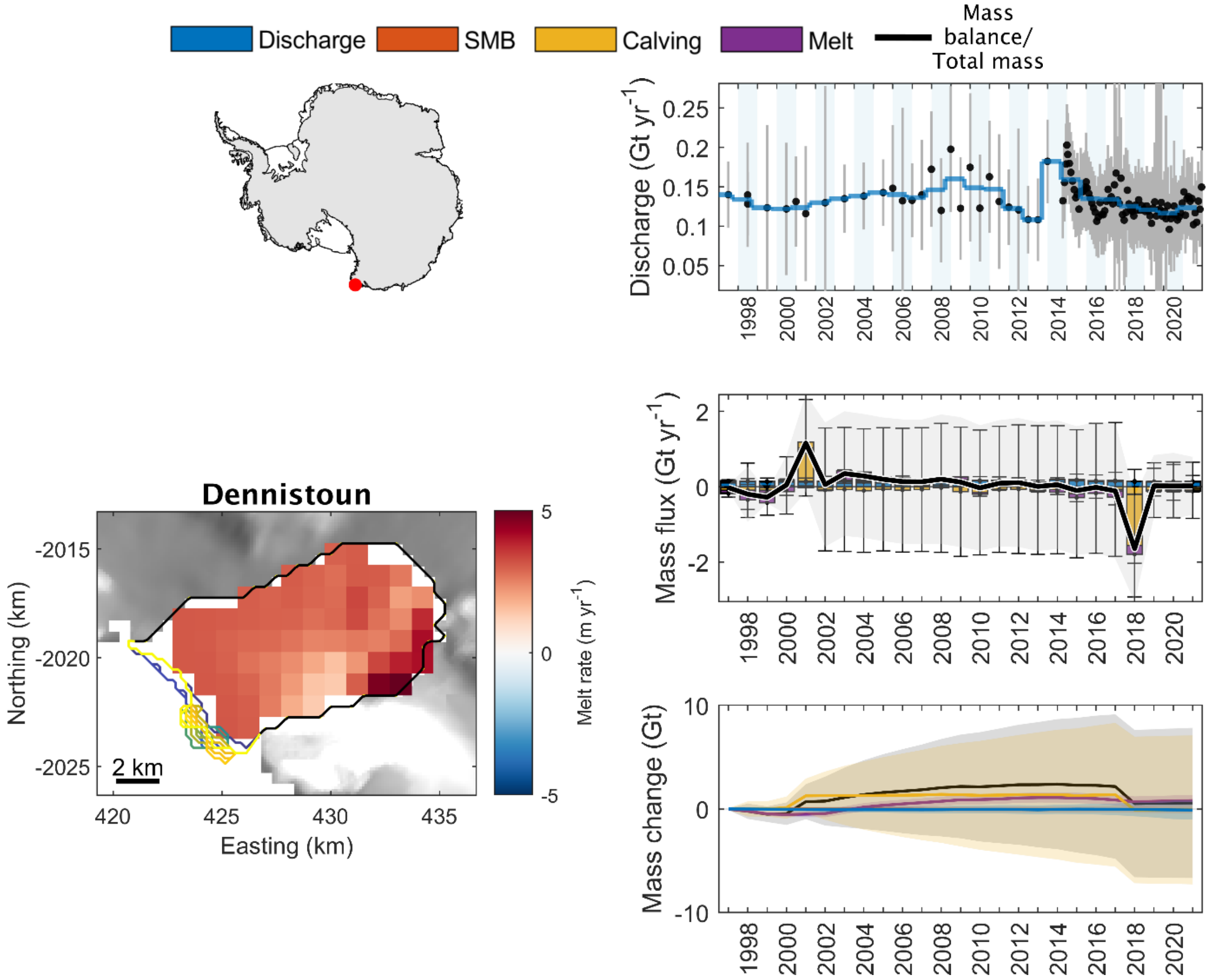

Discharge
SMB
Calving
Melt
Mass balance/ Total mass
Dennistoun
Northing (km)
-2015
-2020
-2025
2 km
420
425
430
435
Easting (km)
5
0
-5
Melt rate (m yr^{-1})
Discharge (Gt yr^{-1})
0.25
0.2
0.15
0.1
0.05
Mass flux (Gt yr^{-1})
2
0
-2
Mass change (Gt)
10
0
-10
1998
2000
2002
2004
2006
2008
2010
2012
2014
2016
2018
2020

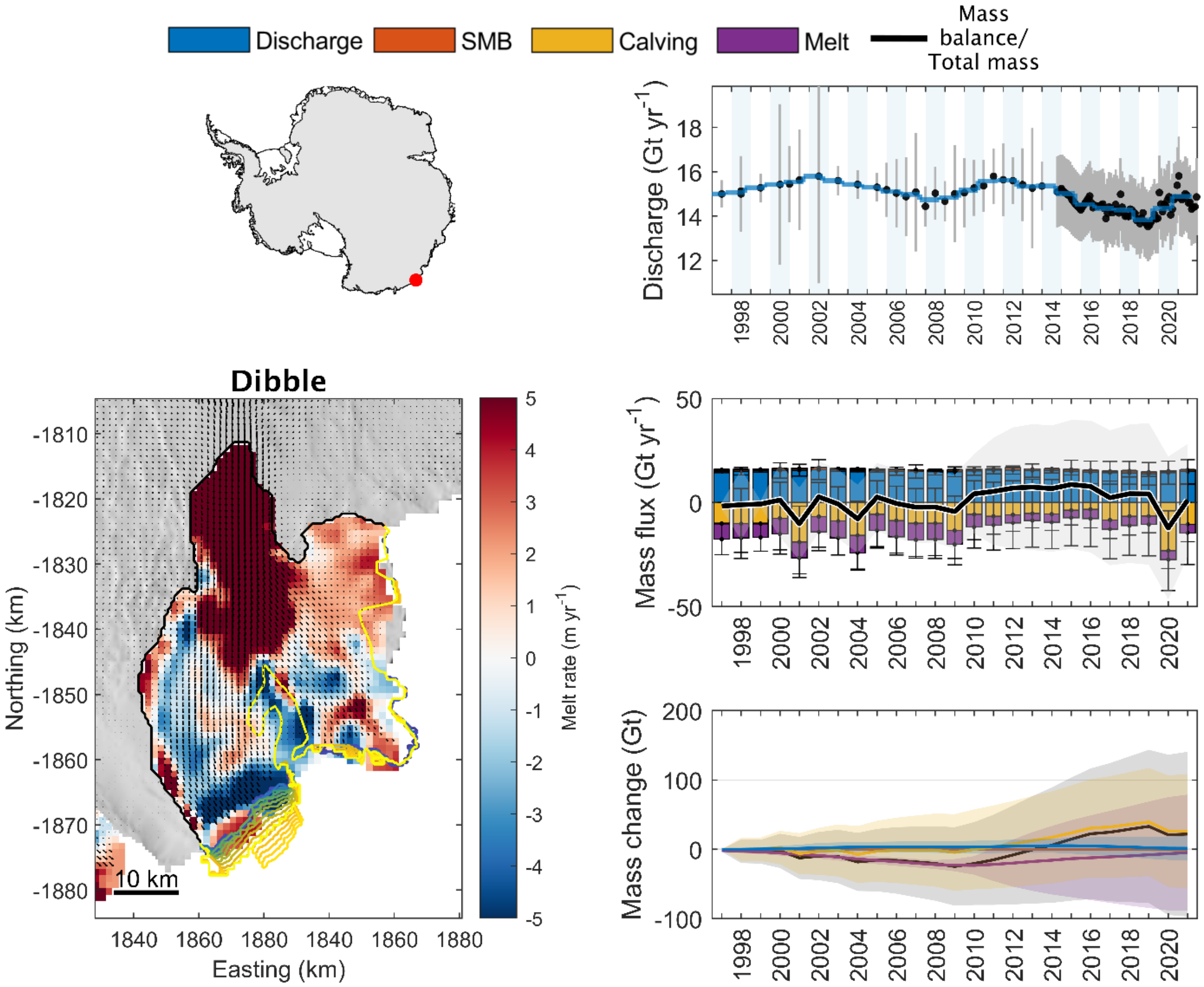
Discharge
SMB
Calving
Melt
Mass balance/ Total mass
Discharge (Gt yr-1)
Dibble
Northing (km)
Easting (km)
10 km
Melt rate (m yr-1)
Mass flux (Gt yr-1)
Mass change (Gt)

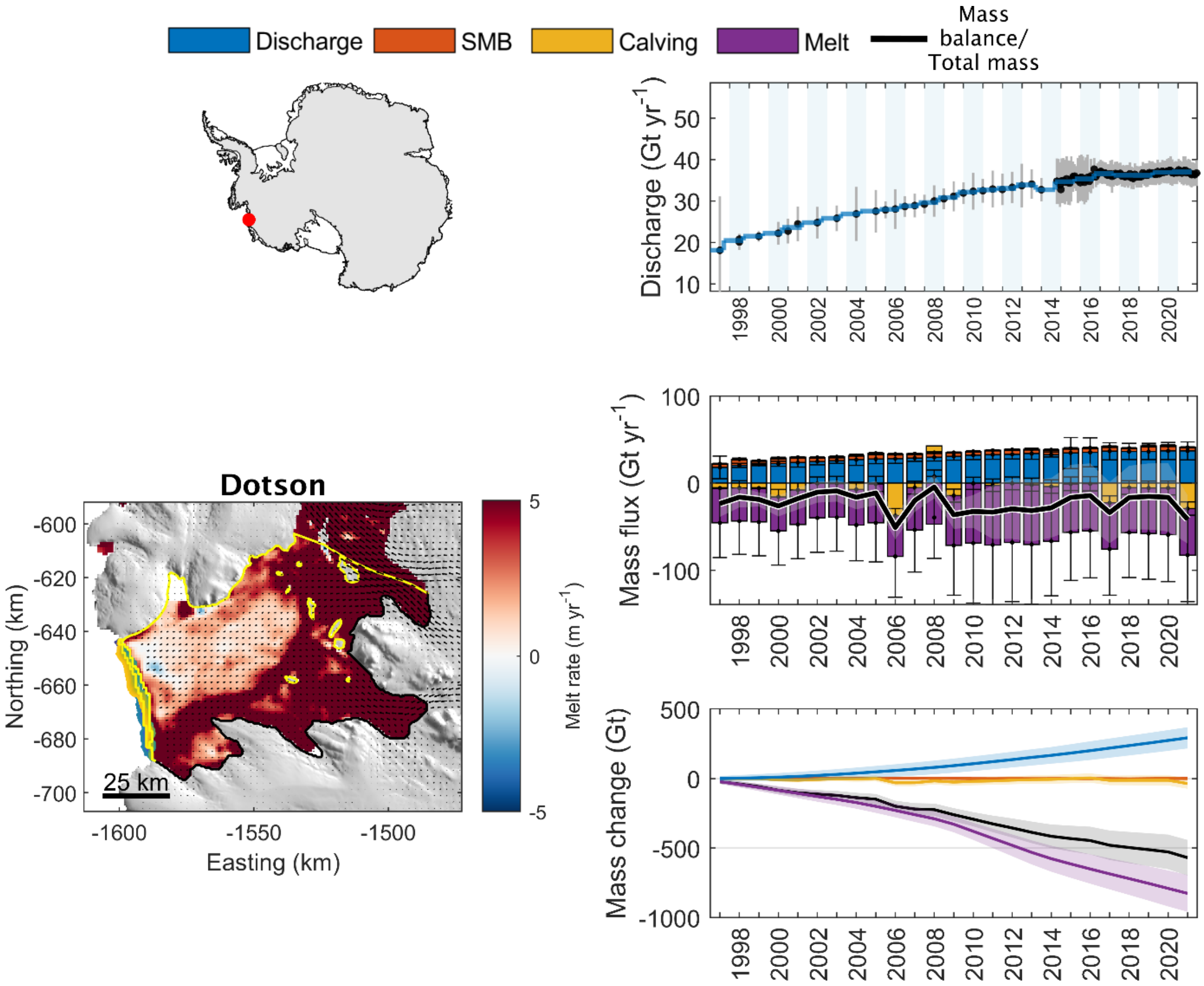
Discharge
SMB
Calving
Melt
Mass balance/ Total mass
Dotson
Northing (km)
Easting (km)
25 km
Melt rate (m yr-1)
Discharge (Gt yr-1)
Mass flux (Gt yr-1)
Mass change (Gt)

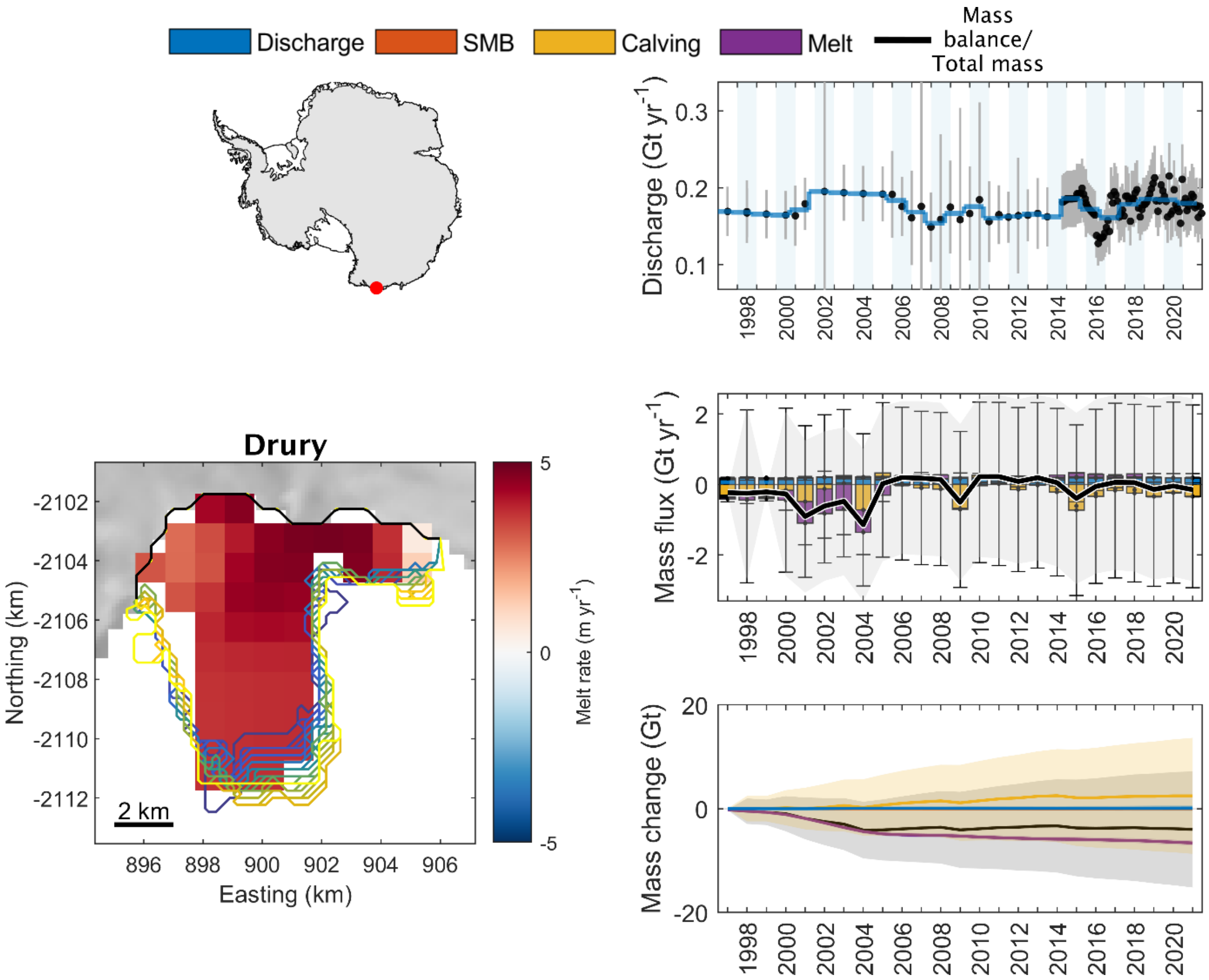

Discharge
SMB
Calving
Melt
Mass balance/ Total mass
Drury
Northing (km)
Easting (km)
2 km
Melt rate (m yr^{-1})
Discharge (Gt yr^{-1})
Mass flux (Gt yr^{-1})
Mass change (Gt)

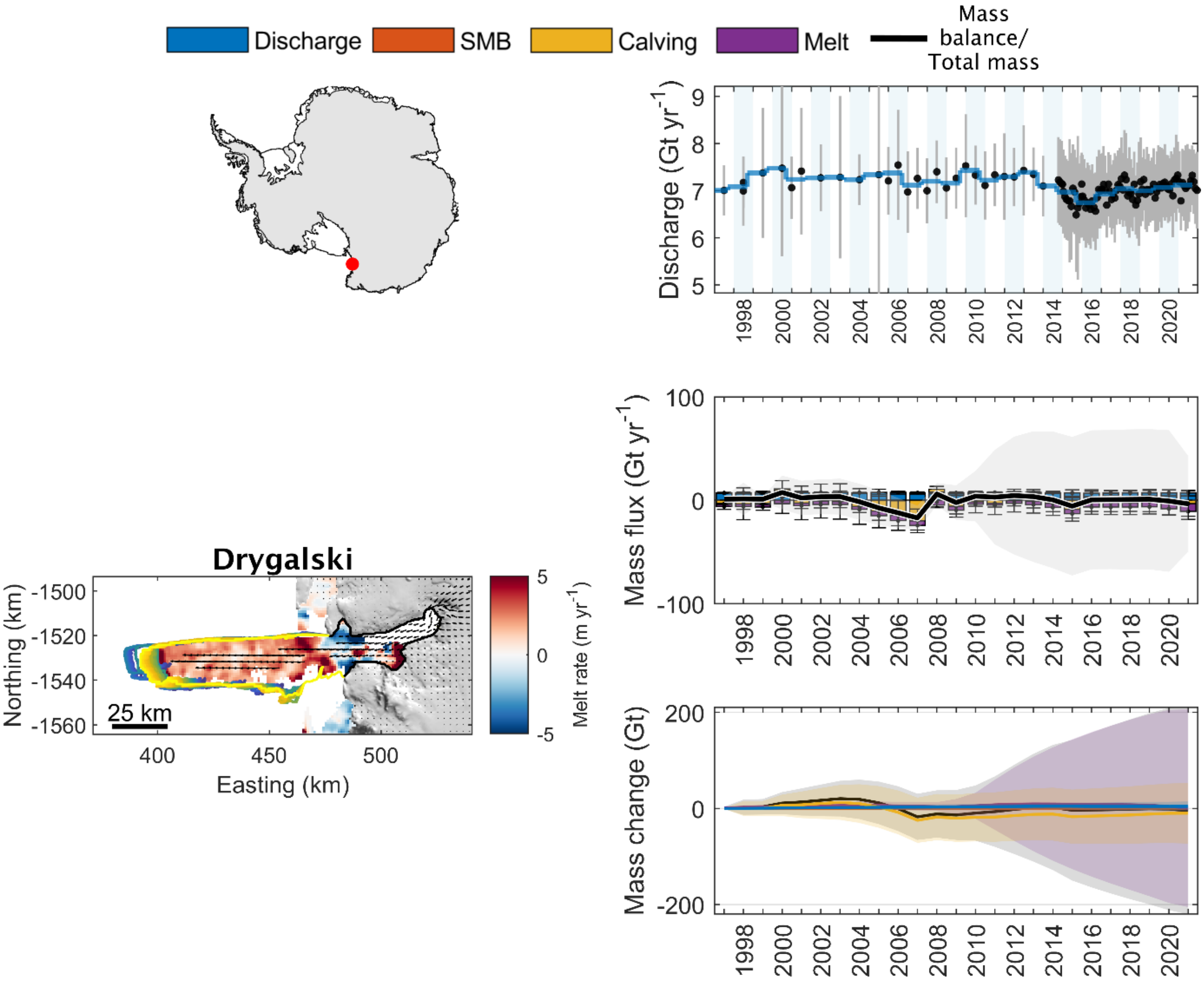

Discharge
SMB
Calving
Melt
Mass balance/ Total mass
Discharge (Gt yr^{-1})
Mass flux (Gt yr^{-1})
Mass change (Gt)
Drygalski
Northing (km)
Easting (km)
25 km
Melt rate (m yr^{-1})

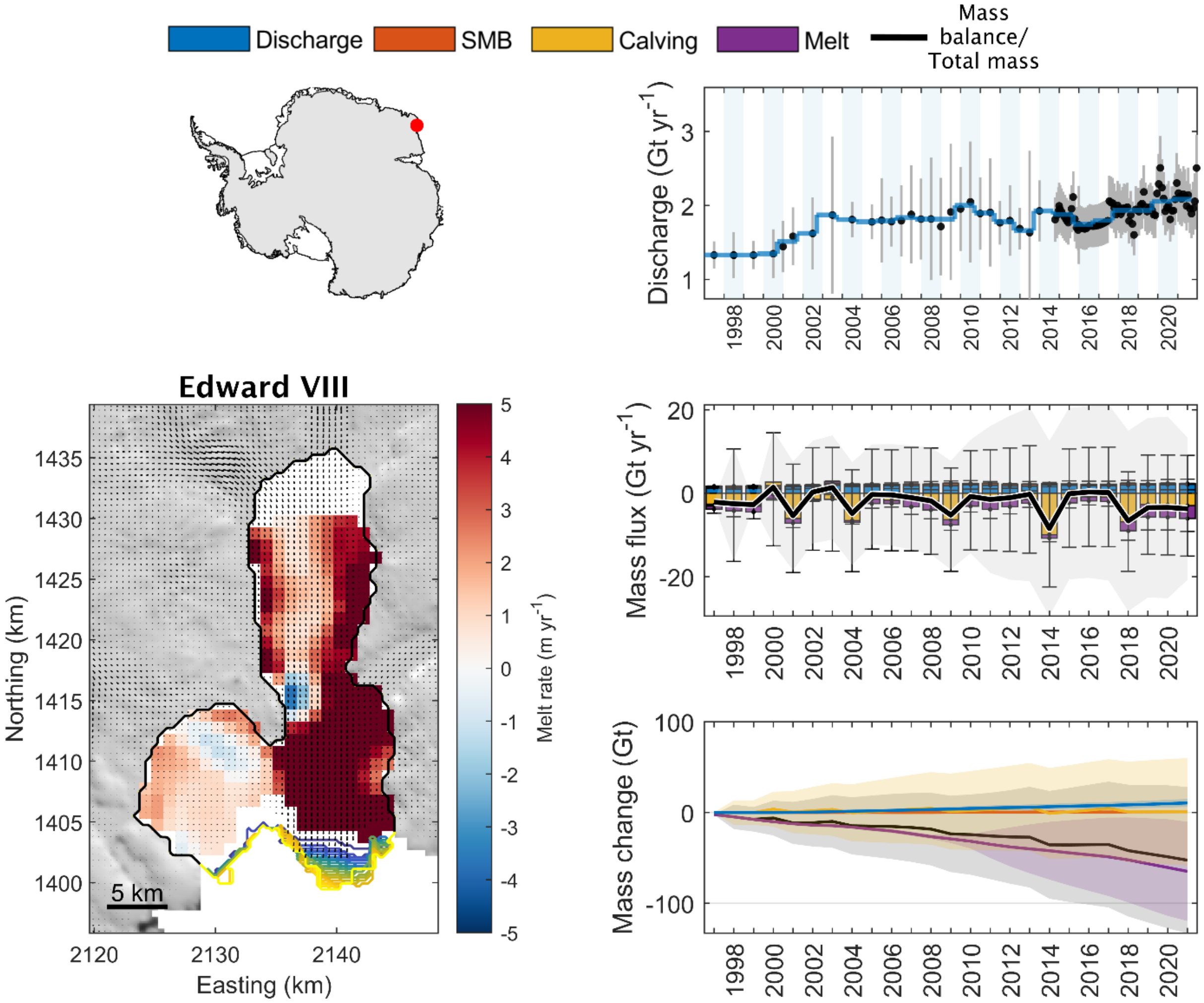

Discharge
SMB
Calving
Melt
Mass balance/ Total mass
Edward VIII
Northing (km)
Easting (km)
5 km
Melt rate (m yr^{-1})
Discharge (Gt yr^{-1})
Mass flux (Gt yr^{-1})
Mass change (Gt)

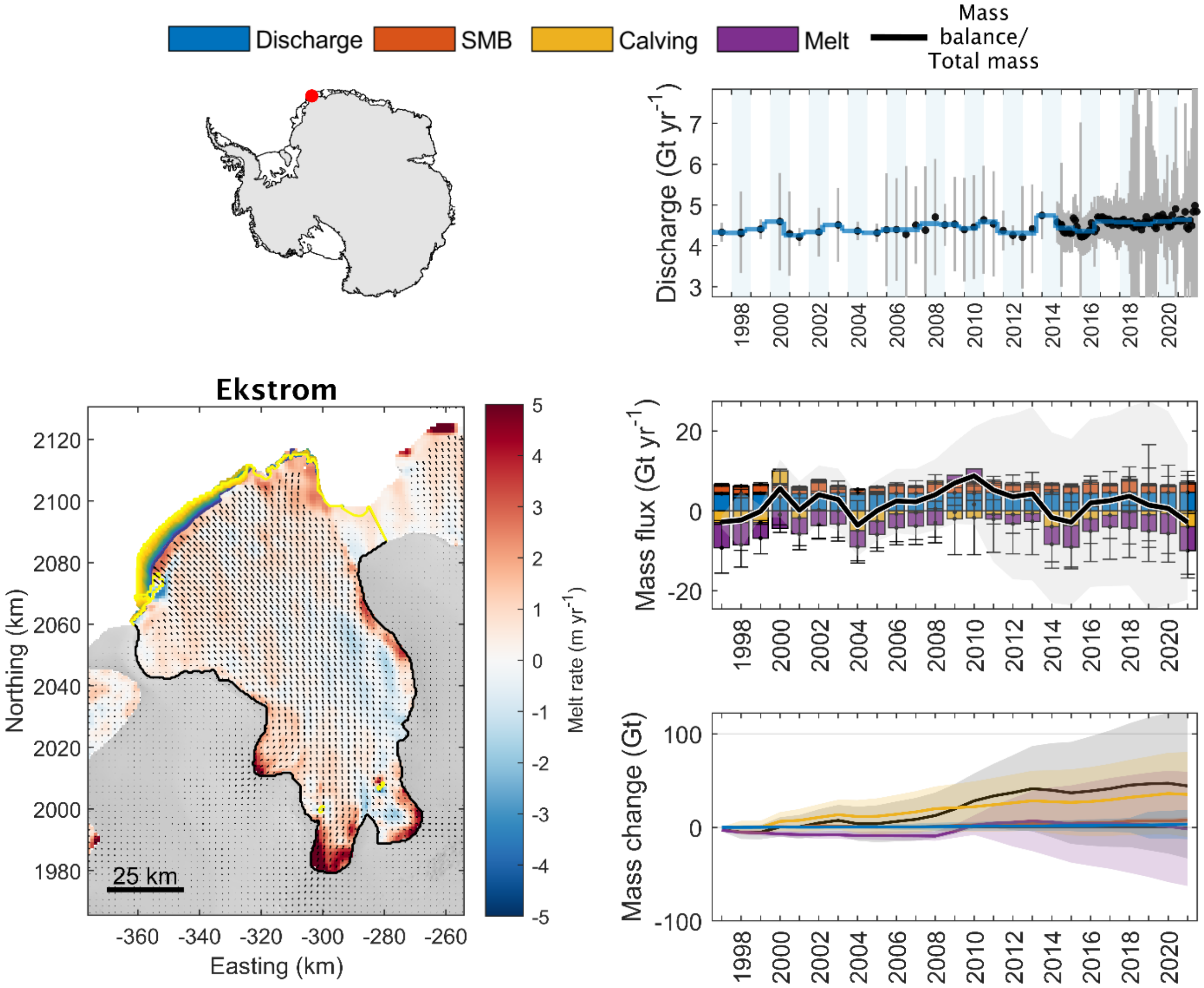

Discharge
SMB
Calving
Melt
Mass balance/ Total mass
Discharge (Gt yr^{-1})
Ekstrom
Northing (km)
Easting (km)
25 km
Melt rate (m yr^{-1})
Mass flux (Gt yr^{-1})
Mass change (Gt)

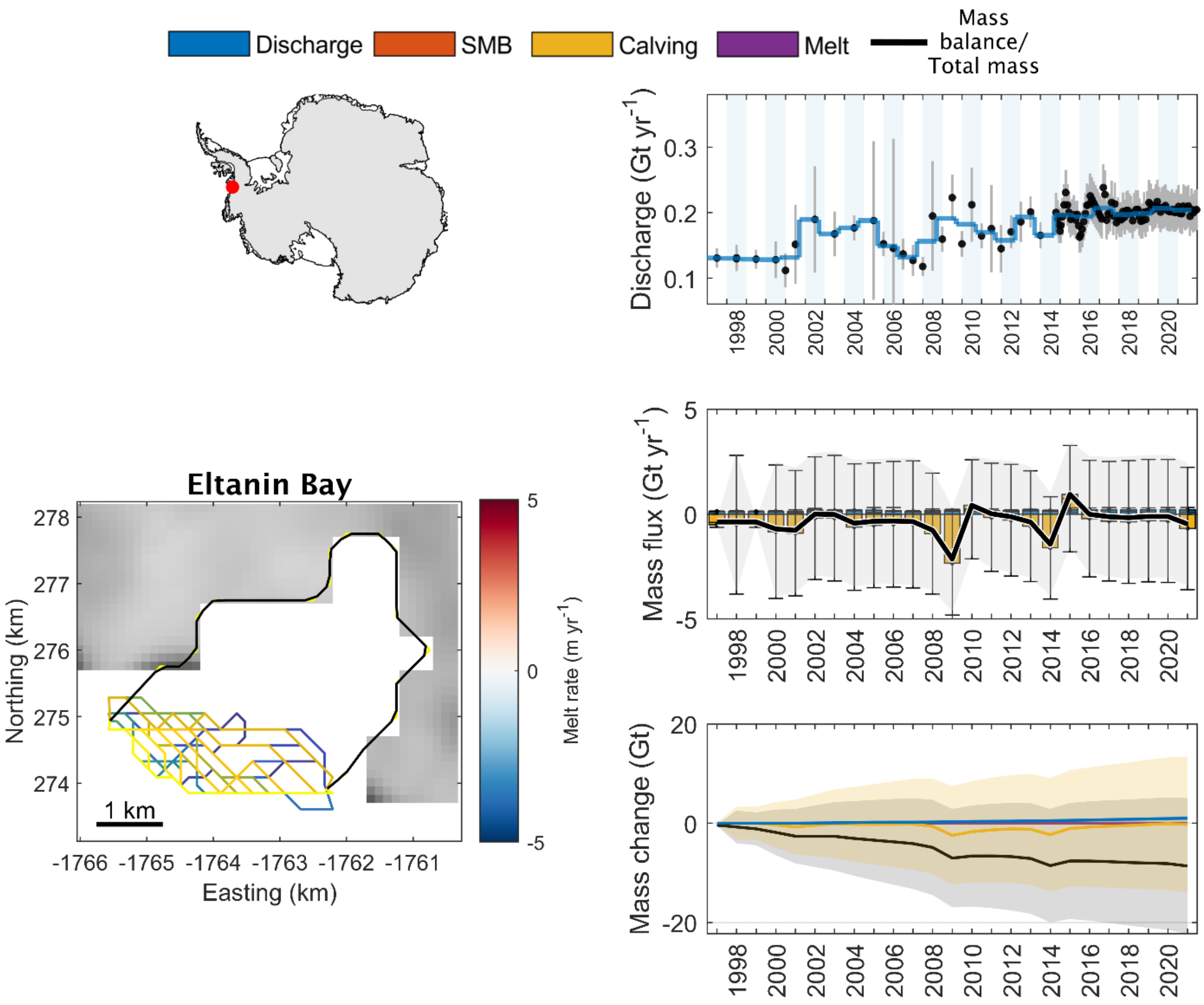

Discharge
SMB
Calving
Melt
Mass balance/ Total mass
Eltanin Bay
1 km
Northing (km)
Easting (km)
Melt rate (m yr-1)
Discharge (Gt yr-1)
Mass flux (Gt yr-1)
Mass change (Gt)

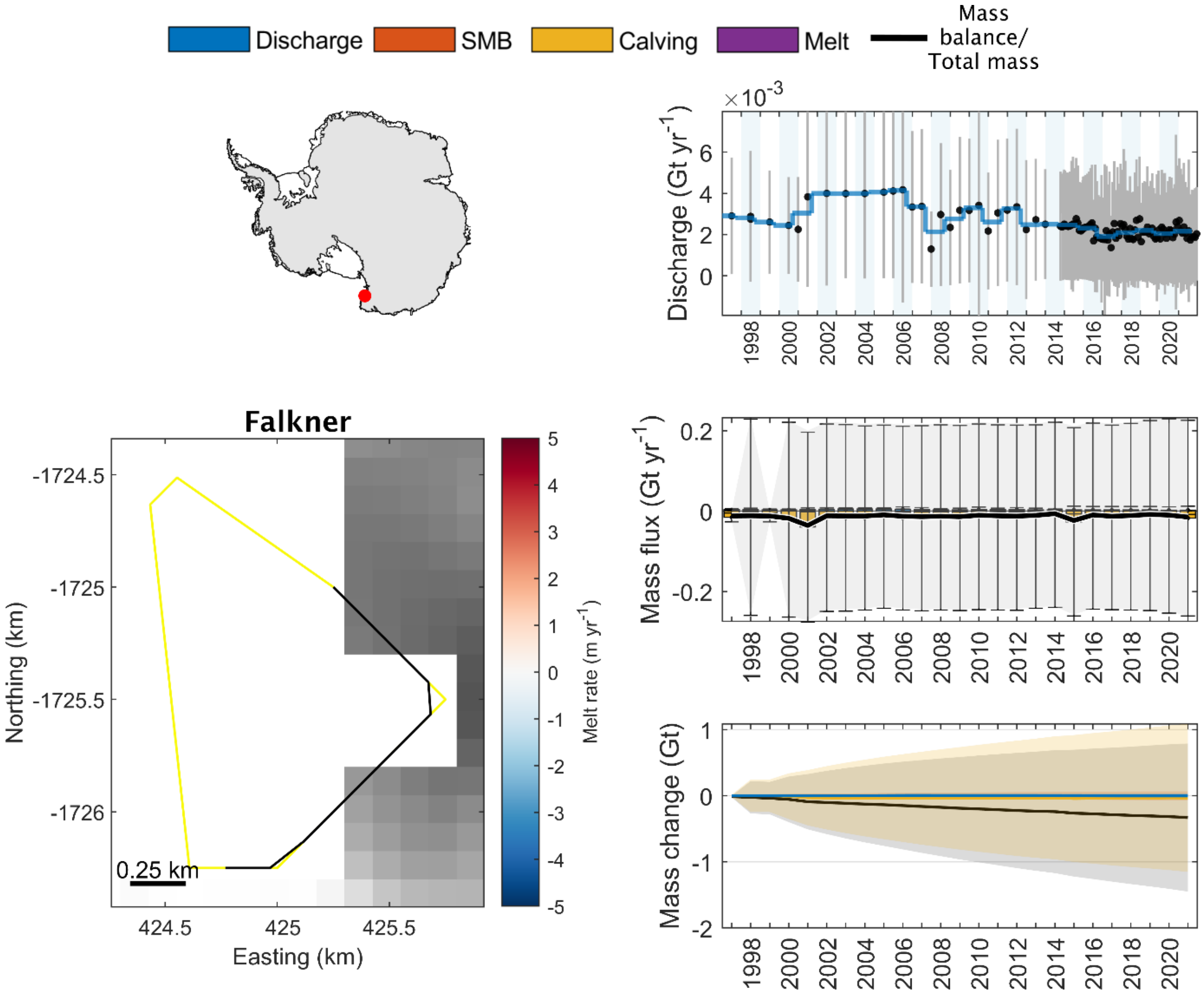
Discharge
SMB
Calving
Melt
Mass balance/ Total mass
Falkner
Northing (km)
Easting (km)
0.25 km
Melt rate (m yr-1)
Discharge (Gt yr-1)
Mass flux (Gt yr-1)
Mass change (Gt)

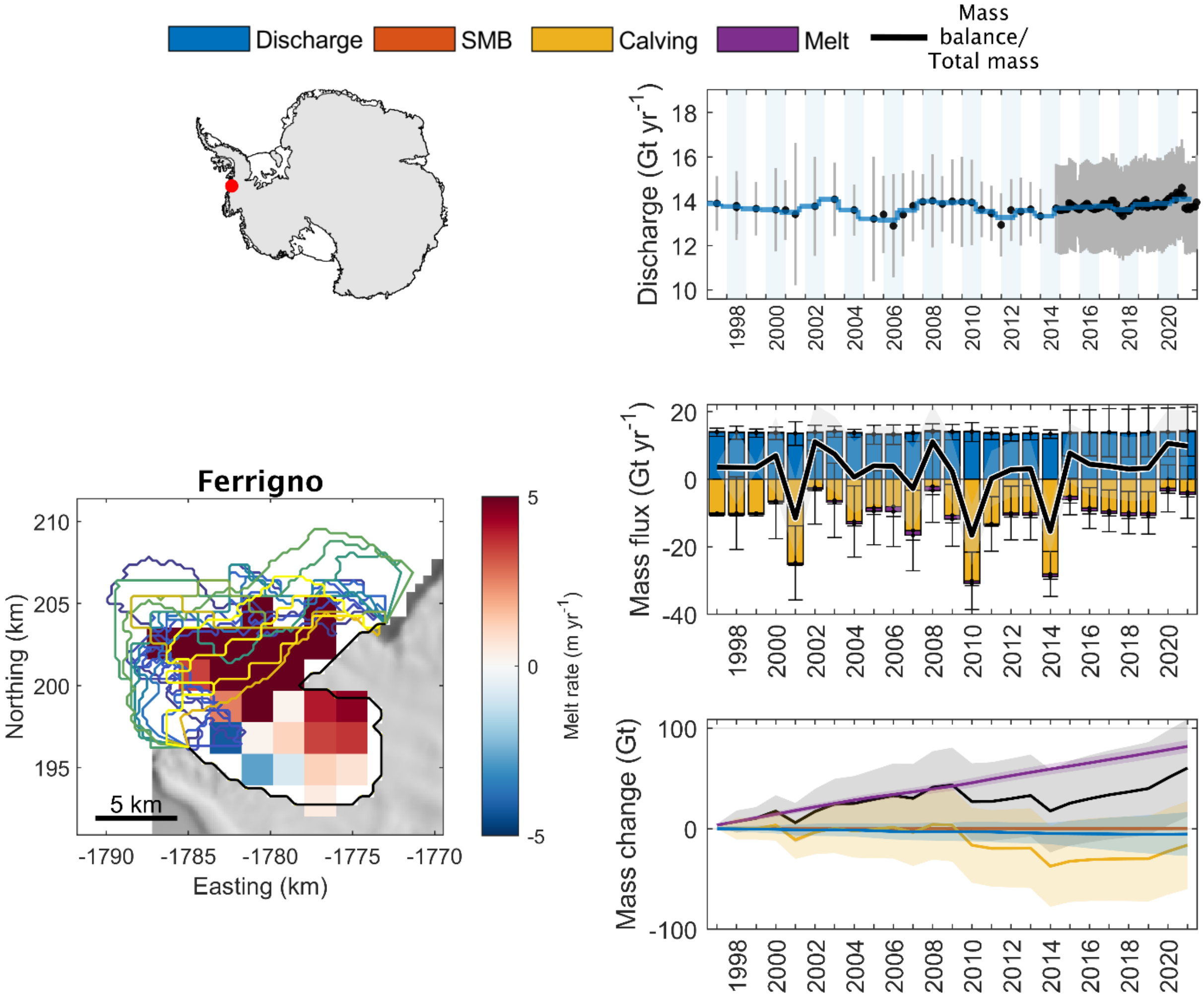

Discharge
SMB
Calving
Melt
Mass balance/ Total mass
Discharge (Gt yr^{-1})
Mass flux (Gt yr^{-1})
Mass change (Gt)
Ferrigno
Northing (km)
Easting (km)
Melt rate (m yr^{-1})
5 km

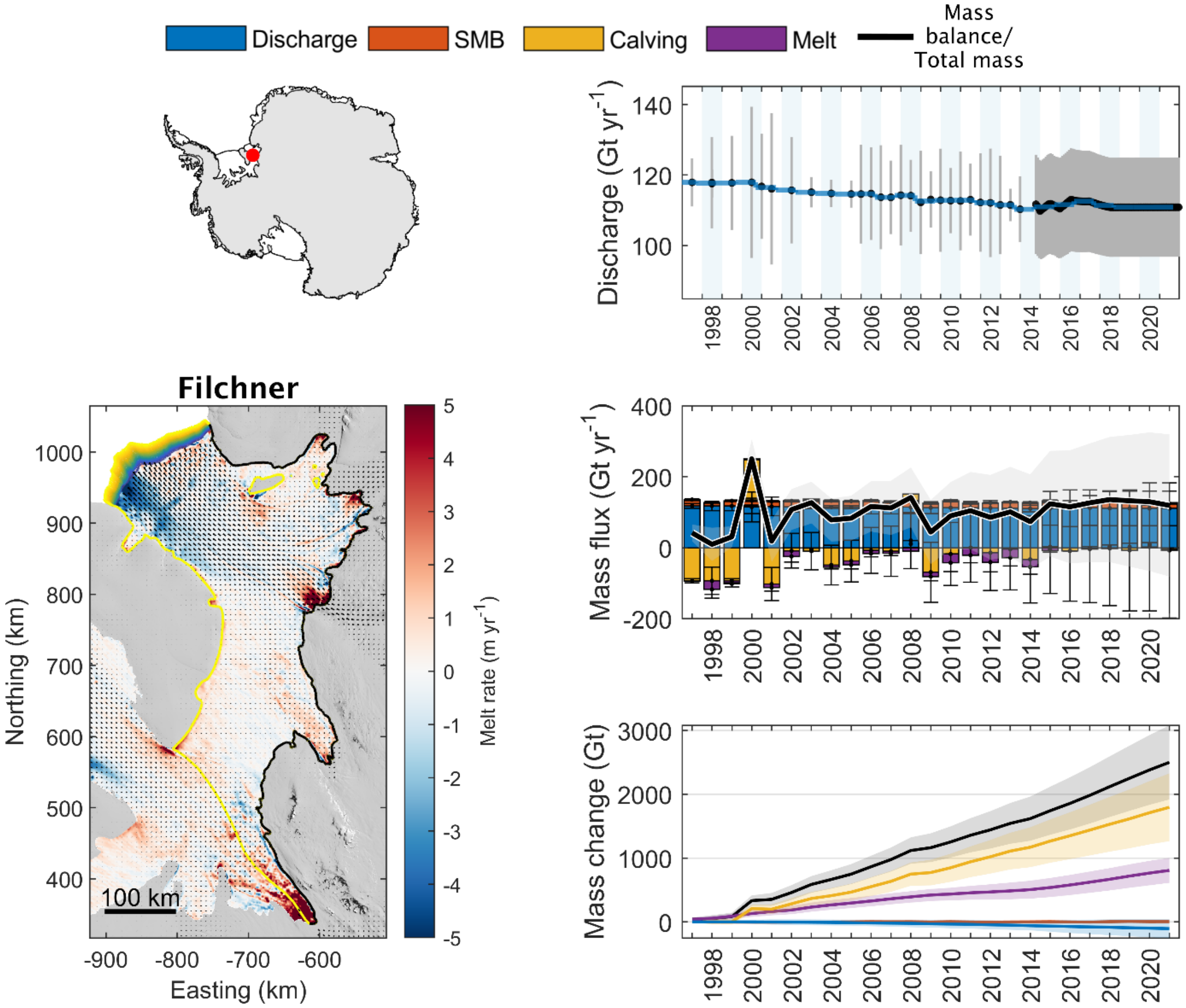

Discharge
SMB
Calving
Melt
Mass balance/ Total mass
Filchner
100 km
Northing (km)
Easting (km)
Melt rate (m yr-1)
Discharge (Gt yr-1)
Mass flux (Gt yr-1)
Mass change (Gt)

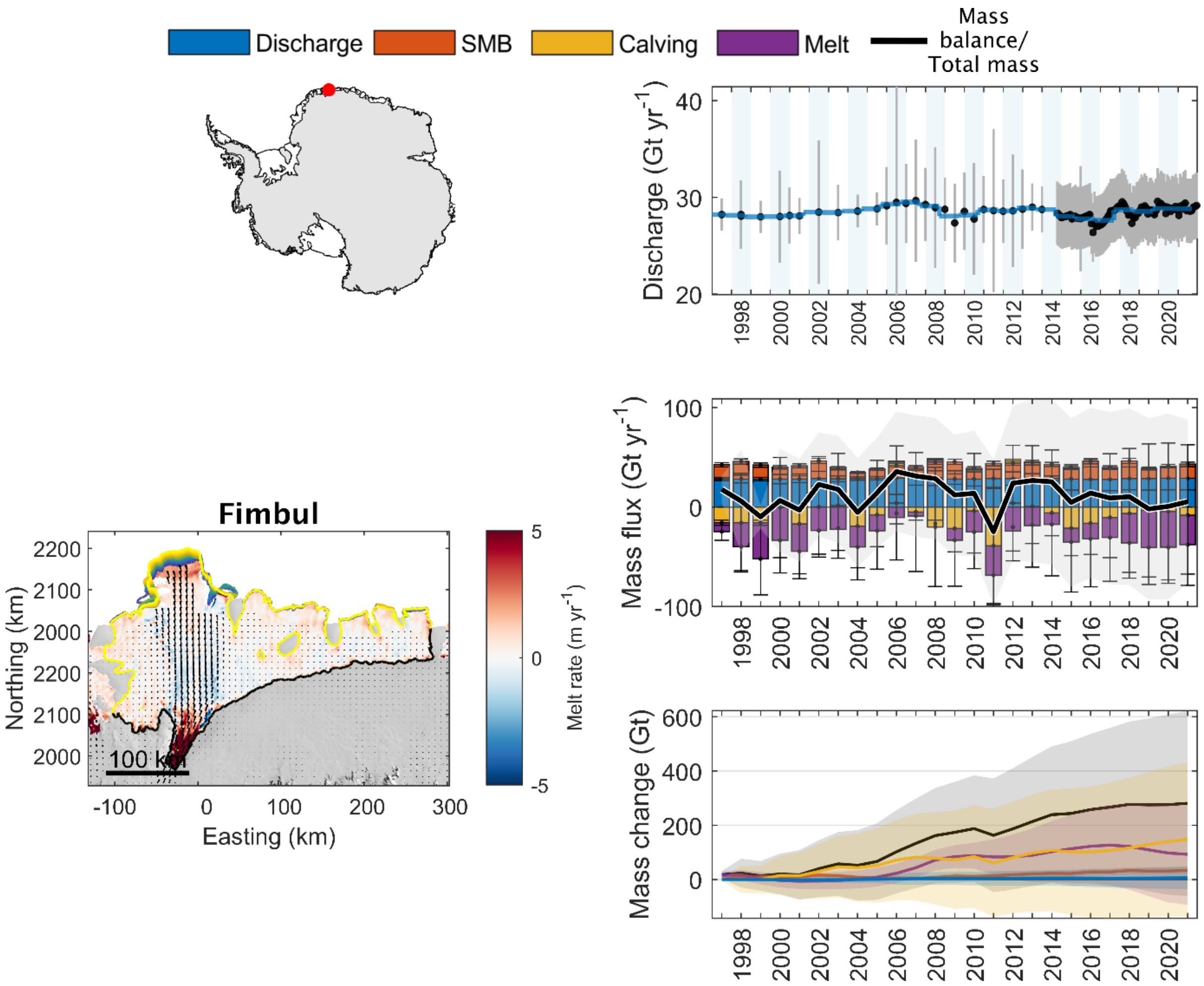

Discharge
SMB
Calving
Melt
Mass balance/ Total mass
Fimbul
Northing (km)
Easting (km)
100 km
Melt rate (m yr-1)
Discharge (Gt yr-1)
Mass flux (Gt yr-1)
Mass change (Gt)

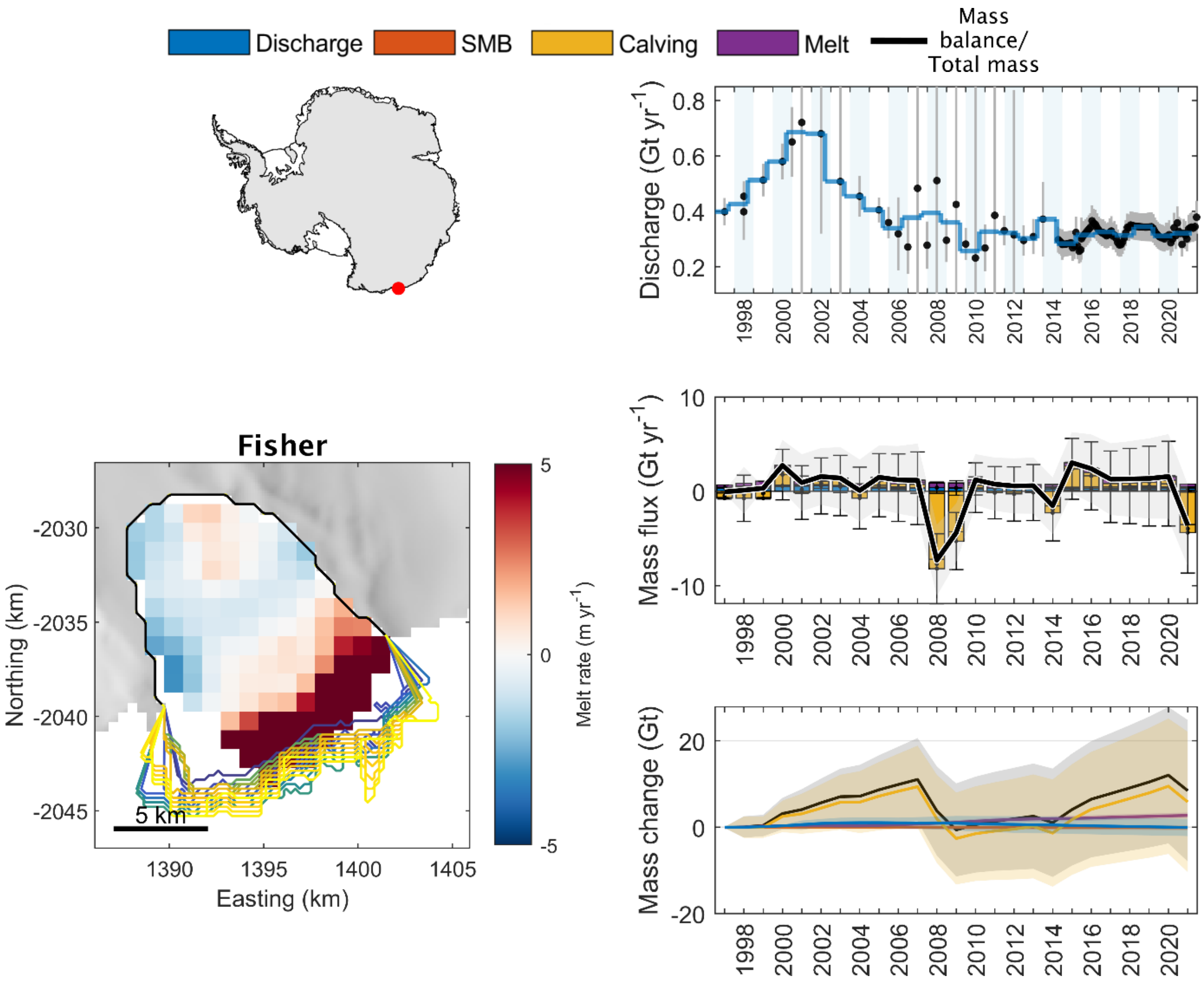

Discharge
SMB
Calving
Melt
Mass balance/ Total mass
Fisher
Northing (km)
Easting (km)
Melt rate (m yr-1)
5 km
Discharge (Gt yr-1)
Mass flux (Gt yr-1)
Mass change (Gt)

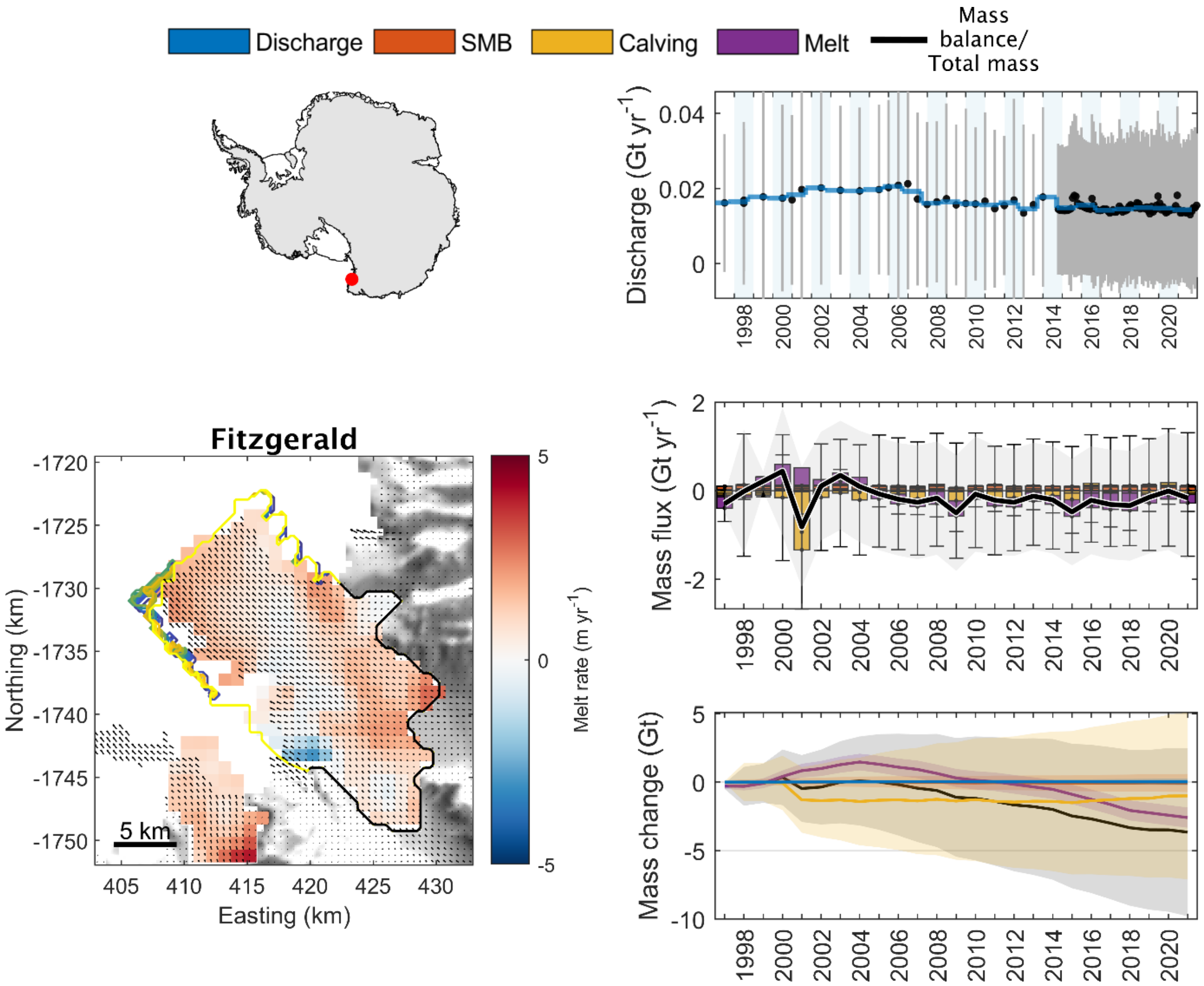

Discharge
SMB
Calving
Melt
Mass balance/ Total mass
Discharge (Gt yr-1)
Fitzgerald
Northing (km)
Easting (km)
5 km
Melt rate (m yr-1)
Mass flux (Gt yr-1)
Mass change (Gt)

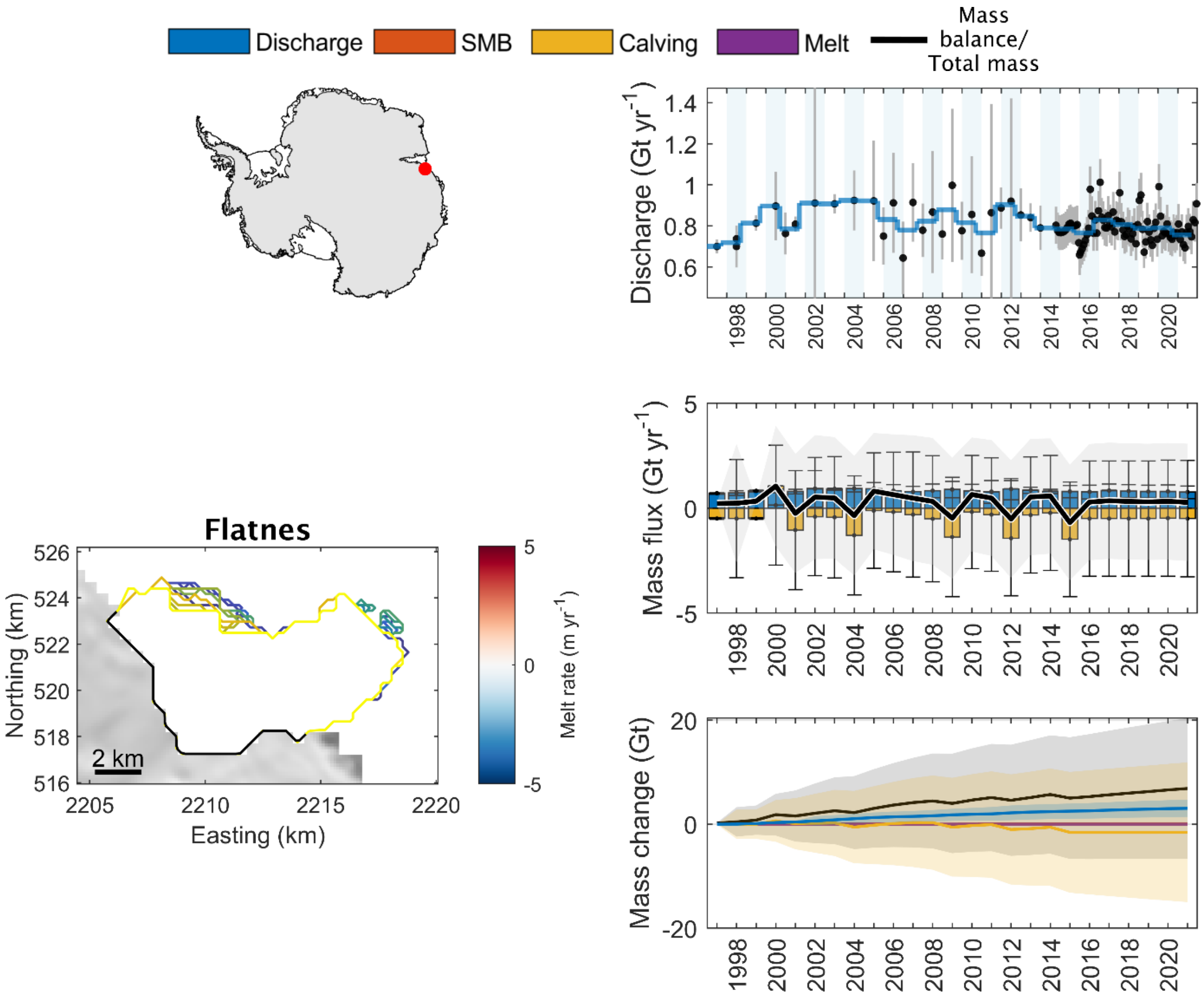

Discharge
SMB
Calving
Melt
Mass balance/ Total mass
Discharge (Gt yr^{-1})
Mass flux (Gt yr^{-1})
Mass change (Gt)
1998
2000
2002
2004
2006
2008
2010
2012
2014
2016
2018
2020
Flatnes
Northing (km)
Easting (km)
2 km
Melt rate (m yr^{-1})

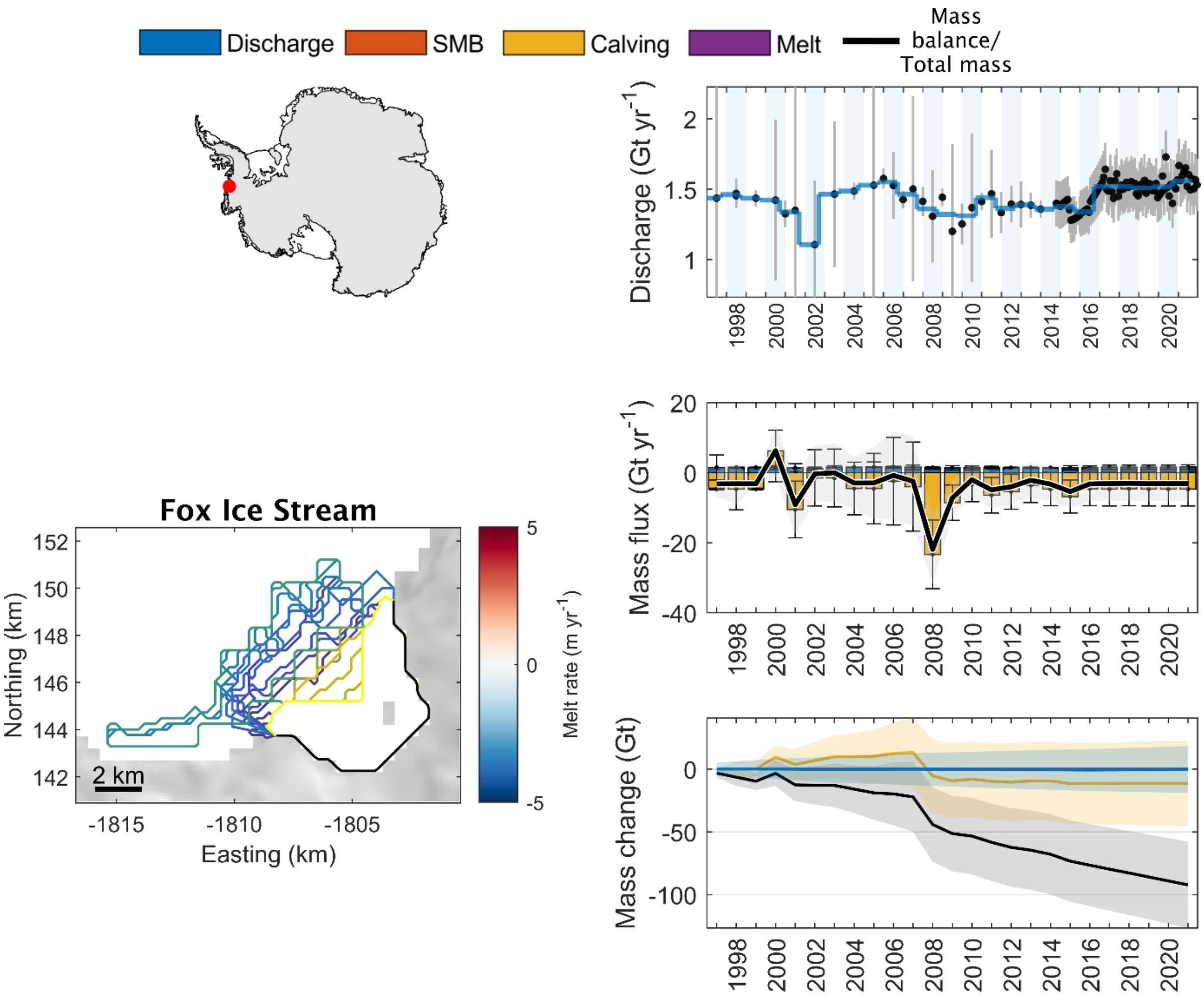

Discharge
SMB
Calving
Melt
Mass balance/ Total mass
Fox Ice Stream
Northing (km)
Easting (km)
2 km
Melt rate (m yr-1)
Discharge (Gt yr-1)
Mass flux (Gt yr-1)
Mass change (Gt)

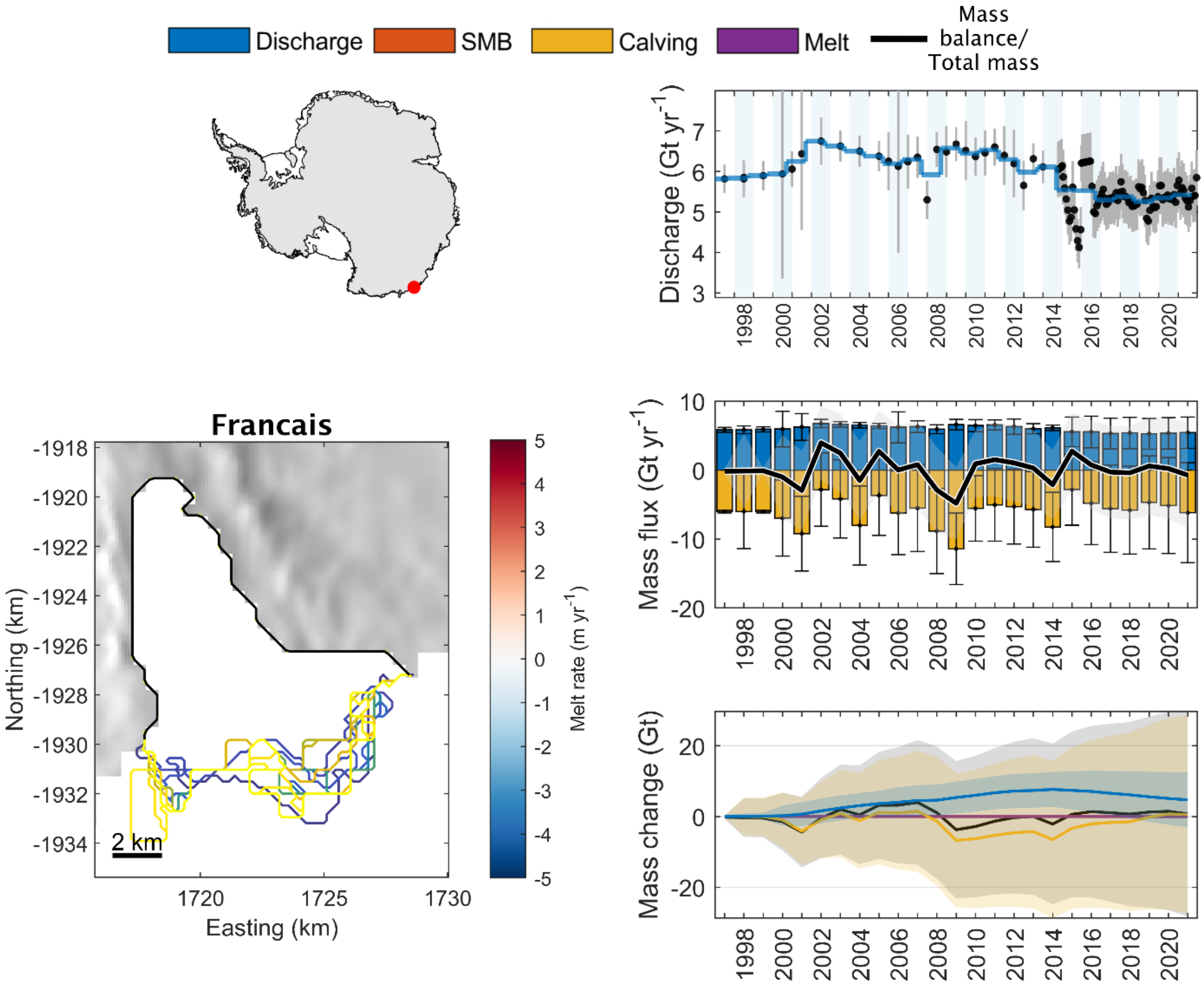

Discharge
SMB
Calving
Melt
Mass balance/ Total mass
Francais
Northing (km)
Easting (km)
2 km
Melt rate (m yr^{-1})
Discharge (Gt yr^{-1})
Mass flux (Gt yr^{-1})
Mass change (Gt)

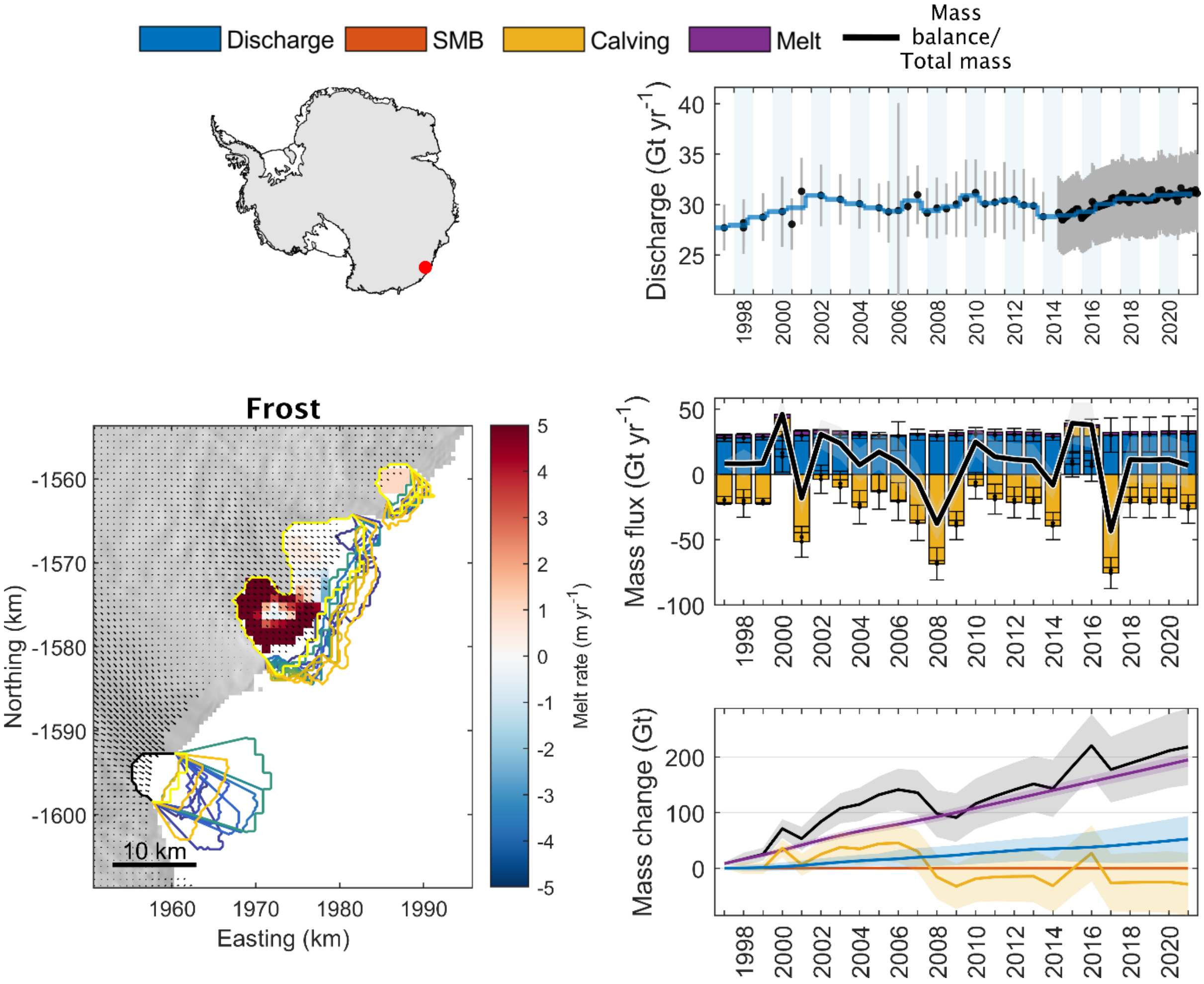
Discharge
SMB
Calving
Melt
Mass balance/ Total mass
Discharge (Gt yr^{-1})
Frost
Northing (km)
Easting (km)
10 km
Melt rate (m yr^{-1})
Mass flux (Gt yr^{-1})
Mass change (Gt)

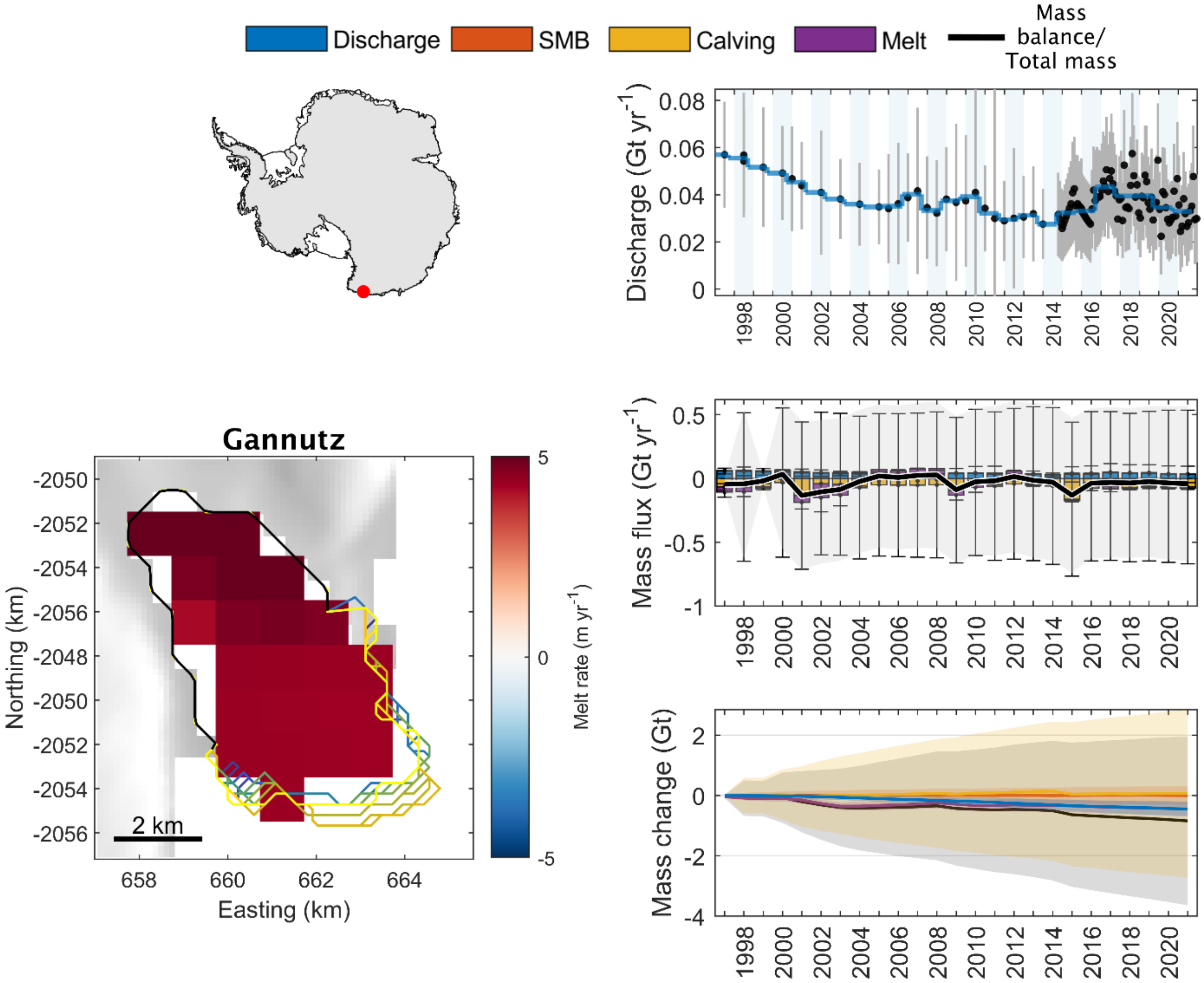
Discharge
SMB
Calving
Melt
Mass balance/ Total mass
Gannutz
Northing (km)
Easting (km)
2 km
Melt rate (m yr-1)
Discharge (Gt yr-1)
Mass flux (Gt yr-1)
Mass change (Gt)

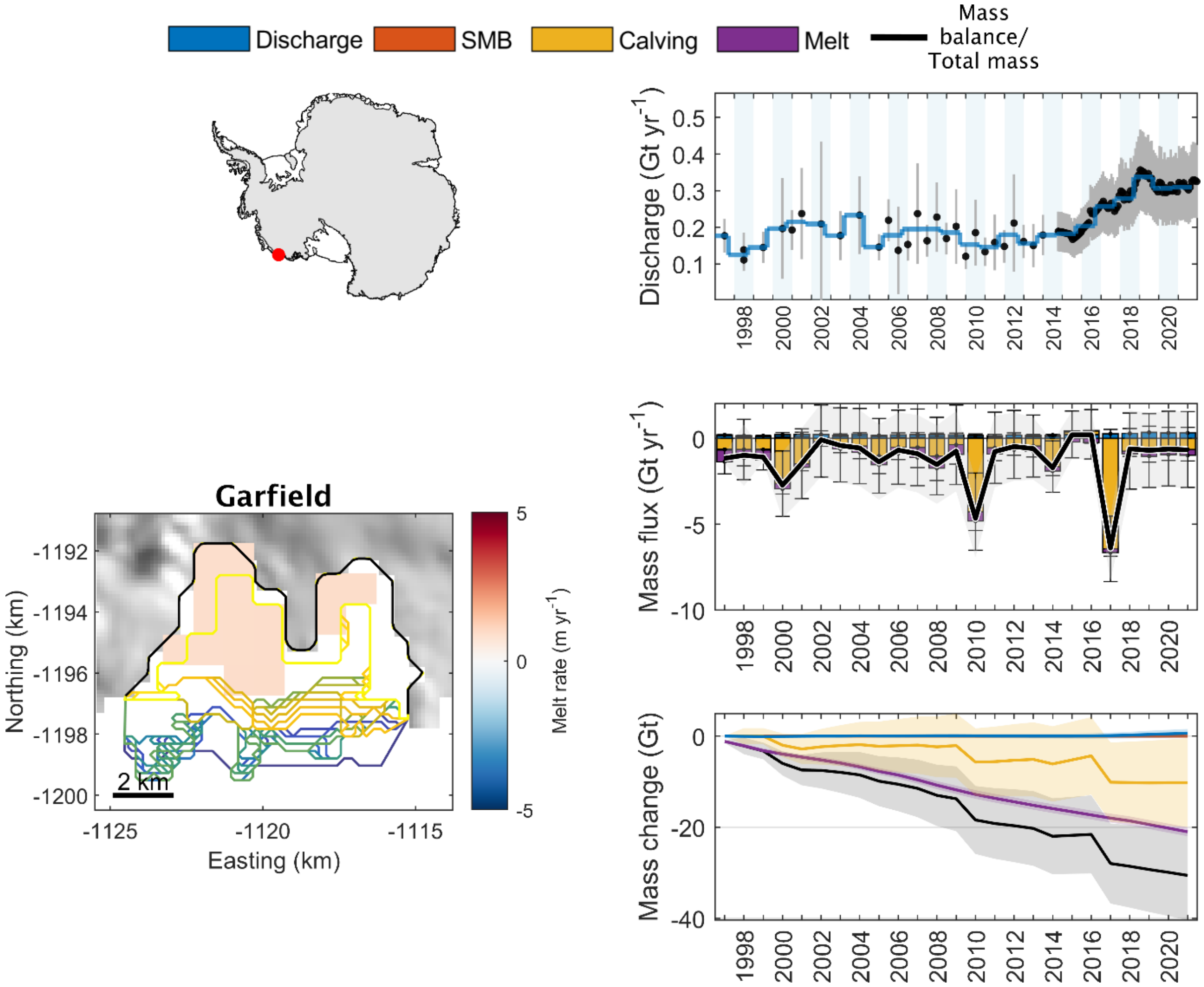
Discharge
SMB
Calving
Melt
Mass balance/ Total mass
Garfield
Northing (km)
Easting (km)
2 km
Melt rate (m yr⁻¹)
Discharge (Gt yr⁻¹)
Mass flux (Gt yr⁻¹)
Mass change (Gt)

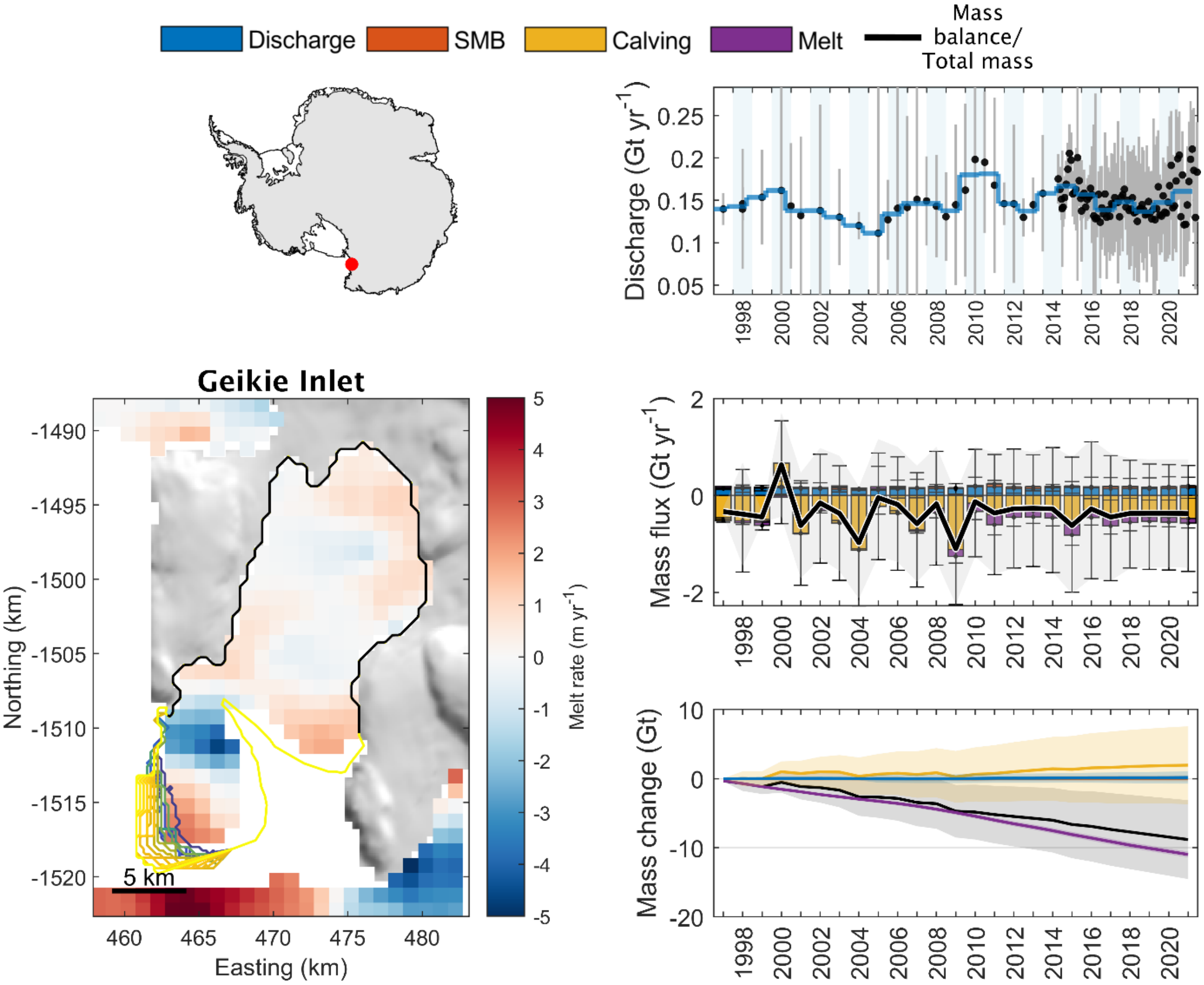

Discharge
SMB
Calving
Melt
Mass balance/ Total mass
Discharge (Gt yr-1)
Geikie Inlet
Northing (km)
Easting (km)
5 km
Melt rate (m yr-1)
Mass flux (Gt yr-1)
Mass change (Gt)

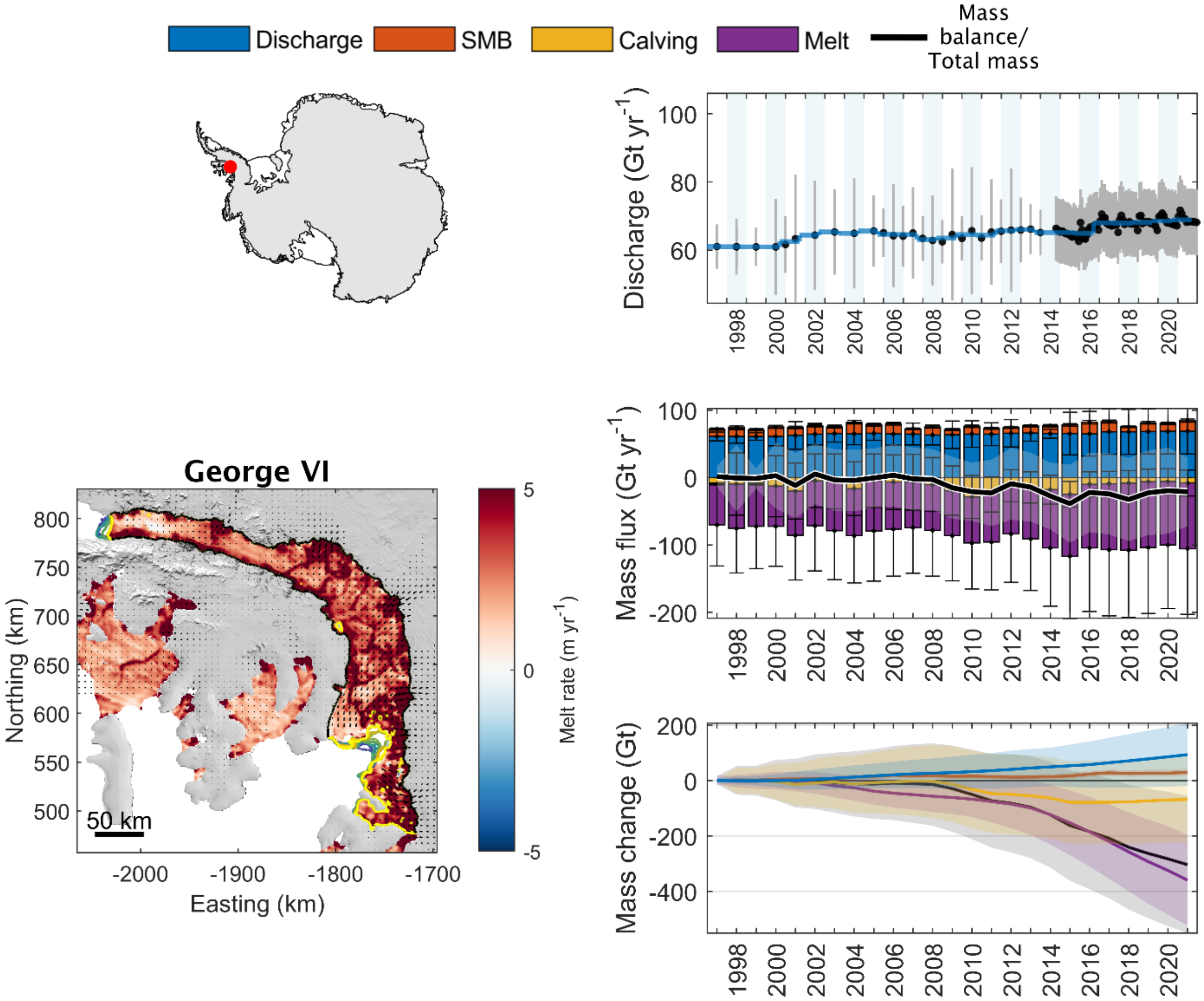

Discharge
SMB
Calving
Melt
Mass balance/ Total mass
George VI
Northing (km)
Easting (km)
50 km
Melt rate (m yr^{-1})
Discharge (Gt yr^{-1})
Mass flux (Gt yr^{-1})
Mass change (Gt)

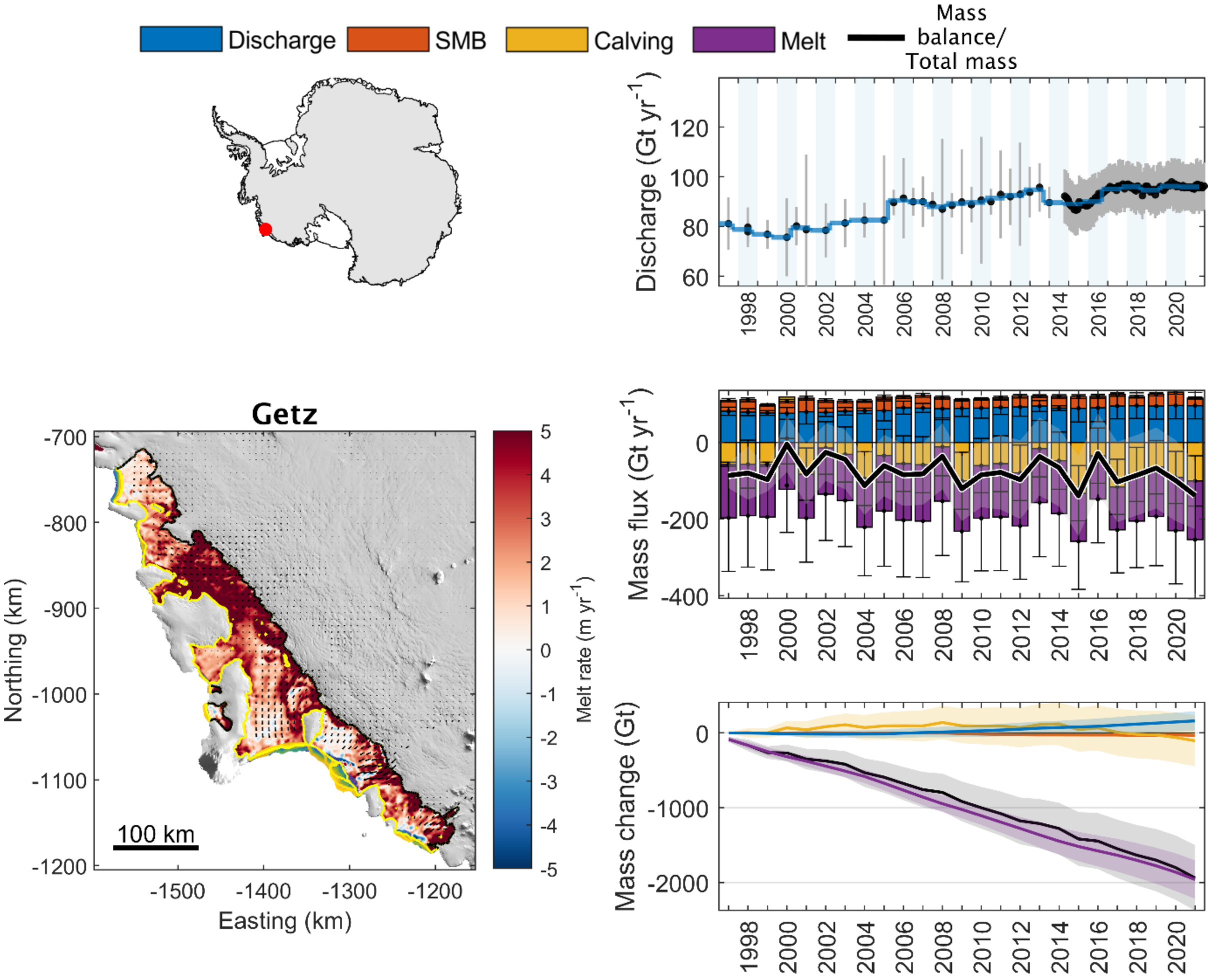

Discharge
SMB
Calving
Melt
Mass balance/ Total mass
Getz
Northing (km)
Easting (km)
100 km
Melt rate (m yr^{-1})
Discharge (Gt yr^{-1})
Mass flux (Gt yr^{-1})
Mass change (Gt)

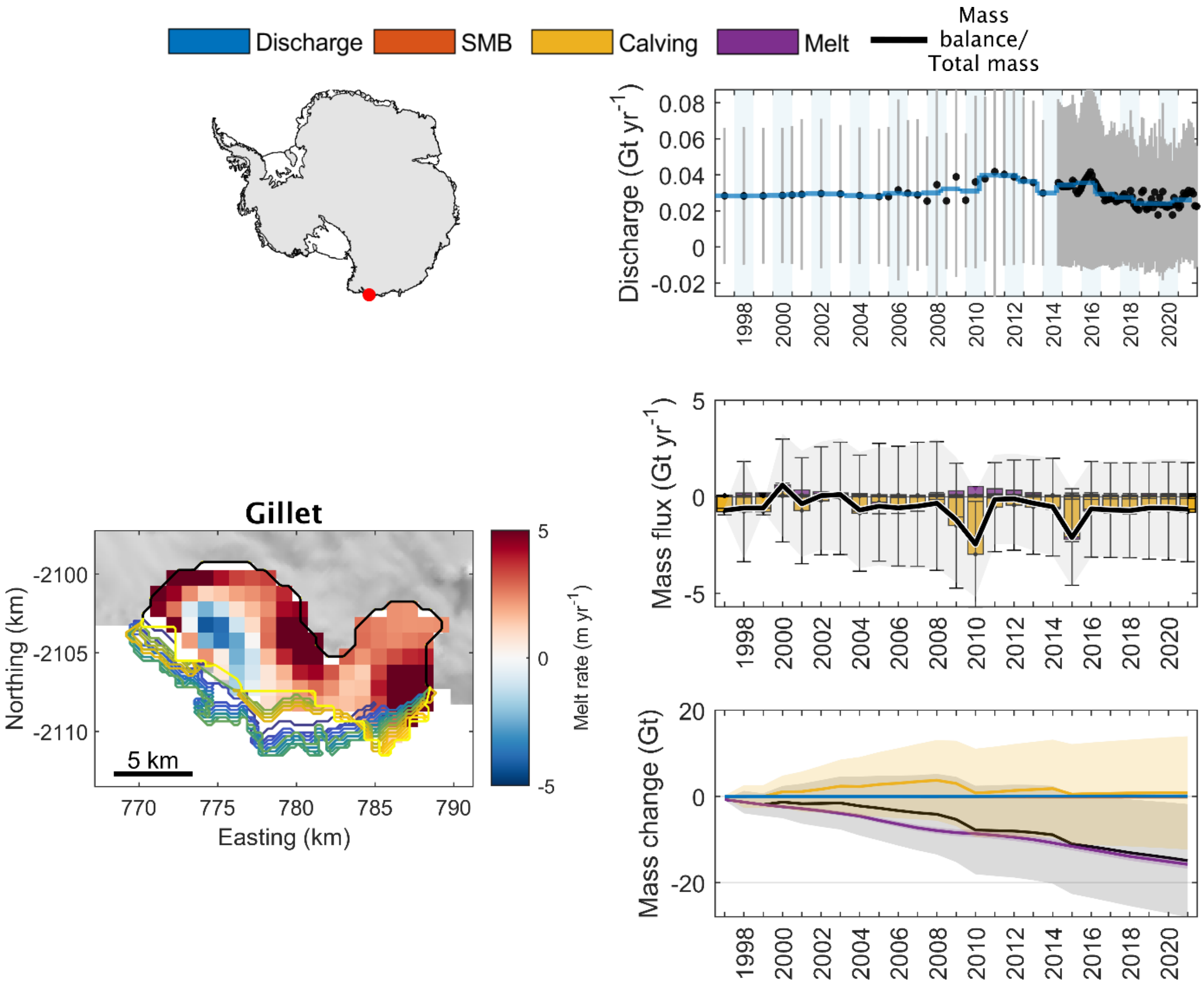
Discharge
SMB
Calving
Melt
Mass balance/ Total mass
Gillet
Northing (km)
Easting (km)
5 km
Melt rate (m yr^{-1})
Discharge (Gt yr^{-1})
Mass flux (Gt yr^{-1})
Mass change (Gt)

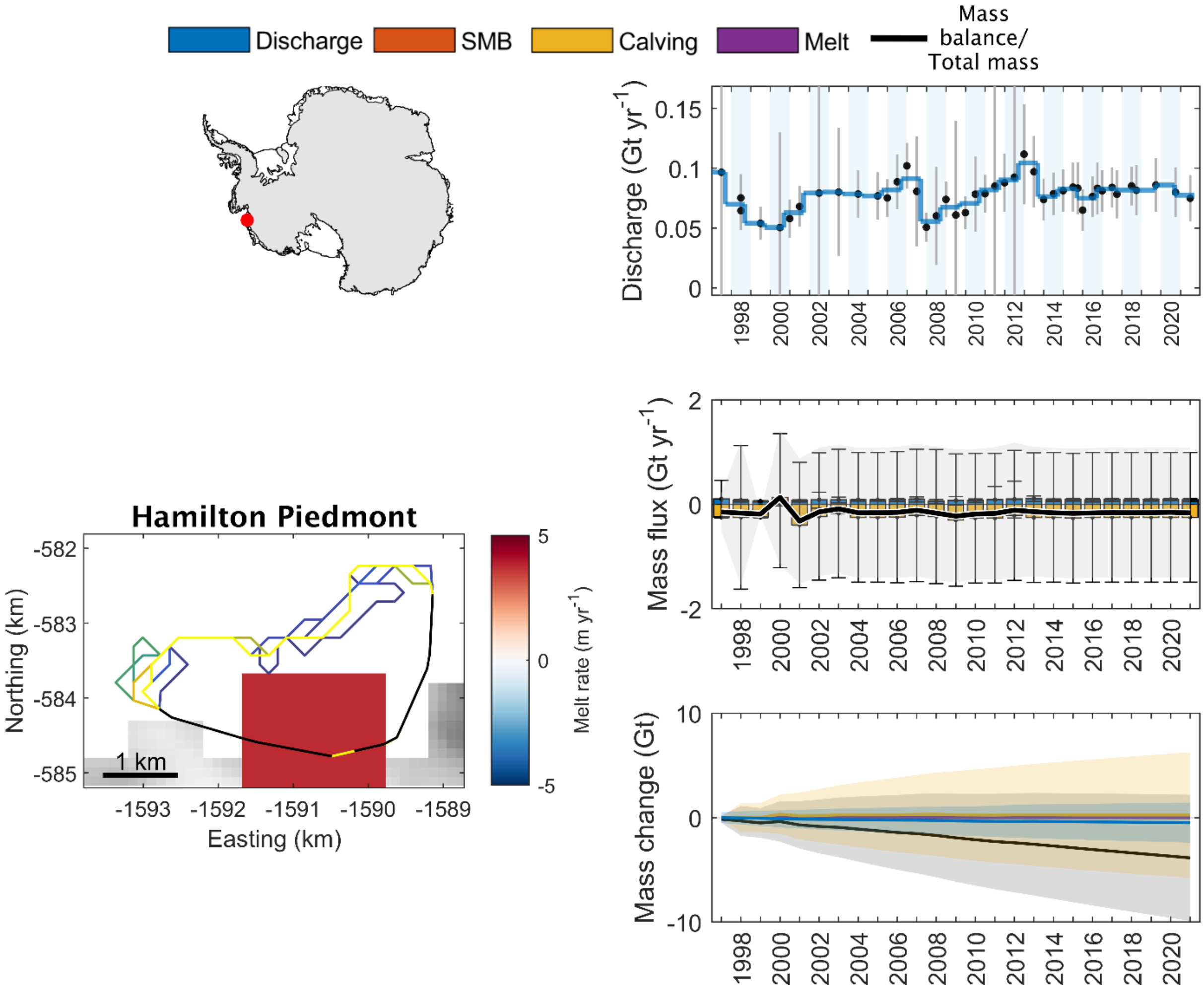
Discharge
SMB
Calving
Melt
Mass balance/ Total mass
Hamilton Piedmont
Northing (km)
Easting (km)
1 km
Melt rate (m yr^{-1})
Discharge (Gt yr^{-1})
Mass flux (Gt yr^{-1})
Mass change (Gt)

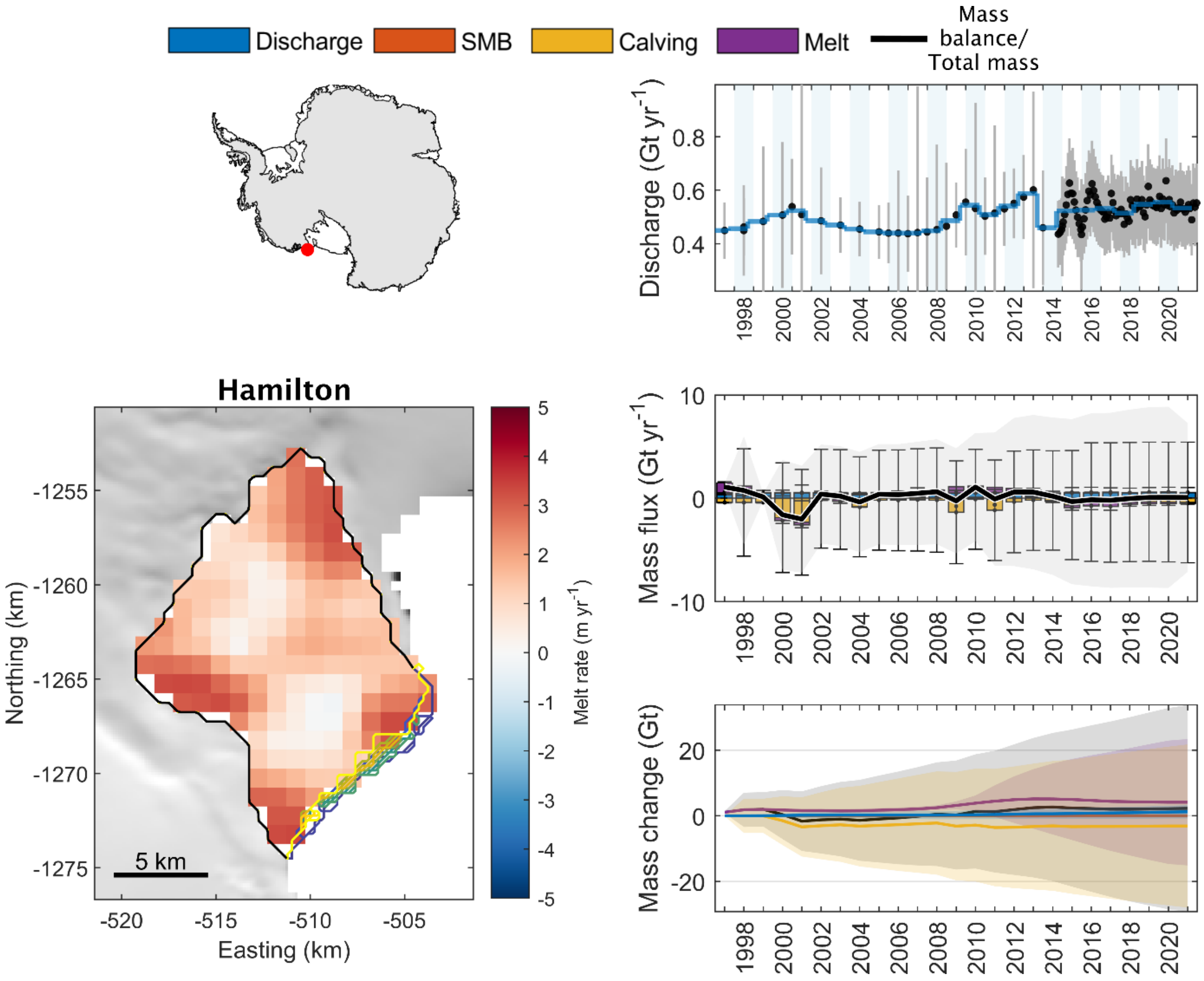
Discharge
SMB
Calving
Melt
Mass balance/ Total mass
Hamilton
Northing (km)
Easting (km)
5 km
Melt rate (m yr^{-1})
Discharge (Gt yr^{-1})
Mass flux (Gt yr^{-1})
Mass change (Gt)

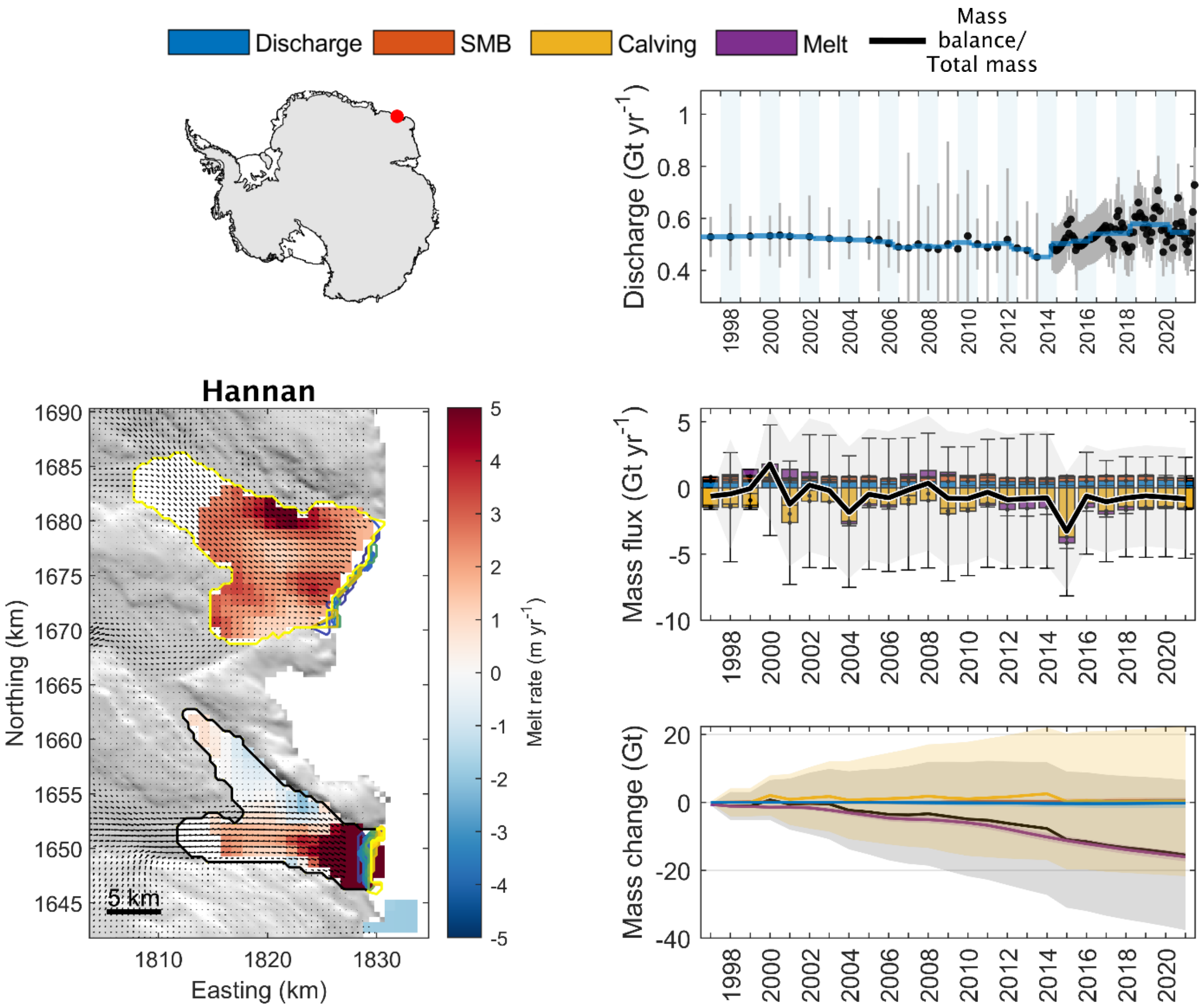

Discharge
SMB
Calving
Melt
Mass balance/ Total mass
Hannan
Northing (km)
Easting (km)
5 km
Melt rate (m yr^{-1})
Discharge (Gt yr^{-1})
Mass flux (Gt yr^{-1})
Mass change (Gt)

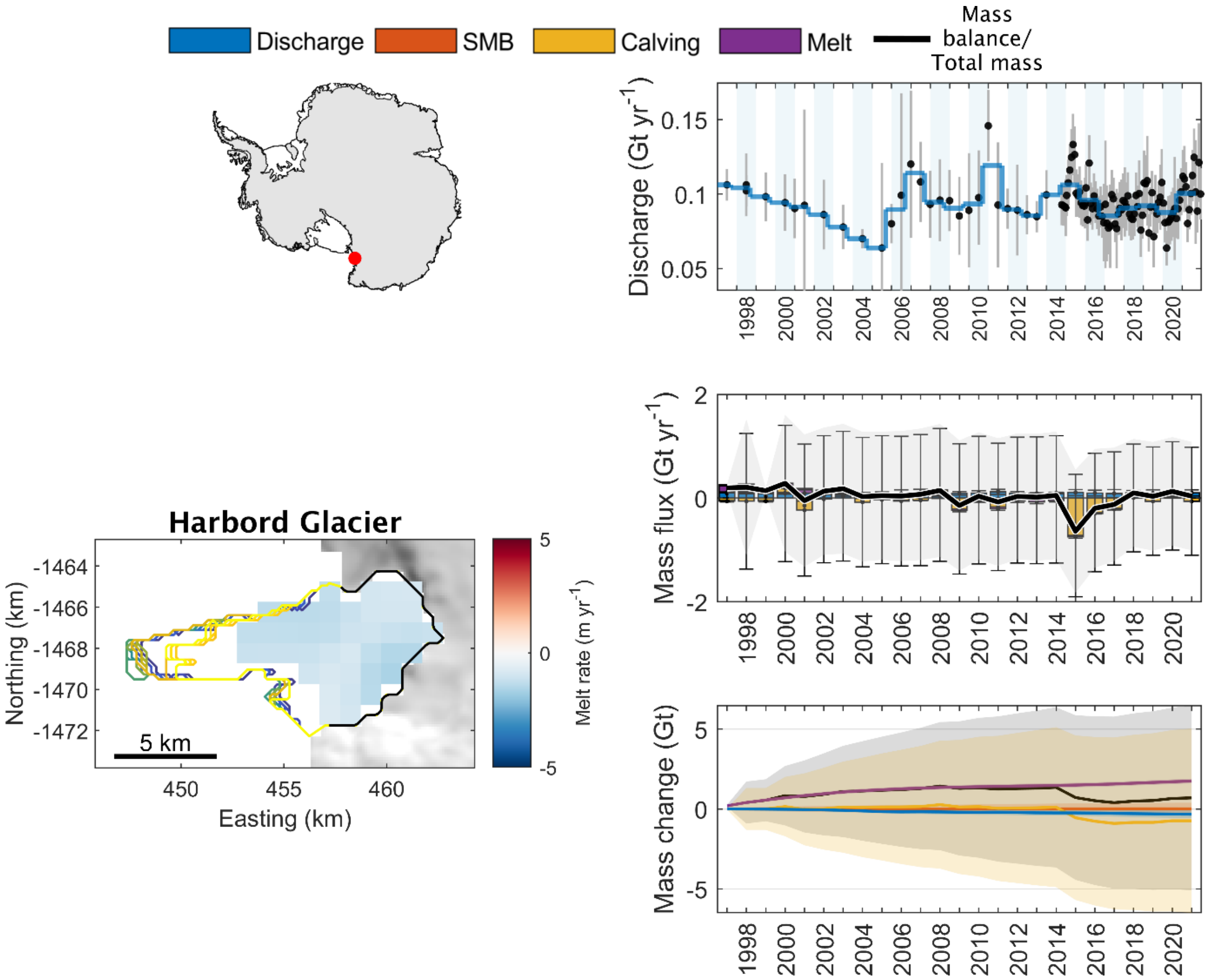
Discharge
SMB
Calving
Melt
Mass balance/ Total mass
Harbord Glacier
Northing (km)
Easting (km)
5 km
Melt rate (m yr-1)
Discharge (Gt yr-1)
Mass flux (Gt yr-1)
Mass change (Gt)

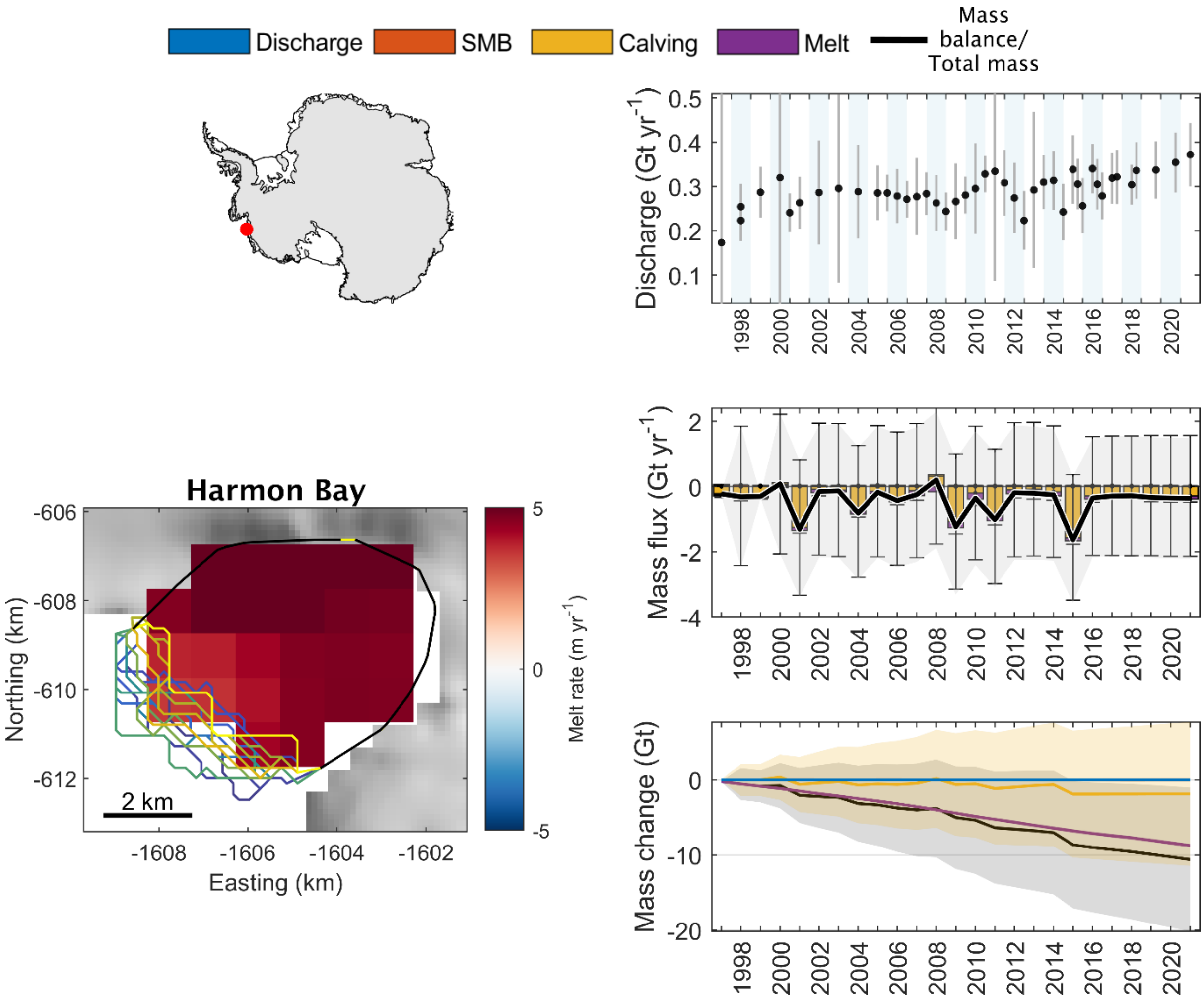

Discharge
SMB
Calving
Melt
Mass balance/ Total mass
Harmon Bay
Northing (km)
Easting (km)
2 km
Melt rate (m yr^{-1})
Discharge (Gt yr^{-1})
Mass flux (Gt yr^{-1})
Mass change (Gt)

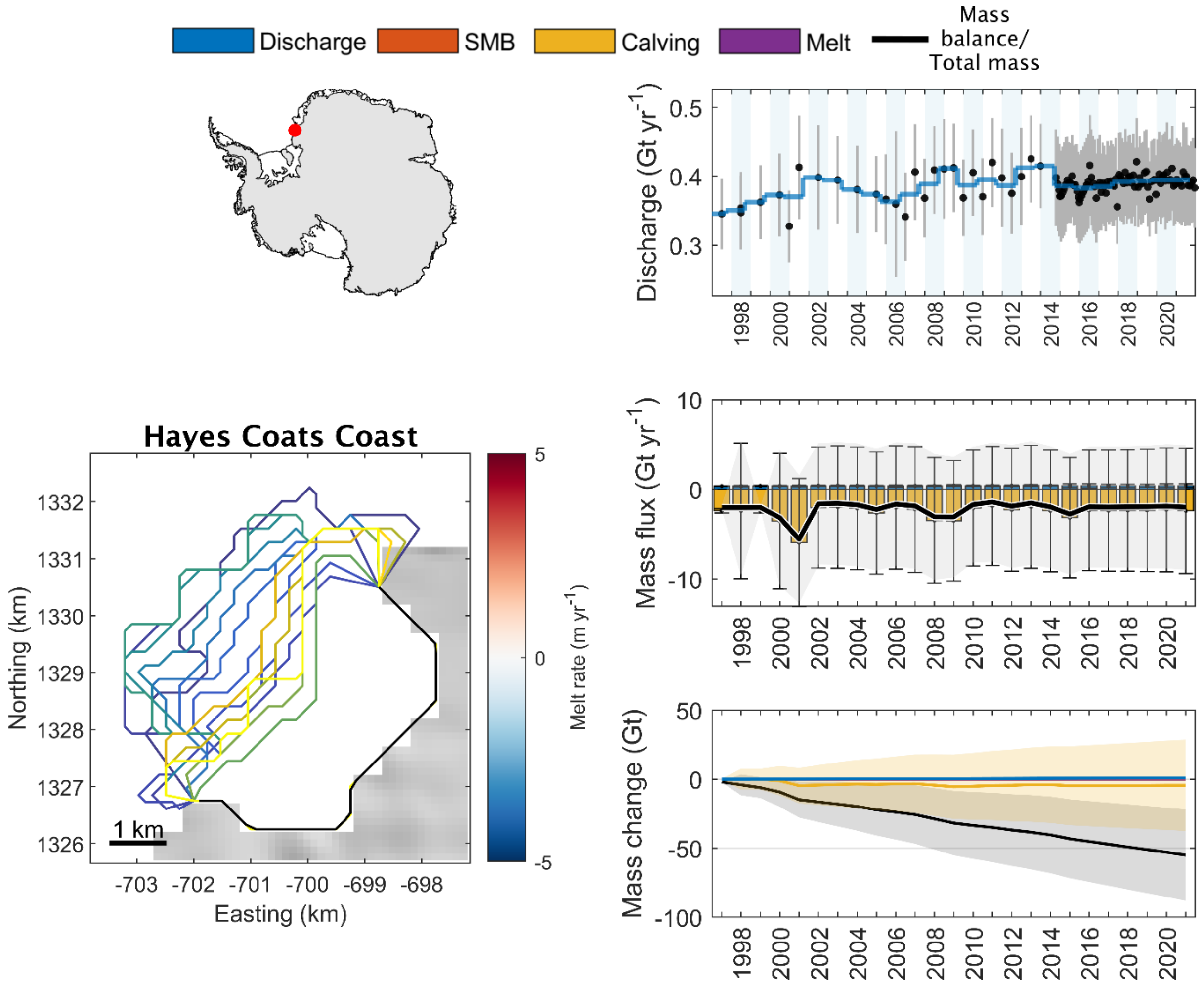

Discharge
SMB
Calving
Melt
Mass balance/ Total mass
Discharge (Gt yr^-1)
Hayes Coats Coast
Northing (km)
Easting (km)
1 km
Melt rate (m yr^-1)
Mass flux (Gt yr^-1)
Mass change (Gt)

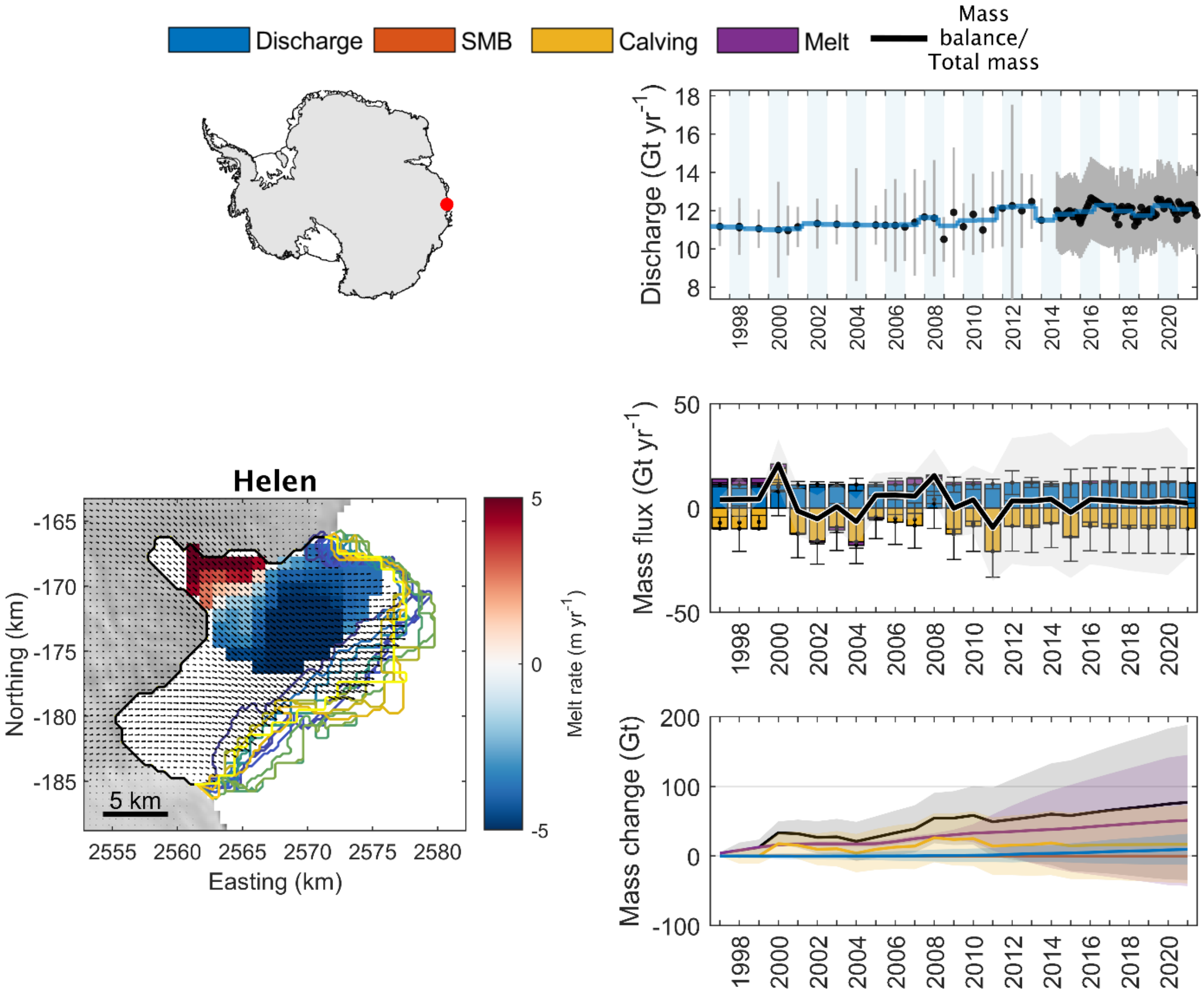
Discharge
SMB
Calving
Melt
Mass balance/ Total mass
Discharge (Gt yr^{-1})
Helen
Northing (km)
Easting (km)
5 km
Melt rate (m yr^{-1})
Mass flux (Gt yr^{-1})
Mass change (Gt)

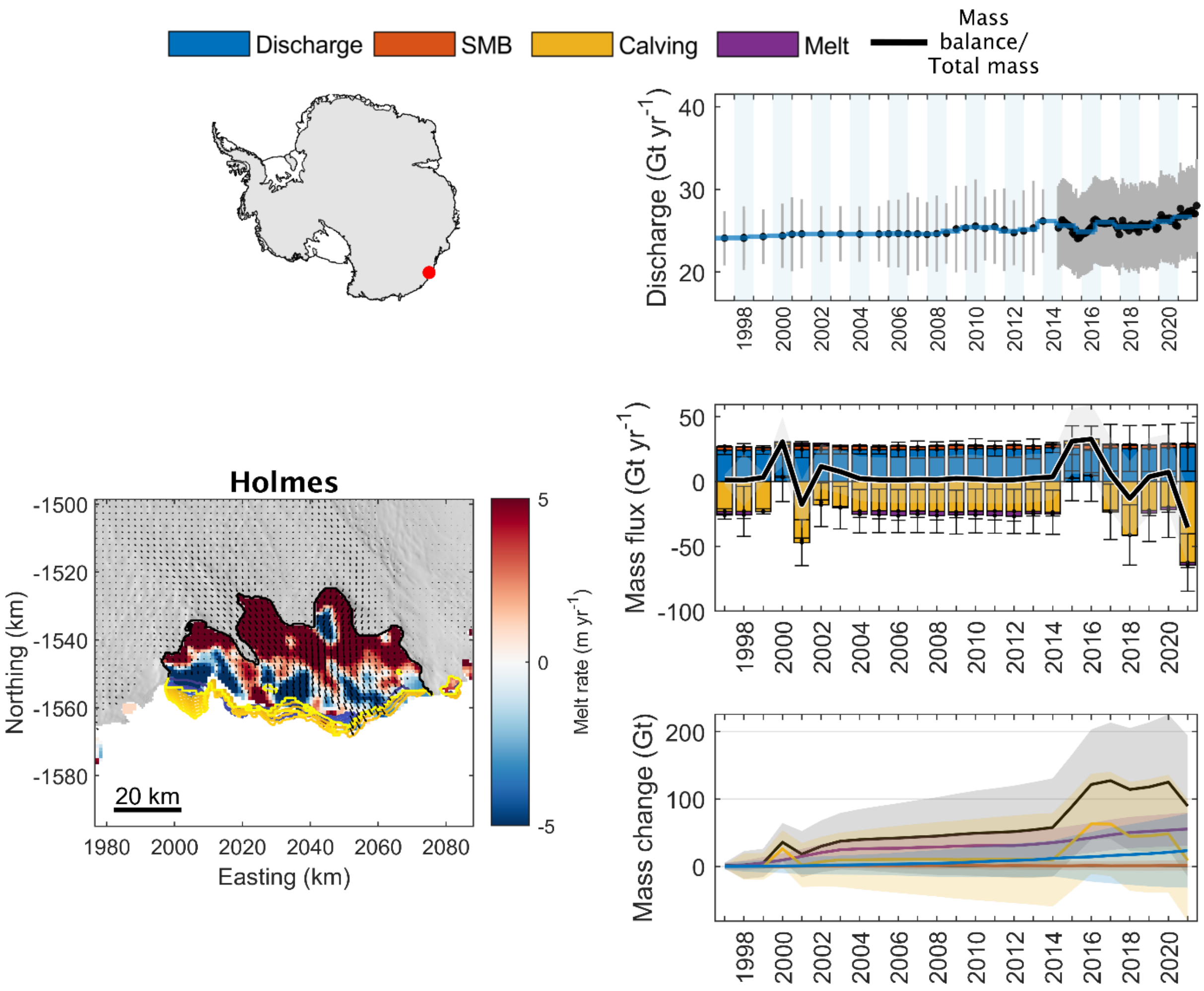
Discharge
SMB
Calving
Melt
Mass balance/ Total mass
Holmes
Northing (km)
Easting (km)
20 km
Melt rate (m yr^{-1})
Discharge (Gt yr^{-1})
Mass flux (Gt yr^{-1})
Mass change (Gt)

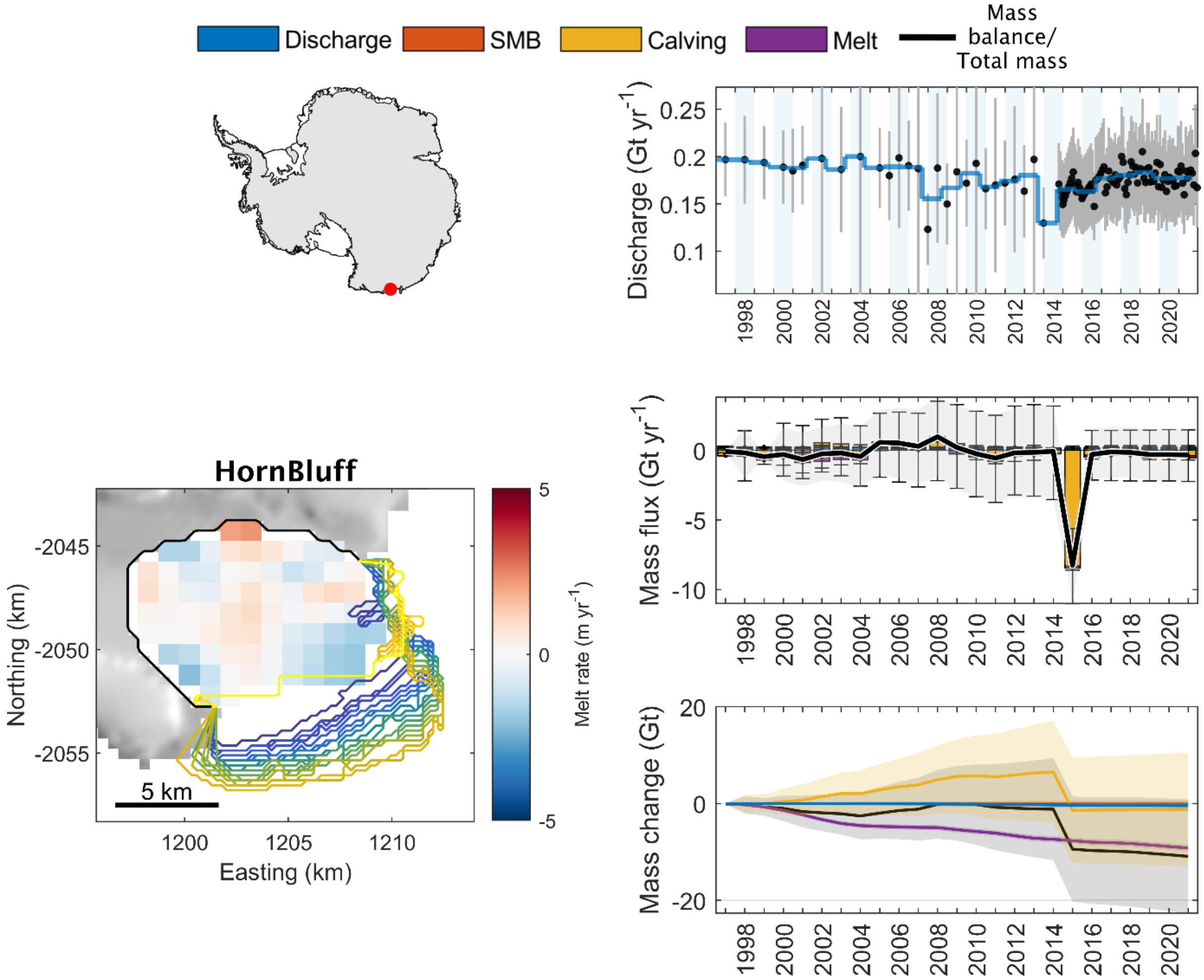

Discharge
SMB
Calving
Melt
Mass balance/ Total mass
HornBluff
Northing (km)
Easting (km)
Melt rate (m yr-1)
5 km
Discharge (Gt yr-1)
Mass flux (Gt yr-1)
Mass change (Gt)

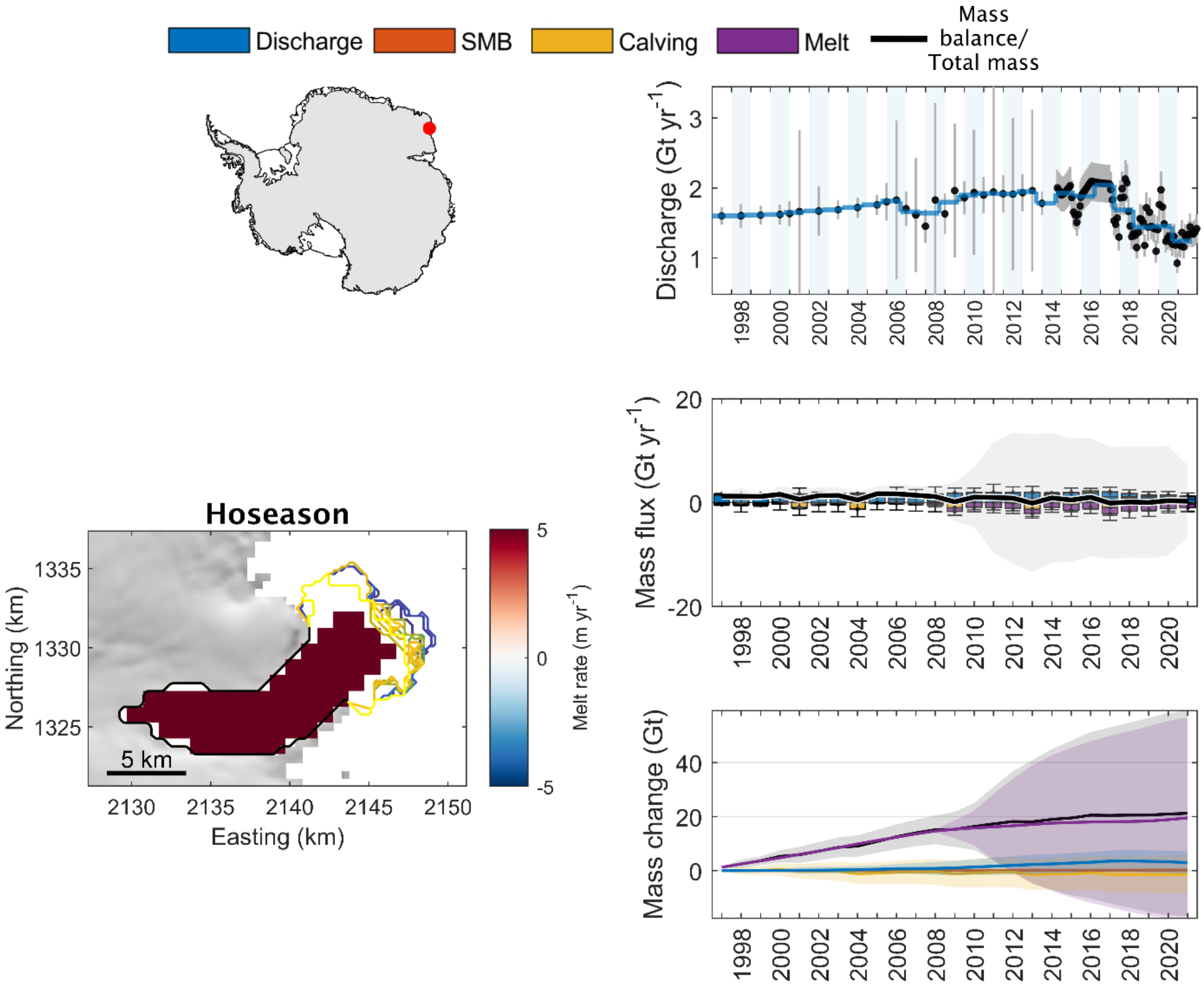
Discharge
SMB
Calving
Melt
Mass balance/ Total mass
Discharge (Gt yr^{-1})
Hoseason
Northing (km)
Easting (km)
5 km
Melt rate (m yr^{-1})
Mass flux (Gt yr^{-1})
Mass change (Gt)

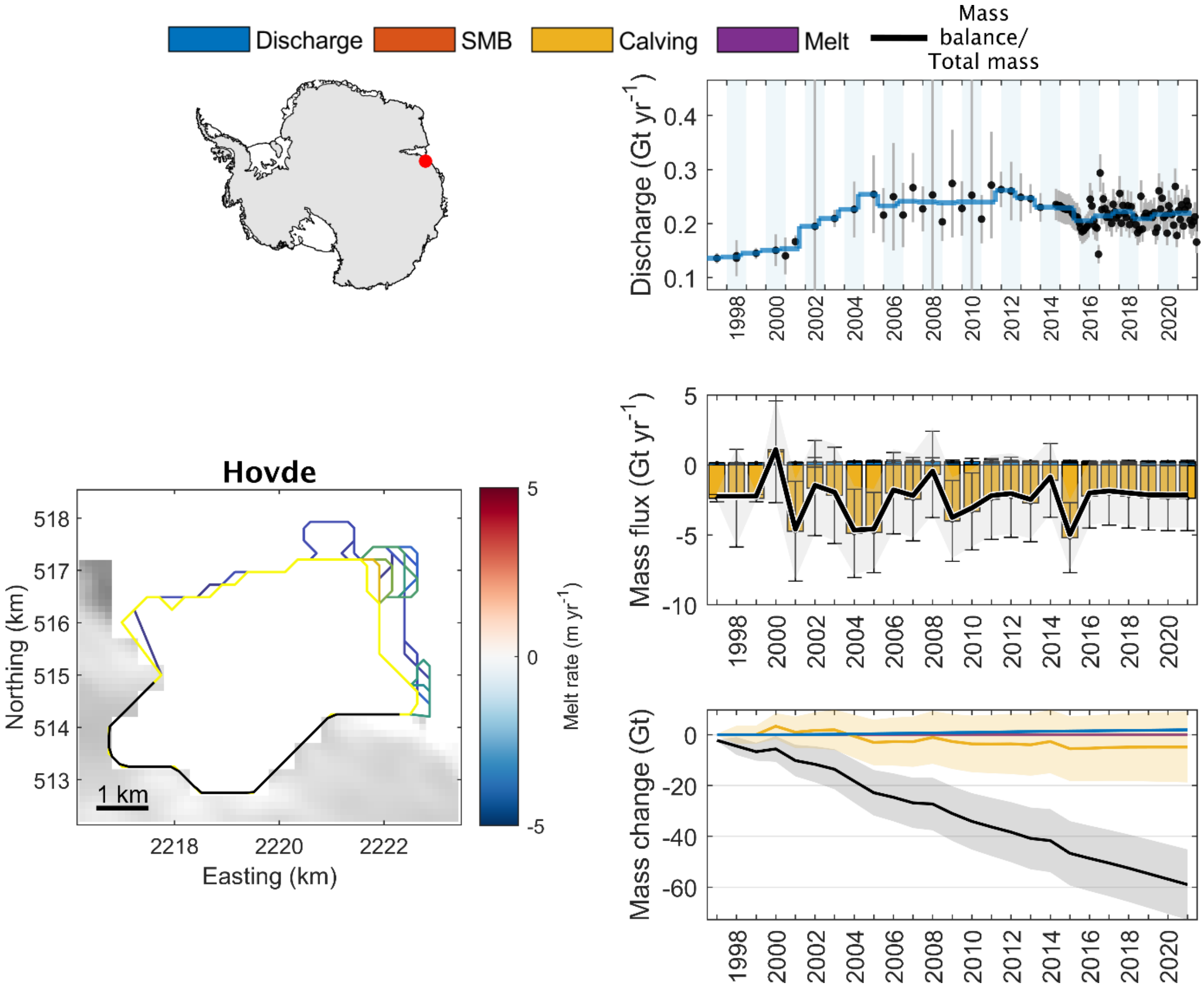
Discharge
SMB
Calving
Melt
Mass balance/ Total mass
Hovde
Northing (km)
Easting (km)
1 km
Melt rate (m yr-1)
Discharge (Gt yr-1)
Mass flux (Gt yr-1)
Mass change (Gt)

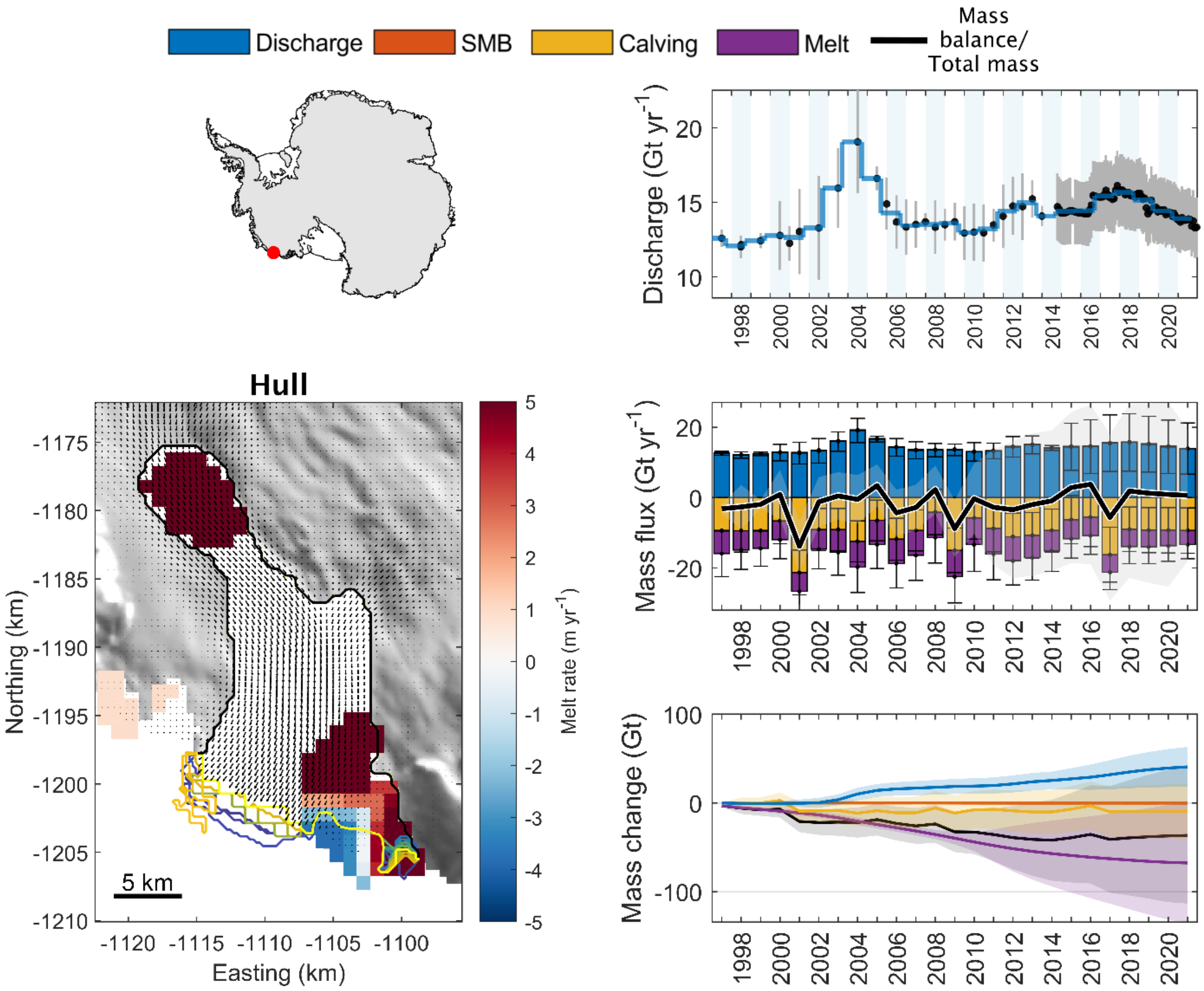

Discharge
SMB
Calving
Melt
Mass balance/ Total mass
Hull
Discharge (Gt yr-1)
Mass flux (Gt yr-1)
Mass change (Gt)
Northing (km)
Easting (km)
Melt rate (m yr-1)
5 km

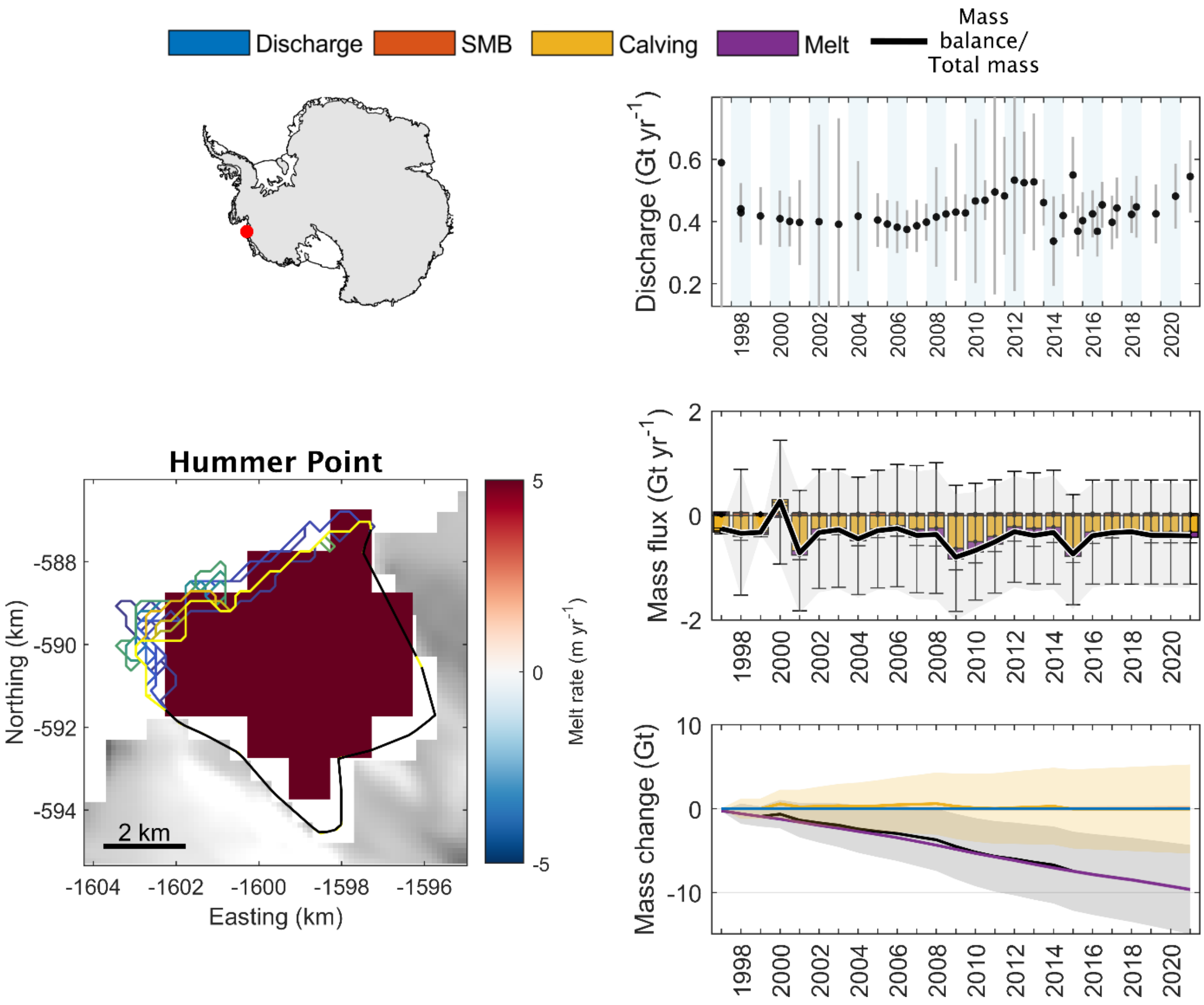

Discharge
SMB
Calving
Melt
Mass balance/ Total mass
Hummer Point
Northing (km)
Easting (km)
2 km
Melt rate (m yr^{-1})
Discharge (Gt yr^{-1})
Mass flux (Gt yr^{-1})
Mass change (Gt)

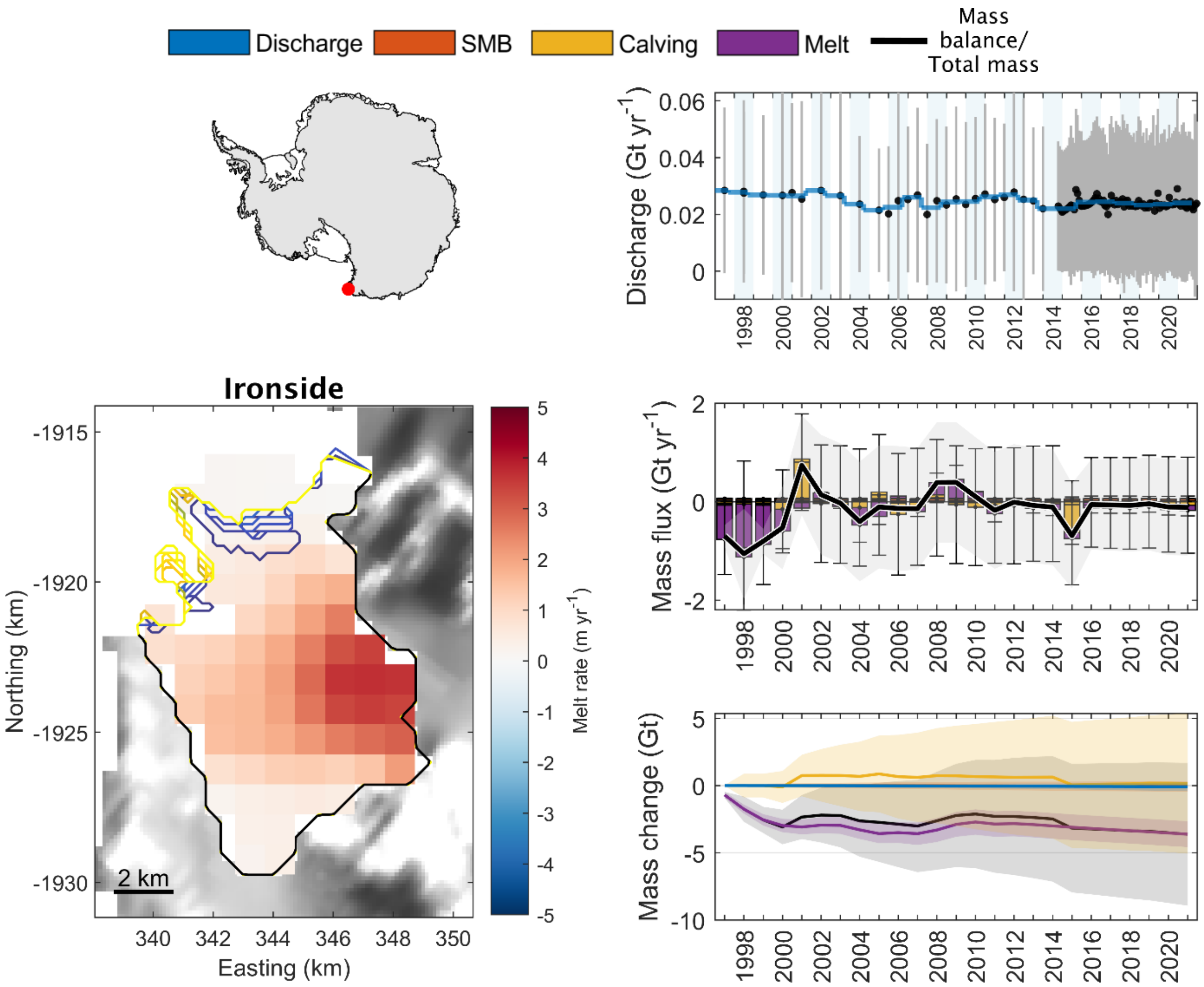
Discharge
SMB
Calving
Melt
Mass balance/ Total mass
Ironside
Northing (km)
Easting (km)
2 km
Melt rate (m yr-1)
Discharge (Gt yr-1)
Mass flux (Gt yr-1)
Mass change (Gt)

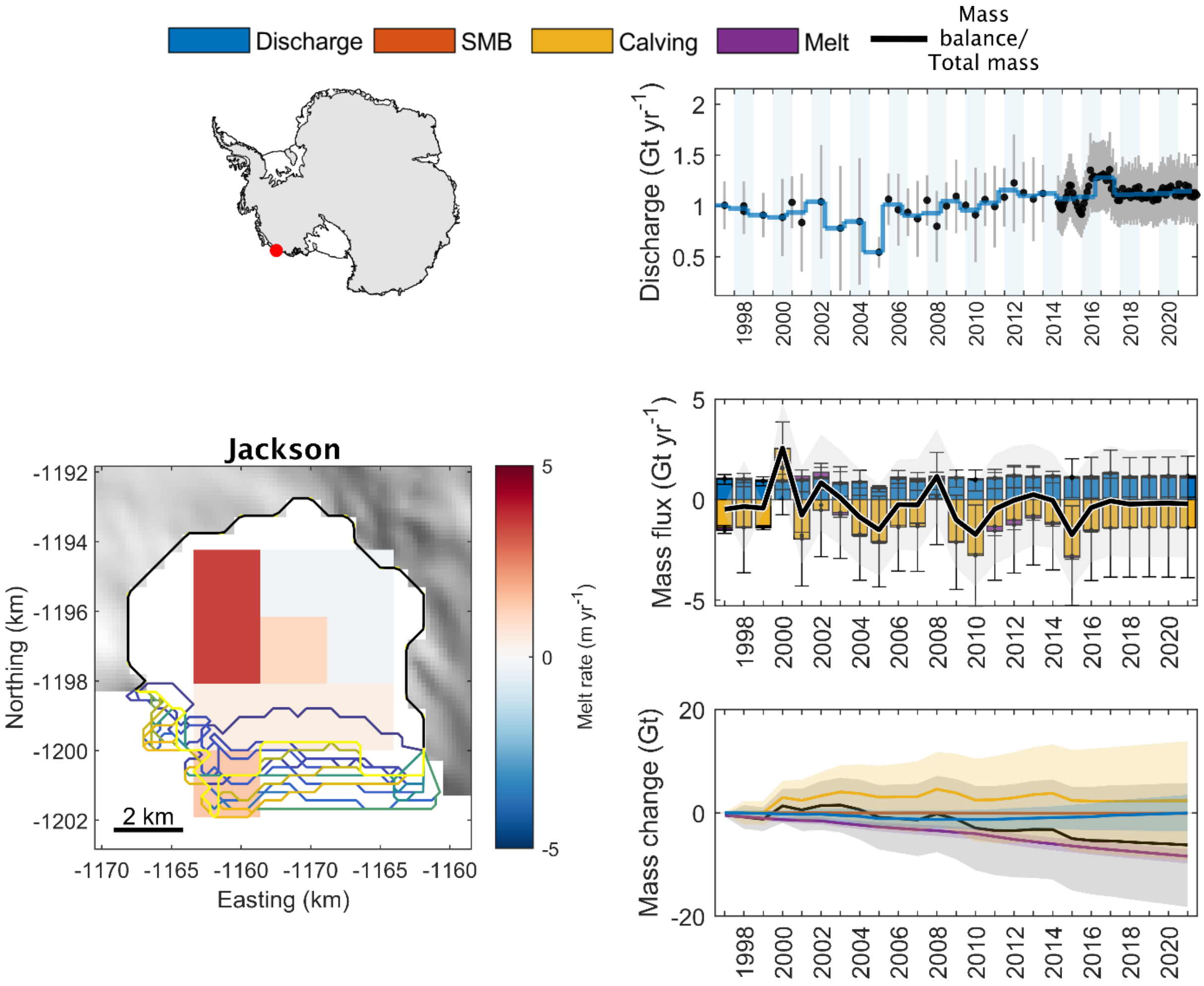

Discharge
SMB
Calving
Melt
Mass balance/ Total mass
Jackson
Northing (km)
Easting (km)
2 km
Melt rate (m yr^{-1})
Discharge (Gt yr^{-1})
Mass flux (Gt yr^{-1})
Mass change (Gt)

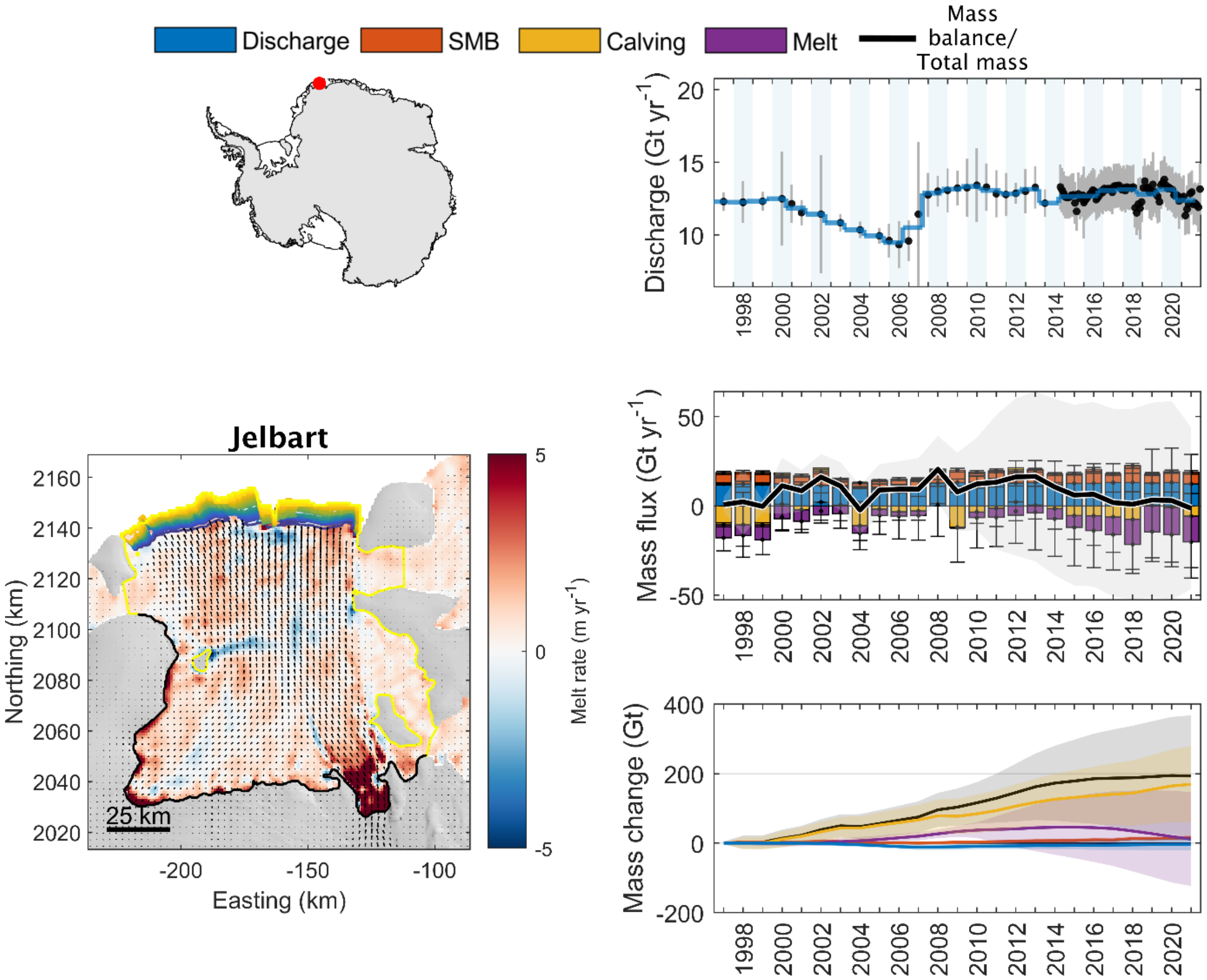

Discharge
SMB
Calving
Melt
Mass balance/ Total mass
Jelbart
25 km
Northing (km)
Easting (km)
Melt rate (m yr^{-1})
Discharge (Gt yr^{-1})
Mass flux (Gt yr^{-1})
Mass change (Gt)

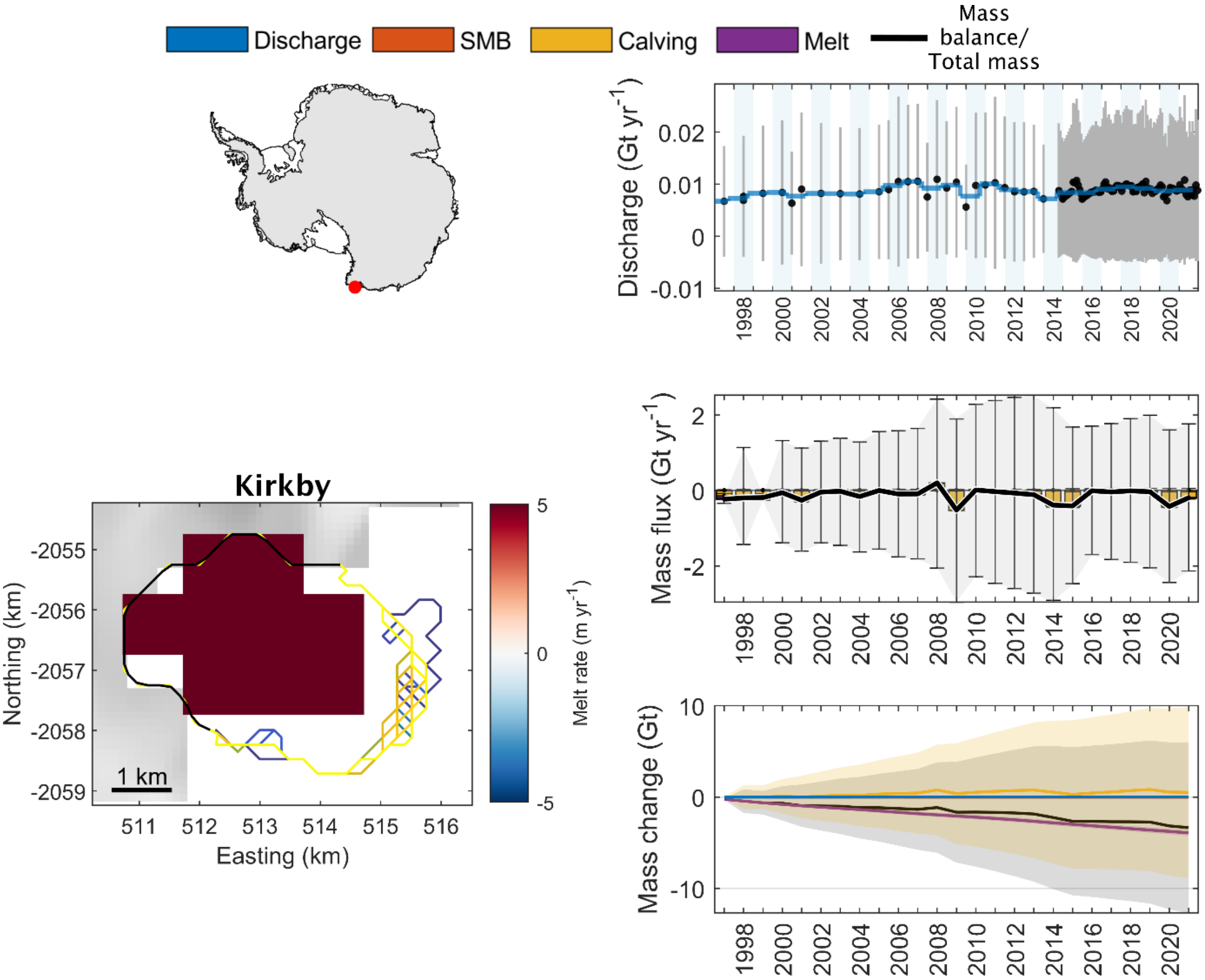
Discharge
SMB
Calving
Melt
Mass balance/ Total mass
Kirkby
Northing (km)
Easting (km)
Melt rate (m yr-1)
1 km
Discharge (Gt yr-1)
Mass flux (Gt yr-1)
Mass change (Gt)

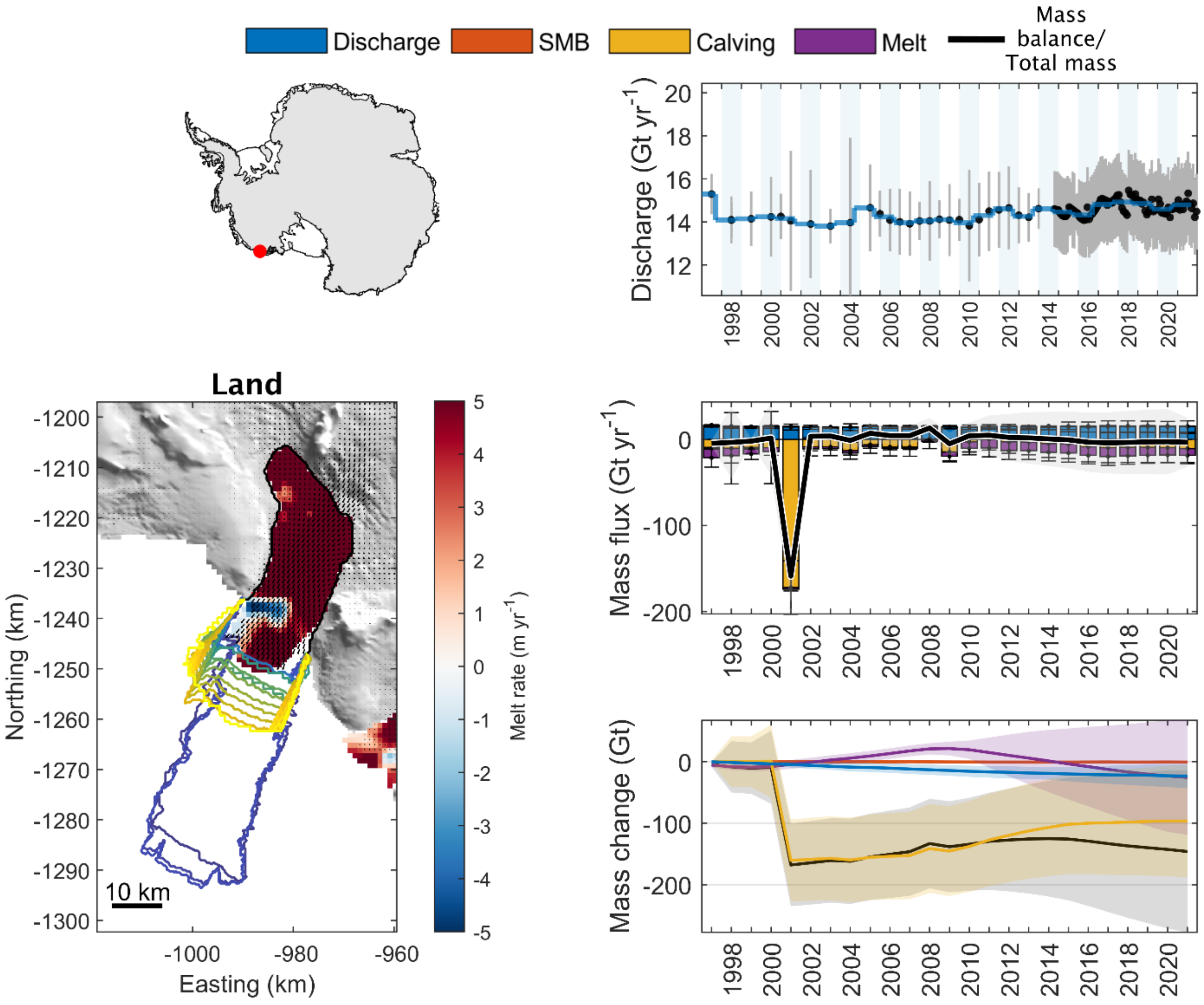

Discharge
SMB
Calving
Melt
Mass balance/ Total mass
Land
Discharge (Gt yr^{-1})
Mass flux (Gt yr^{-1})
Mass change (Gt)
Northing (km)
Easting (km)
Melt rate (m yr^{-1})
10 km

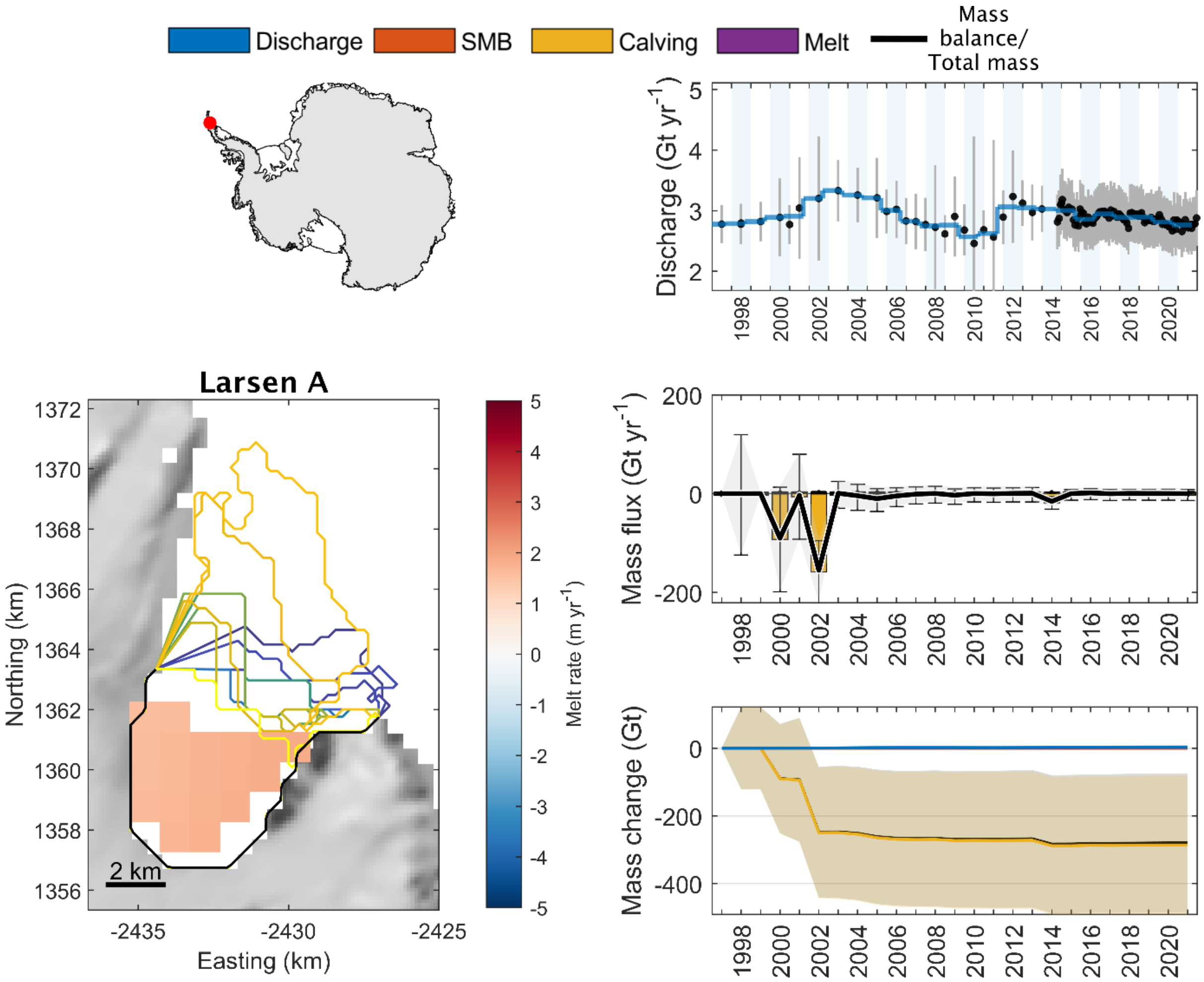

Discharge
SMB
Calving
Melt
Mass balance/ Total mass
Larsen A
Northing (km)
Easting (km)
2 km
Melt rate (m yr-1)
Discharge (Gt yr-1)
Mass flux (Gt yr-1)
Mass change (Gt)

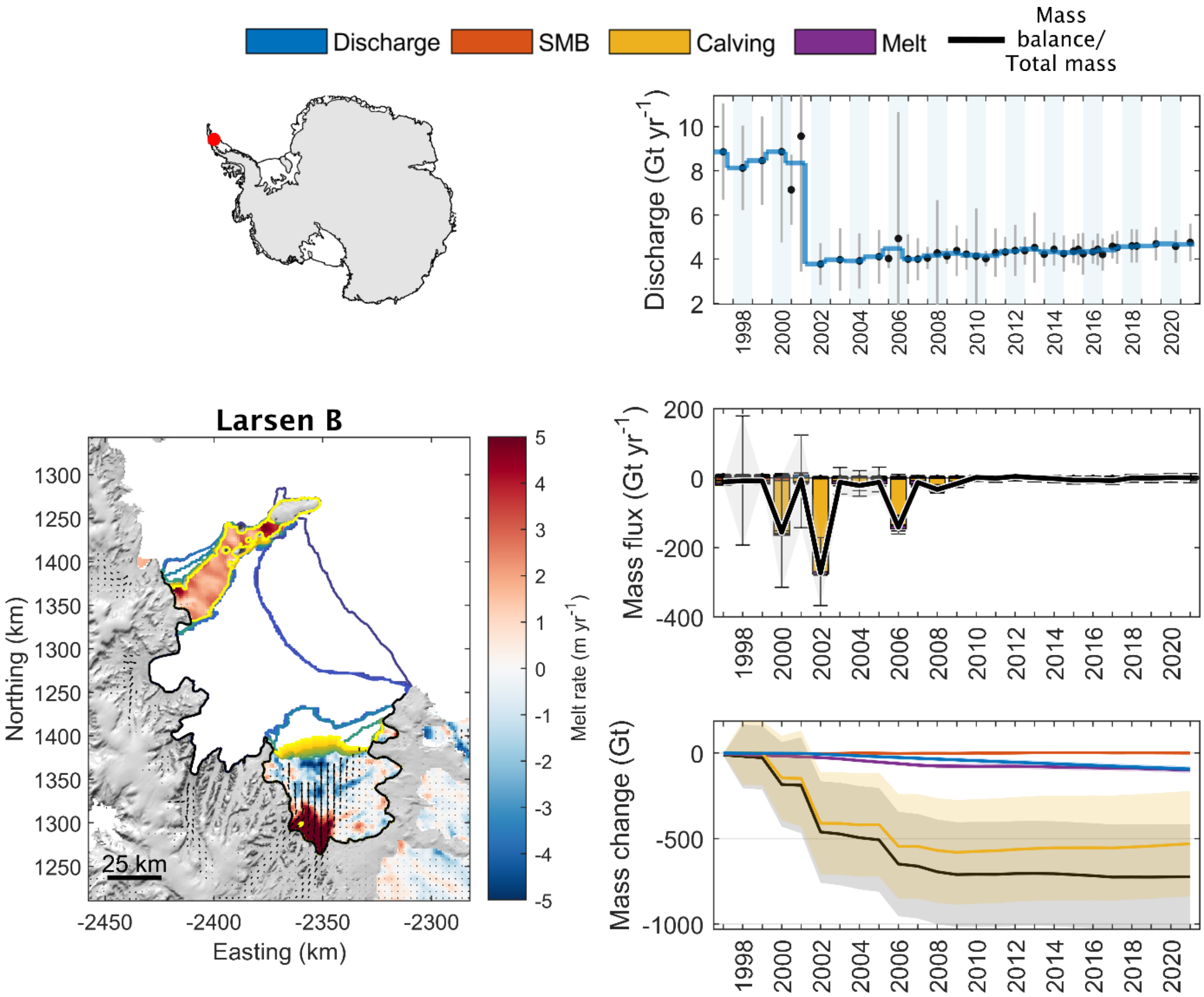

Discharge
SMB
Calving
Melt
Mass balance/ Total mass
Larsen B
25 km
Northing (km)
Easting (km)
Melt rate (m yr-1)
Discharge (Gt yr-1)
Mass flux (Gt yr-1)
Mass change (Gt)

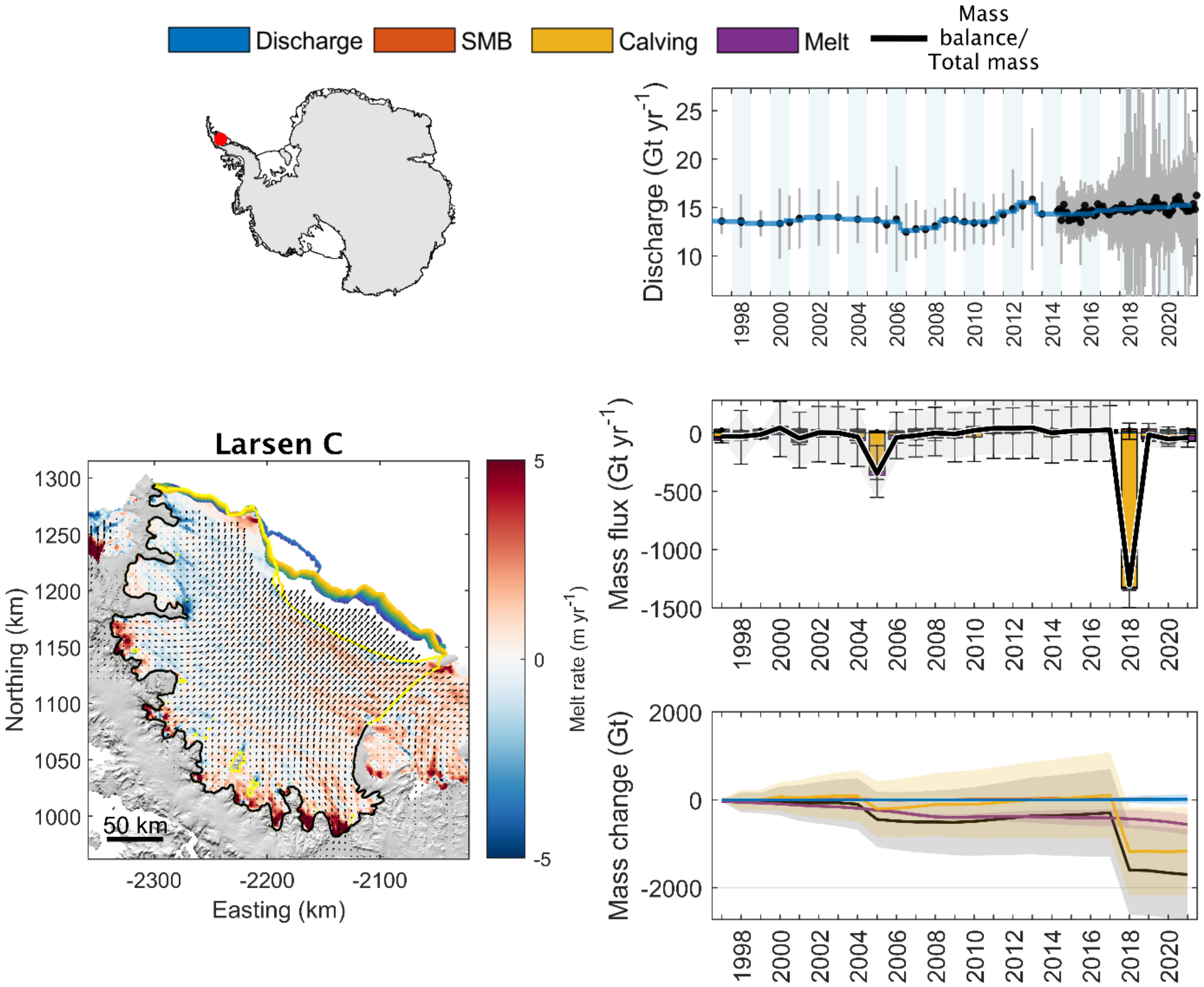
Discharge
SMB
Calving
Melt
Mass balance/ Total mass
Discharge (Gt yr-1)
Larsen C
Northing (km)
Easting (km)
50 km
Melt rate (m yr-1)
Mass flux (Gt yr-1)
Mass change (Gt)

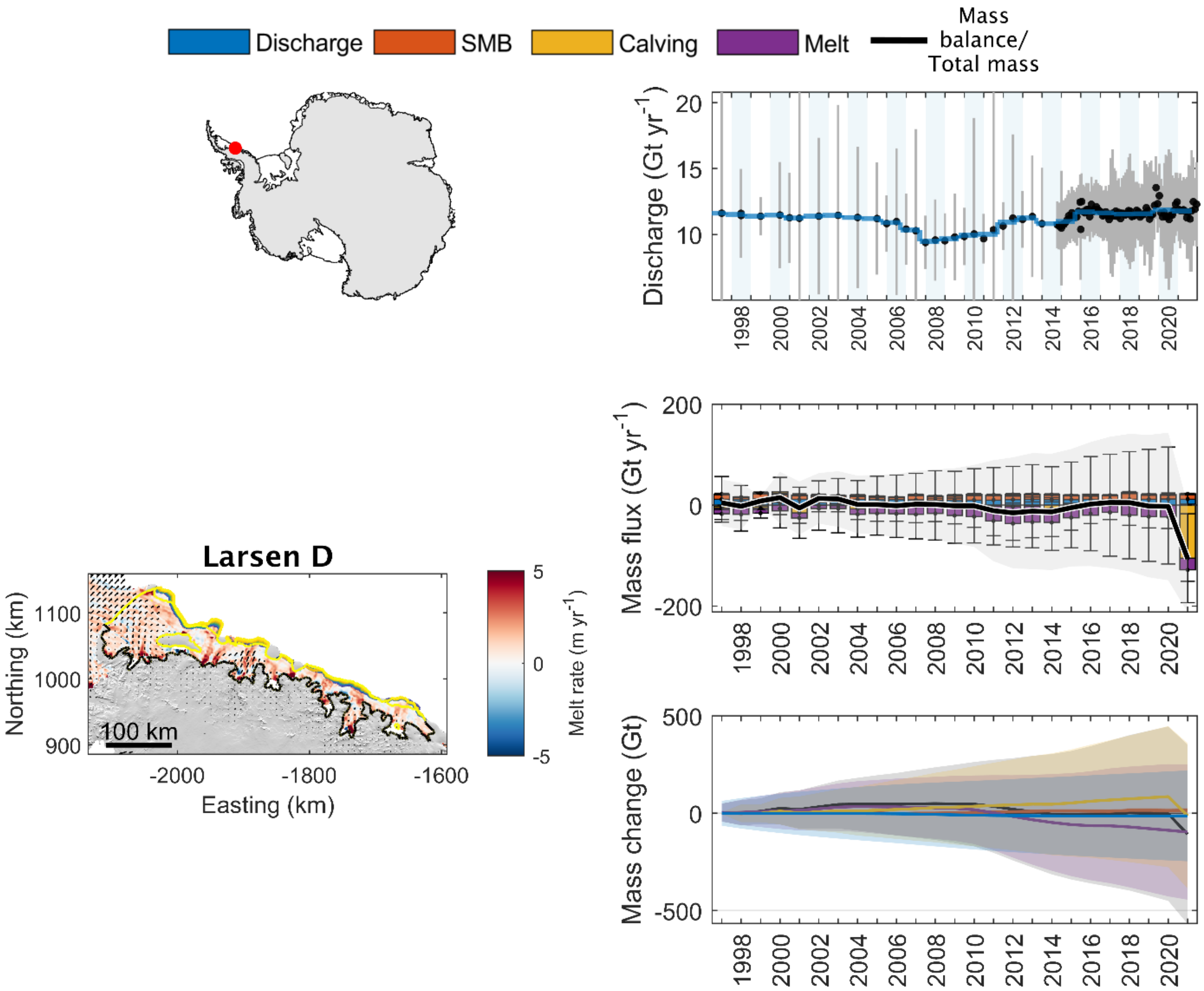
Discharge
SMB
Calving
Melt
Mass balance/ Total mass
Larsen D
Northing (km)
Easting (km)
100 km
Melt rate (m yr-1)
Discharge (Gt yr-1)
Mass flux (Gt yr-1)
Mass change (Gt)

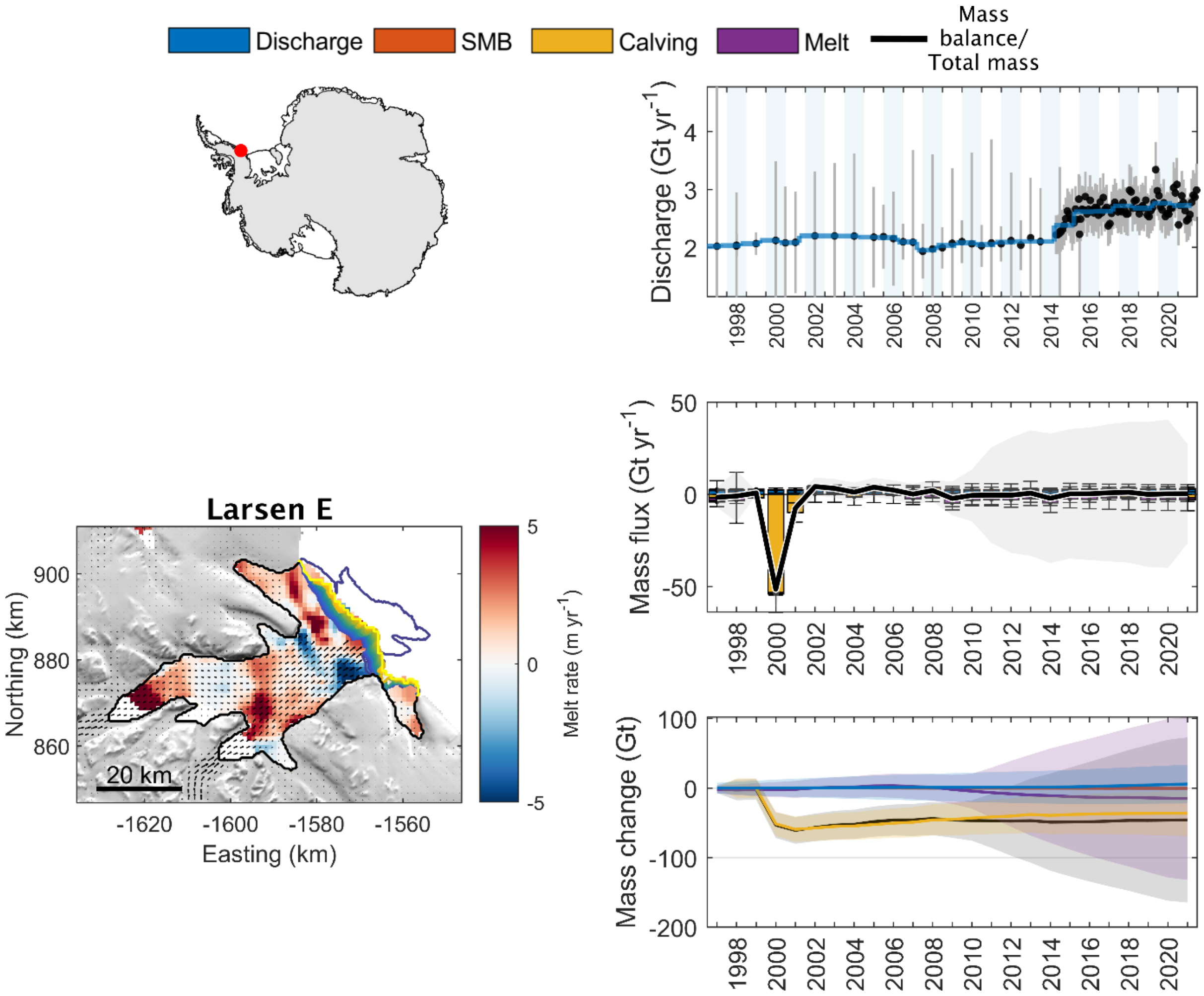
Discharge
SMB
Calving
Melt
Mass balance/ Total mass
Larsen E
Northing (km)
Easting (km)
20 km
Melt rate (m yr^{-1})
Discharge (Gt yr^{-1})
Mass flux (Gt yr^{-1})
Mass change (Gt)

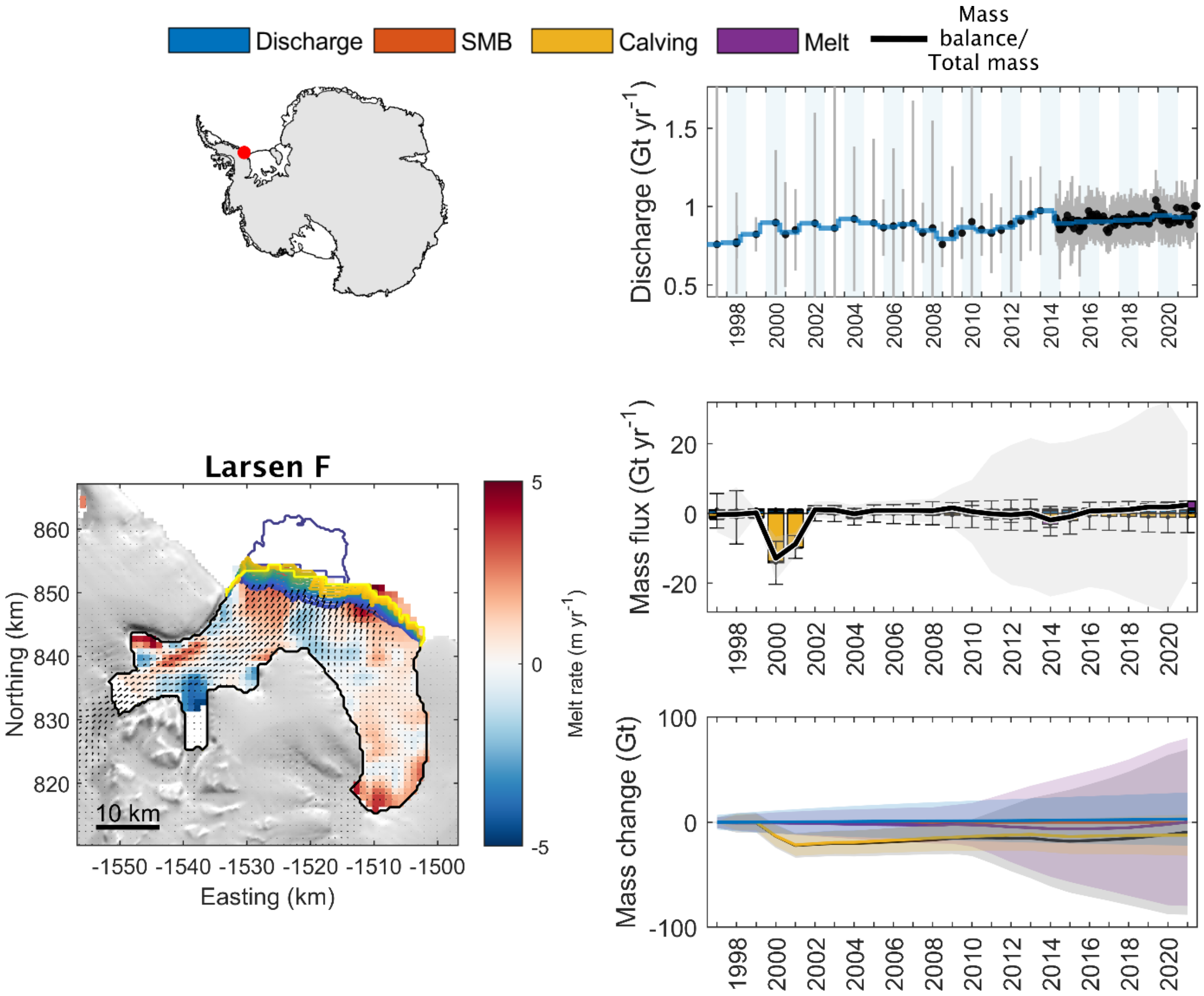

Discharge
SMB
Calving
Melt
Mass balance/ Total mass
Larsen F
Northing (km)
Easting (km)
10 km
Melt rate (m yr^{-1})
Discharge (Gt yr^{-1})
Mass flux (Gt yr^{-1})
Mass change (Gt)

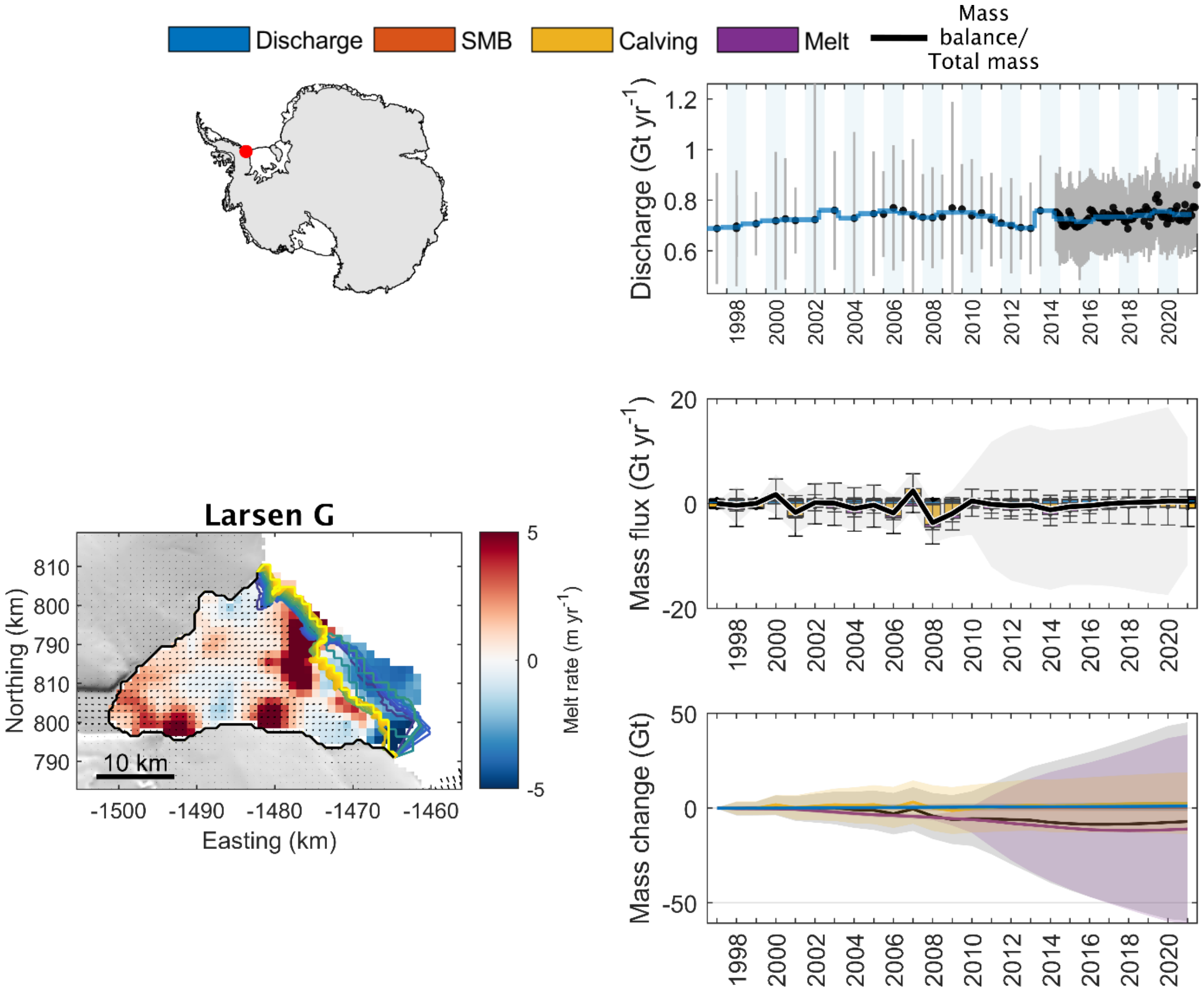
Discharge
SMB
Calving
Melt
Mass balance/ Total mass
Larsen G
Northing (km)
Easting (km)
10 km
Melt rate (m yr-1)
Discharge (Gt yr-1)
Mass flux (Gt yr-1)
Mass change (Gt)

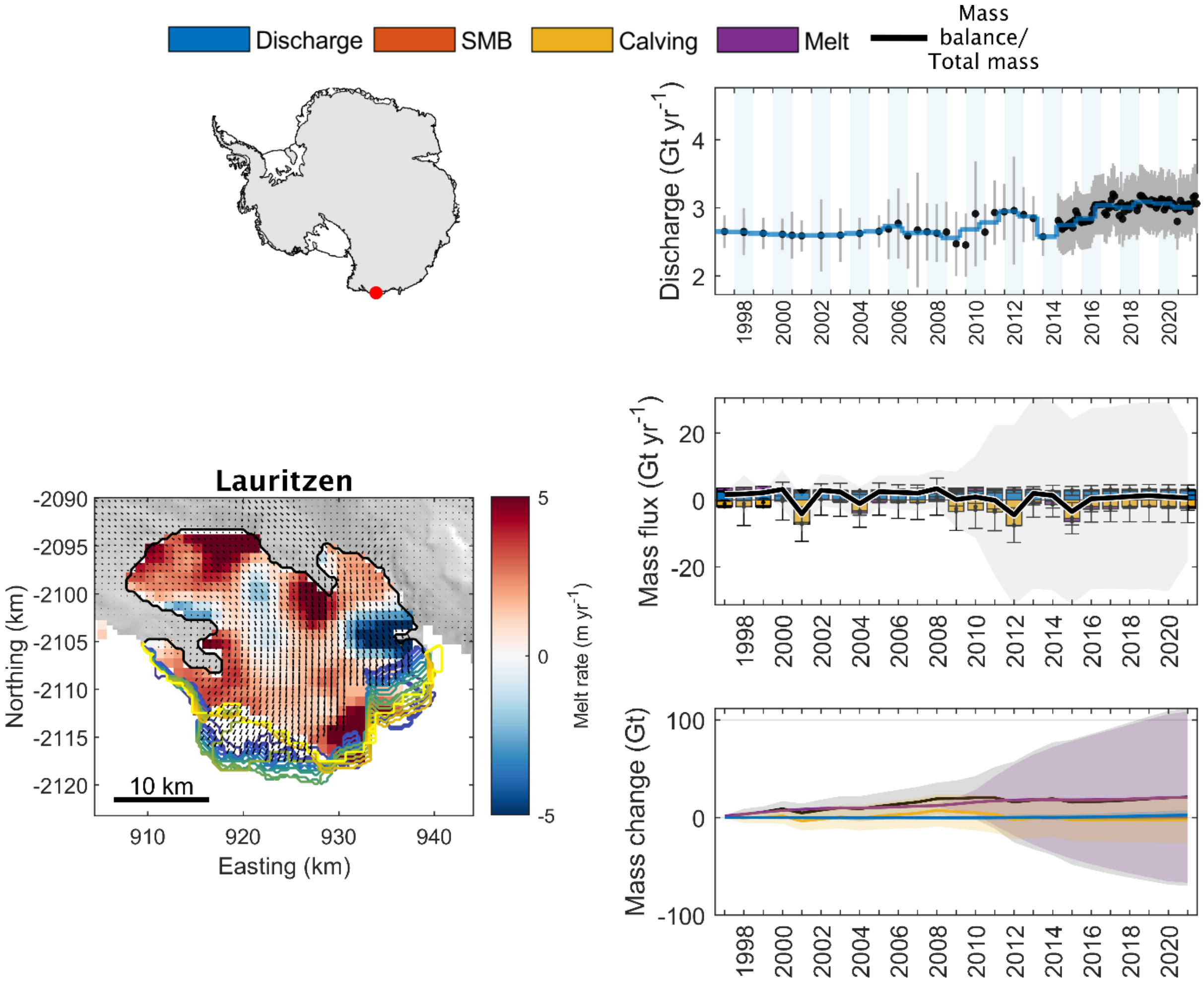
Discharge
SMB
Calving
Melt
Mass balance/ Total mass
Lauritzen
Northing (km)
Easting (km)
10 km
Melt rate (m yr^{-1})
Discharge (Gt yr^{-1})
Mass flux (Gt yr^{-1})
Mass change (Gt)

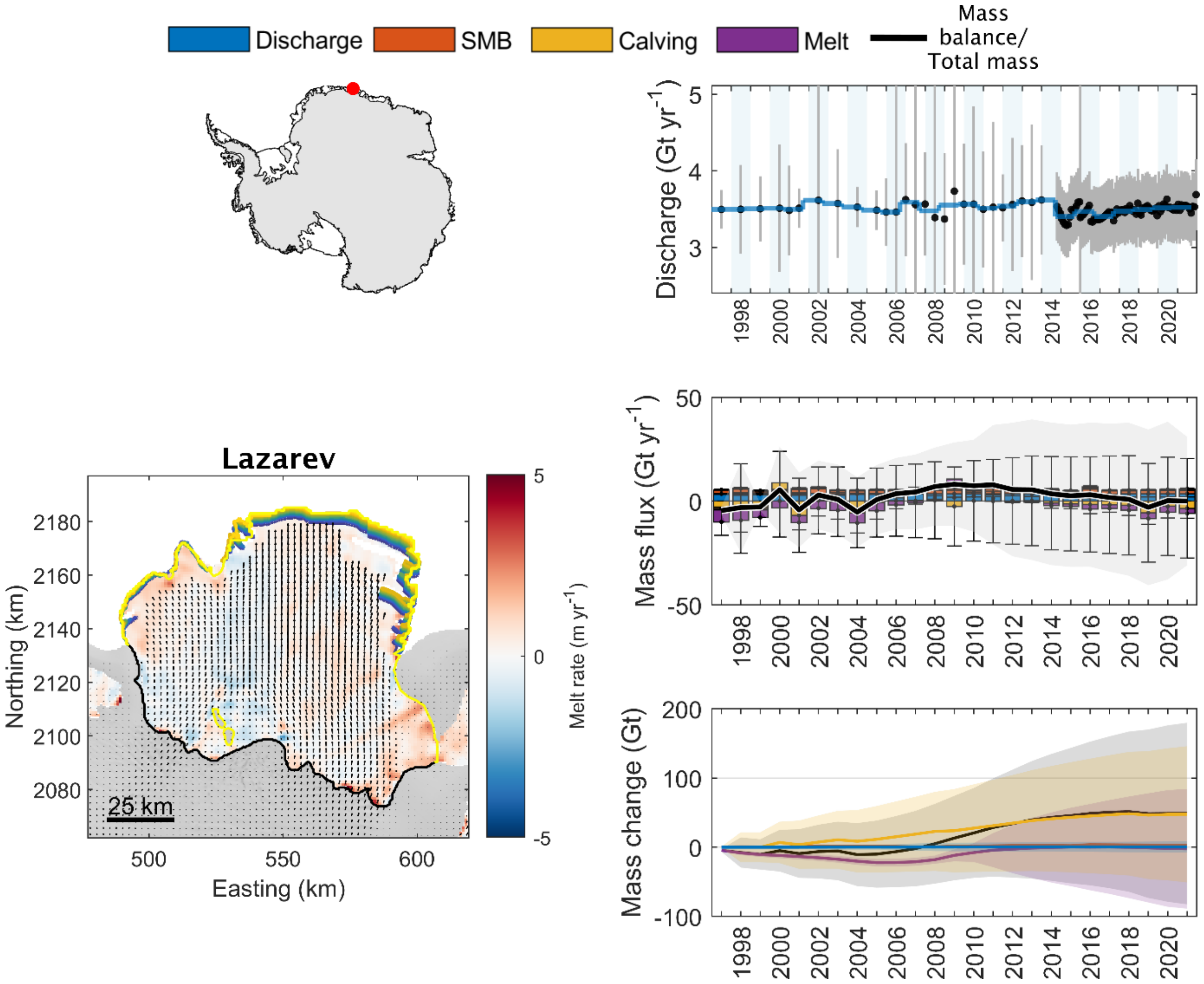

Discharge
SMB
Calving
Melt
Mass balance/ Total mass
Lazarev
Northing (km)
Easting (km)
25 km
Melt rate (m yr-1)
Discharge (Gt yr-1)
Mass flux (Gt yr-1)
Mass change (Gt)

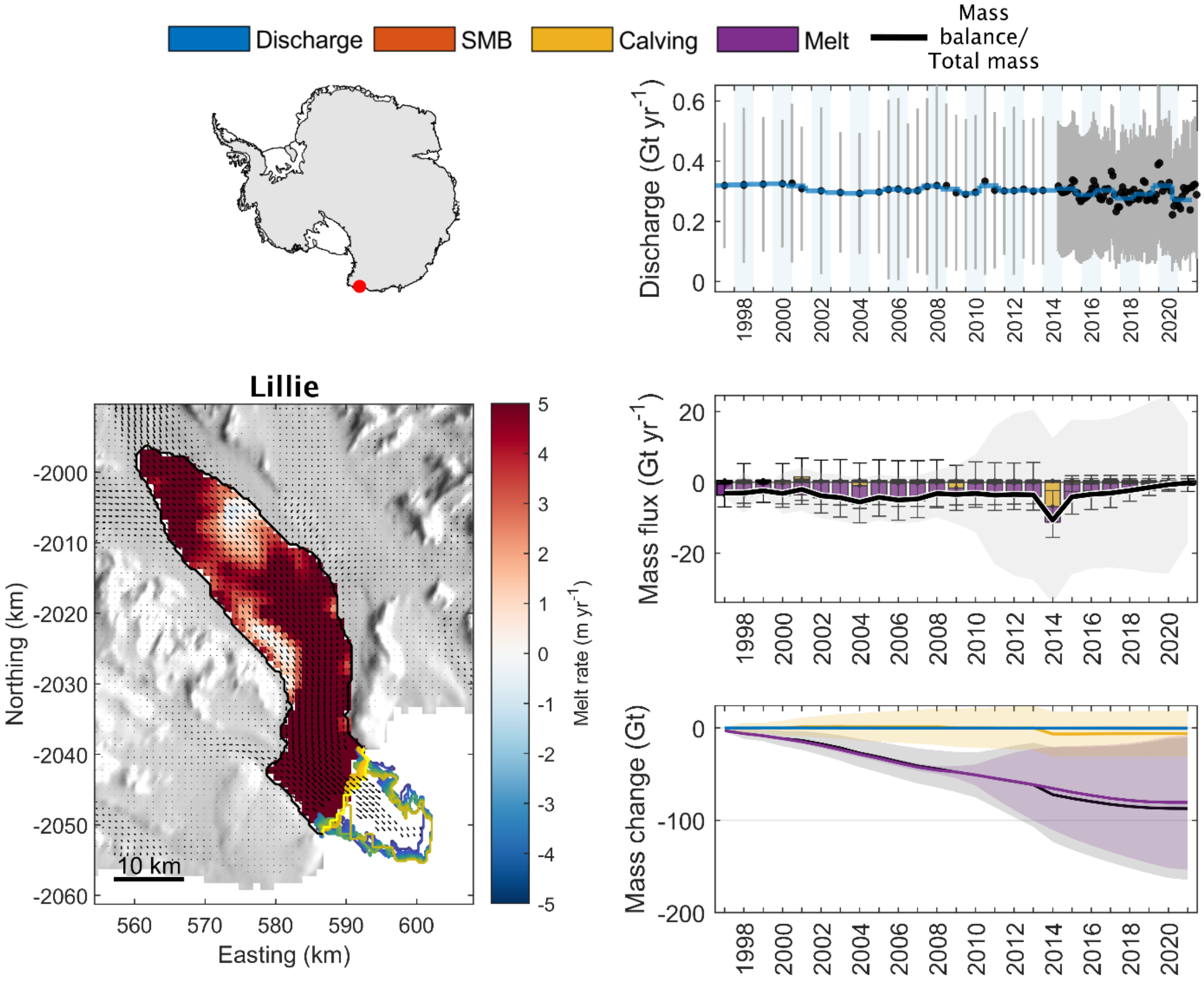
Discharge
SMB
Calving
Melt
Mass balance/ Total mass
Lillie
Northing (km)
Easting (km)
10 km
Melt rate (m yr^{-1})
Discharge (Gt yr^{-1})
Mass flux (Gt yr^{-1})
Mass change (Gt)

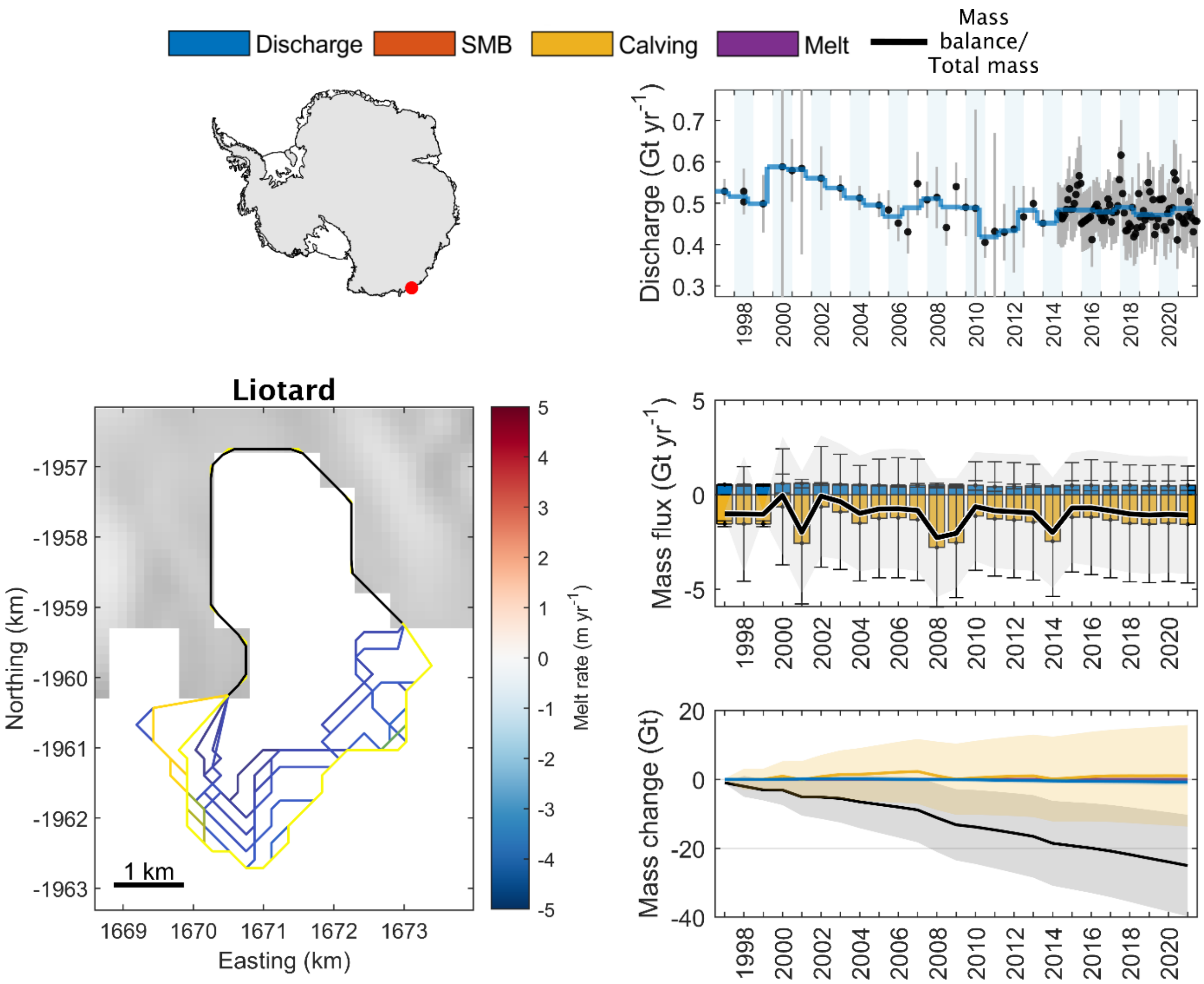
Discharge
SMB
Calving
Melt
Mass balance/ Total mass
Liotard
Northing (km)
Easting (km)
1 km
Melt rate (m yr-1)
Discharge (Gt yr-1)
Mass flux (Gt yr-1)
Mass change (Gt)

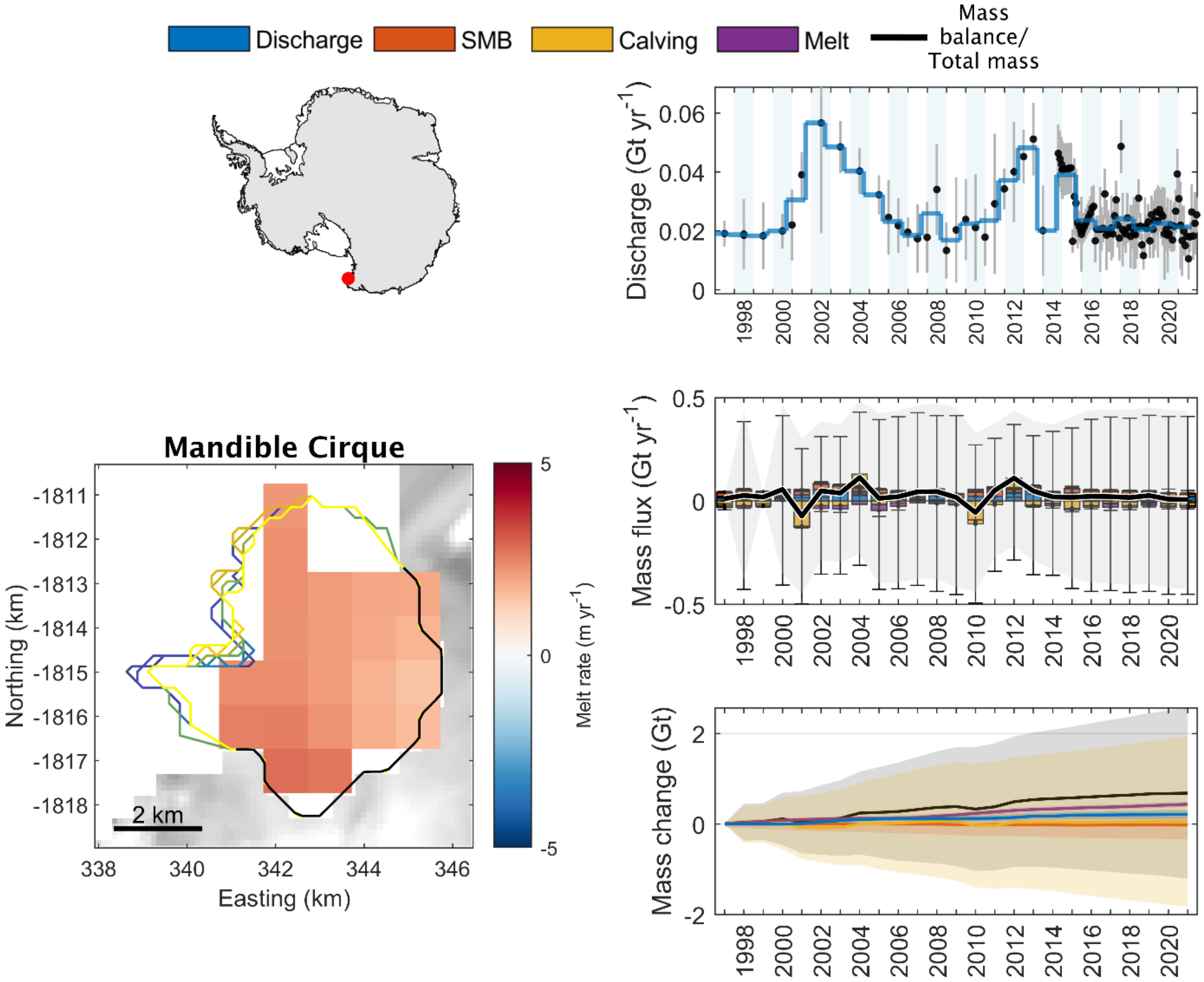
Discharge
SMB
Calving
Melt
Mass balance/ Total mass
Mandible Cirque
Northing (km)
Easting (km)
2 km
Melt rate (m yr^{-1})
Discharge (Gt yr^{-1})
Mass flux (Gt yr^{-1})
Mass change (Gt)

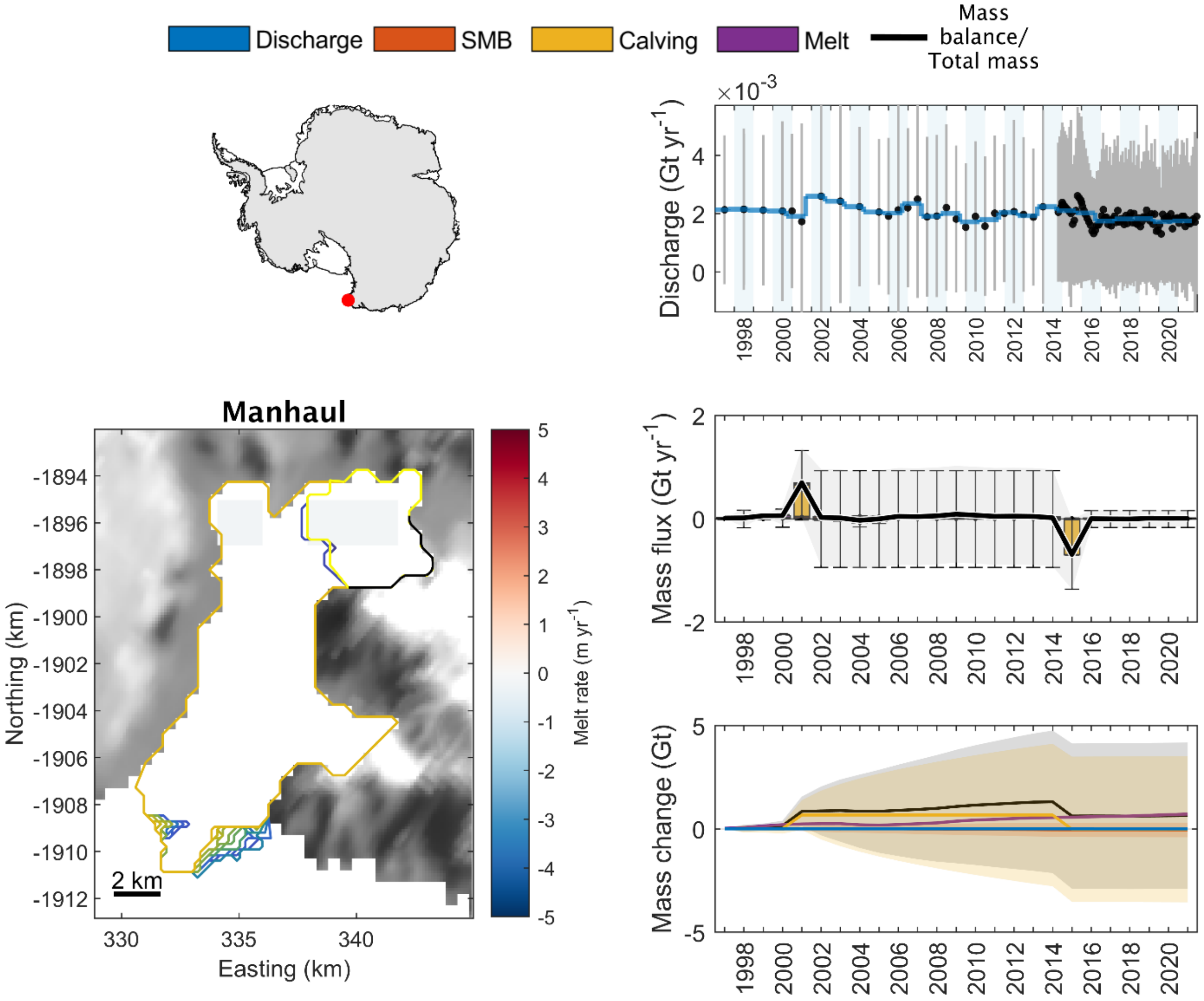
Discharge
SMB
Calving
Melt
Mass balance/ Total mass
Manhaul
2 km
Northing (km)
Easting (km)
Melt rate (m yr-1)
Discharge (Gt yr-1)
×10-3
Mass flux (Gt yr-1)
Mass change (Gt)

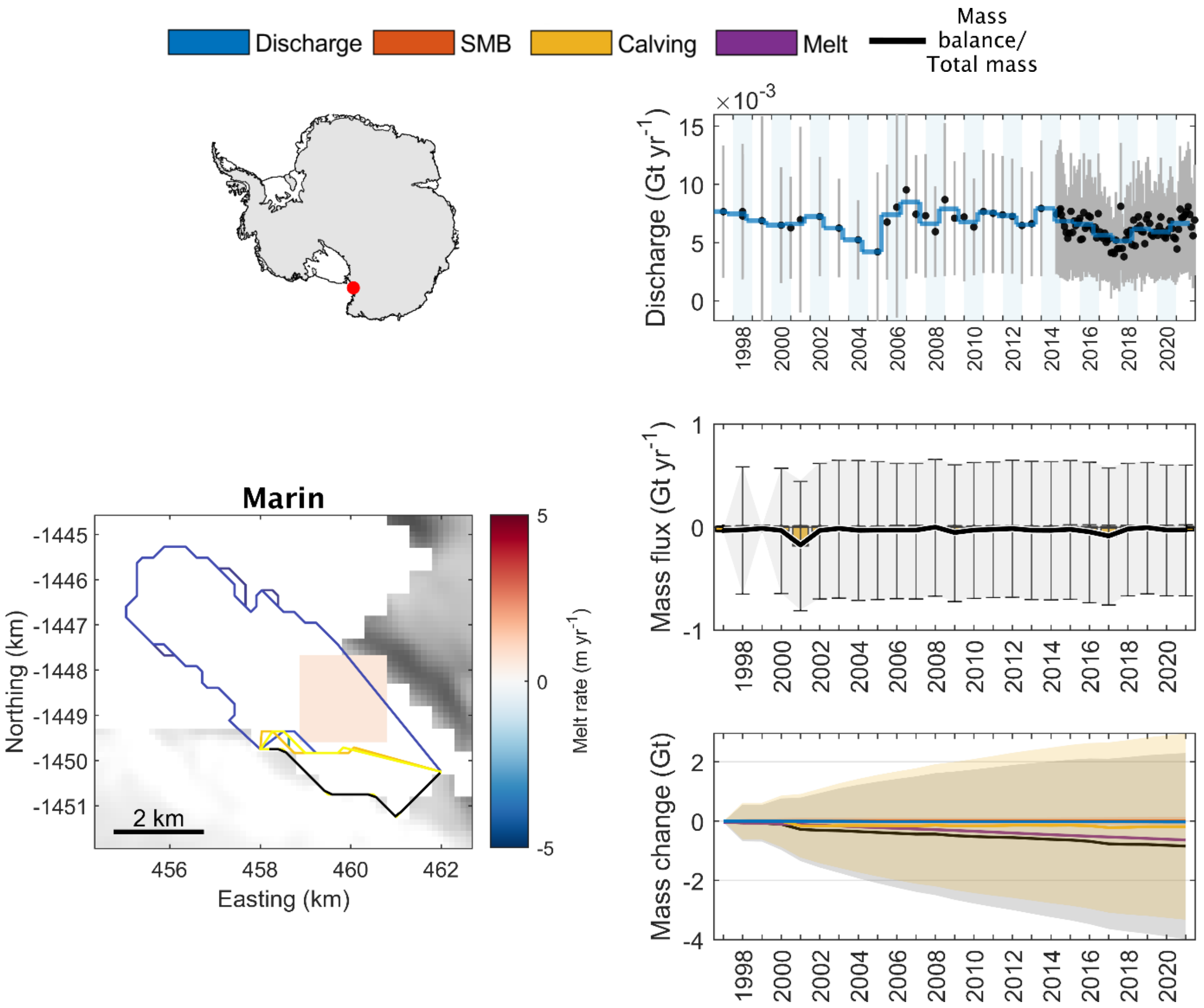

Discharge
SMB
Calving
Melt
Mass balance/ Total mass
Marin
Northing (km)
Easting (km)
2 km
Melt rate (m yr^-1)
Discharge (Gt yr^-1)
×10^-3
Mass flux (Gt yr^-1)
Mass change (Gt)

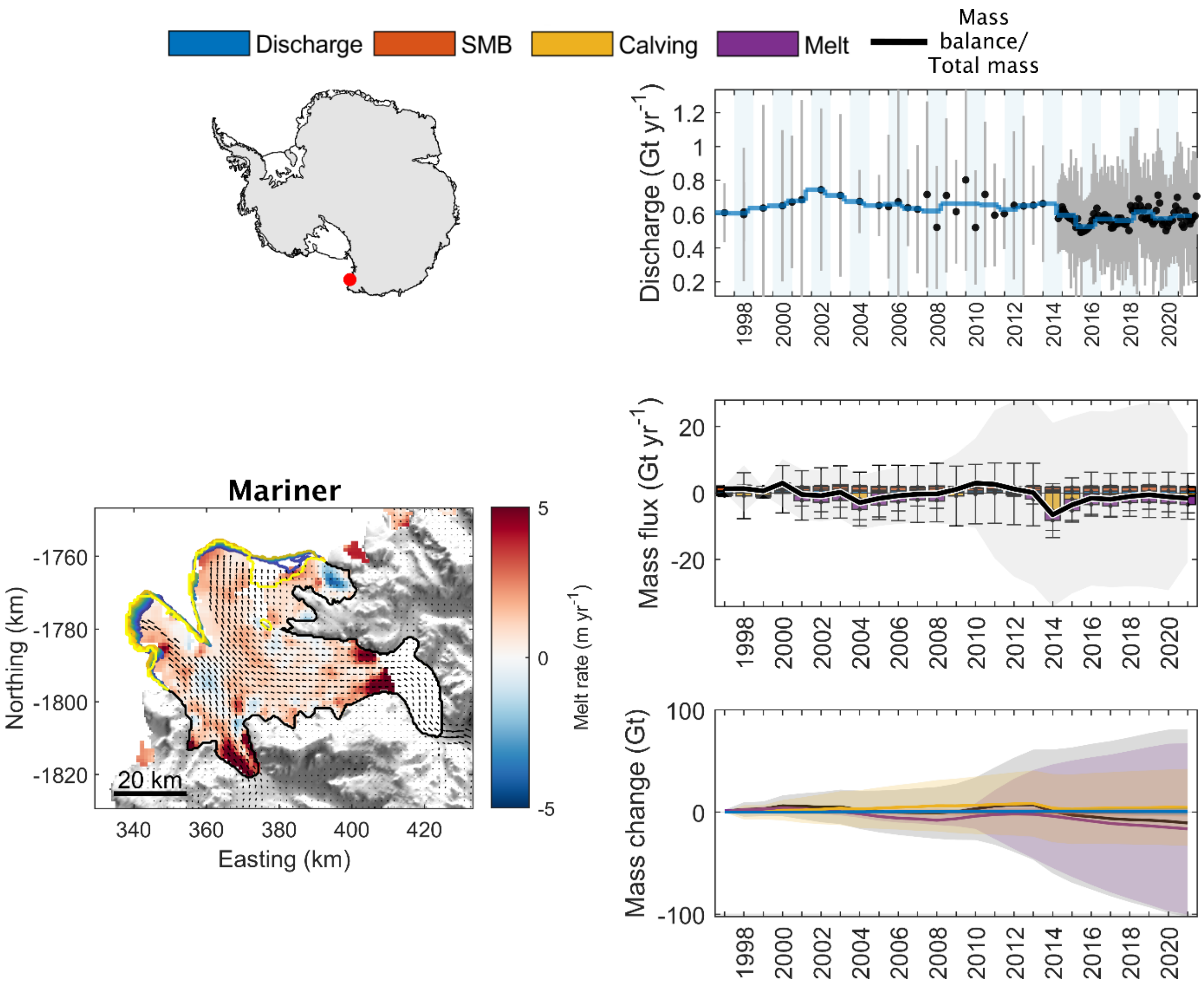
Discharge
SMB
Calving
Melt
Mass balance/ Total mass
Discharge (Gt yr⁻¹)
Mass flux (Gt yr⁻¹)
Mass change (Gt)
Mariner
Northing (km)
Easting (km)
20 km
Melt rate (m yr⁻¹)

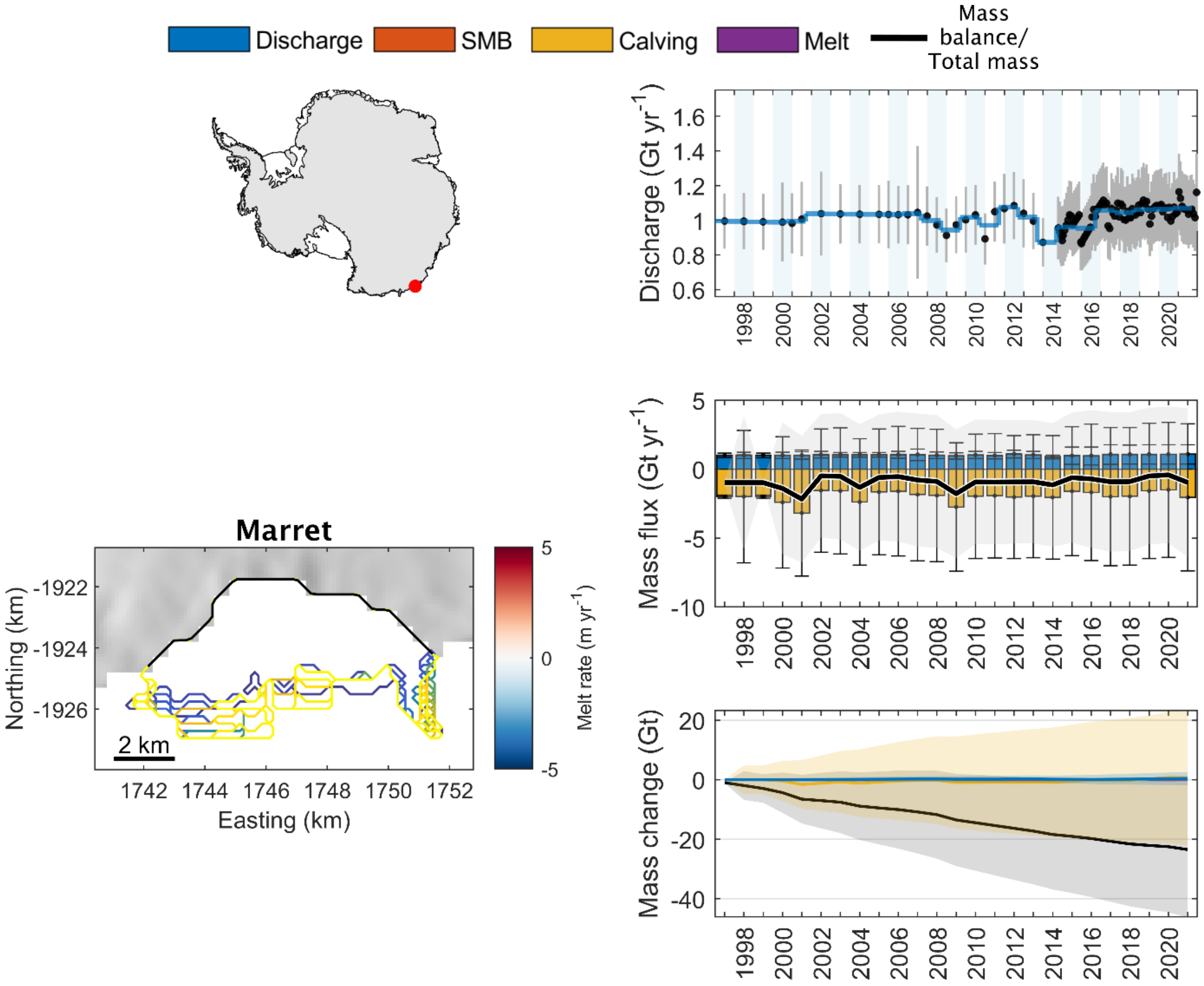
Discharge
SMB
Calving
Melt
Mass balance/ Total mass
Marret
Northing (km)
Easting (km)
2 km
Melt rate (m yr^{-1})
Discharge (Gt yr^{-1})
Mass flux (Gt yr^{-1})
Mass change (Gt)

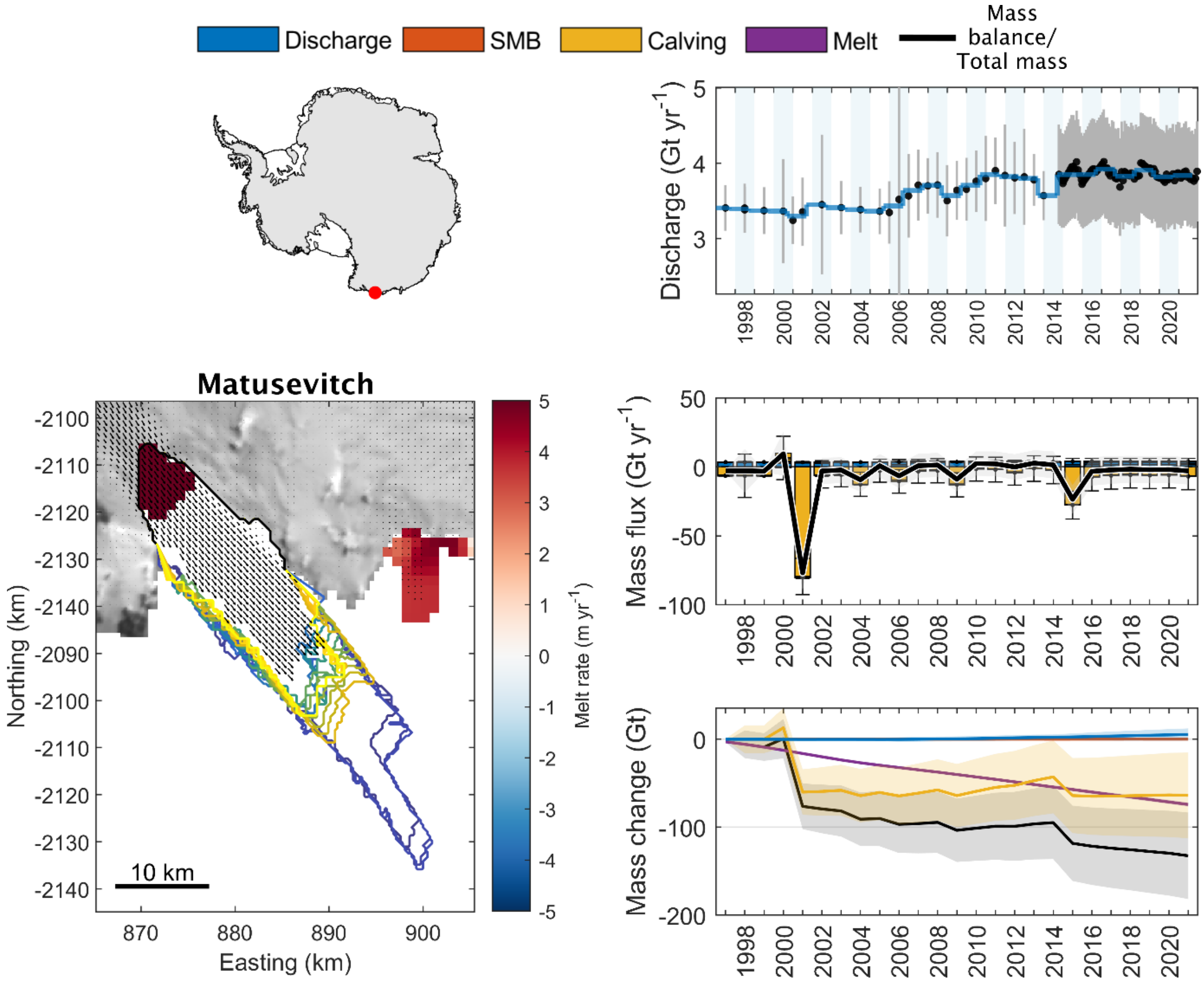
Discharge
SMB
Calving
Melt
Mass balance/ Total mass
Discharge (Gt yr-1)
Matusevitch
Northing (km)
Easting (km)
10 km
Melt rate (m yr-1)
Mass flux (Gt yr-1)
Mass change (Gt)

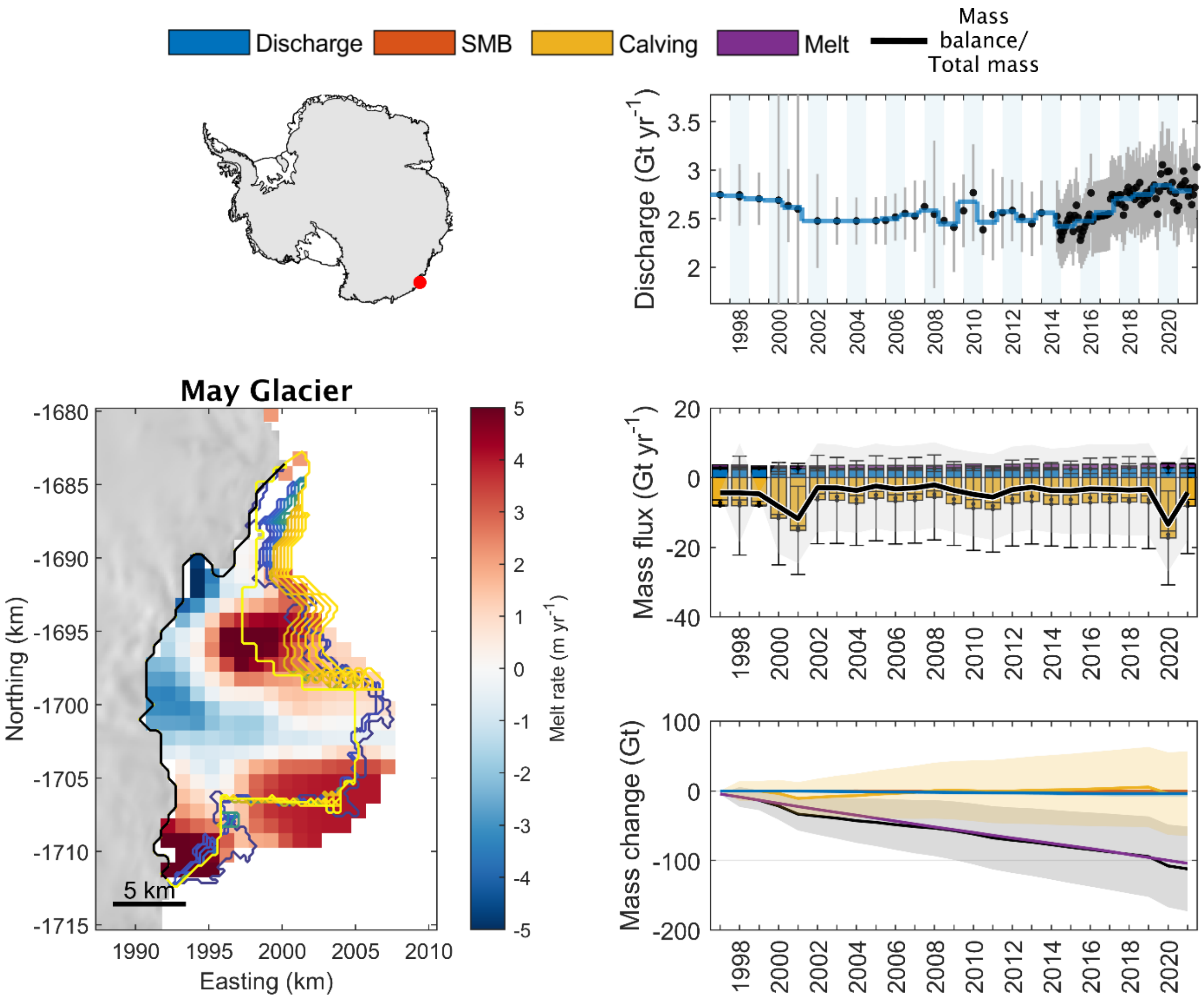
Discharge
SMB
Calving
Melt
Mass balance/ Total mass
May Glacier
Northing (km)
Easting (km)
5 km
Melt rate (m yr-1)
Discharge (Gt yr-1)
Mass flux (Gt yr-1)
Mass change (Gt)

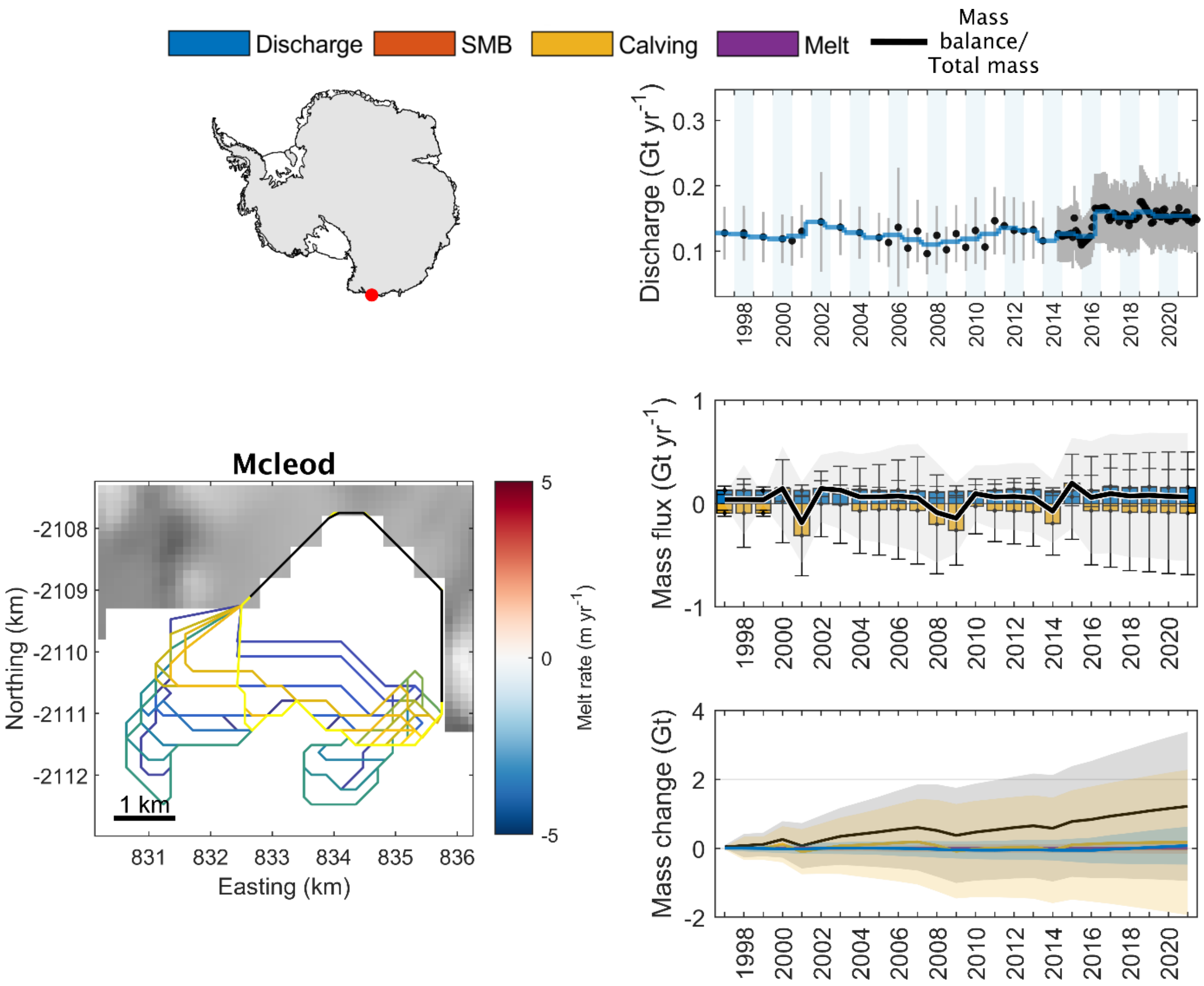

Discharge
SMB
Calving
Melt
Mass balance/ Total mass
Mcleod
Northing (km)
-2108
-2109
-2110
-2111
-2112
831
832
833
834
835
836
Easting (km)
1 km
Melt rate (m yr^{-1})
5
0
-5
Discharge (Gt yr^{-1})
Mass flux (Gt yr^{-1})
Mass change (Gt)
1998
2000
2002
2004
2006
2008
2010
2012
2014
2016
2018
2020

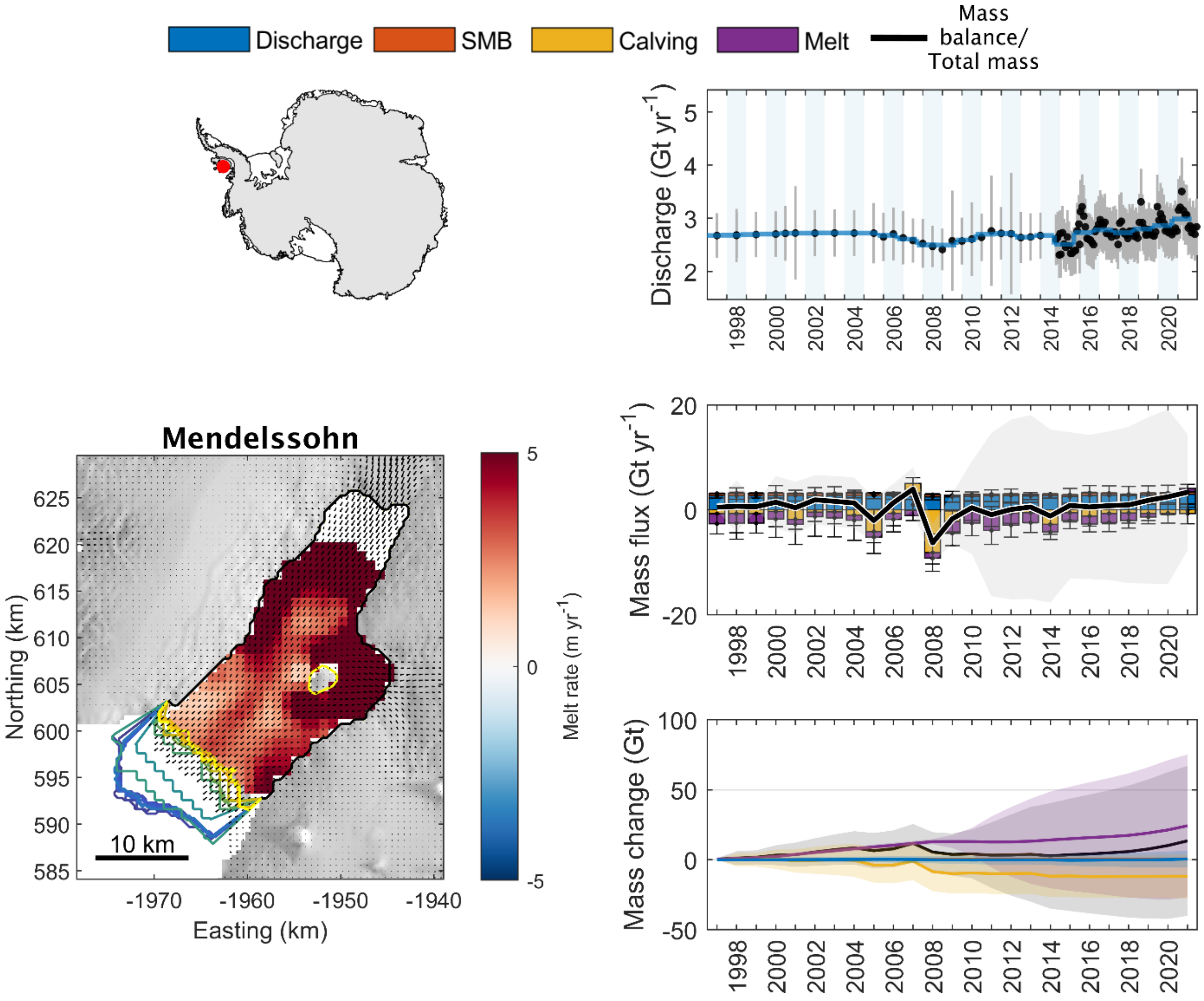

Discharge
SMB
Calving
Melt
Mass balance/ Total mass
Mendelssohn
Northing (km)
Easting (km)
10 km
Melt rate (m yr-1)
Discharge (Gt yr-1)
Mass flux (Gt yr-1)
Mass change (Gt)

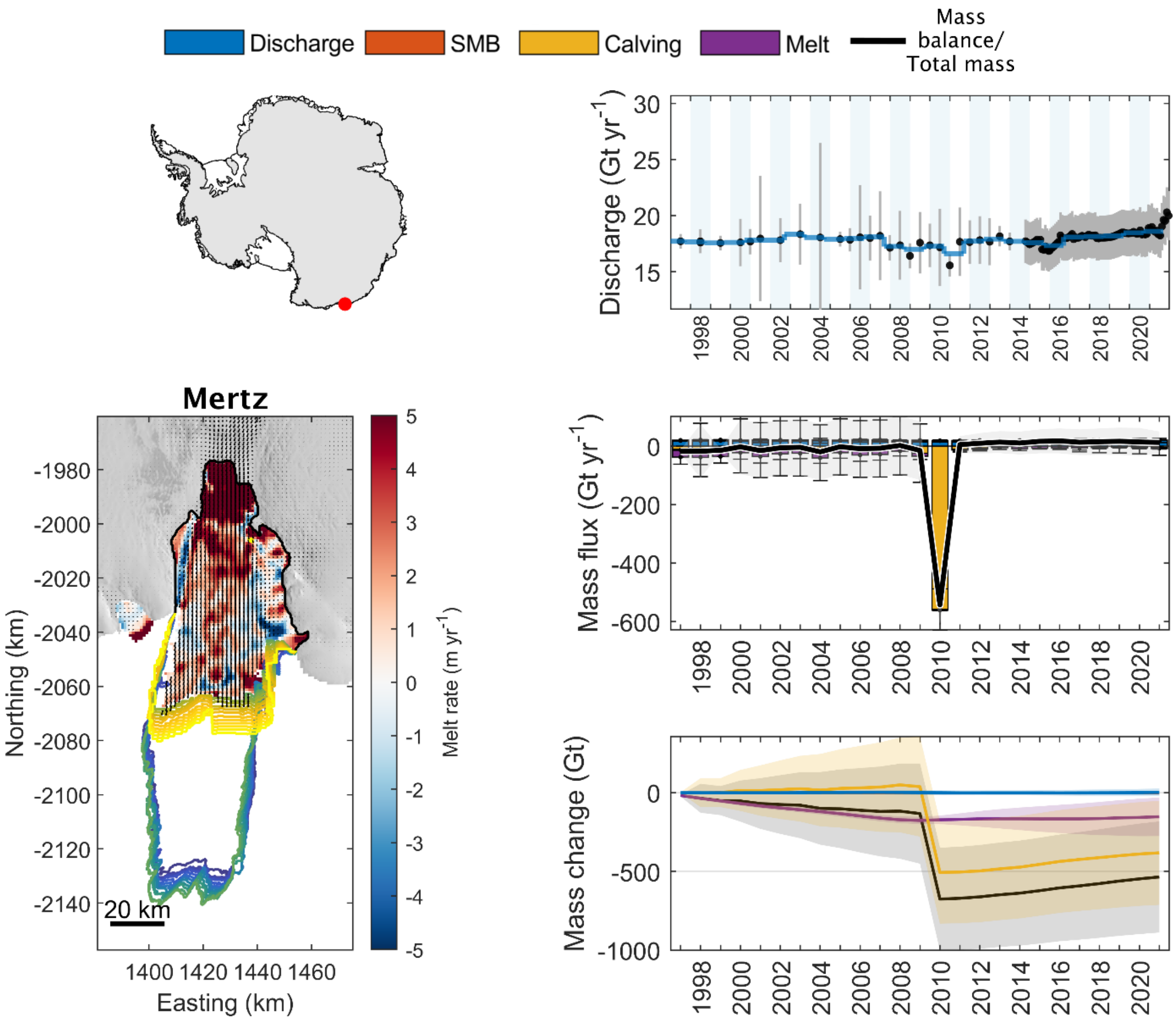

Discharge
SMB
Calving
Melt
Mass balance/ Total mass
Mertz
Northing (km)
Easting (km)
20 km
Melt rate (m yr^{-1})
Discharge (Gt yr^{-1})
Mass flux (Gt yr^{-1})
Mass change (Gt)

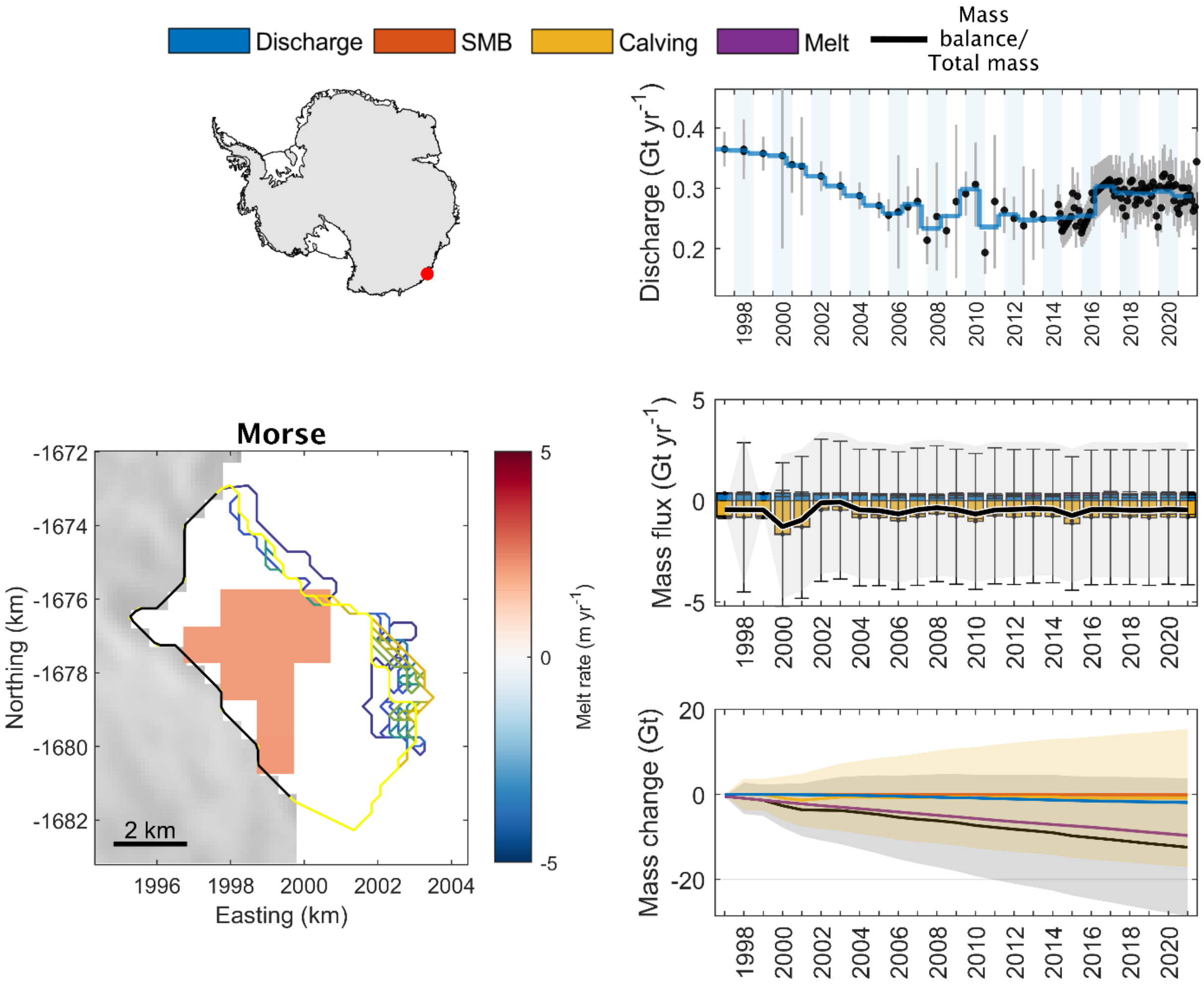
Discharge
SMB
Calving
Melt
Mass balance/ Total mass
Discharge (Gt yr-1)
Morse
Northing (km)
Easting (km)
2 km
Melt rate (m yr-1)
Mass flux (Gt yr-1)
Mass change (Gt)

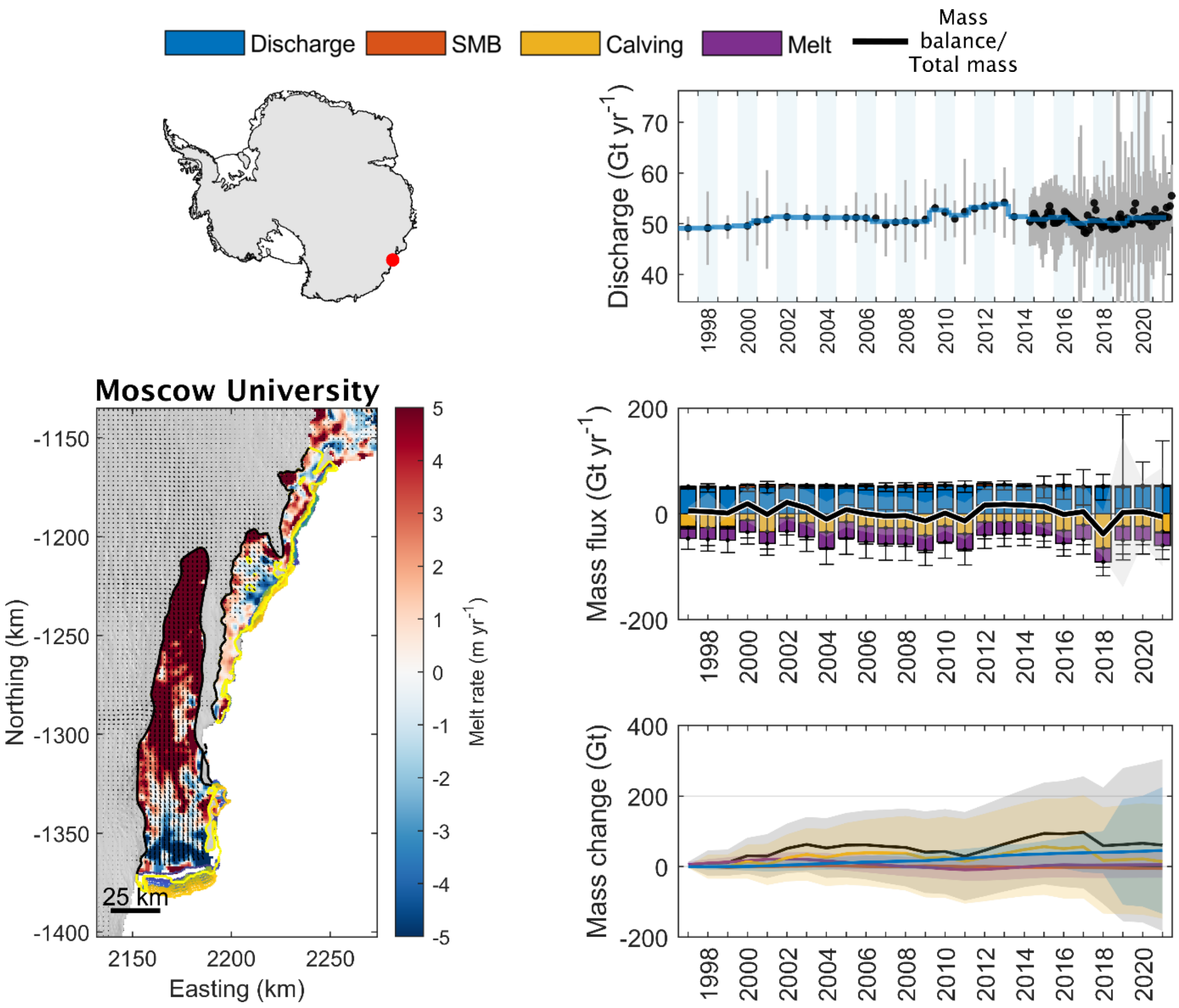
Discharge
SMB
Calving
Melt
Mass balance/ Total mass
Moscow University
25 km
Northing (km)
Easting (km)
Melt rate (m yr^{-1})
Discharge (Gt yr^{-1})
Mass flux (Gt yr^{-1})
Mass change (Gt)

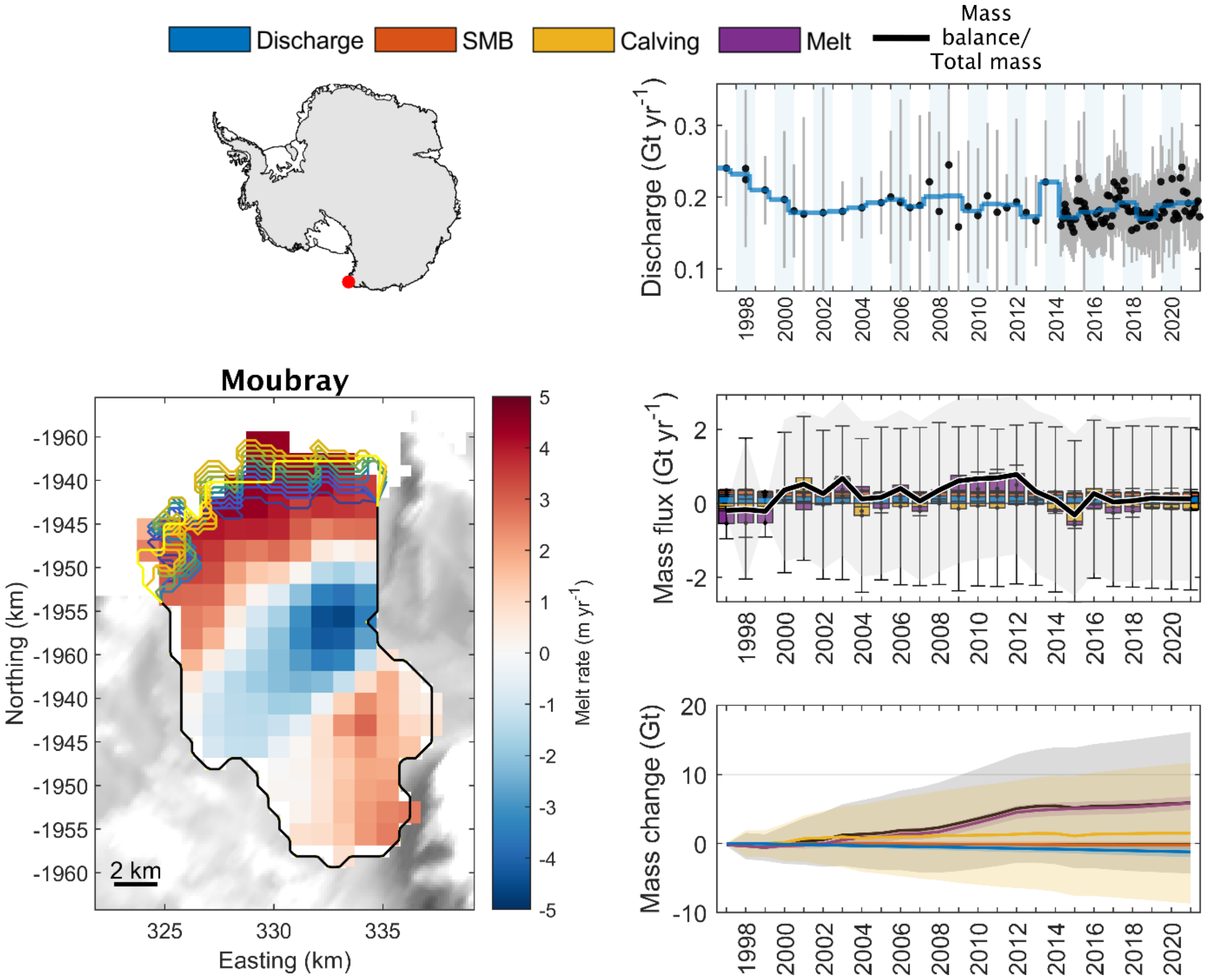

Discharge
SMB
Calving
Melt
Mass balance/ Total mass
Moubray
Northing (km)
Easting (km)
2 km
Melt rate (m yr-1)
Discharge (Gt yr-1)
Mass flux (Gt yr-1)
Mass change (Gt)

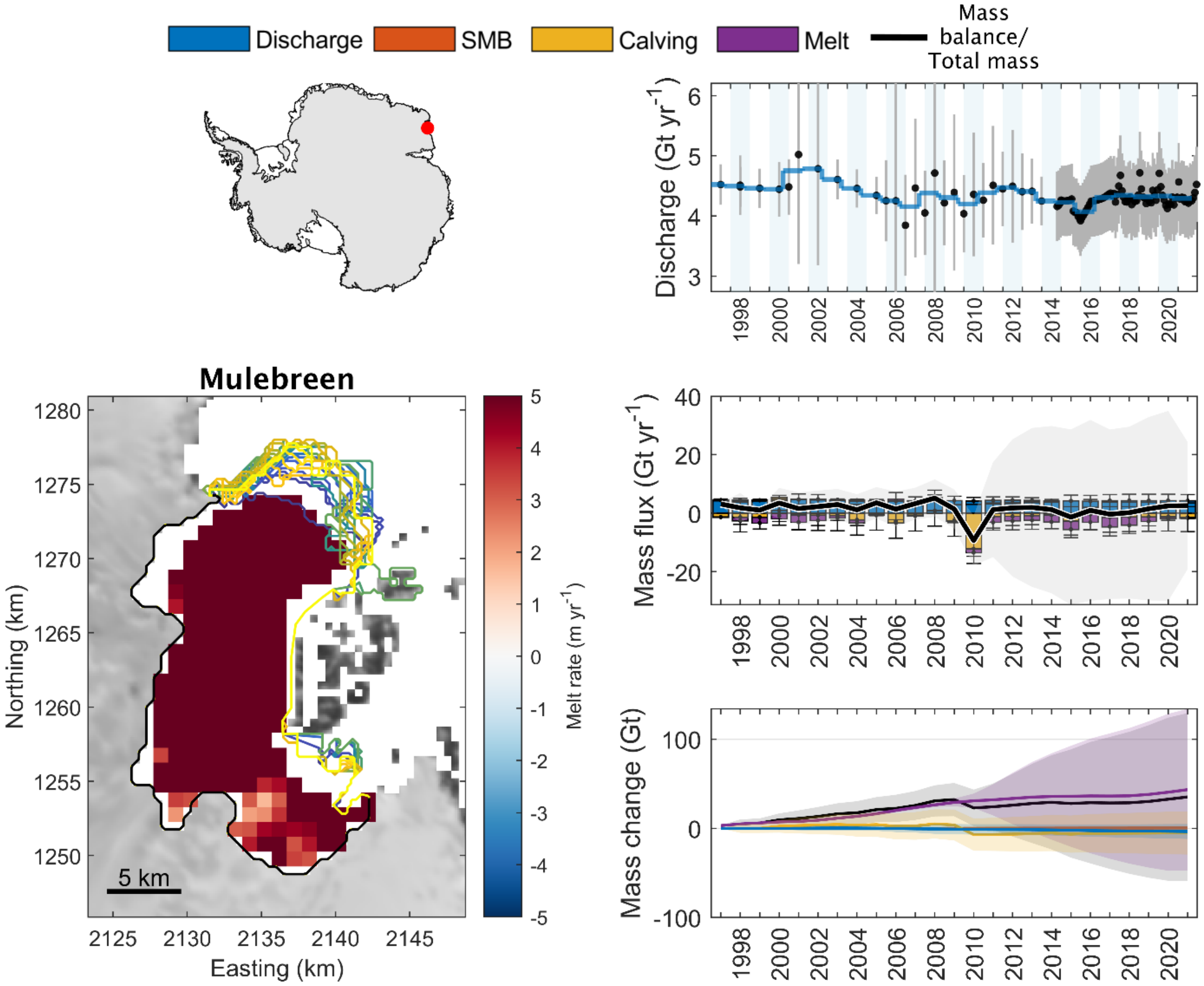

Discharge
SMB
Calving
Melt
Mass balance/ Total mass
Mulebreen
Discharge (Gt yr-1)
Mass flux (Gt yr-1)
Mass change (Gt)
Northing (km)
Easting (km)
Melt rate (m yr-1)
5 km

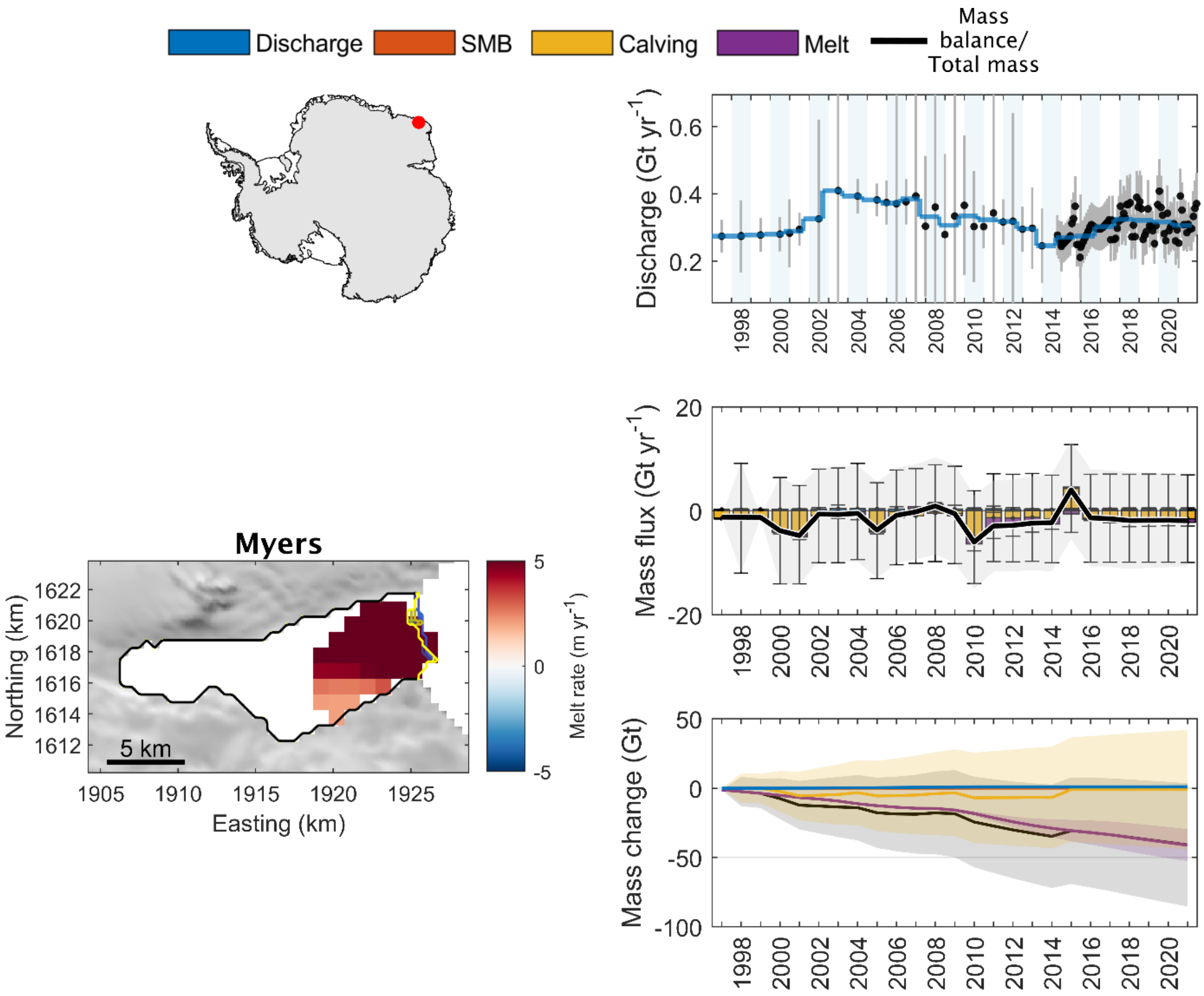

Discharge
SMB
Calving
Melt
Mass balance/ Total mass
Myers
Northing (km)
Easting (km)
5 km
Melt rate (m yr-1)
Discharge (Gt yr-1)
Mass flux (Gt yr-1)
Mass change (Gt)

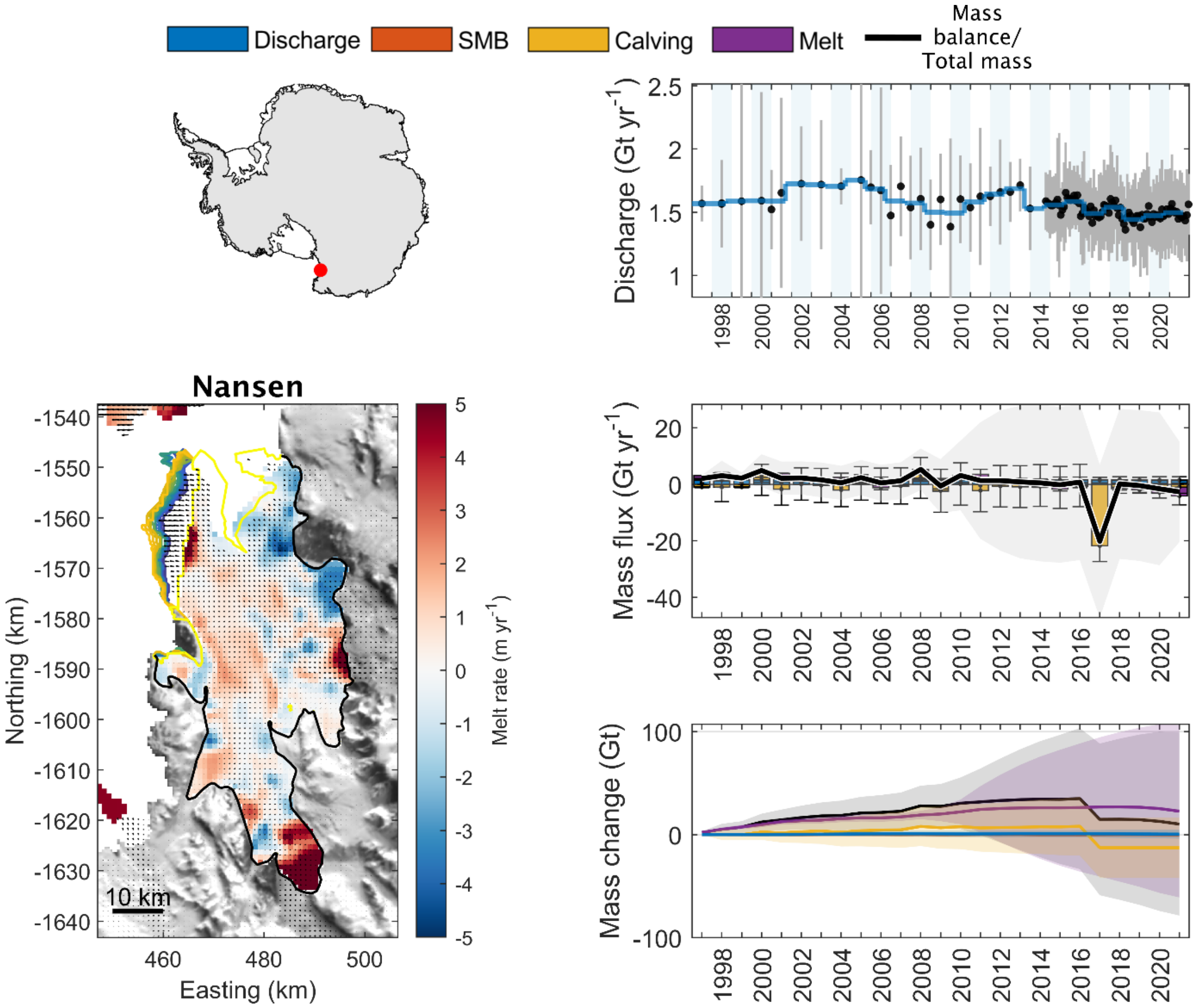

Discharge
SMB
Calving
Melt
Mass balance/ Total mass
Nansen
Discharge (Gt yr^{-1})
Mass flux (Gt yr^{-1})
Mass change (Gt)
Northing (km)
Easting (km)
Melt rate (m yr^{-1})
10 km

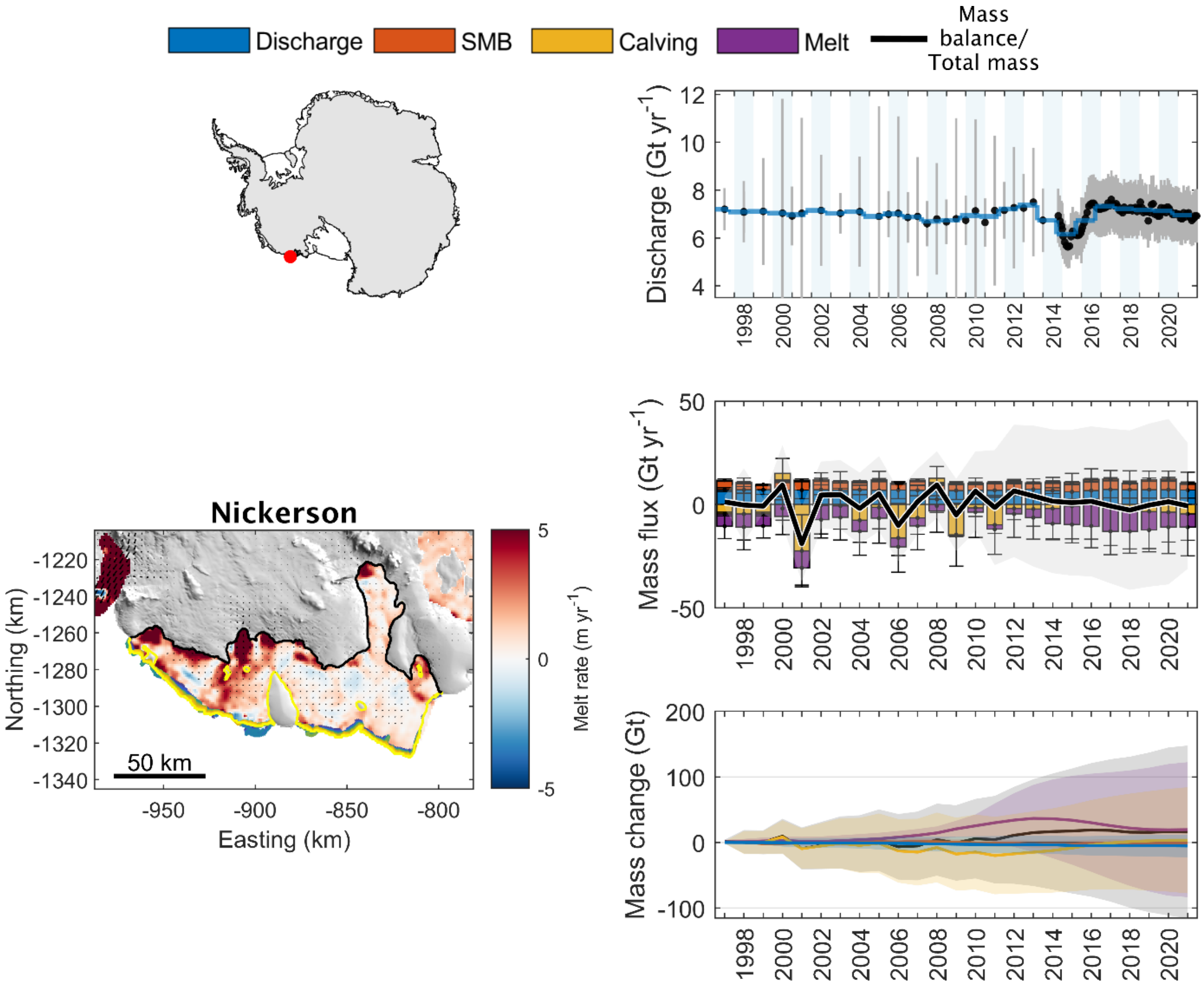
Discharge
SMB
Calving
Melt
Mass balance/ Total mass
Nickerson
Northing (km)
Easting (km)
50 km
Melt rate (m yr-1)
Discharge (Gt yr-1)
Mass flux (Gt yr-1)
Mass change (Gt)

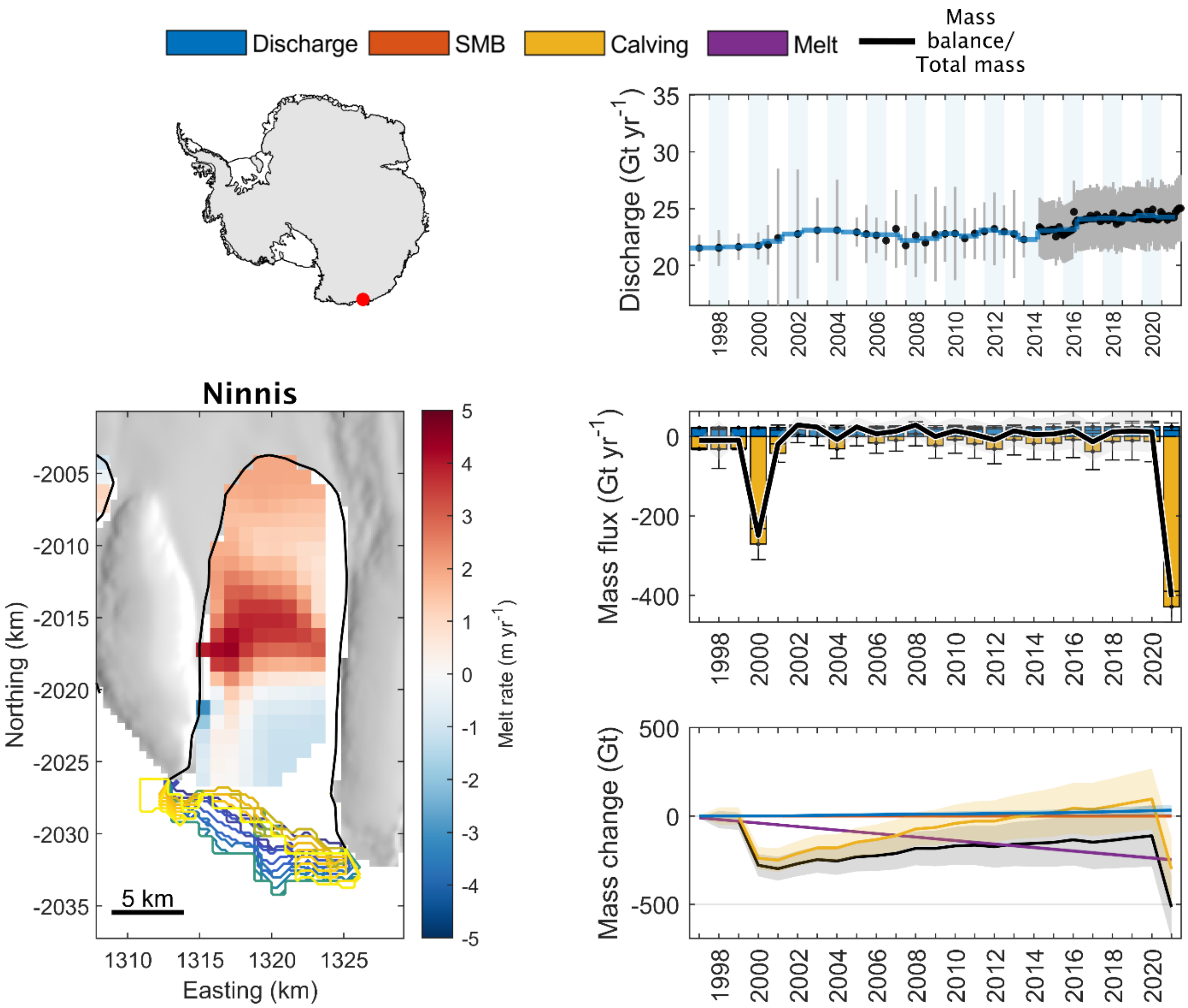
Discharge
SMB
Calving
Melt
Mass balance/ Total mass
Ninnis
Northing (km)
Easting (km)
5 km
Melt rate (m yr^{-1})
Discharge (Gt yr^{-1})
Mass flux (Gt yr^{-1})
Mass change (Gt)

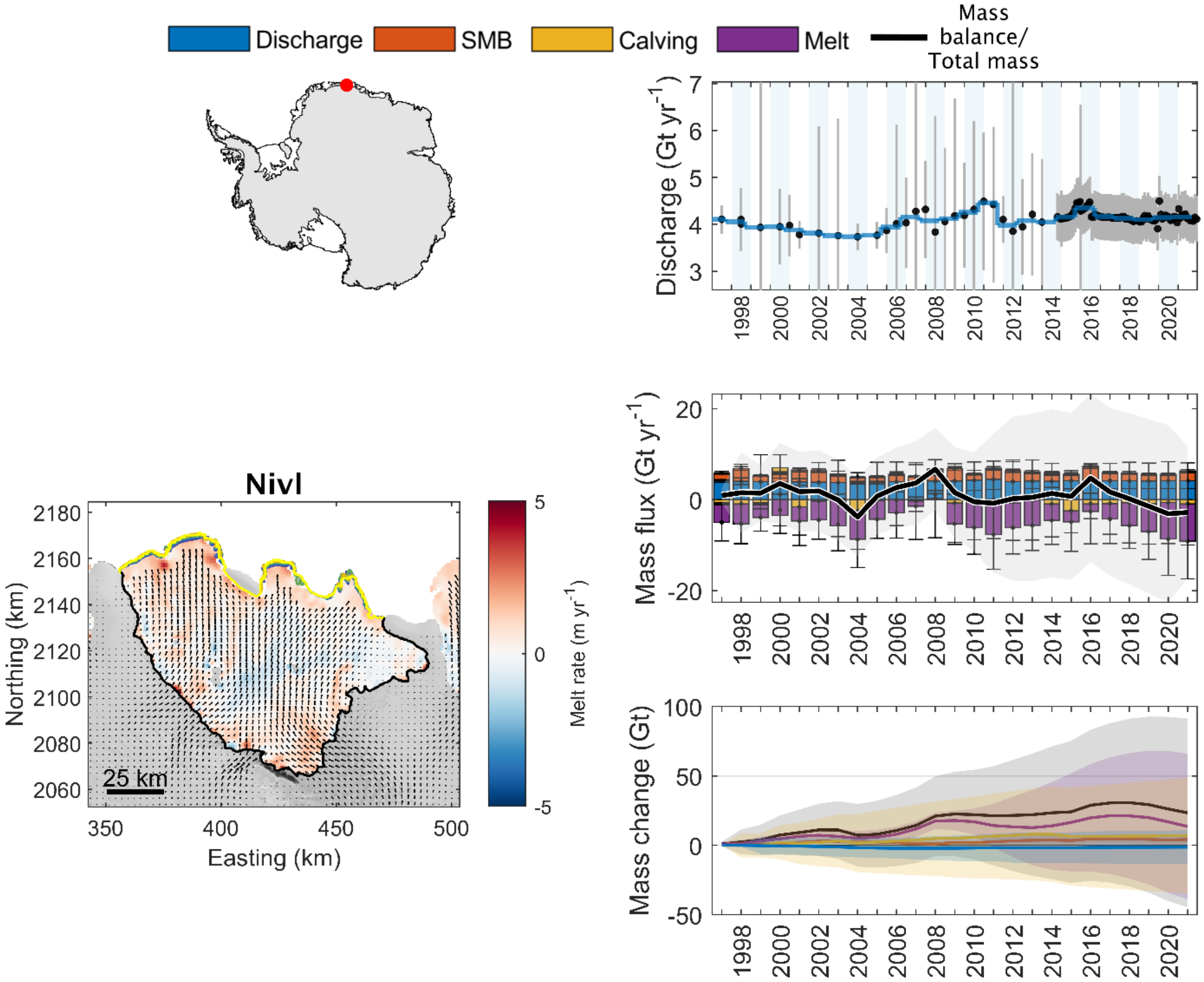

Discharge
SMB
Calving
Melt
Mass balance/ Total mass
Nivl
Northing (km)
Easting (km)
25 km
Melt rate (m yr-1)
Discharge (Gt yr-1)
Mass flux (Gt yr-1)
Mass change (Gt)

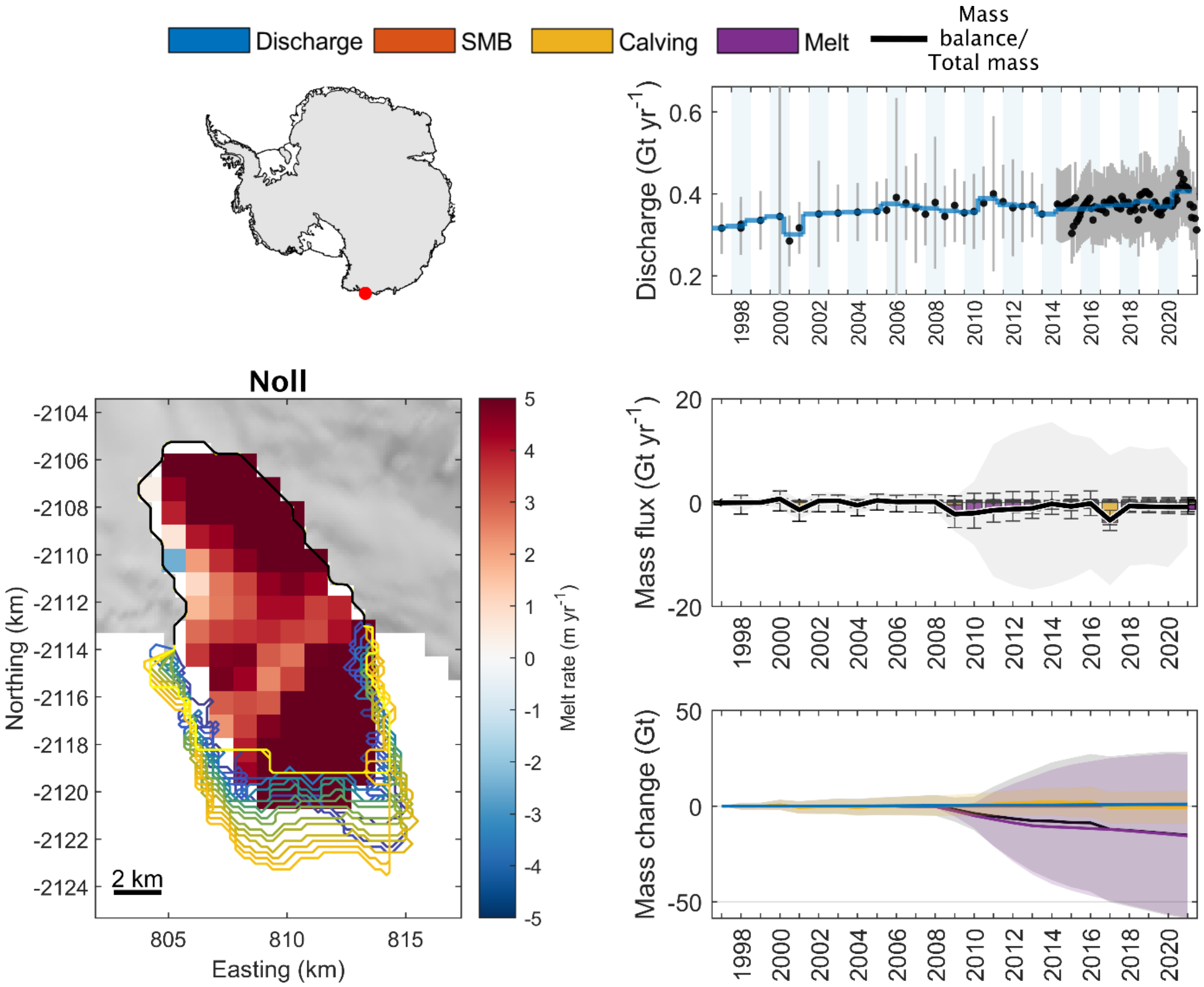
Discharge
SMB
Calving
Melt
Mass balance/ Total mass
Noll
Discharge (Gt yr^{-1})
Mass flux (Gt yr^{-1})
Mass change (Gt)
Northing (km)
Easting (km)
Melt rate (m yr^{-1})
2 km

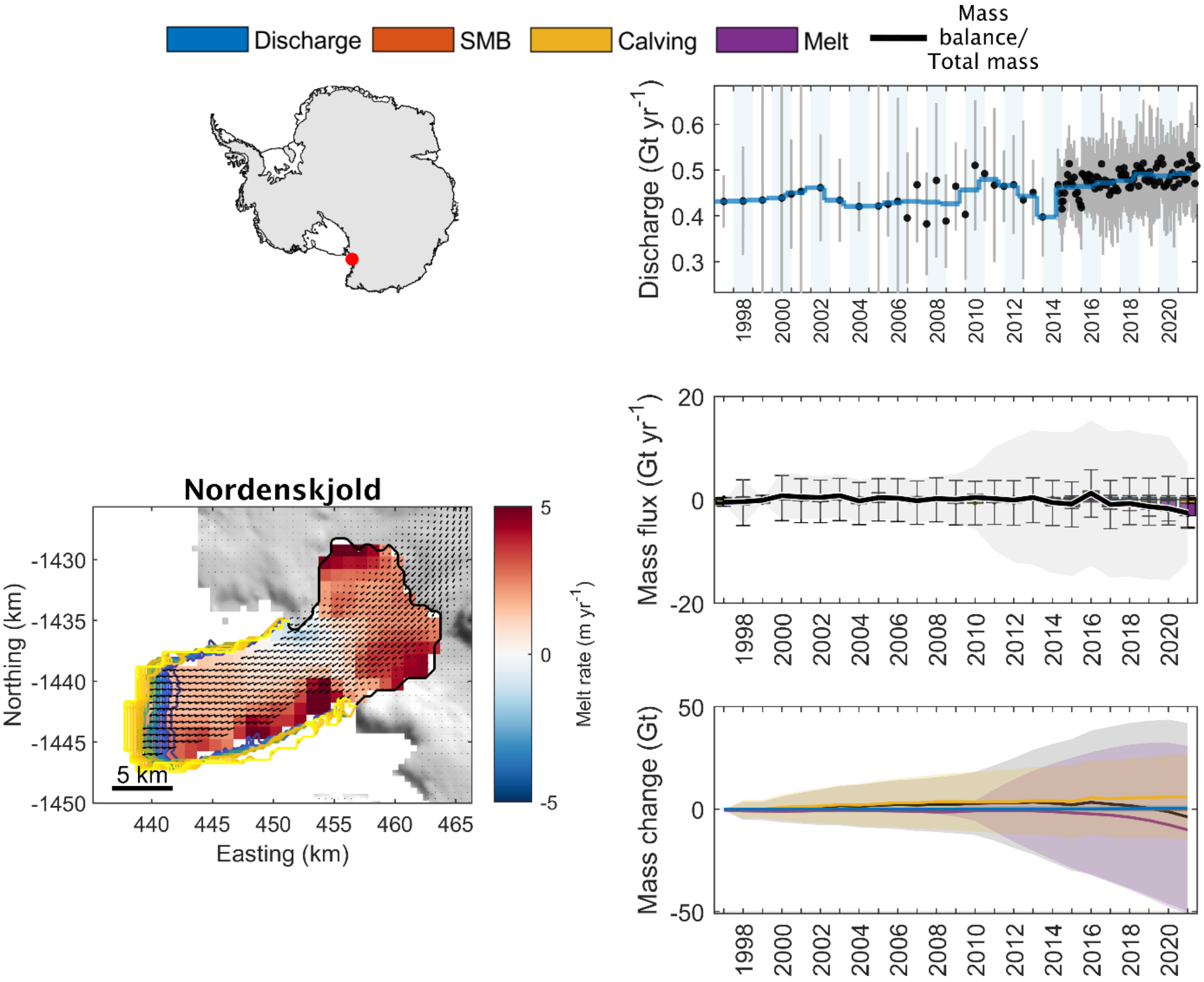

Discharge
SMB
Calving
Melt
Mass balance/ Total mass
Discharge (Gt yr-1)
Nordenskjold
Northing (km)
Easting (km)
5 km
Melt rate (m yr-1)
Mass flux (Gt yr-1)
Mass change (Gt)

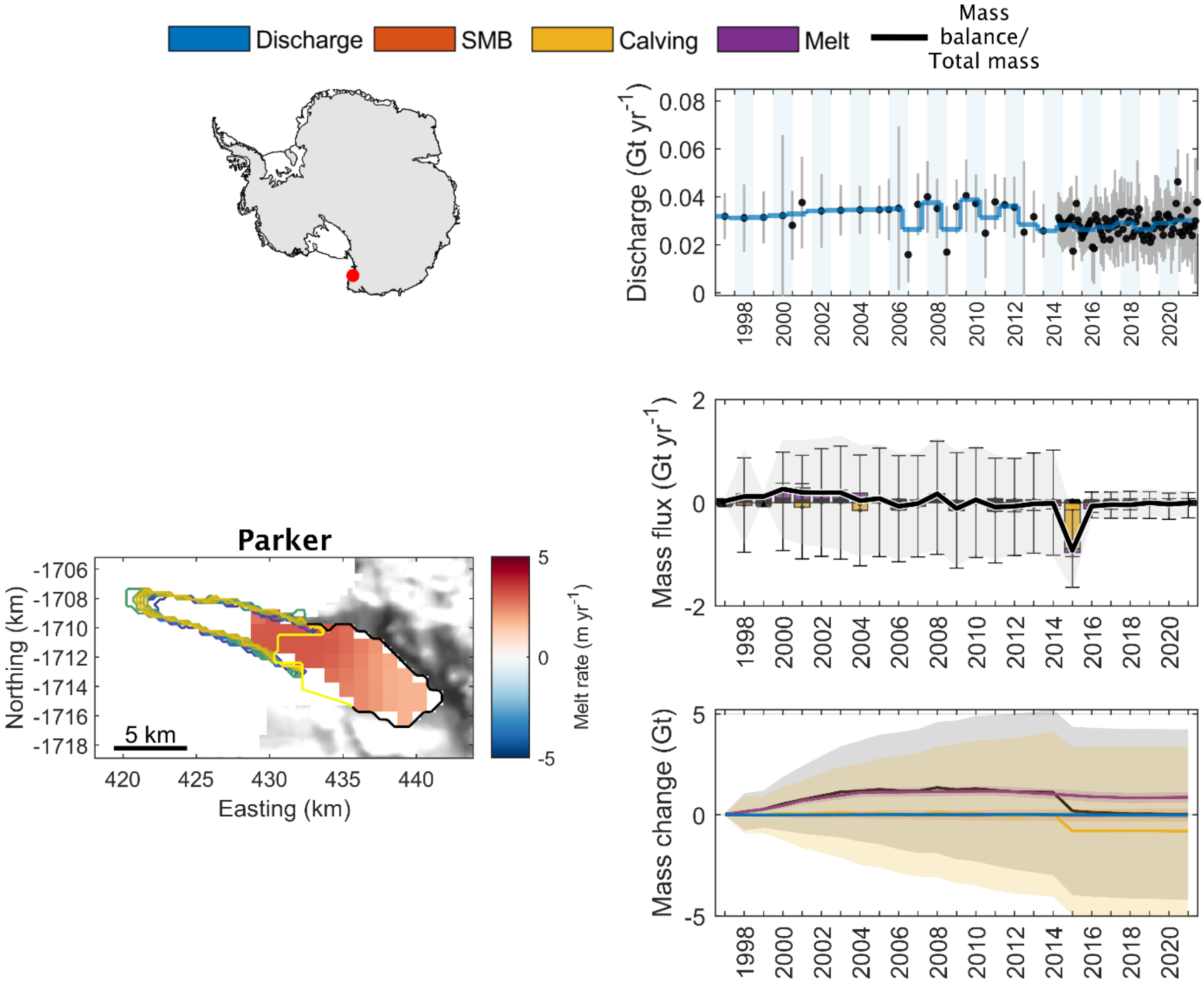
Discharge
SMB
Calving
Melt
Mass balance/ Total mass
Parker
Northing (km)
Easting (km)
5 km
Melt rate (m yr^-1)
Discharge (Gt yr^-1)
Mass flux (Gt yr^-1)
Mass change (Gt)

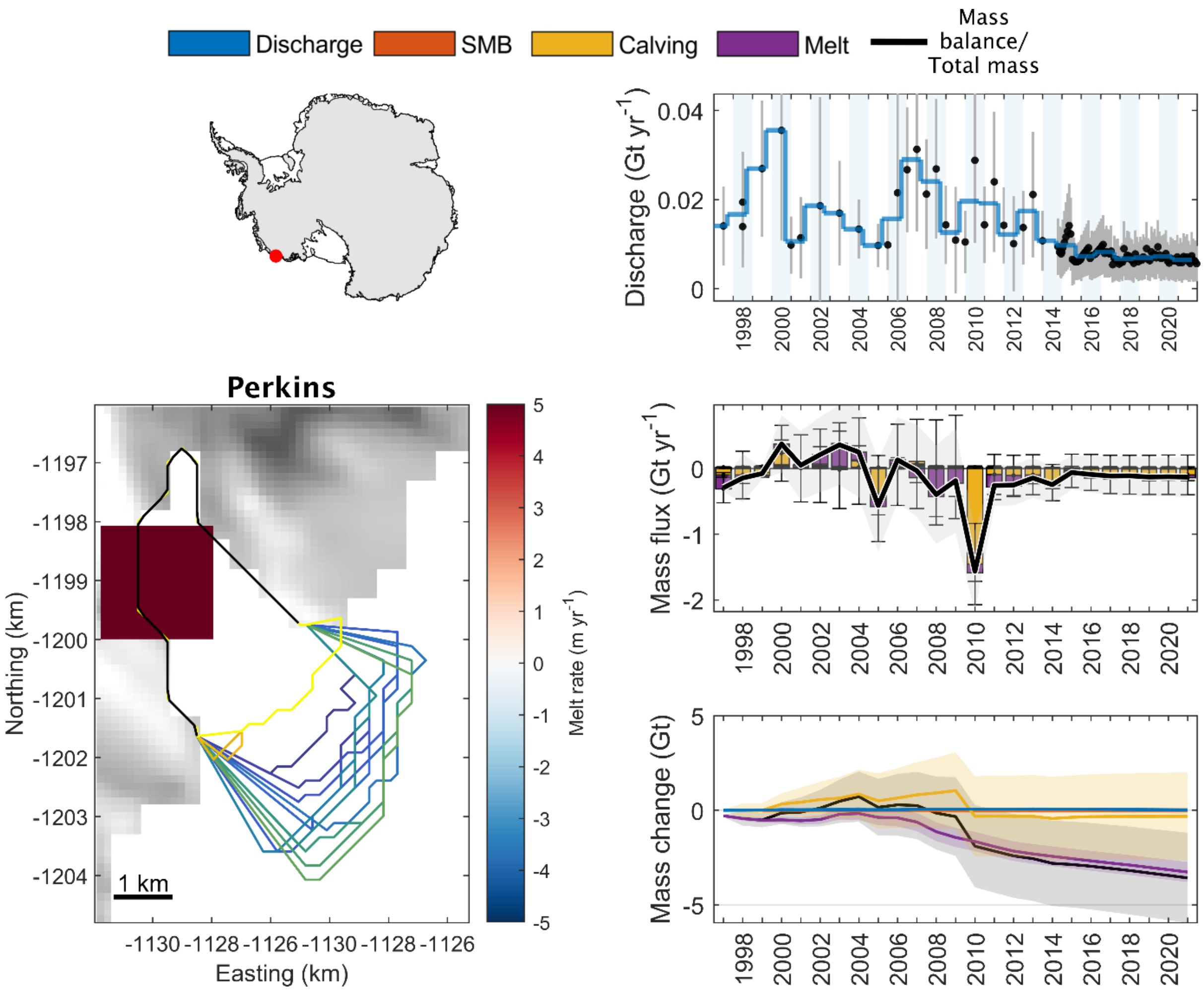

Discharge
SMB
Calving
Melt
Mass balance/ Total mass
Discharge (Gt yr-1)
Perkins
1 km
Northing (km)
Easting (km)
Melt rate (m yr-1)
Mass flux (Gt yr-1)
Mass change (Gt)

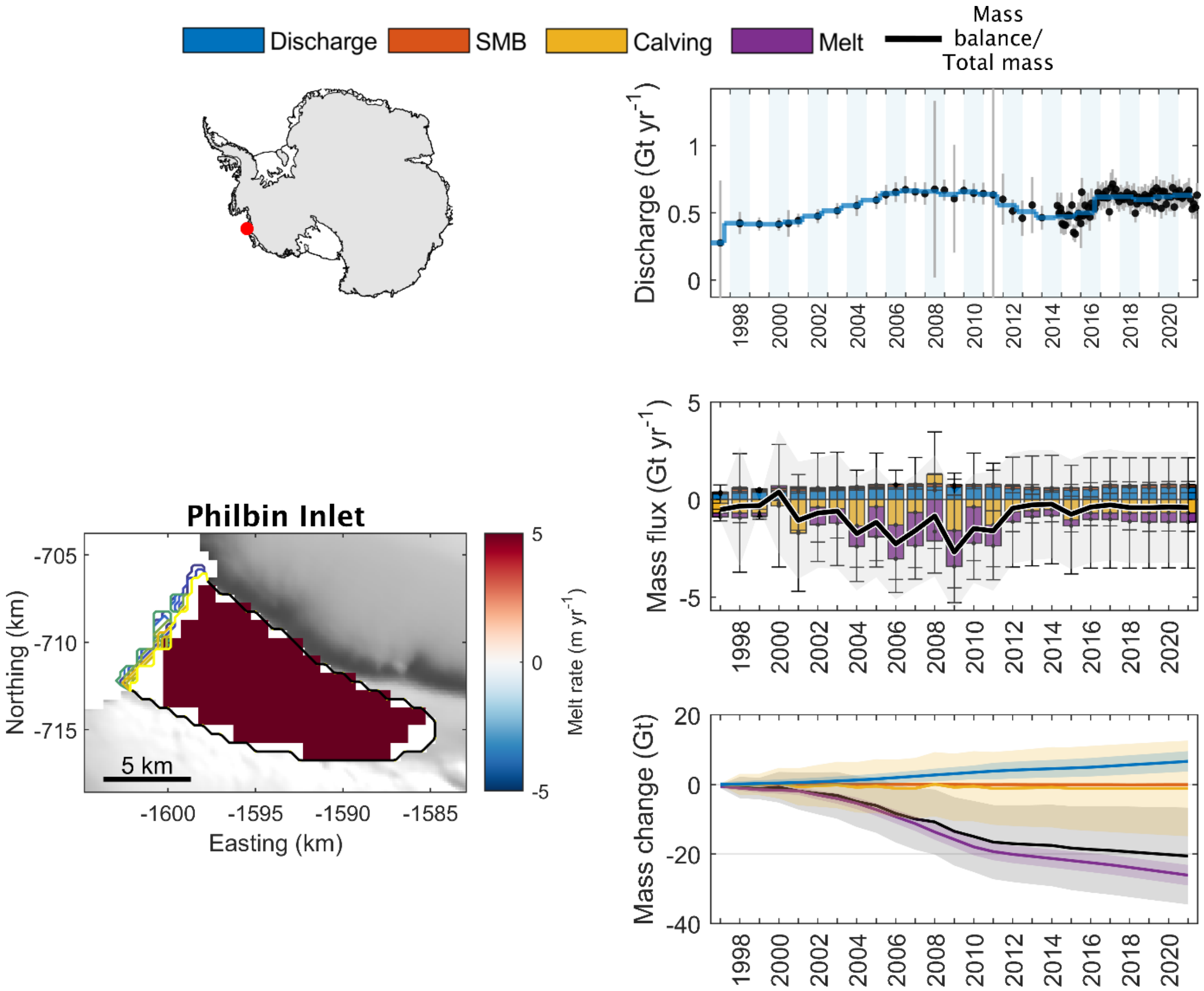
Discharge
SMB
Calving
Melt
Mass balance/ Total mass
Philbin Inlet
Northing (km)
Easting (km)
5 km
Melt rate (m yr-1)
Discharge (Gt yr-1)
Mass flux (Gt yr-1)
Mass change (Gt)

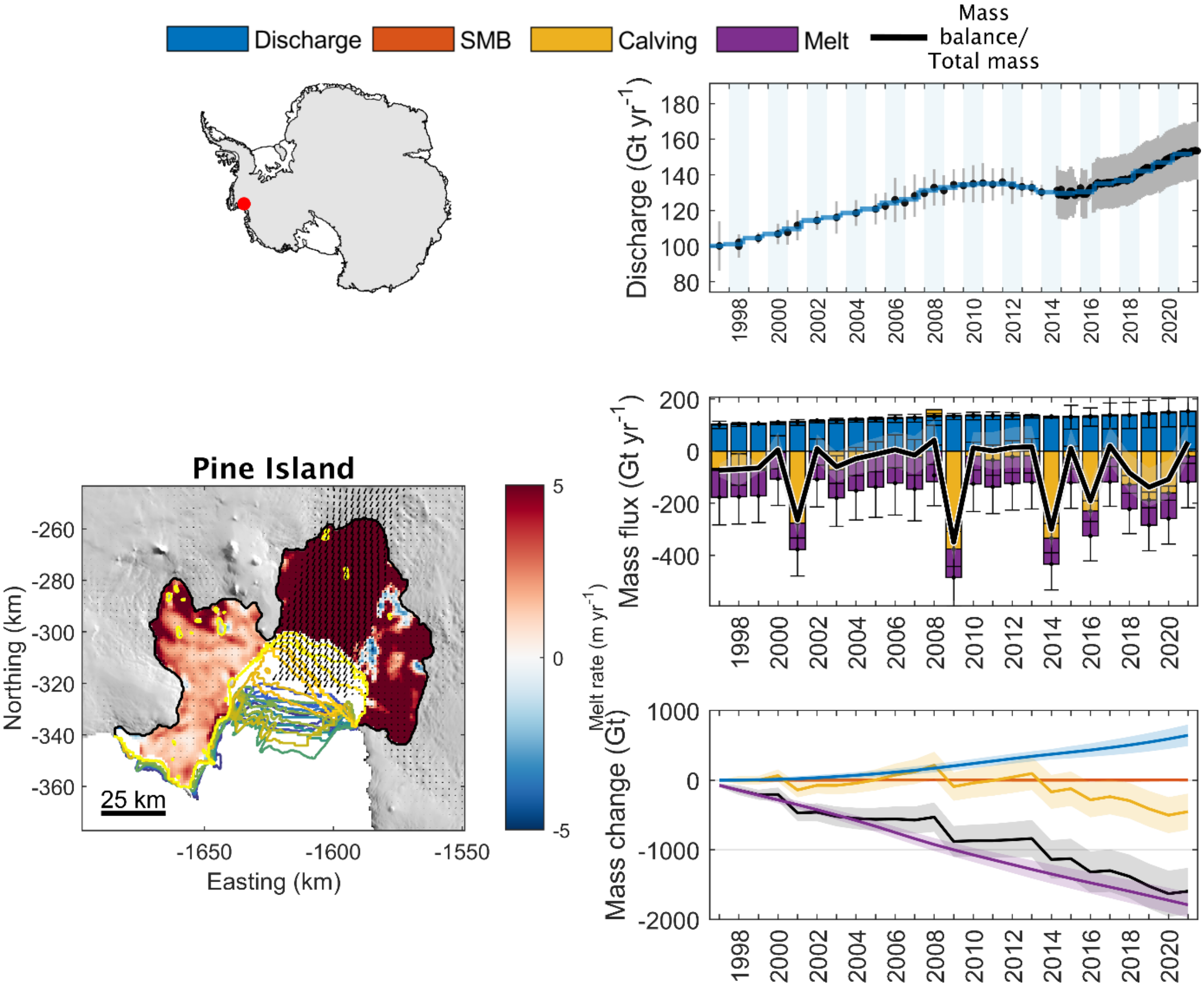
Discharge
SMB
Calving
Melt
Mass balance/ Total mass
Pine Island
Northing (km)
Easting (km)
25 km
-1650
-1600
-1550
-260
-280
-300
-320
-340
-360
Melt rate (m yr-1)
5
0
-5
Discharge (Gt yr-1)
Mass flux (Gt yr-1)
Mass change (Gt)
1998
2000
2002
2004
2006
2008
2010
2012
2014
2016
2018
2020

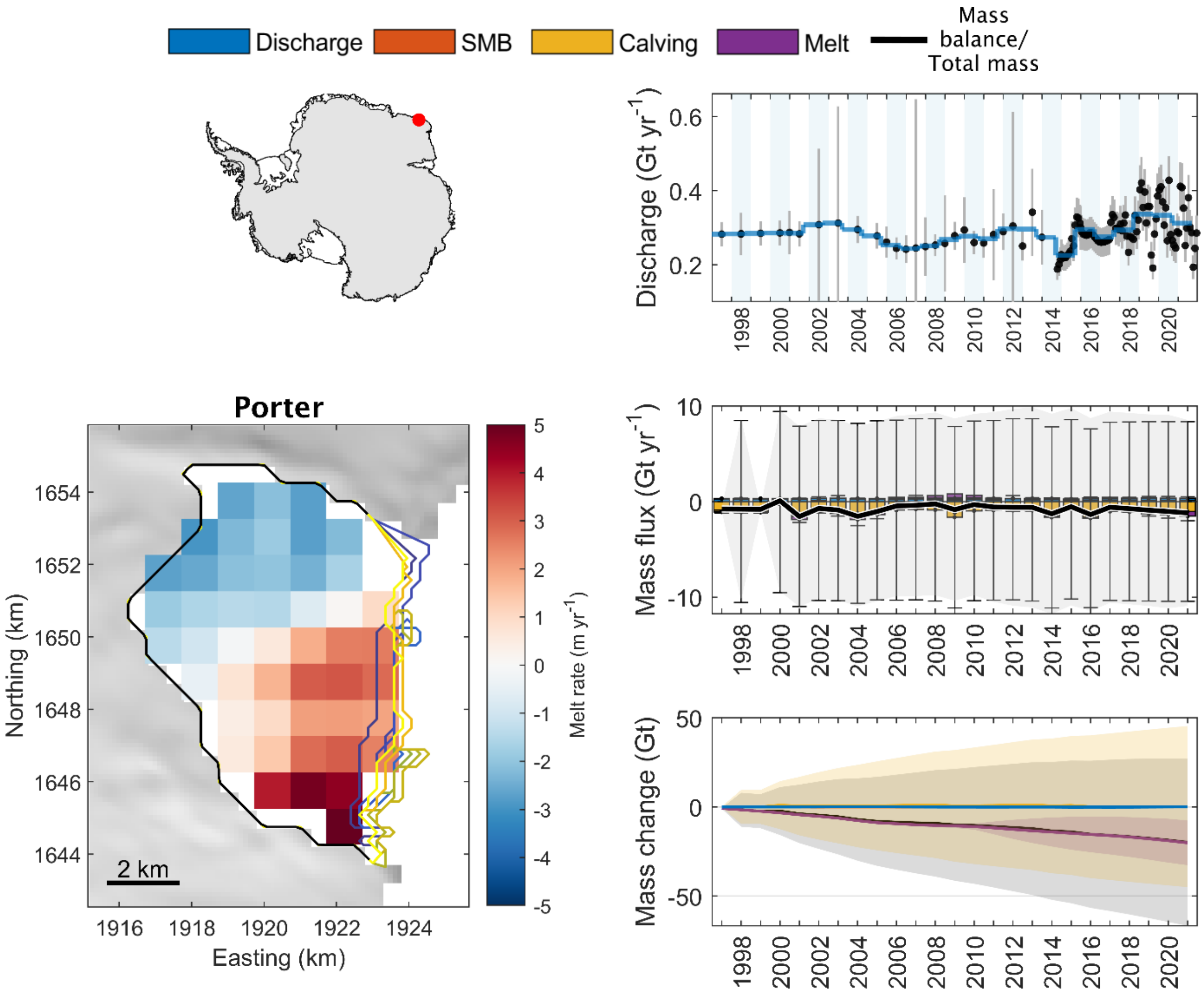

Discharge
SMB
Calving
Melt
Mass balance/ Total mass
Porter
Northing (km)
Easting (km)
2 km
Melt rate (m yr-1)
Discharge (Gt yr-1)
Mass flux (Gt yr-1)
Mass change (Gt)

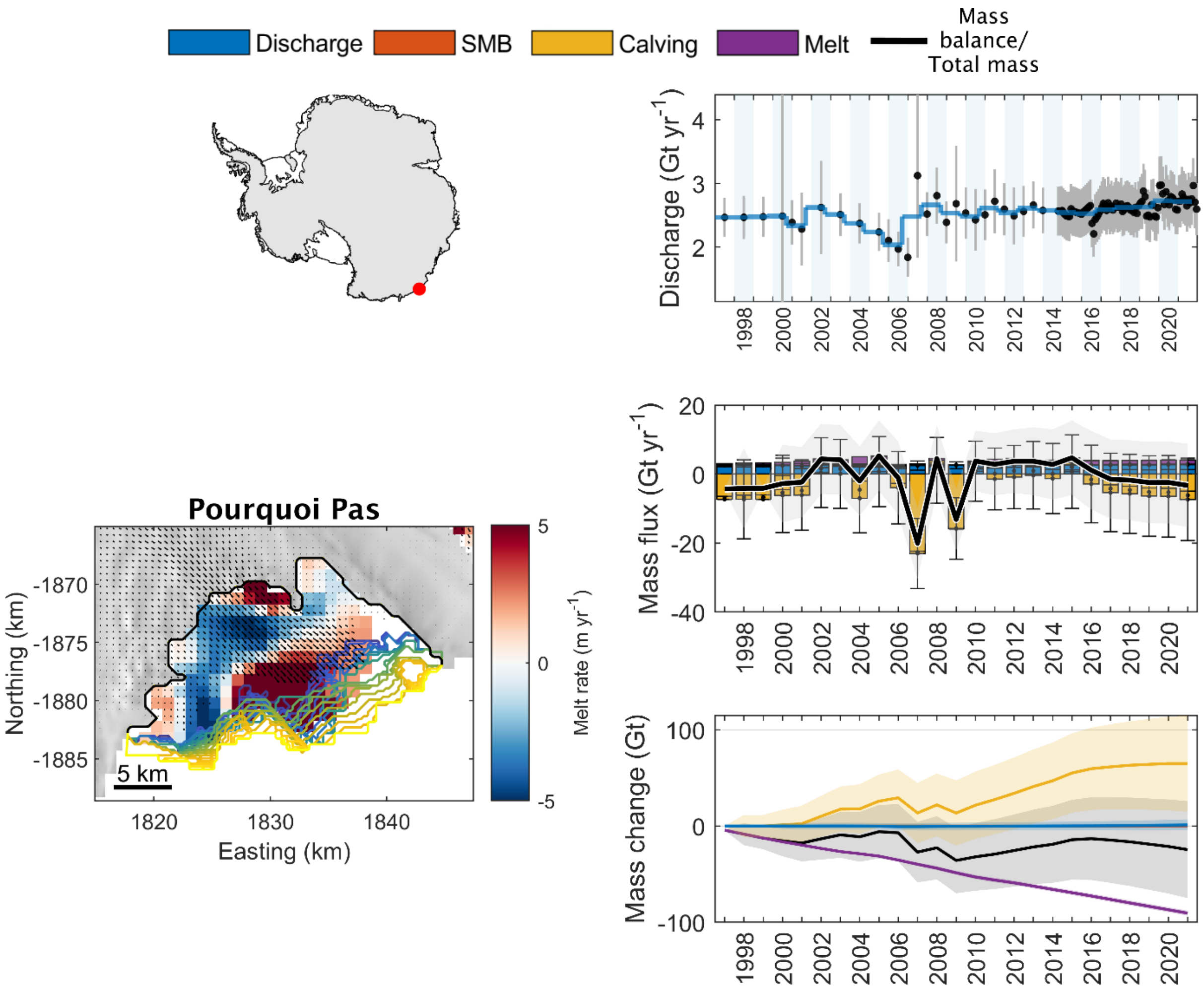
Discharge
SMB
Calving
Melt
Mass balance/ Total mass
Pourquoi Pas
Northing (km)
-1870
-1875
-1880
-1885
5 km
1820
1830
1840
Easting (km)
Melt rate (m yr-1)
5
0
-5
Discharge (Gt yr-1)
Mass flux (Gt yr-1)
Mass change (Gt)
1998
2000
2002
2004
2006
2008
2010
2012
2014
2016
2018
2020

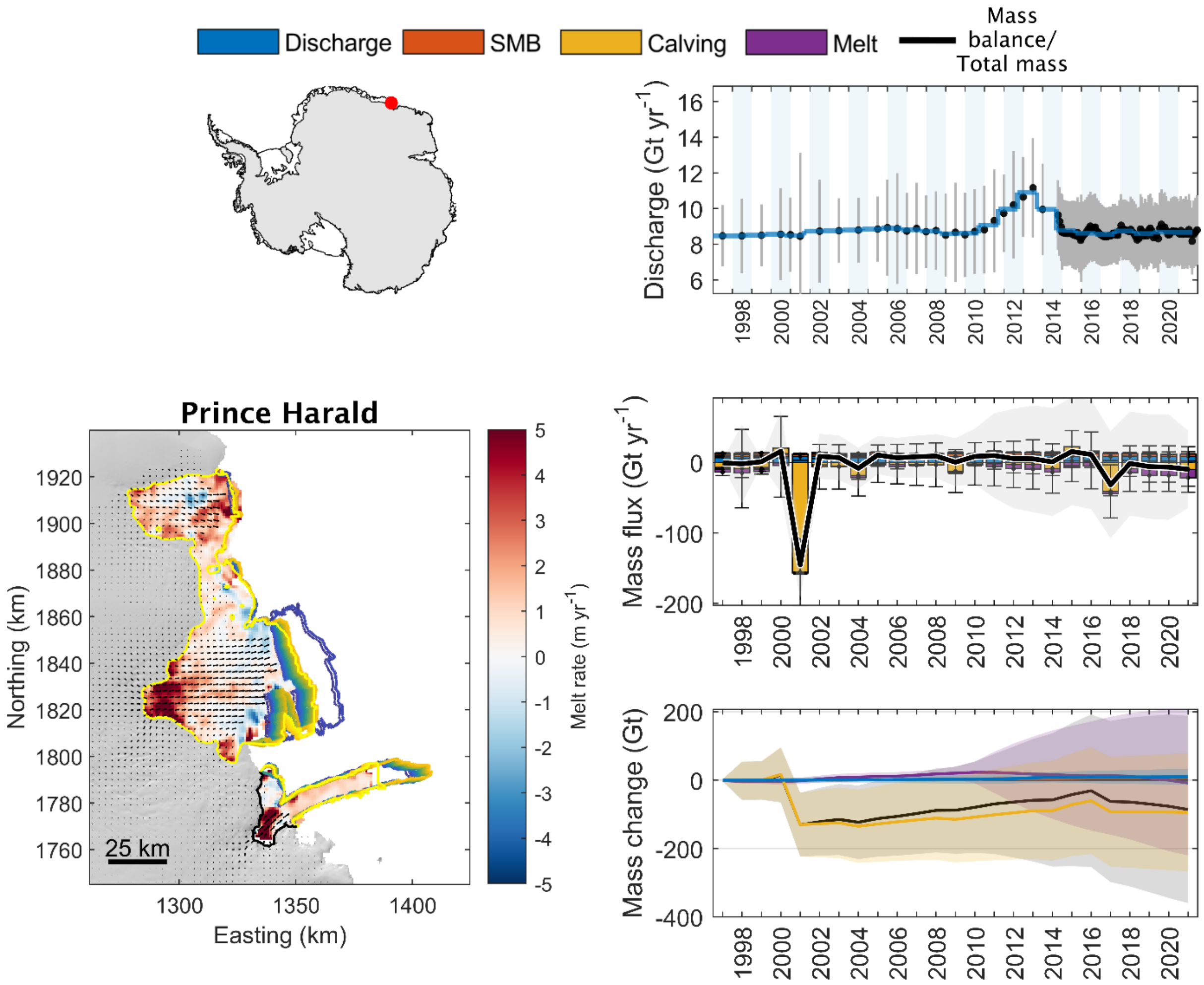
Discharge
SMB
Calving
Melt
Mass balance/ Total mass
Discharge (Gt yr-1)
Prince Harald
Northing (km)
Easting (km)
25 km
Melt rate (m yr-1)
Mass flux (Gt yr-1)
Mass change (Gt)

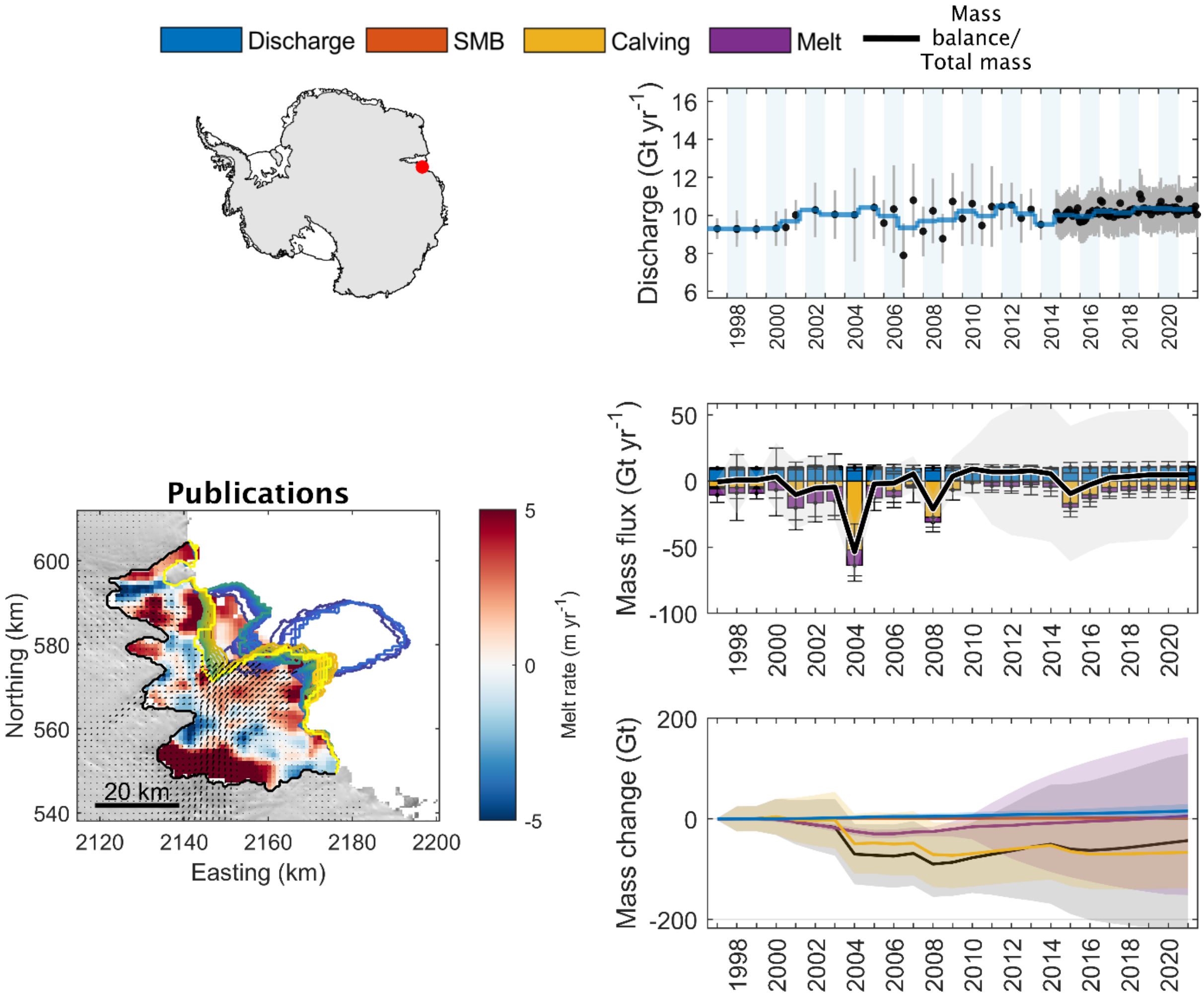
Discharge
SMB
Calving
Melt
Mass balance/ Total mass
Discharge (Gt yr^{-1})
Mass flux (Gt yr^{-1})
Mass change (Gt)
Publications
Northing (km)
Easting (km)
20 km
Melt rate (m yr^{-1})

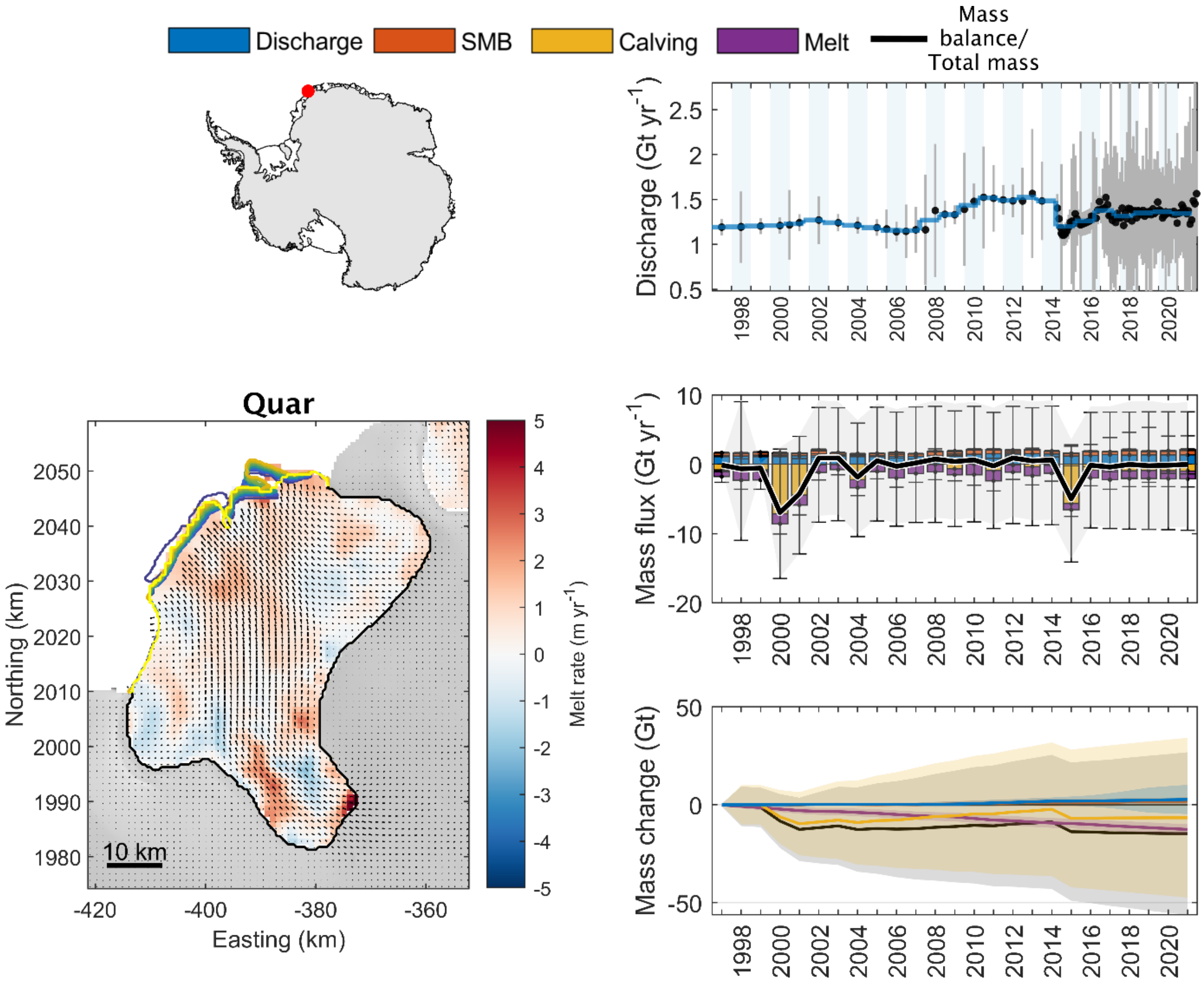
Discharge
SMB
Calving
Melt
Mass balance/ Total mass
Quar
Northing (km)
Easting (km)
10 km
Melt rate (m yr^{-1})
Discharge (Gt yr^{-1})
Mass flux (Gt yr^{-1})
Mass change (Gt)

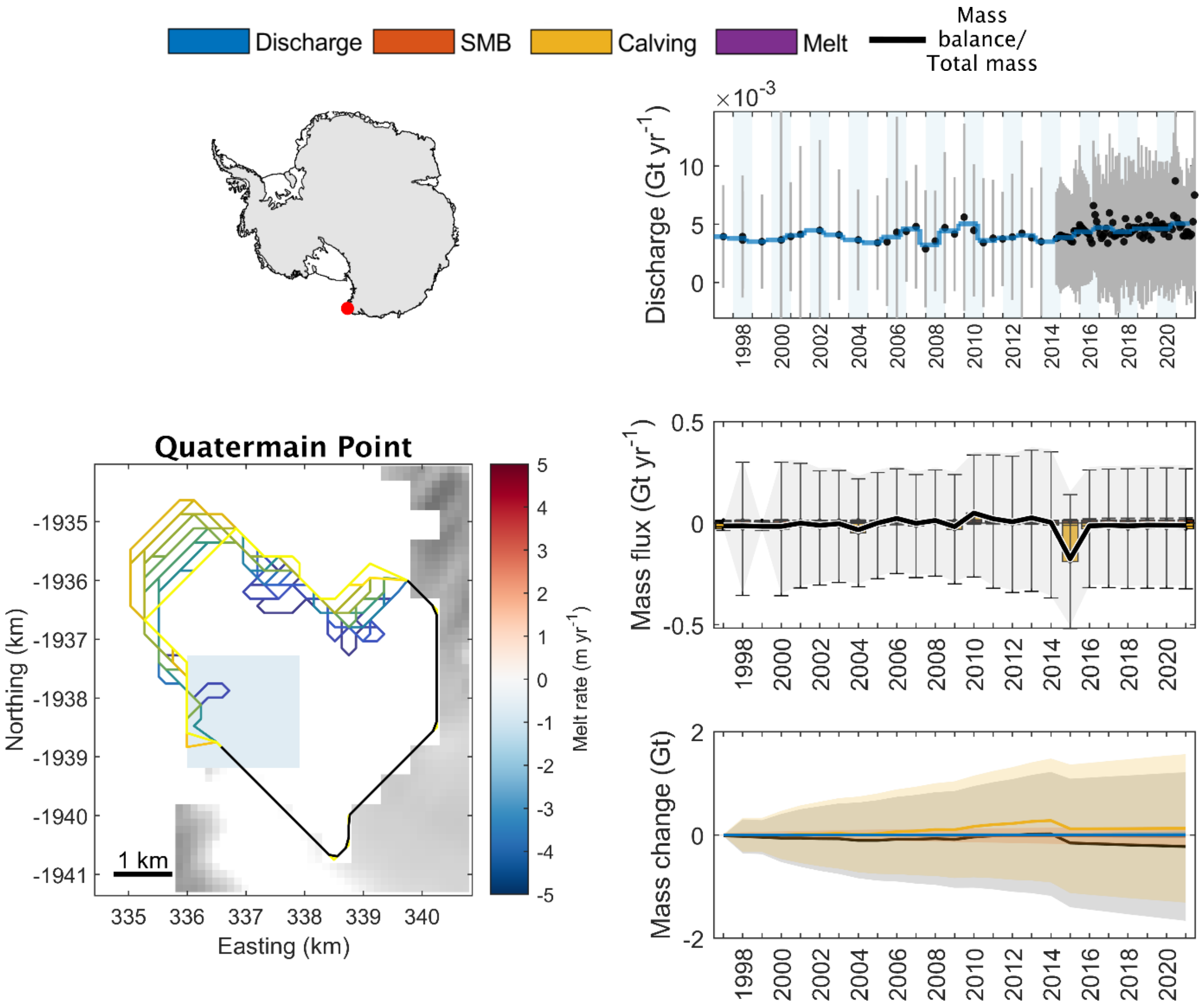
Discharge
SMB
Calving
Melt
Mass balance/ Total mass
Quatermain Point
Northing (km)
Easting (km)
1 km
Melt rate (m yr-1)
Discharge (Gt yr-1)
×10-3
Mass flux (Gt yr-1)
Mass change (Gt)

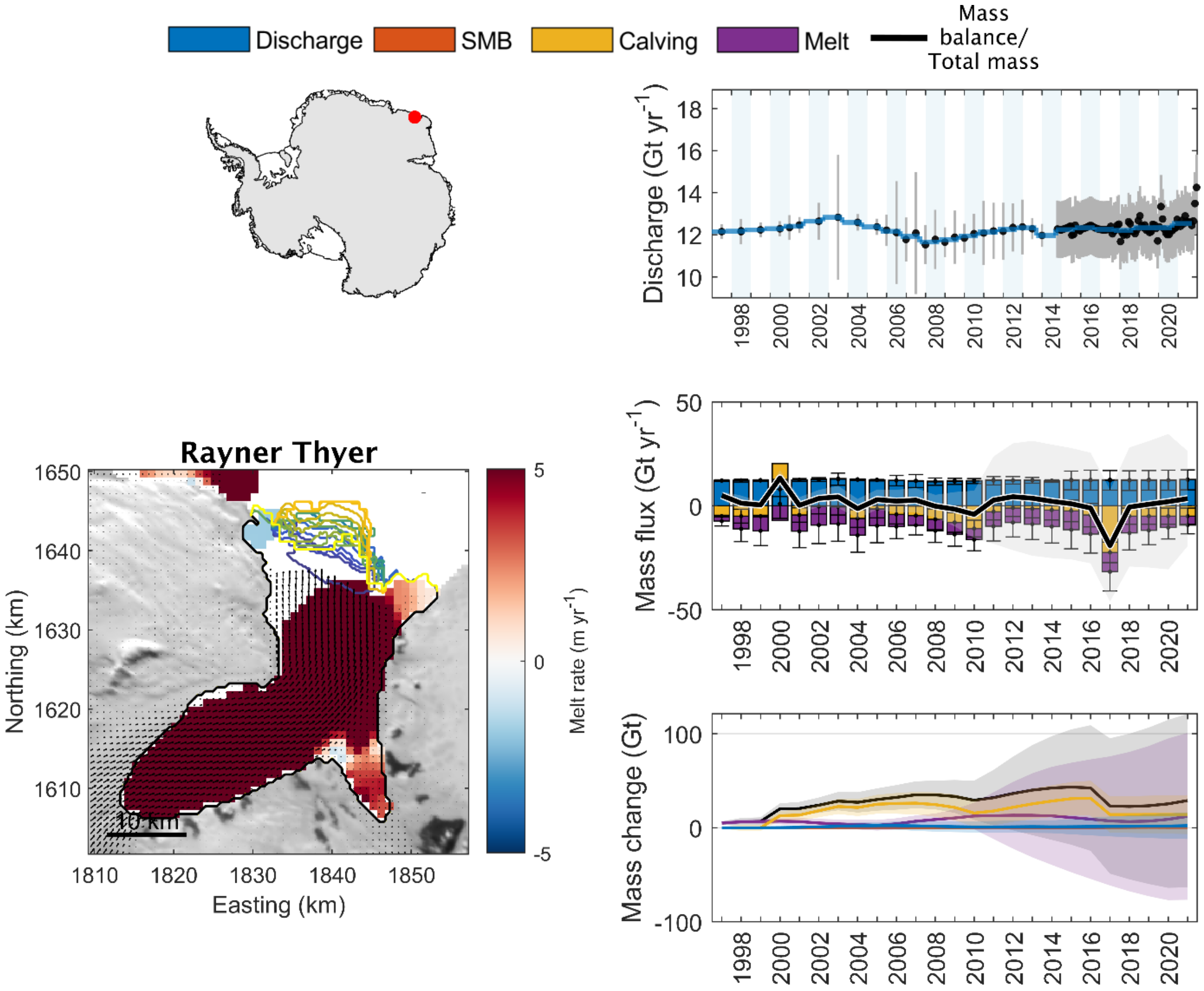

Discharge
SMB
Calving
Melt
Mass balance/ Total mass
Rayner Thyer
Northing (km)
Easting (km)
Melt rate (m yr^{-1})
10 km
Discharge (Gt yr^{-1})
Mass flux (Gt yr^{-1})
Mass change (Gt)

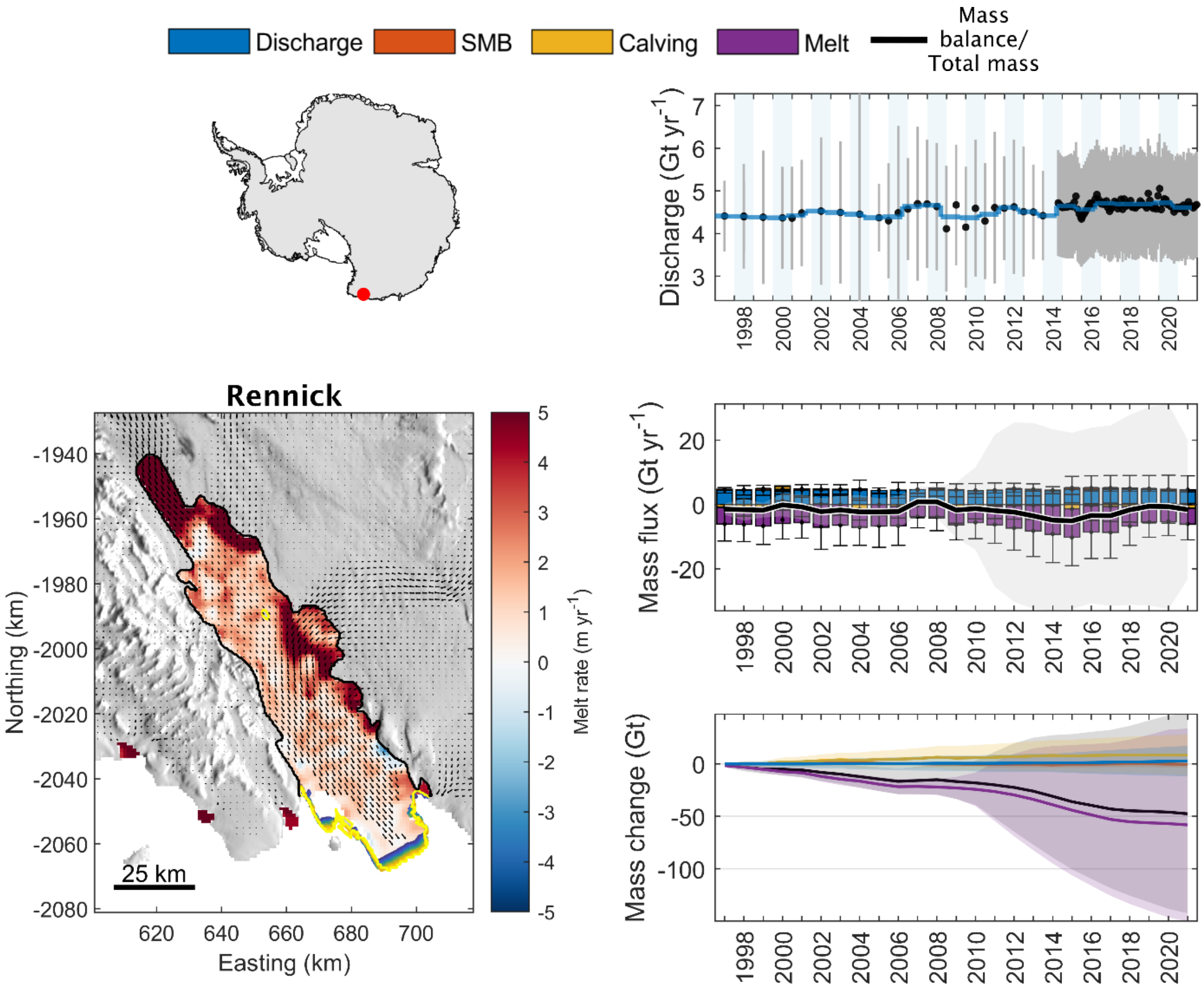
Discharge
SMB
Calving
Melt
Mass balance/ Total mass
Discharge (Gt yr^{-1})
Rennick
Northing (km)
Easting (km)
25 km
Melt rate (m yr^{-1})
Mass flux (Gt yr^{-1})
Mass change (Gt)

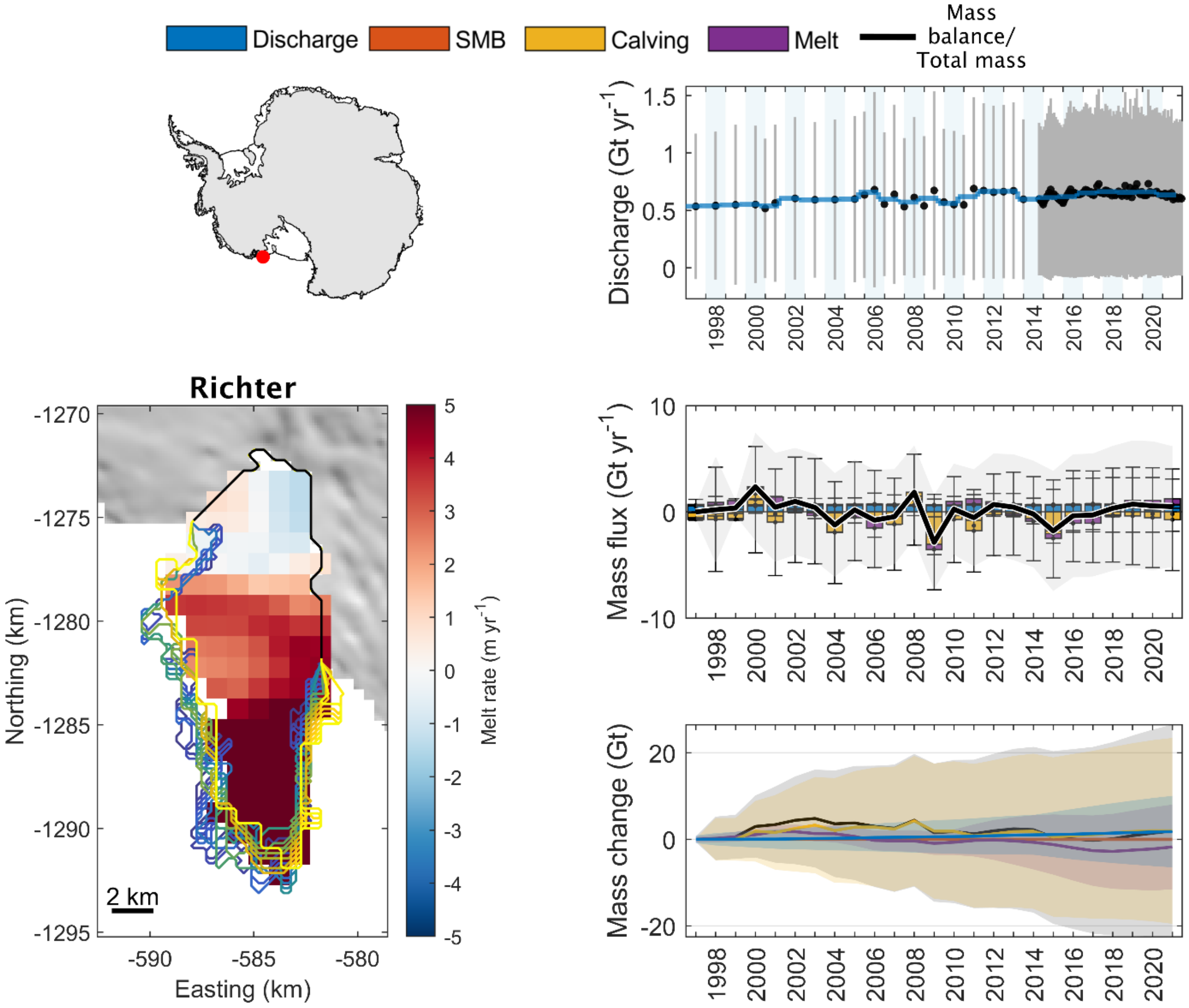
Discharge
SMB
Calving
Melt
Mass balance/ Total mass
Richter
Northing (km)
Easting (km)
Melt rate (m yr^{-1})
2 km
Discharge (Gt yr^{-1})
Mass flux (Gt yr^{-1})
Mass change (Gt)

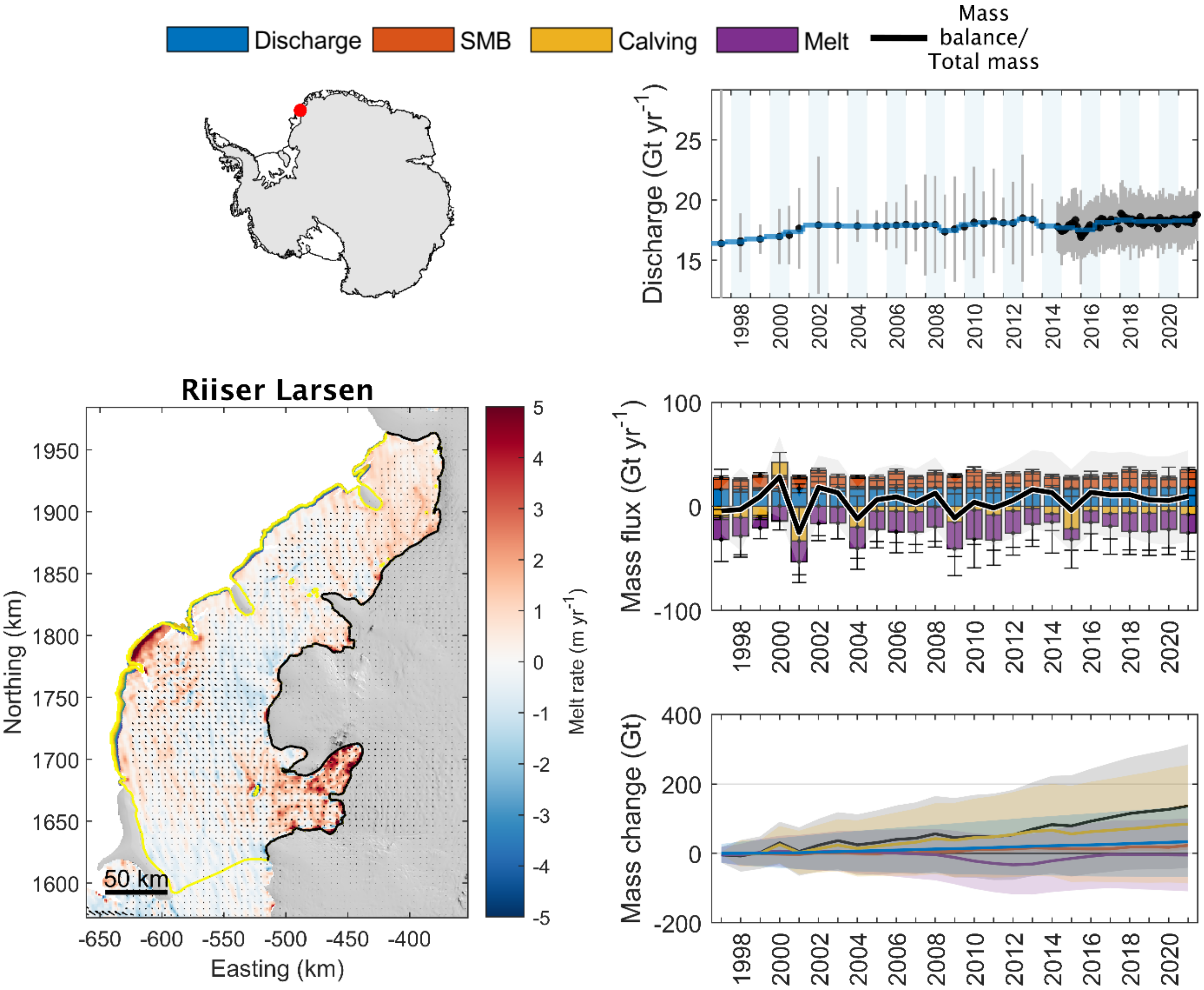
Discharge
SMB
Calving
Melt
Mass balance/ Total mass
Riiser Larsen
50 km
Northing (km)
Easting (km)
Melt rate (m yr^{-1})
Discharge (Gt yr^{-1})
Mass flux (Gt yr^{-1})
Mass change (Gt)

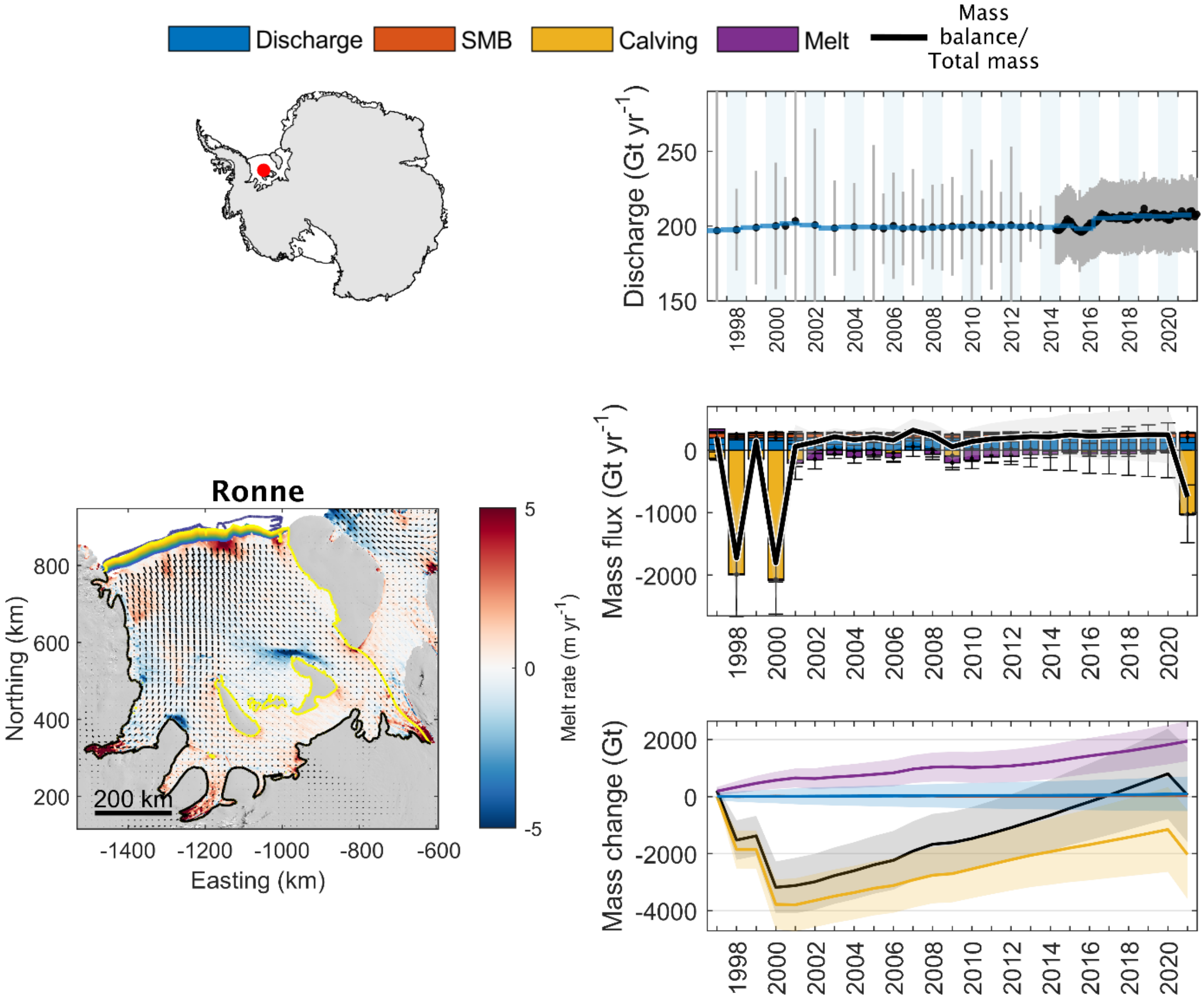

Discharge
SMB
Calving
Melt
Mass balance/ Total mass
Ronne
Northing (km)
Easting (km)
200 km
Melt rate (m yr-1)
Discharge (Gt yr-1)
Mass flux (Gt yr-1)
Mass change (Gt)

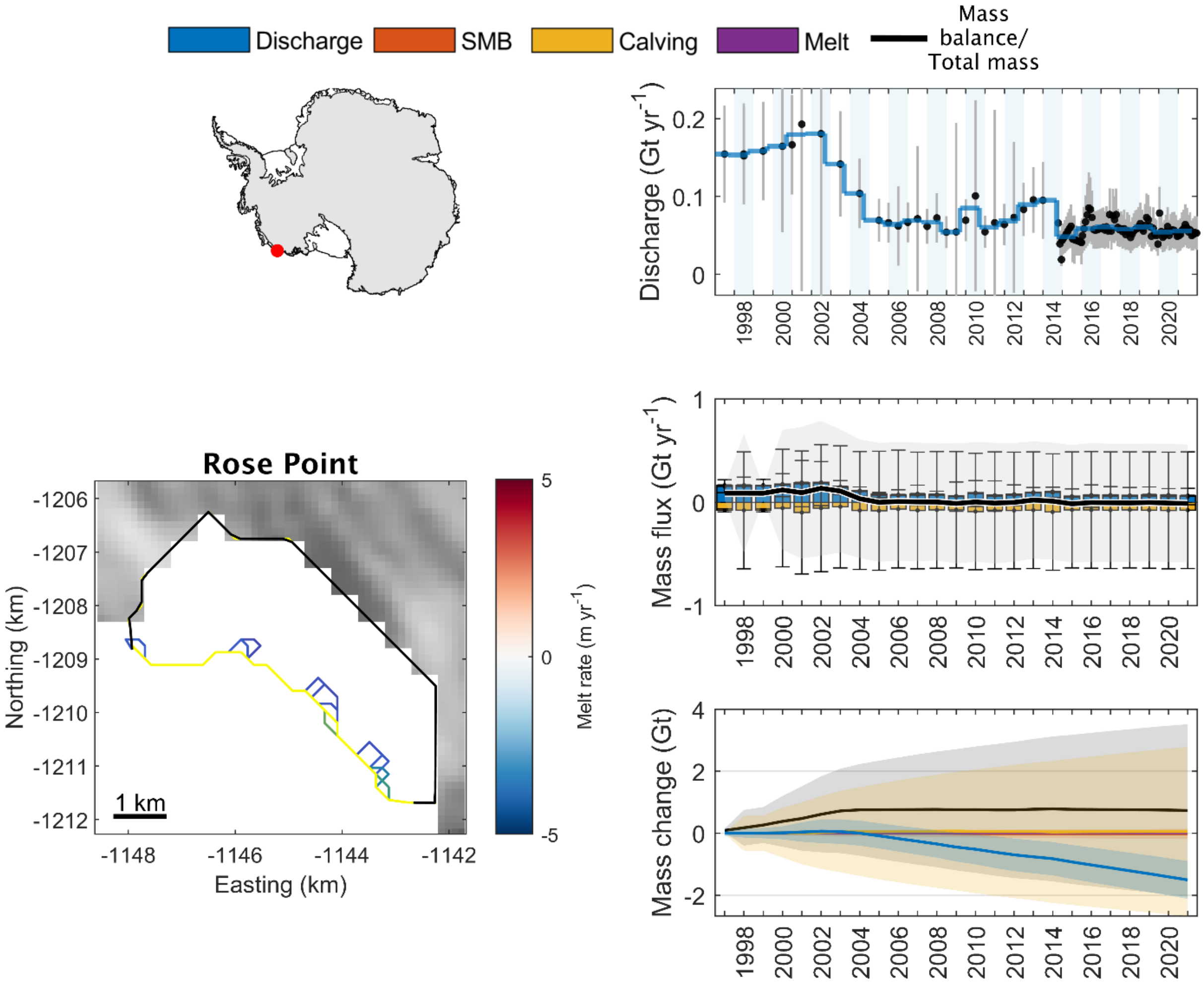

Discharge
SMB
Calving
Melt
Mass balance/ Total mass
Rose Point
Northing (km)
Easting (km)
1 km
Melt rate (m yr^{-1})
Discharge (Gt yr^{-1})
Mass flux (Gt yr^{-1})
Mass change (Gt)

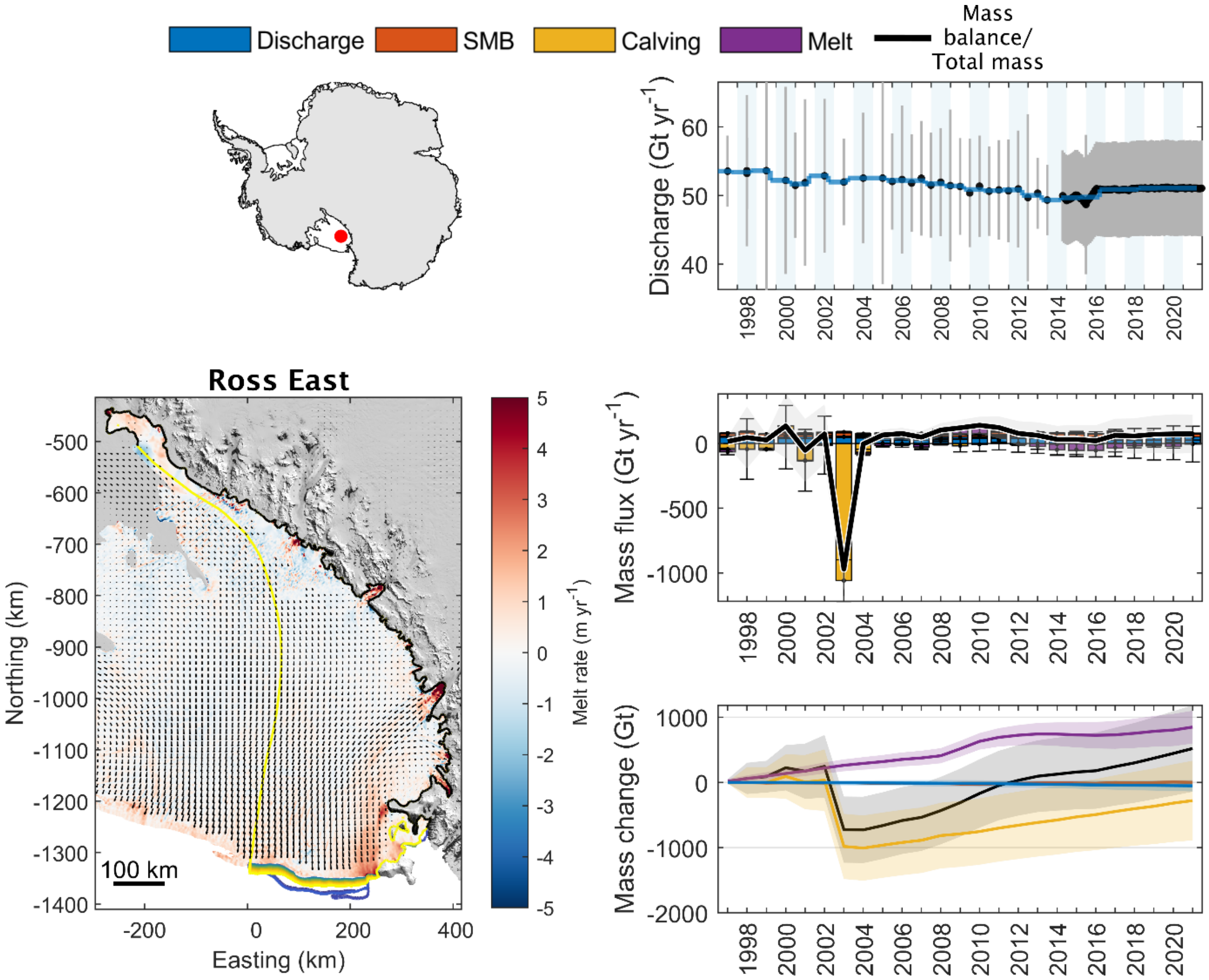

Discharge
SMB
Calving
Melt
Mass balance/ Total mass
Discharge (Gt yr^{-1})
Ross East
Northing (km)
Easting (km)
100 km
Melt rate (m yr^{-1})
Mass flux (Gt yr^{-1})
Mass change (Gt)

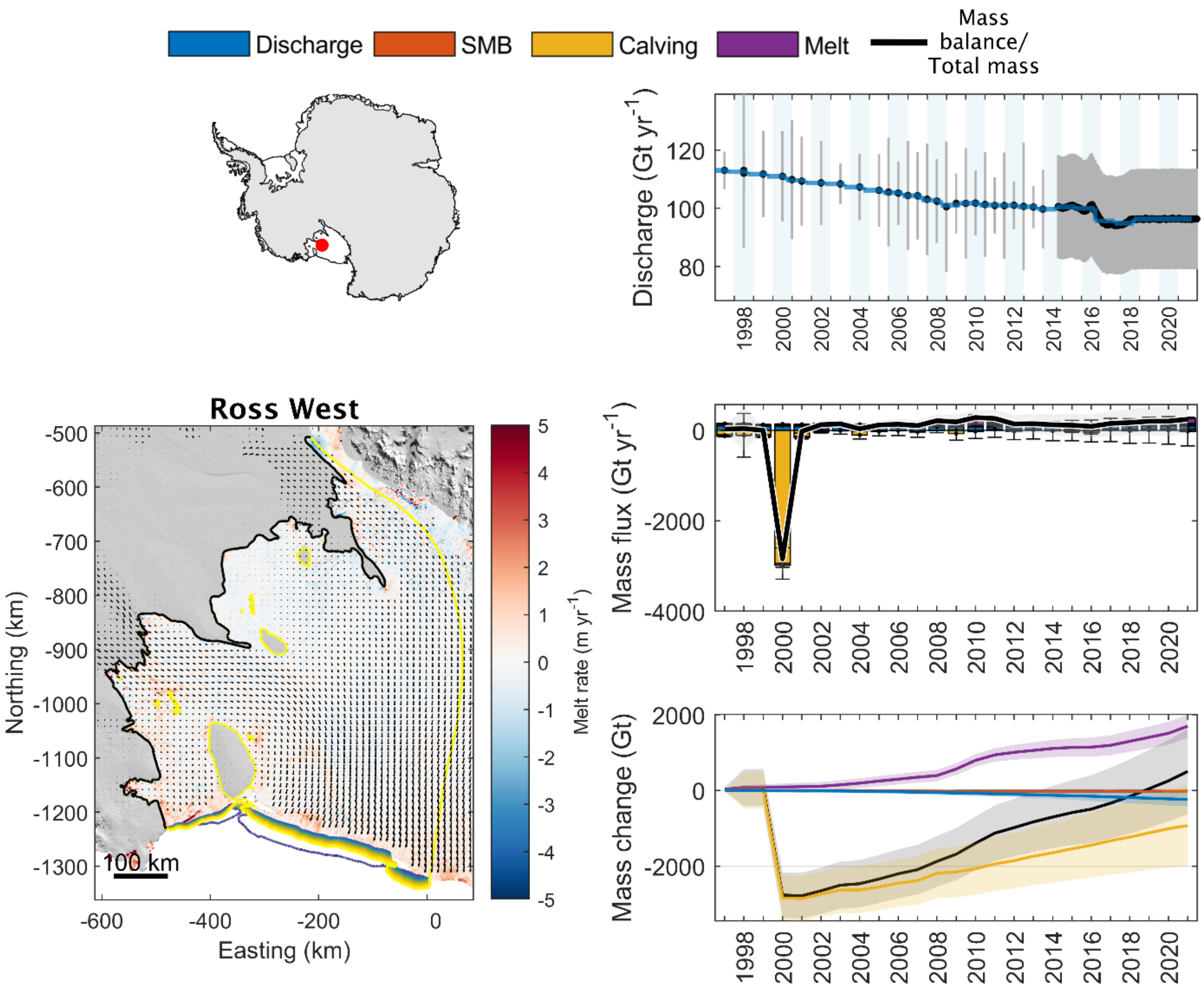

Discharge
SMB
Calving
Melt
Mass balance/ Total mass
Ross West
Northing (km)
Easting (km)
100 km
Melt rate (m yr^{-1})
Discharge (Gt yr^{-1})
Mass flux (Gt yr^{-1})
Mass change (Gt)

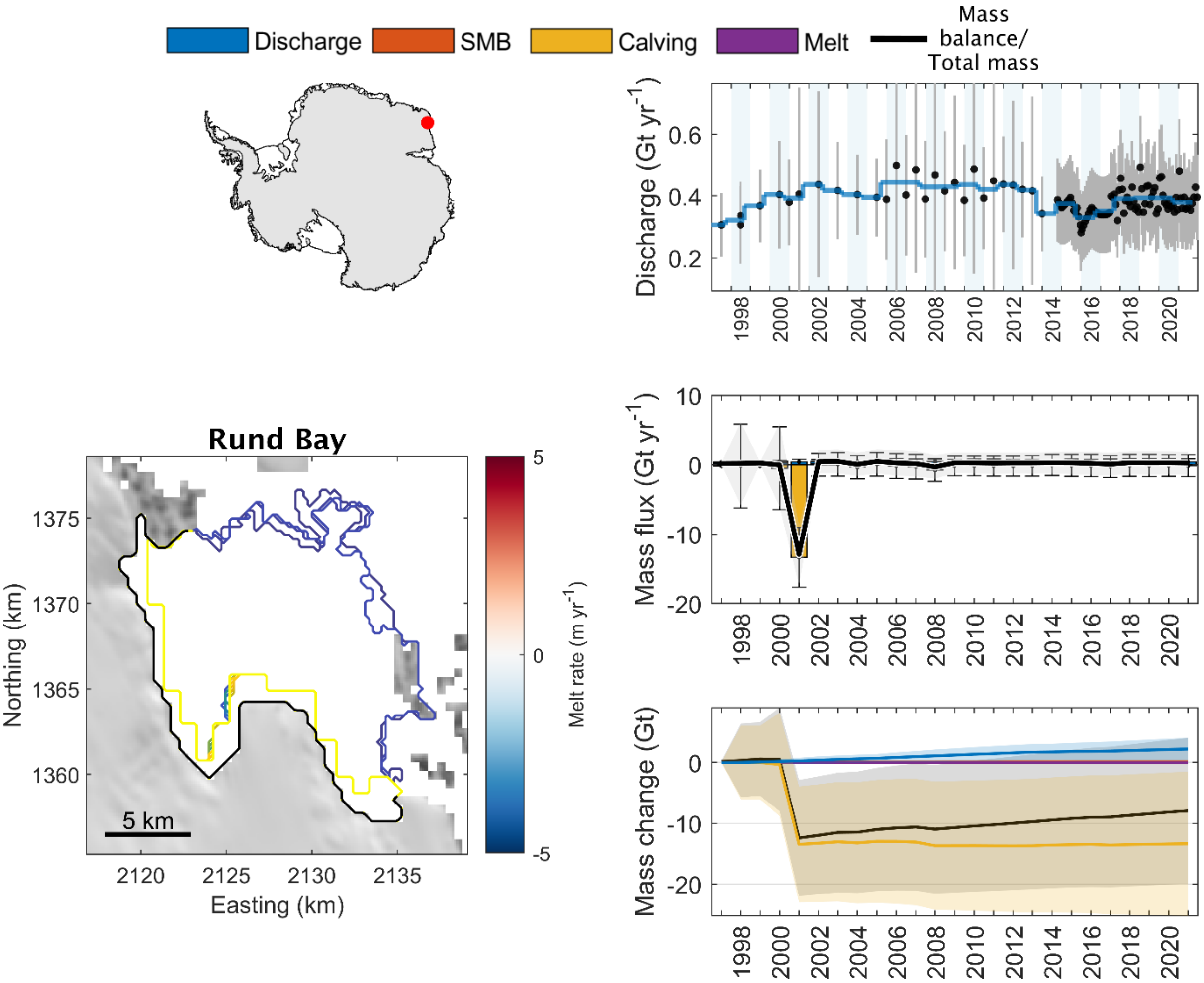

Discharge
SMB
Calving
Melt
Mass balance/ Total mass
Rund Bay
Northing (km)
Easting (km)
5 km
Melt rate (m yr-1)
Discharge (Gt yr-1)
Mass flux (Gt yr-1)
Mass change (Gt)

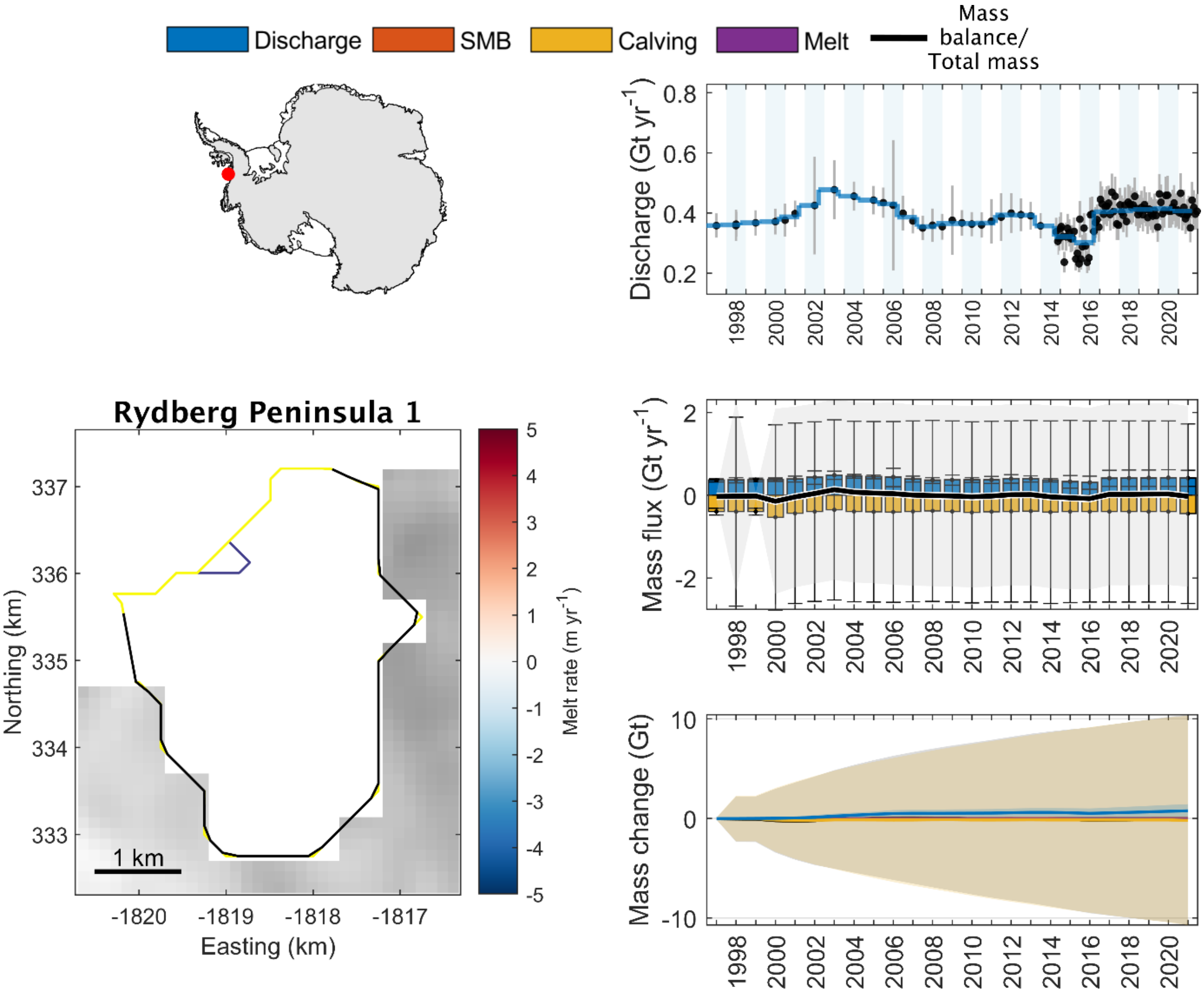
Discharge
SMB
Calving
Melt
Mass balance/ Total mass
Rydberg Peninsula 1
Discharge (Gt yr^{-1})
Mass flux (Gt yr^{-1})
Mass change (Gt)
Northing (km)
Easting (km)
Melt rate (m yr^{-1})
1 km

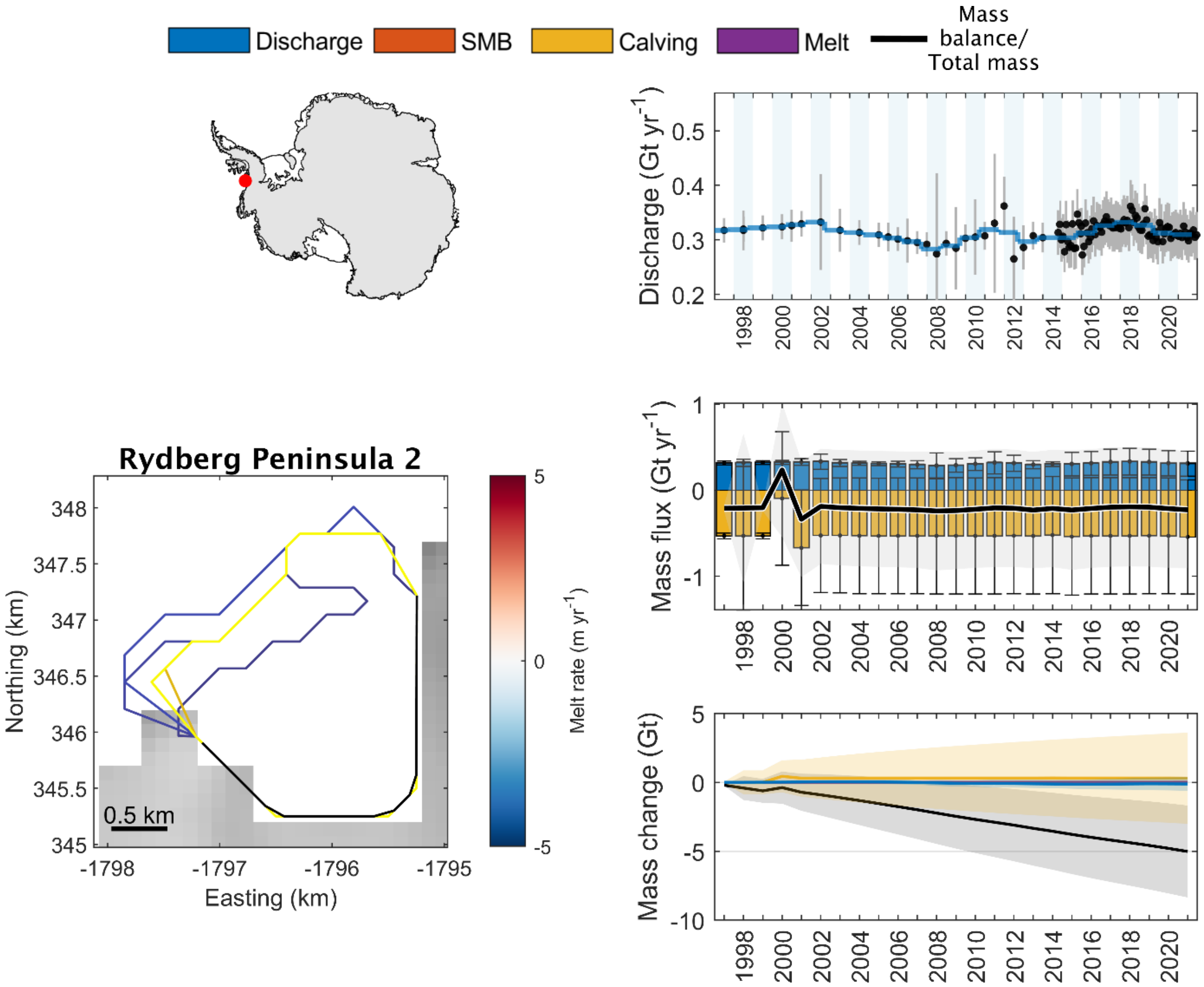

Discharge
SMB
Calving
Melt
Mass balance/ Total mass
Rydberg Peninsula 2
Northing (km)
Easting (km)
0.5 km
Melt rate (m yr-1)
Discharge (Gt yr-1)
Mass flux (Gt yr-1)
Mass change (Gt)

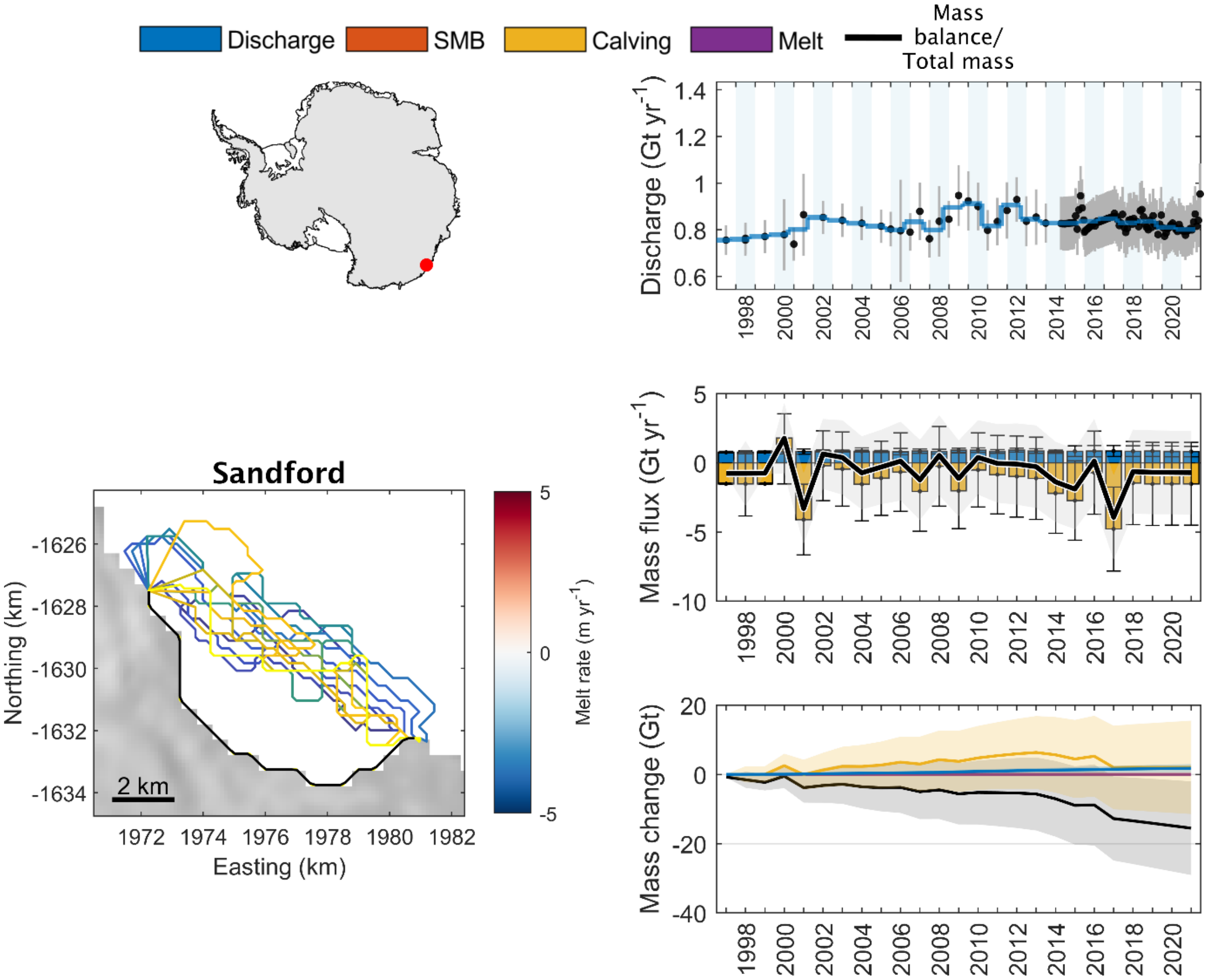

Discharge
SMB
Calving
Melt
Mass balance/ Total mass
Sandford
Northing (km)
Easting (km)
2 km
Melt rate (m yr-1)
Discharge (Gt yr-1)
Mass flux (Gt yr-1)
Mass change (Gt)

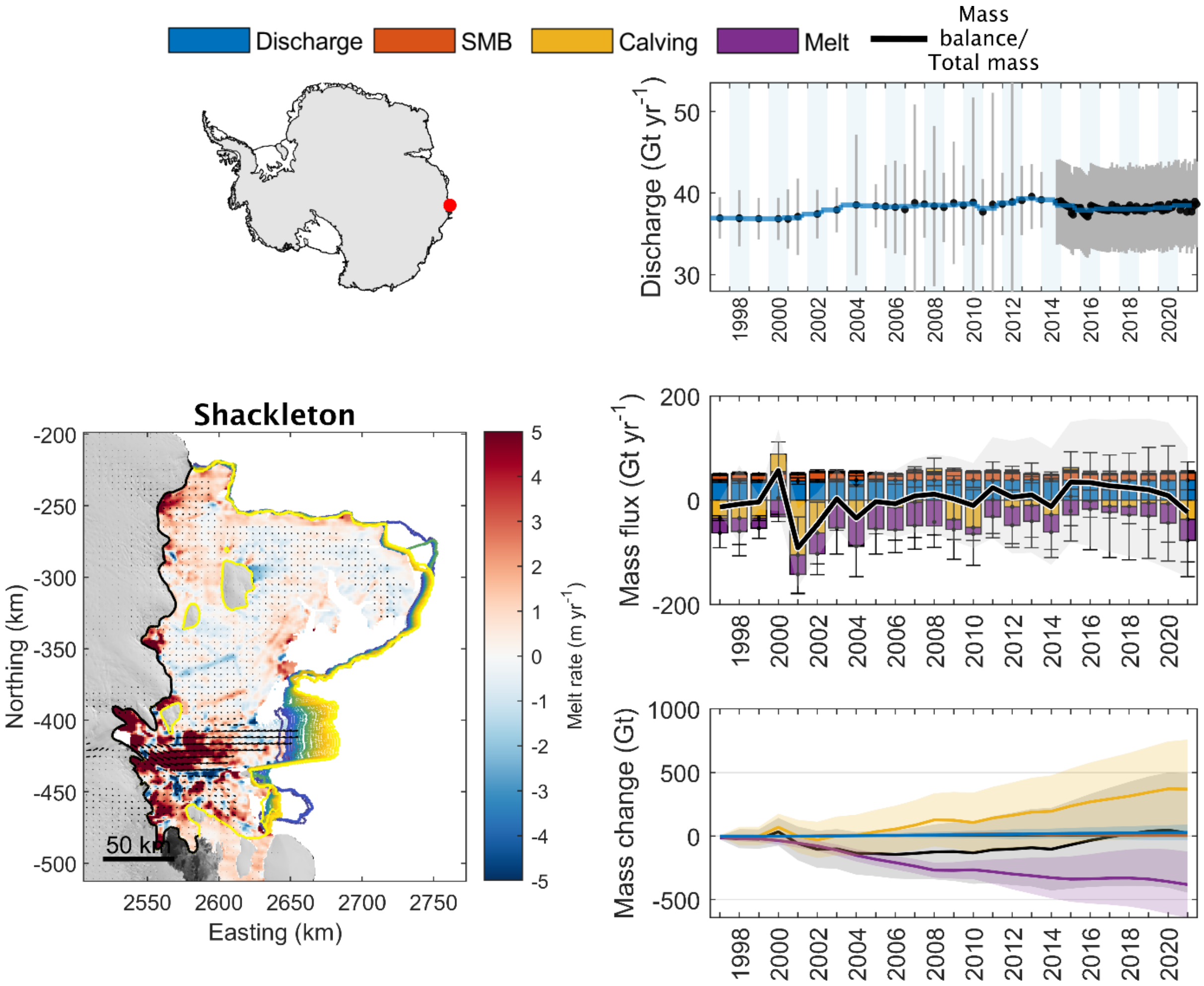
Discharge
SMB
Calving
Melt
Mass balance/ Total mass
Discharge (Gt yr-1)
Shackleton
Northing (km)
Easting (km)
Melt rate (m yr-1)
50 km
Mass flux (Gt yr-1)
Mass change (Gt)

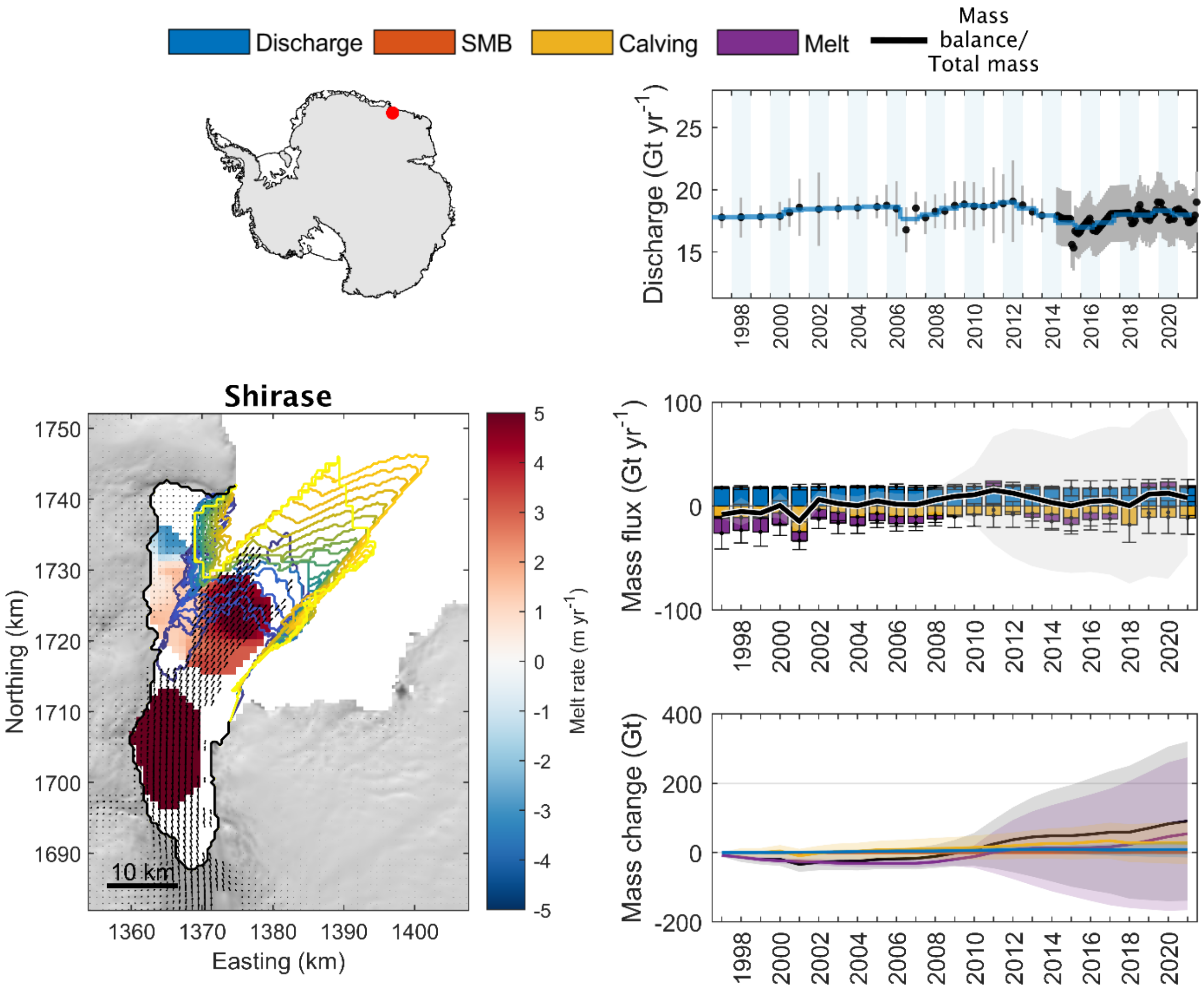

Discharge
SMB
Calving
Melt
Mass balance/ Total mass
Shirase
Northing (km)
Easting (km)
10 km
Melt rate (m yr^{-1})
Discharge (Gt yr^{-1})
Mass flux (Gt yr^{-1})
Mass change (Gt)

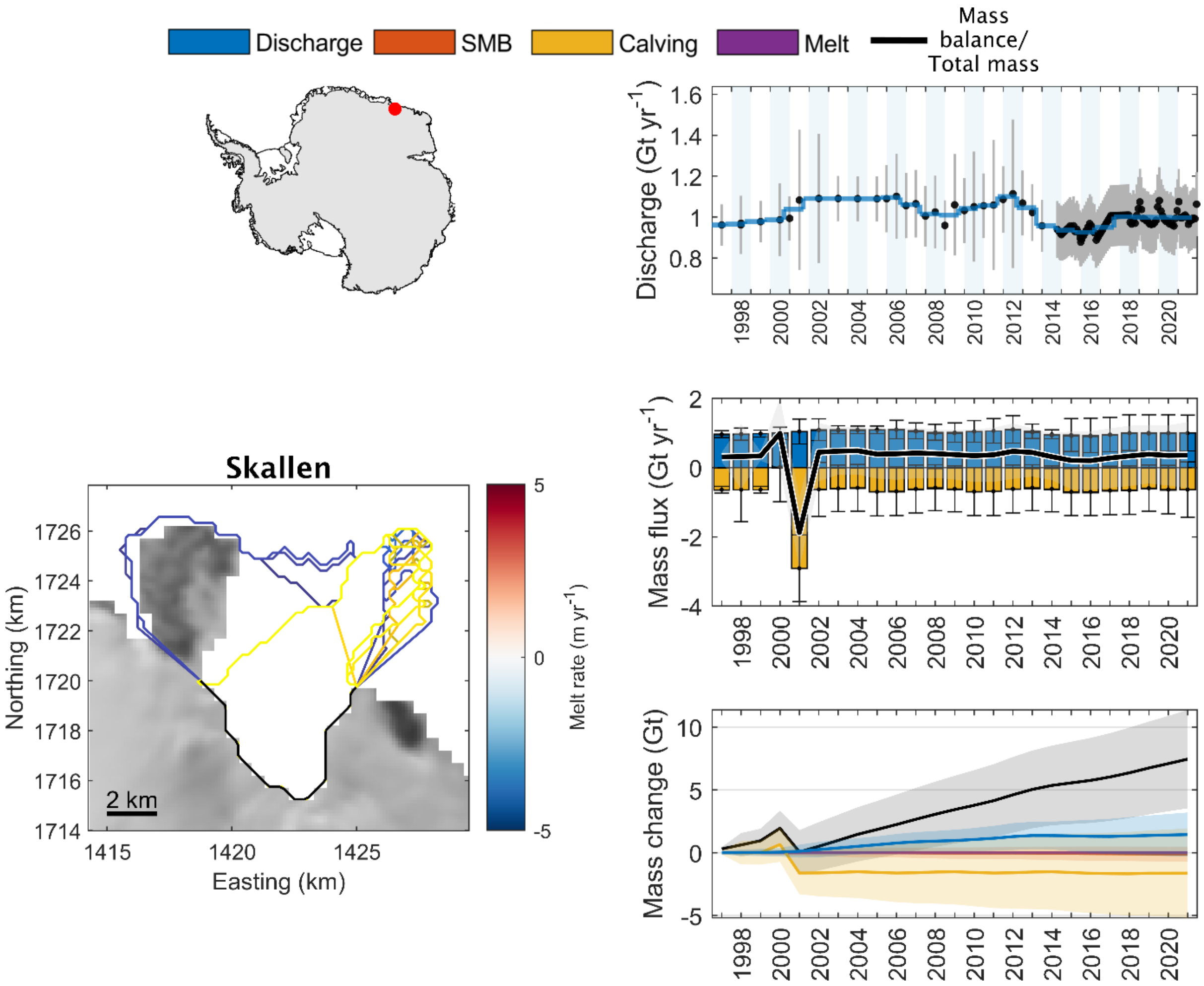
Discharge
SMB
Calving
Melt
Mass balance/ Total mass
Skallen
Northing (km)
Easting (km)
2 km
Melt rate (m yr^{-1})
Discharge (Gt yr^{-1})
Mass flux (Gt yr^{-1})
Mass change (Gt)

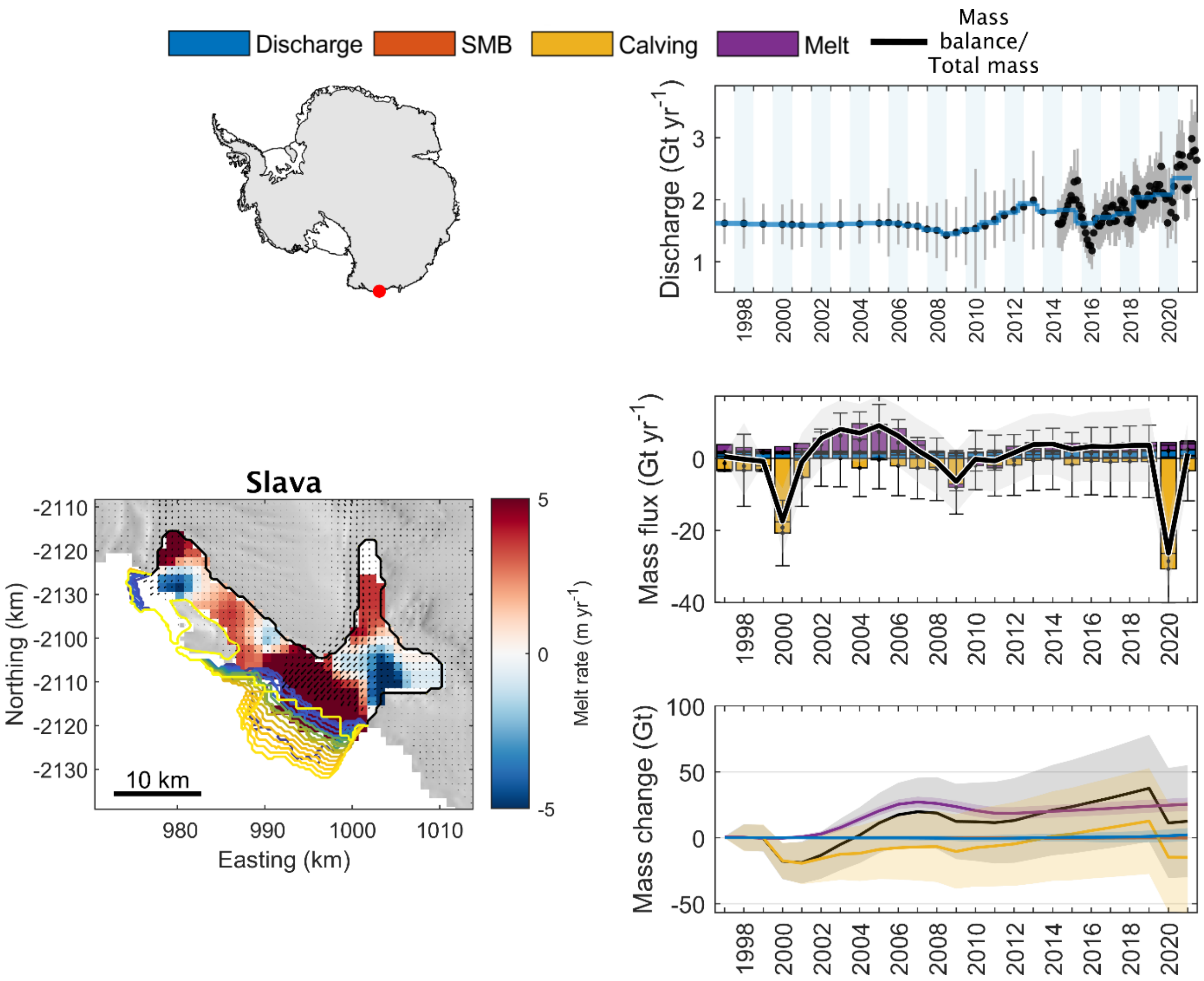

Discharge
SMB
Calving
Melt
Mass balance/ Total mass
Discharge (Gt yr^{-1})
Slava
Northing (km)
Easting (km)
10 km
Melt rate (m yr^{-1})
Mass flux (Gt yr^{-1})
Mass change (Gt)

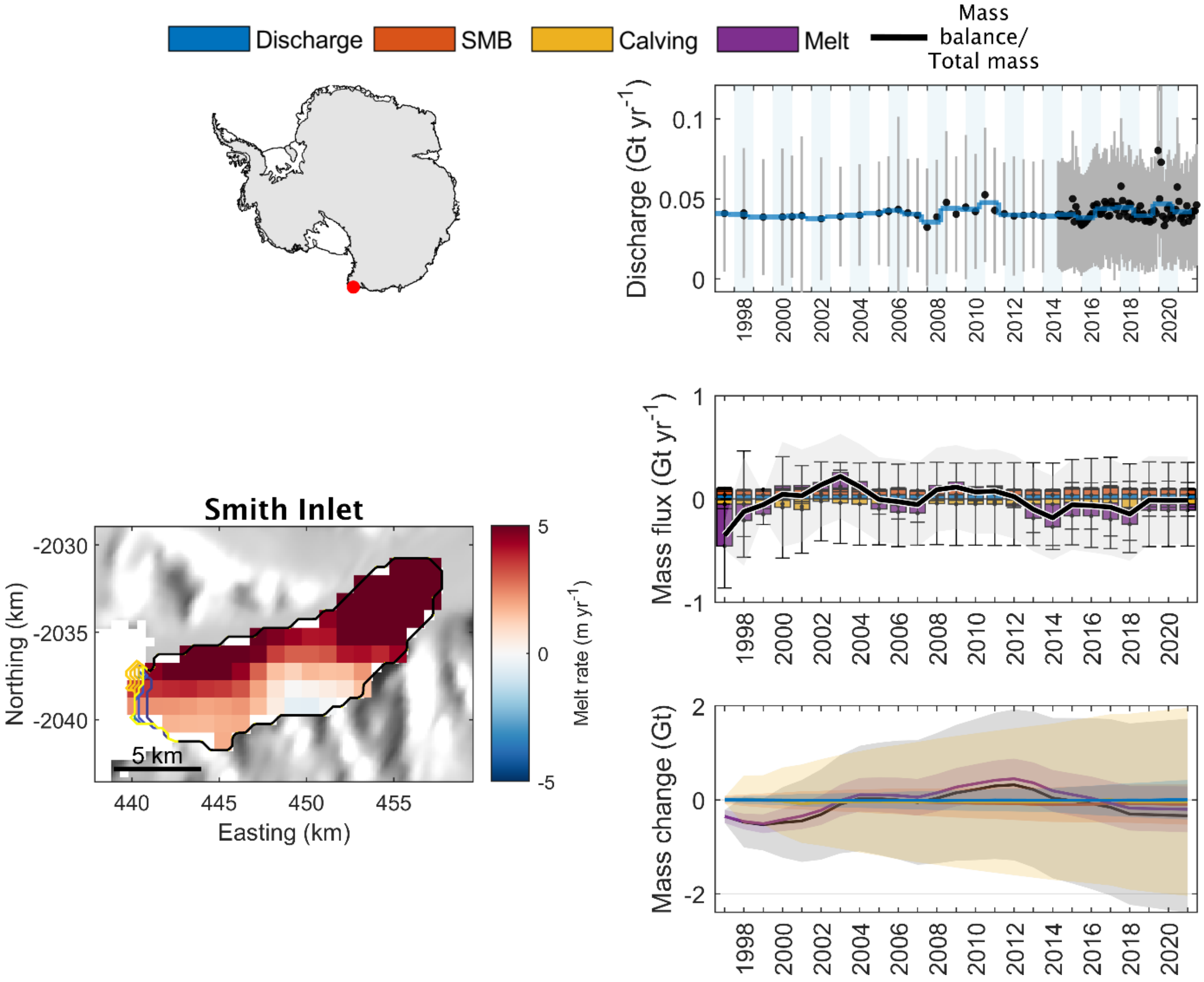

Discharge
SMB
Calving
Melt
Mass balance/ Total mass
Smith Inlet
Northing (km)
Easting (km)
5 km
Melt rate (m yr-1)
Discharge (Gt yr-1)
Mass flux (Gt yr-1)
Mass change (Gt)

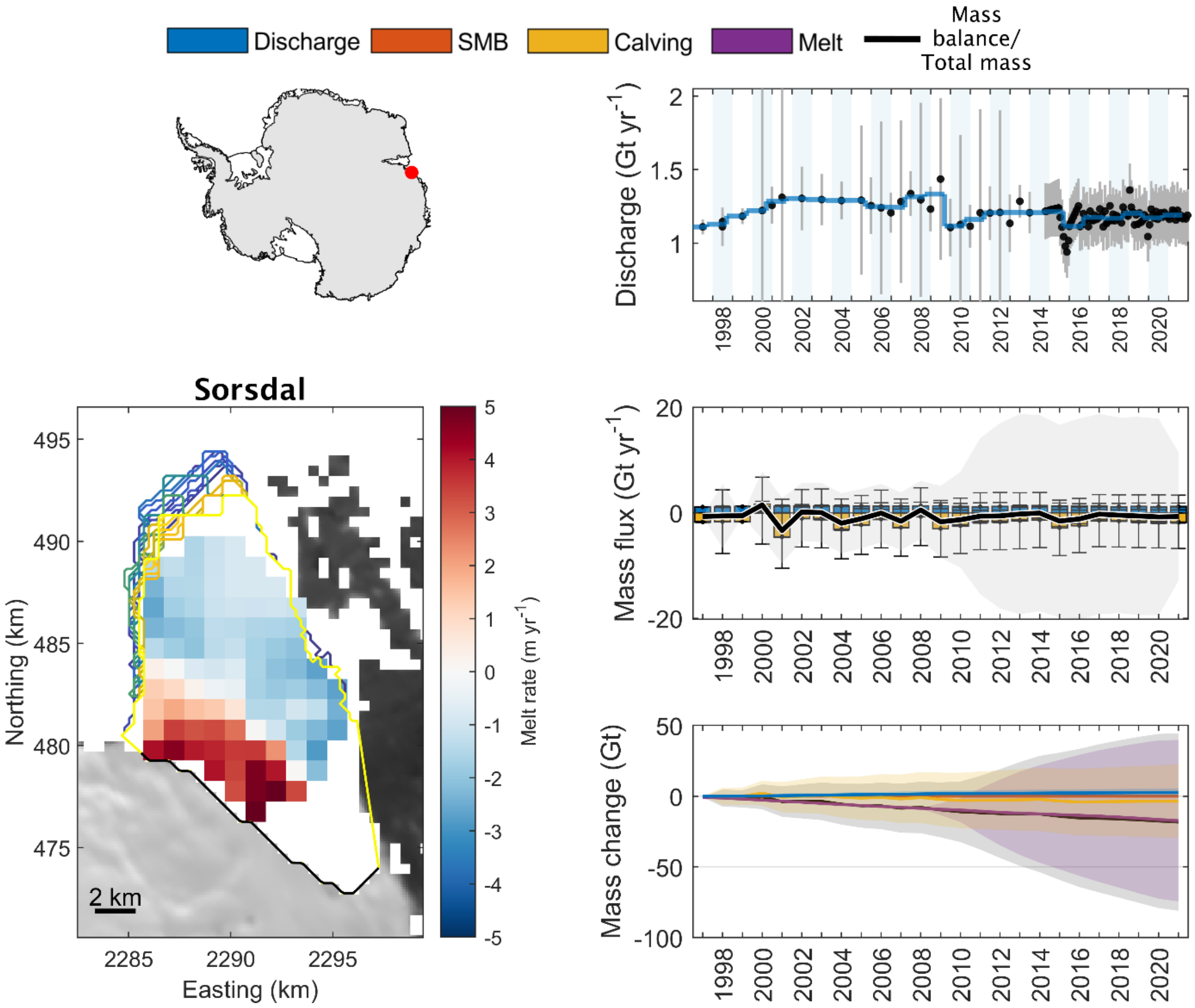
Discharge
SMB
Calving
Melt
Mass balance/ Total mass
Sorsdal
Northing (km)
Easting (km)
2 km
Melt rate (m yr-1)
Discharge (Gt yr-1)
Mass flux (Gt yr-1)
Mass change (Gt)

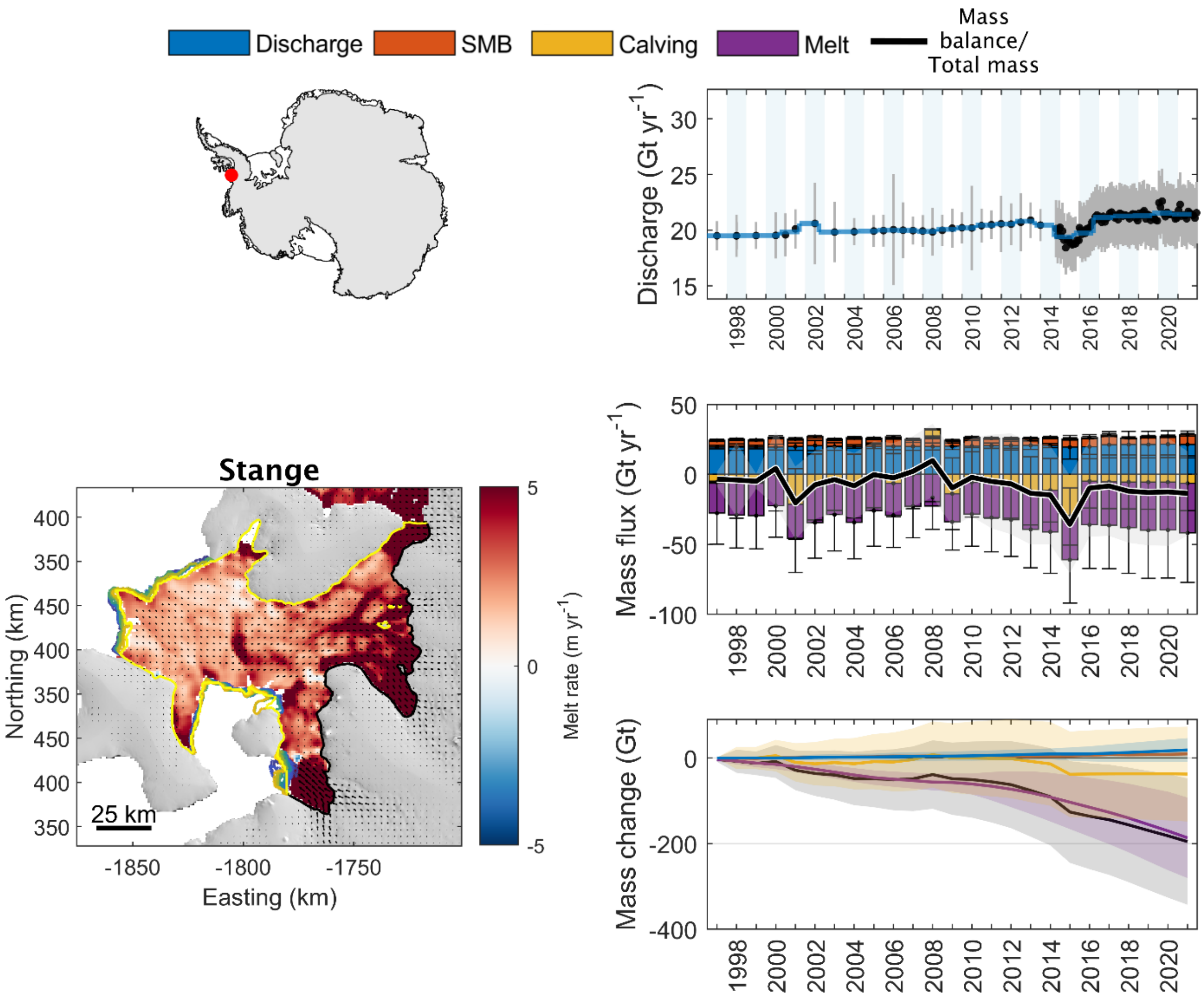
Discharge
SMB
Calving
Melt
Mass balance/ Total mass
Stange
25 km
Northing (km)
Easting (km)
Melt rate (m yr-1)
Discharge (Gt yr-1)
Mass flux (Gt yr-1)
Mass change (Gt)

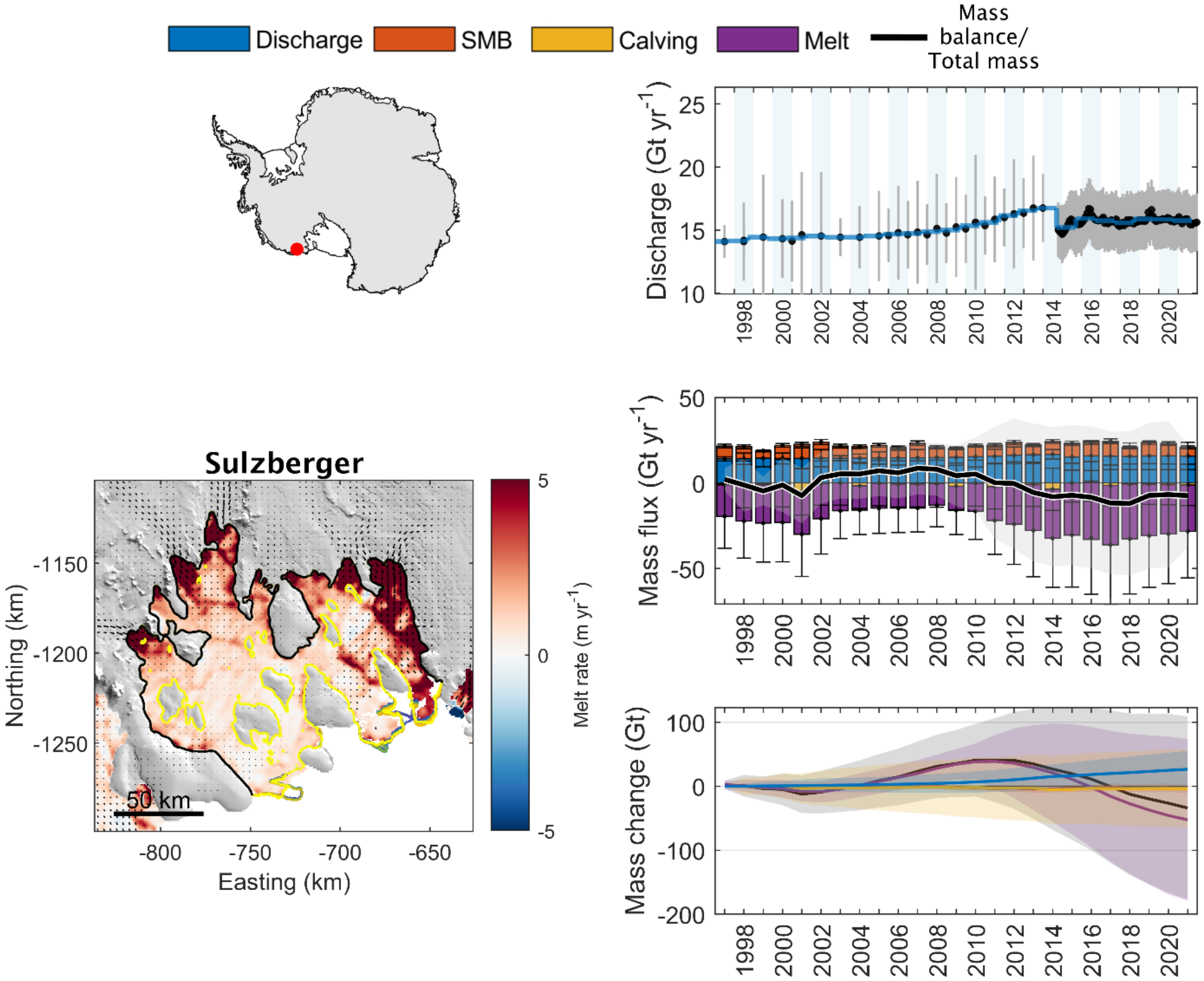

Discharge
SMB
Calving
Melt
Mass balance/ Total mass
Sulzberger
Northing (km)
-1150
-1200
-1250
50 km
-800
-750
-700
-650
Easting (km)
5
0
-5
Melt rate (m yr-1)
Discharge (Gt yr-1)
25
20
15
10
Mass flux (Gt yr-1)
50
0
-50
Mass change (Gt)
100
0
-100
-200
1998
2000
2002
2004
2006
2008
2010
2012
2014
2016
2018
2020

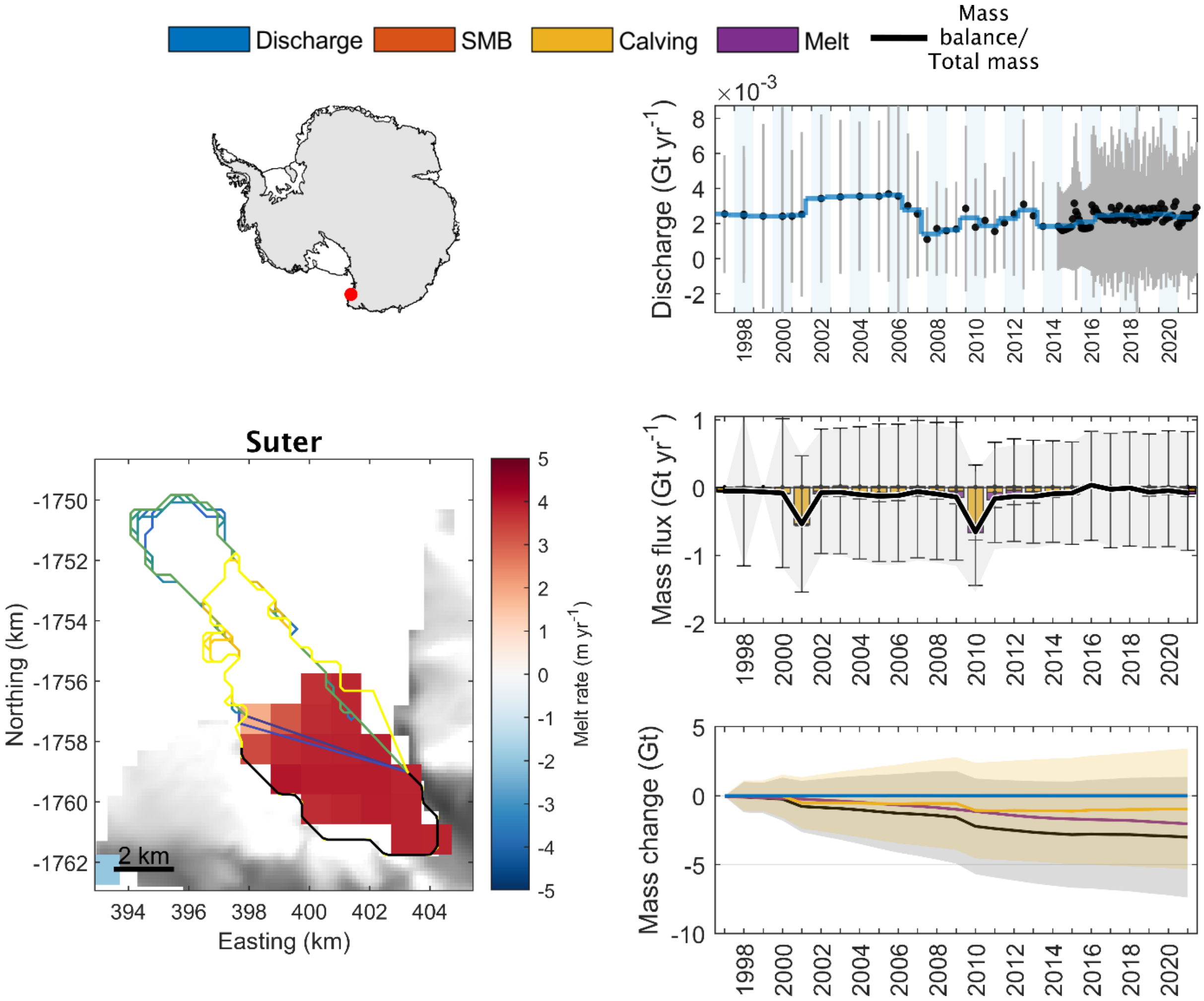
Discharge
SMB
Calving
Melt
Mass balance/ Total mass
Suter
Northing (km)
Easting (km)
2 km
Melt rate (m yr-1)
Discharge (Gt yr-1)
×10-3
Mass flux (Gt yr-1)
Mass change (Gt)

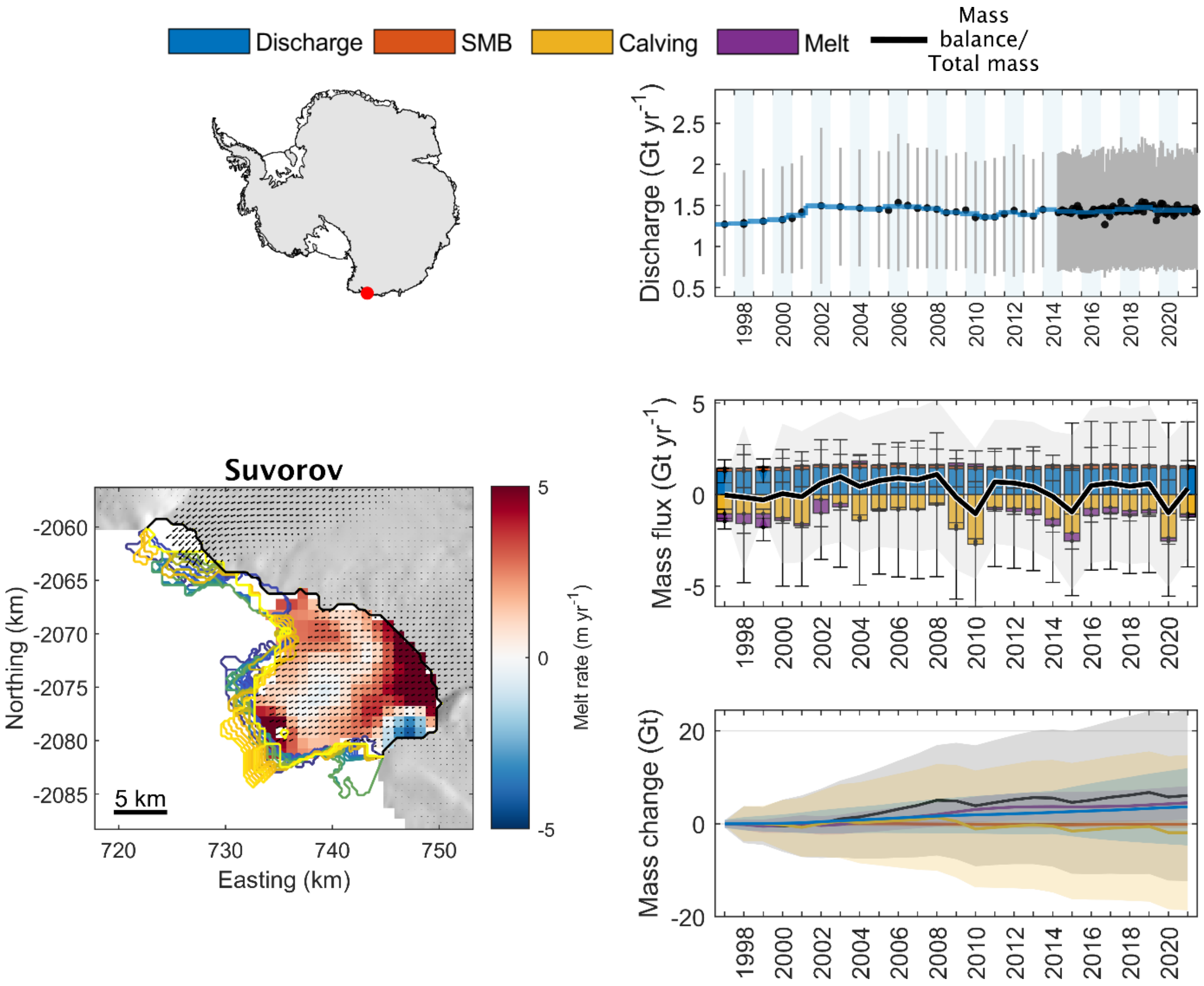
Discharge
SMB
Calving
Melt
Mass balance/ Total mass
Suvorov
Northing (km)
Easting (km)
5 km
Melt rate (m yr⁻¹)
Discharge (Gt yr⁻¹)
Mass flux (Gt yr⁻¹)
Mass change (Gt)

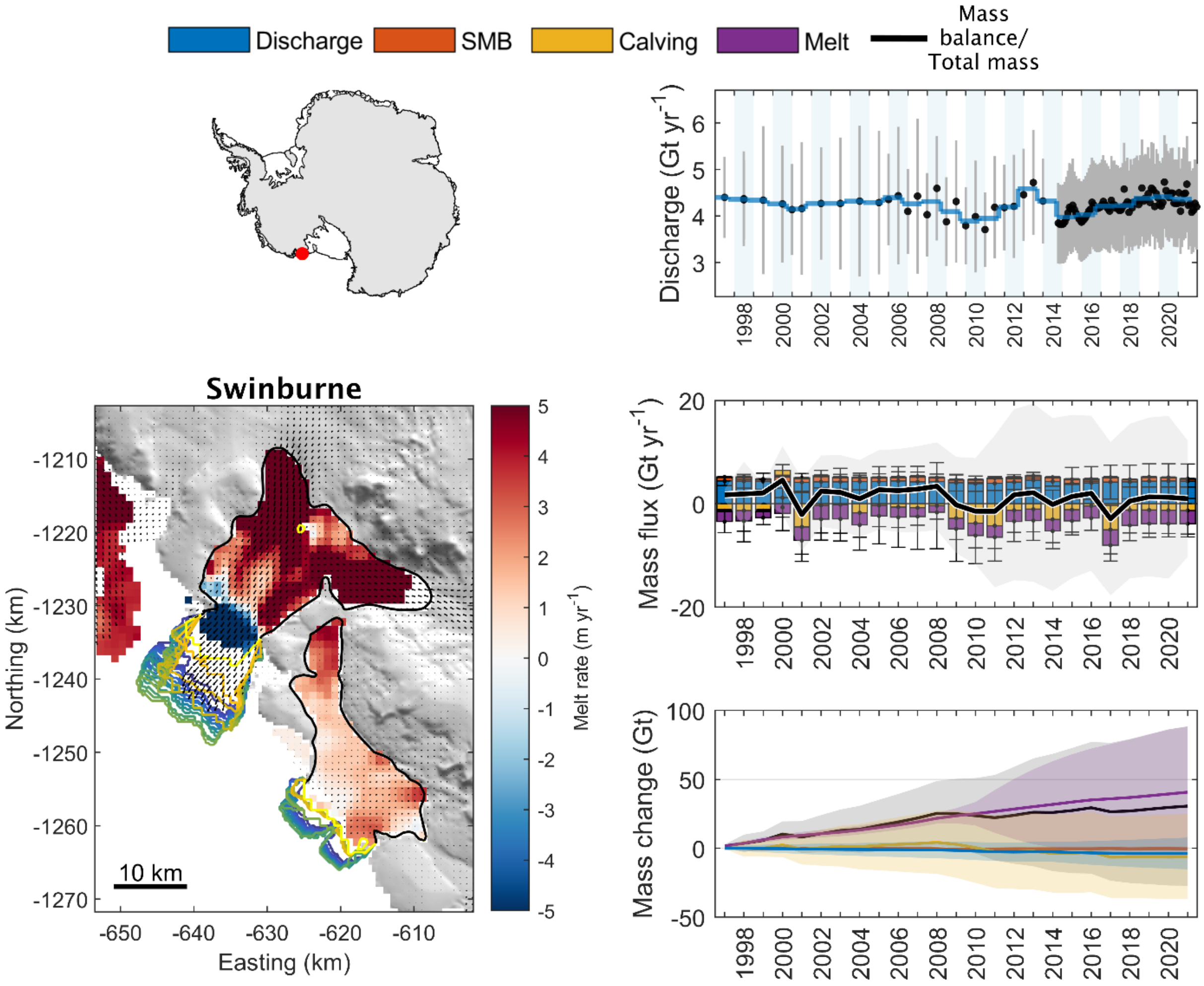
Discharge
SMB
Calving
Melt
Mass balance/ Total mass
Swinburne
Discharge (Gt yr-1)
Mass flux (Gt yr-1)
Mass change (Gt)
Northing (km)
Easting (km)
Melt rate (m yr-1)
10 km

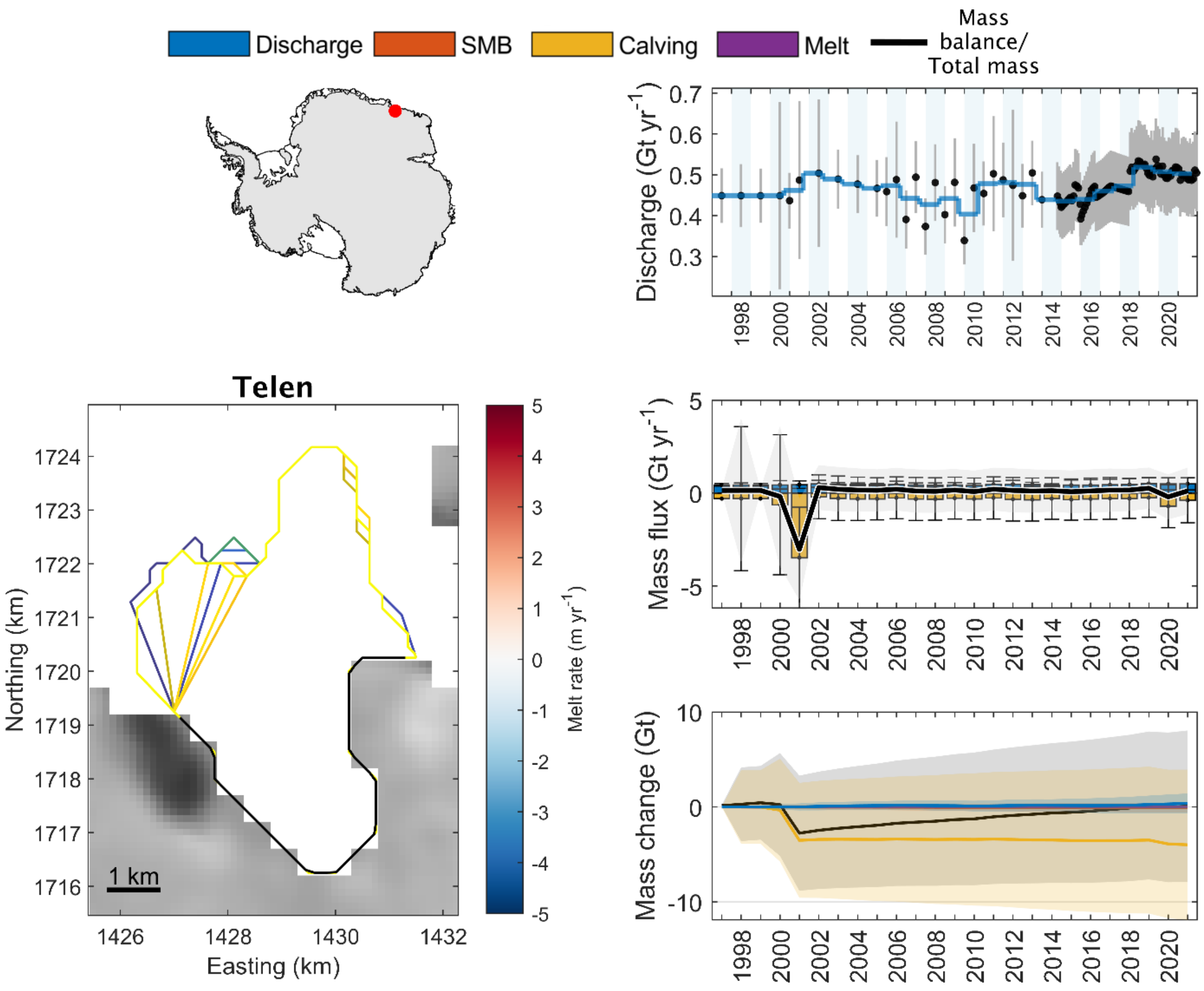

Discharge
SMB
Calving
Melt
Mass balance/ Total mass
Telen
Northing (km)
Easting (km)
1 km
Melt rate (m yr-1)
Discharge (Gt yr-1)
Mass flux (Gt yr-1)
Mass change (Gt)

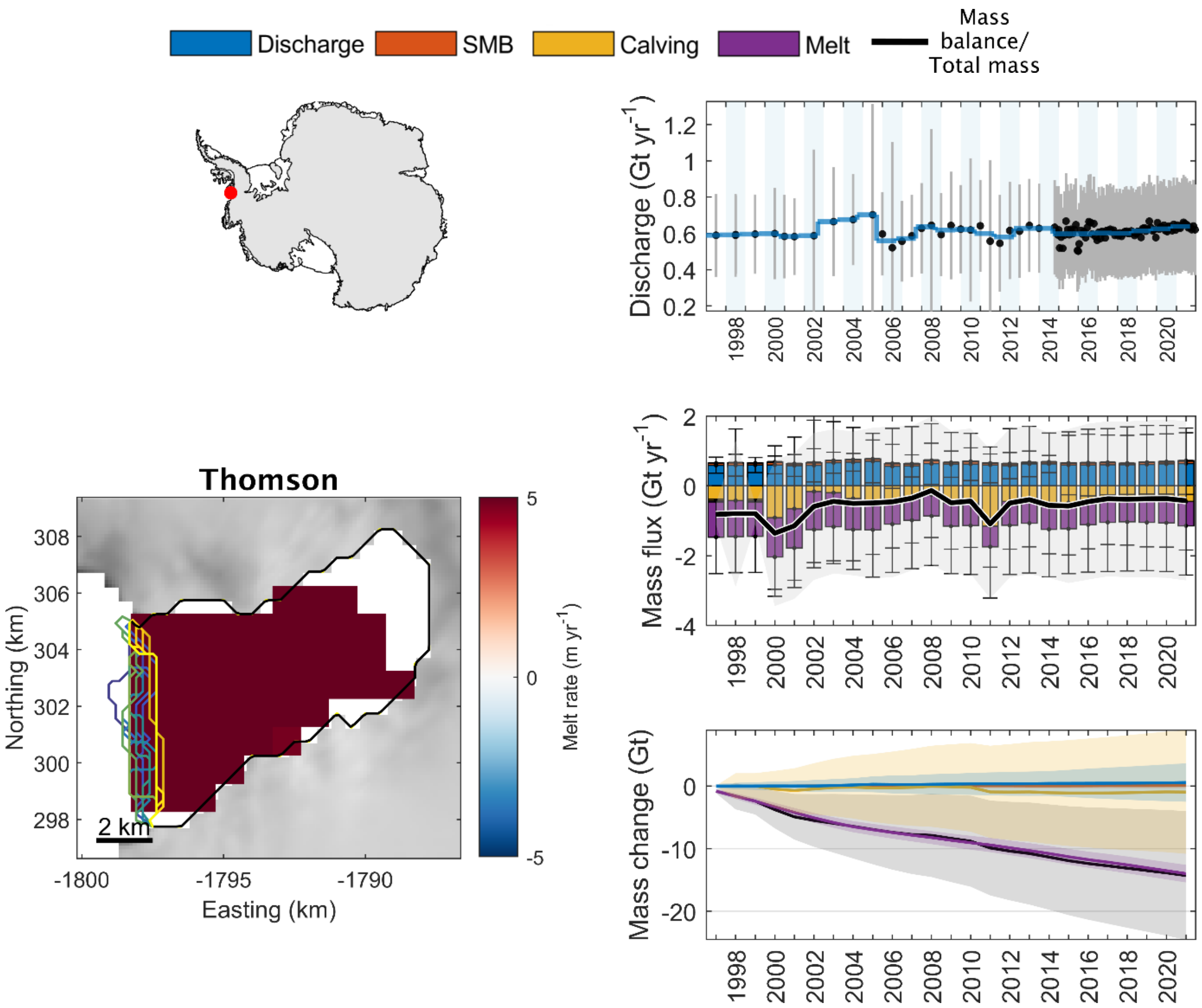
Discharge
SMB
Calving
Melt
Mass balance/ Total mass
Thomson
Discharge (Gt yr-1)
Mass flux (Gt yr-1)
Mass change (Gt)
Northing (km)
Easting (km)
Melt rate (m yr-1)
2 km

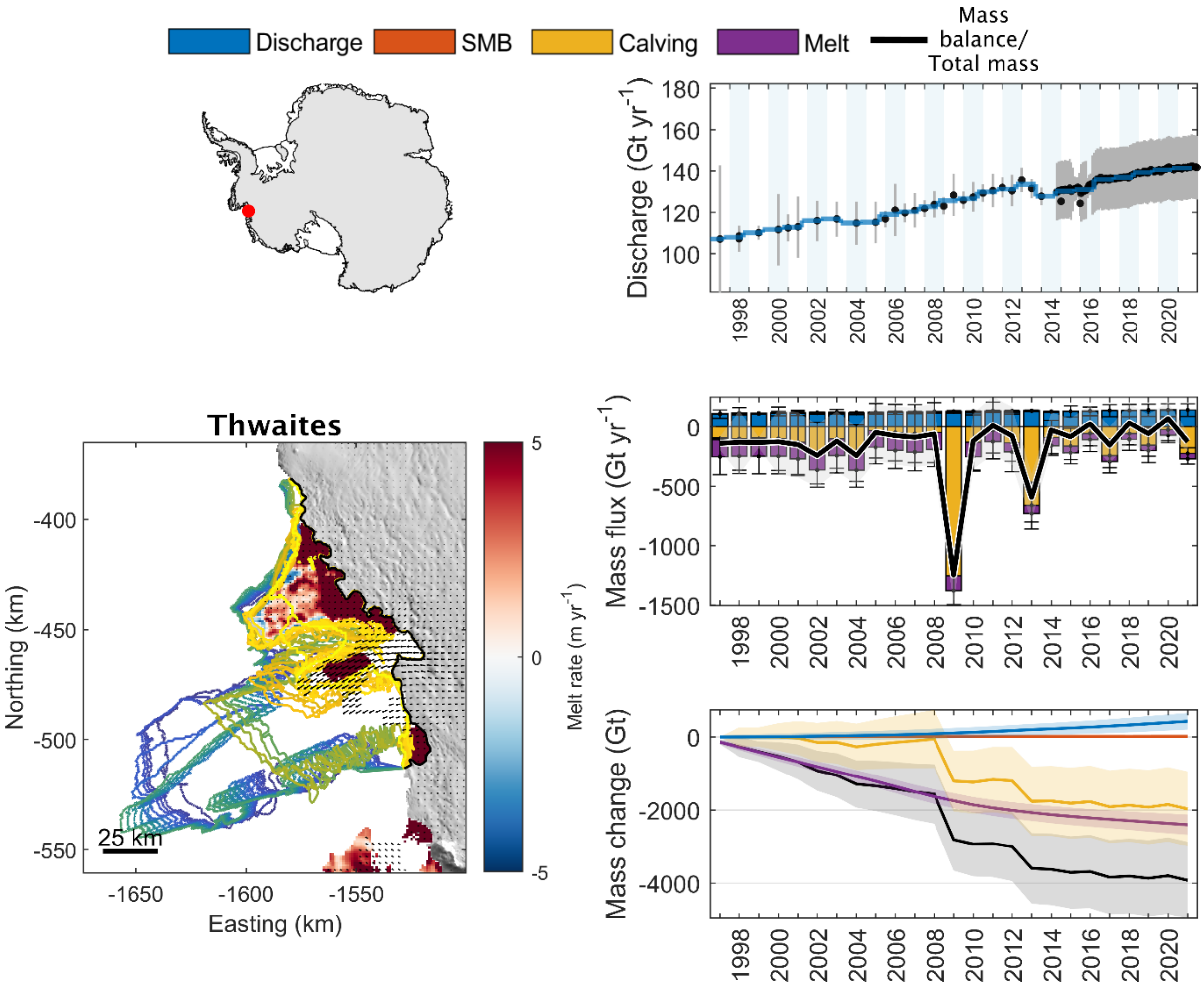

Discharge
SMB
Calving
Melt
Mass balance/ Total mass
Thwaites
Discharge (Gt yr^{-1})
Mass flux (Gt yr^{-1})
Mass change (Gt)
Northing (km)
Easting (km)
Melt rate (m yr^{-1})
25 km

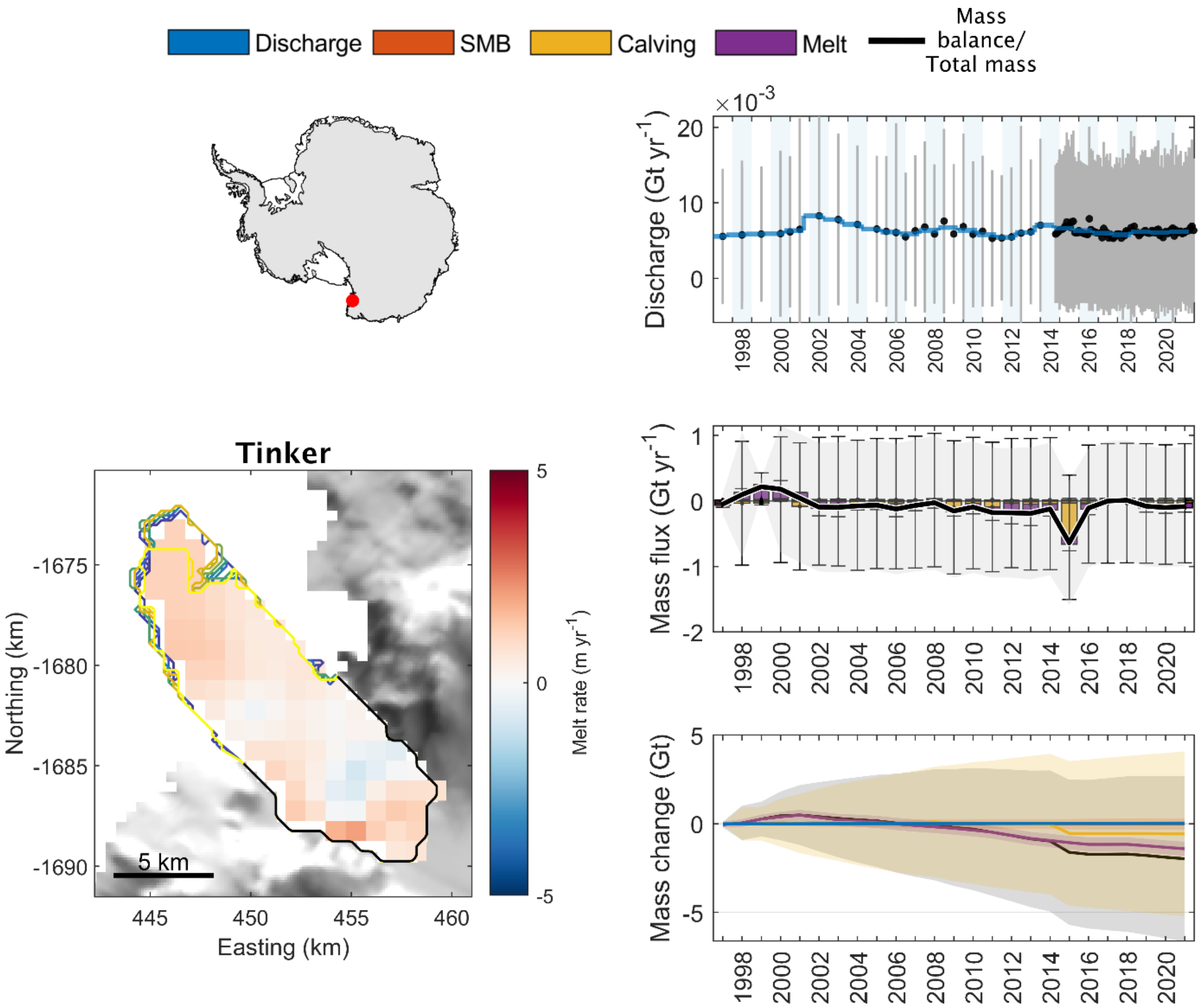
Discharge
SMB
Calving
Melt
Mass balance/ Total mass
Tinker
Northing (km)
Easting (km)
5 km
Melt rate (m yr^{-1})
Discharge (Gt yr^{-1})
$\times 10^{-3}$
Mass flux (Gt yr^{-1})
Mass change (Gt)

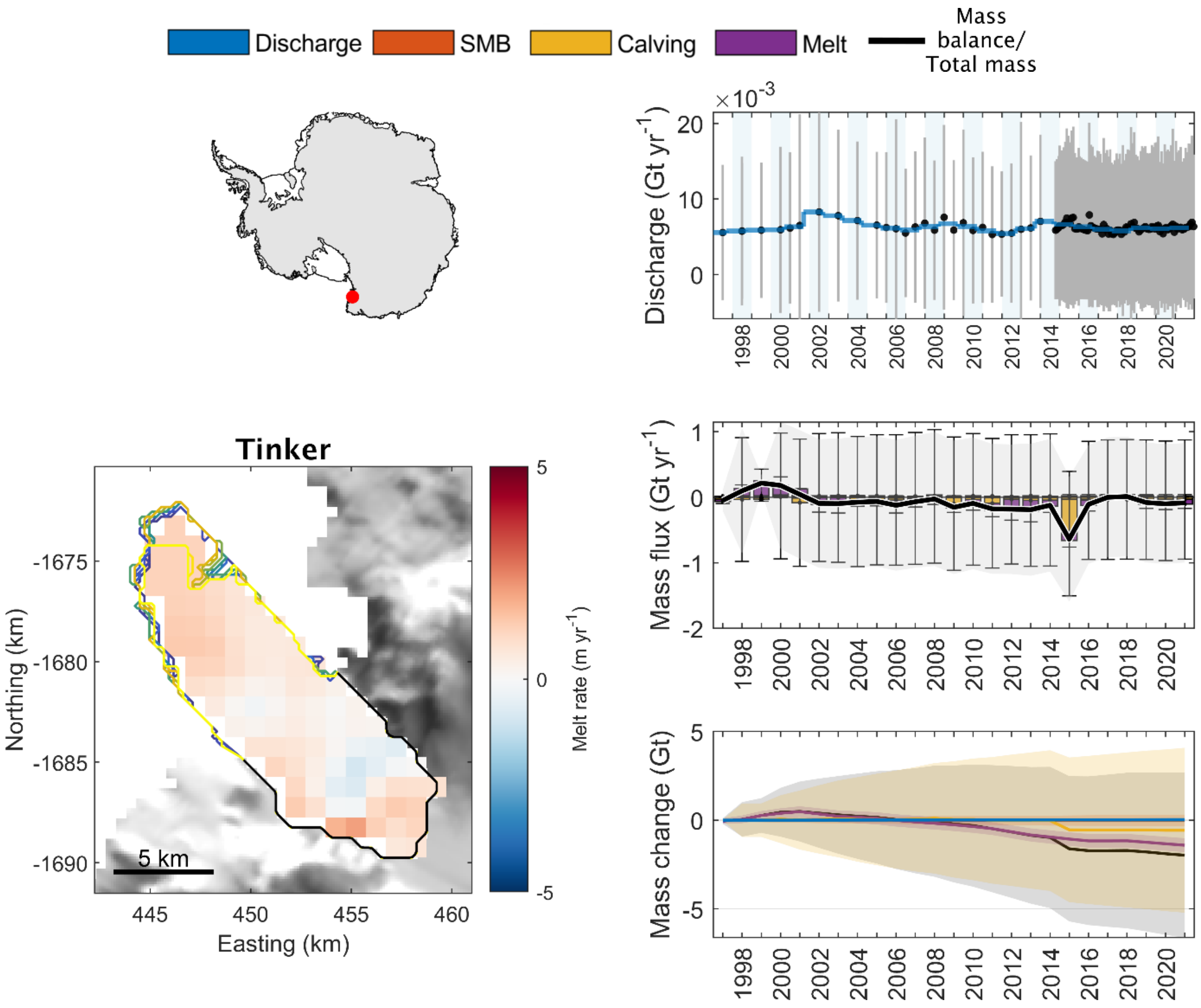

Discharge
SMB
Calving
Melt
Mass balance/ Total mass
Tinker
Northing (km)
Easting (km)
Melt rate (m yr^{-1})
5 km
Discharge (Gt yr^{-1})
$\times 10^{-3}$
Mass flux (Gt yr^{-1})
Mass change (Gt)

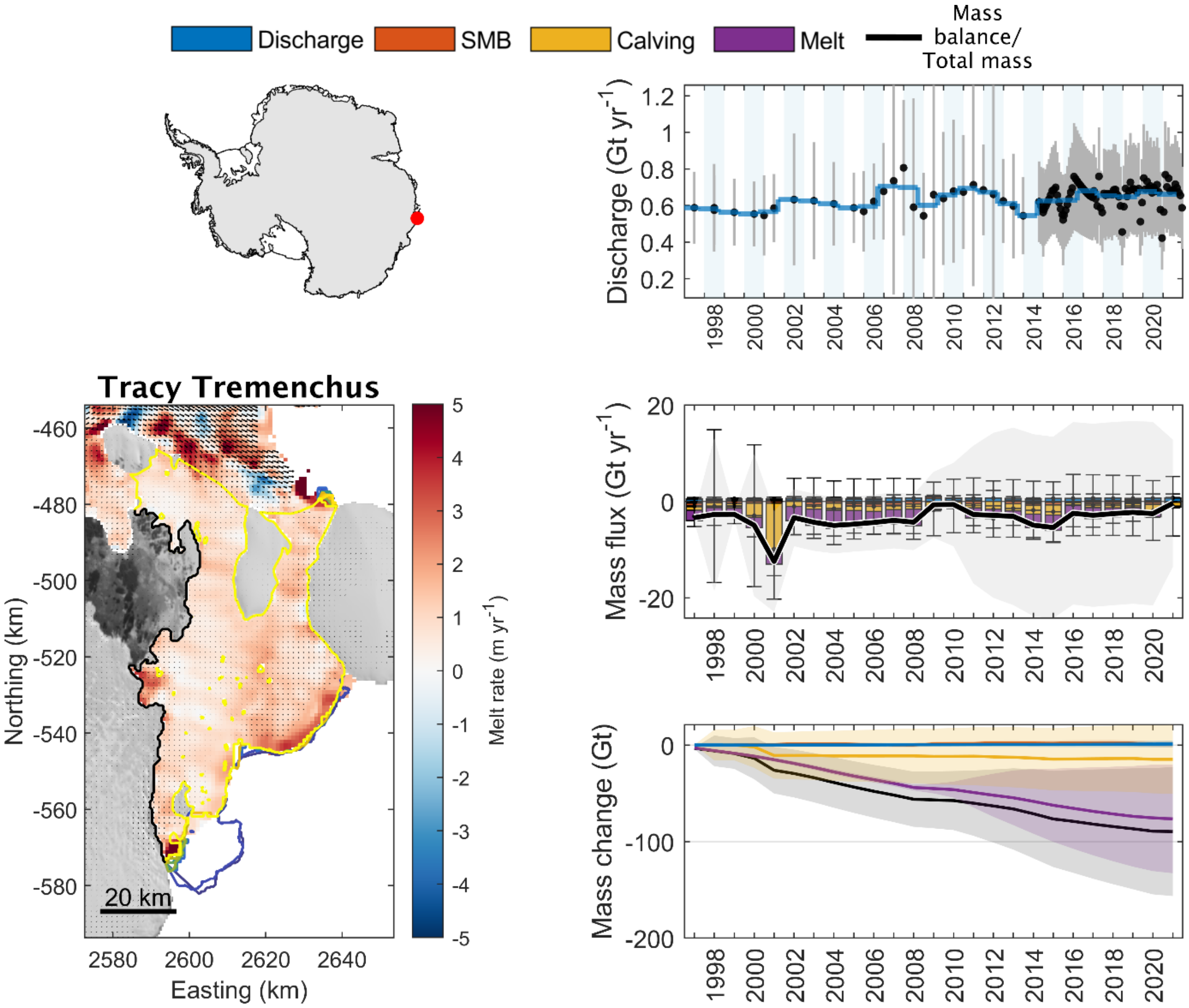
Discharge
SMB
Calving
Melt
Mass balance/ Total mass
Tracy Tremenchus
Discharge (Gt yr-1)
Mass flux (Gt yr-1)
Mass change (Gt)
Northing (km)
Easting (km)
Melt rate (m yr-1)
20 km

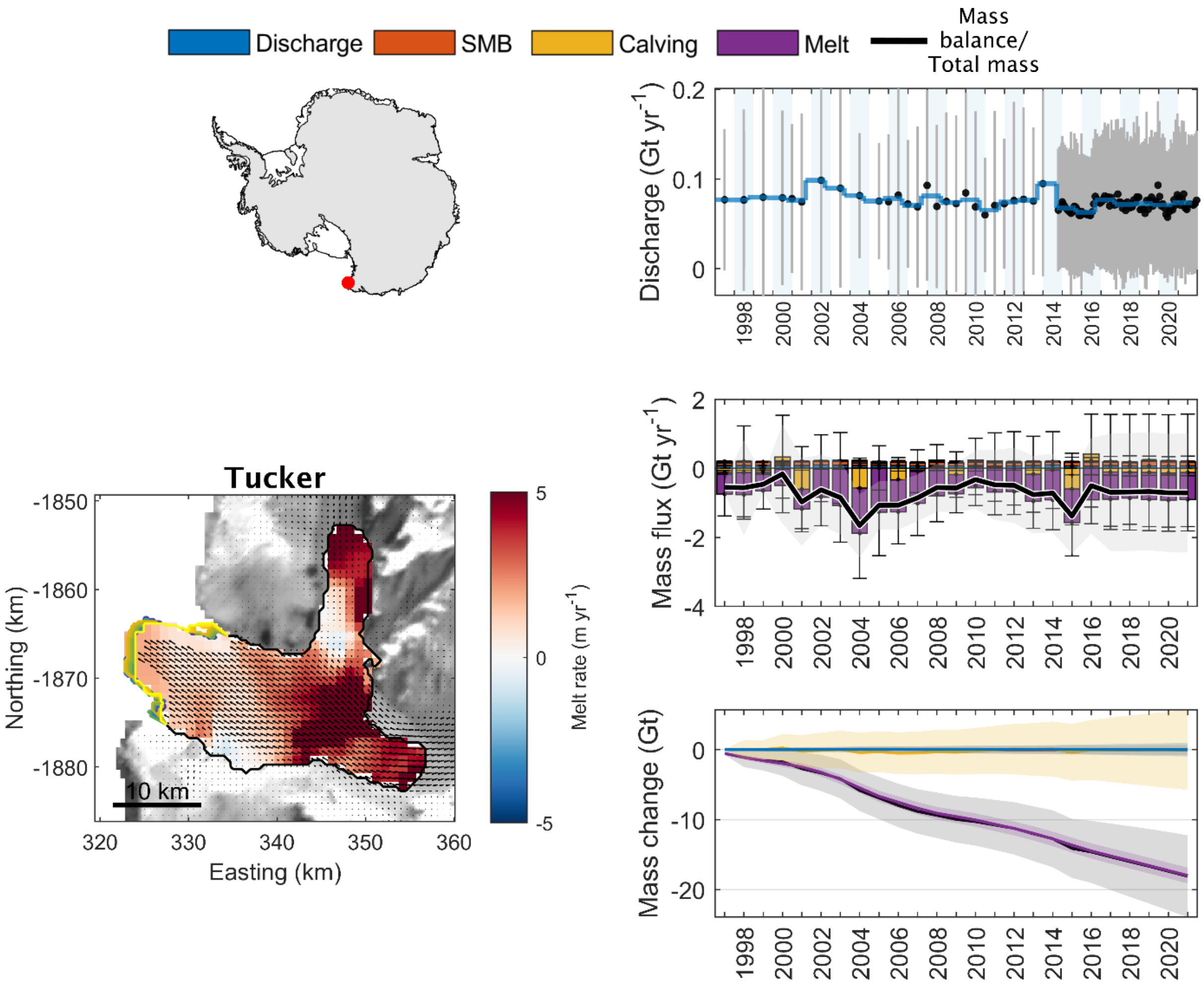

Discharge
SMB
Calving
Melt
Mass balance/ Total mass
Tucker
Northing (km)
Easting (km)
10 km
Melt rate (m yr^{-1})
Discharge (Gt yr^{-1})
Mass flux (Gt yr^{-1})
Mass change (Gt)

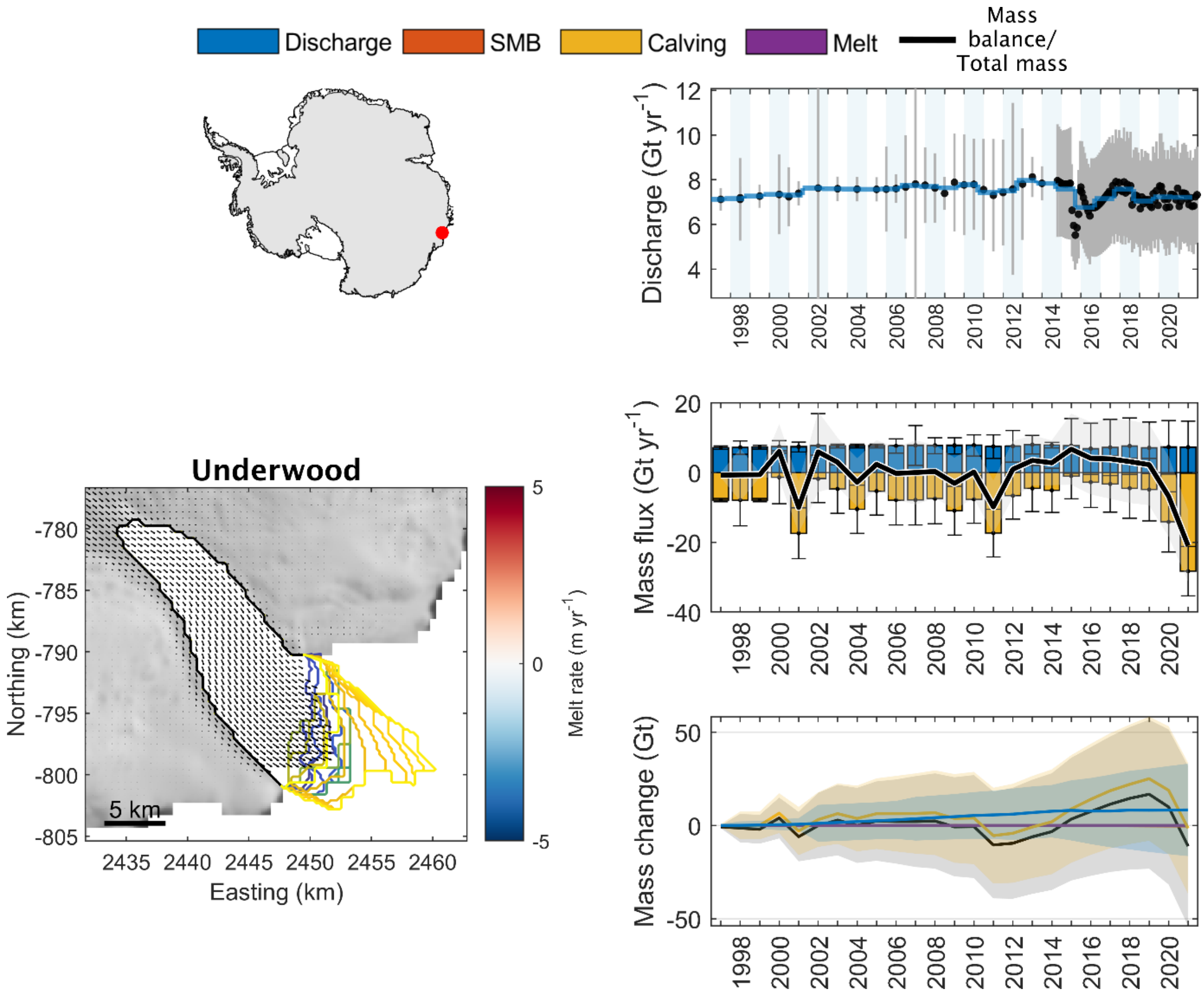
Discharge
SMB
Calving
Melt
Mass balance/ Total mass
Underwood
Northing (km)
Easting (km)
5 km
Melt rate (m yr-1)
Discharge (Gt yr-1)
Mass flux (Gt yr-1)
Mass change (Gt)

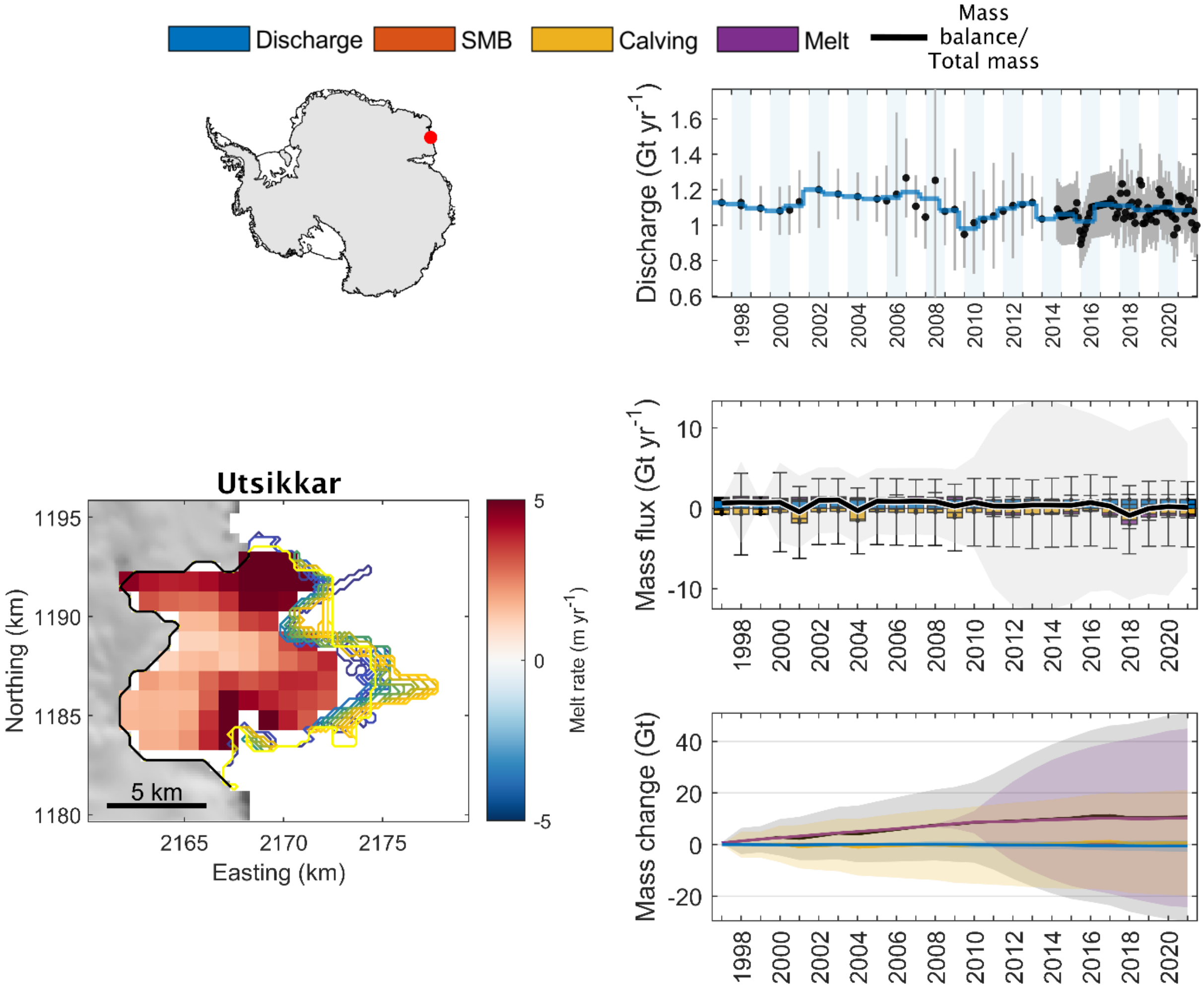

Discharge
SMB
Calving
Melt
Mass balance/ Total mass
Discharge (Gt yr-1)
Mass flux (Gt yr-1)
Mass change (Gt)
Utsikkar
Northing (km)
Easting (km)
5 km
Melt rate (m yr-1)

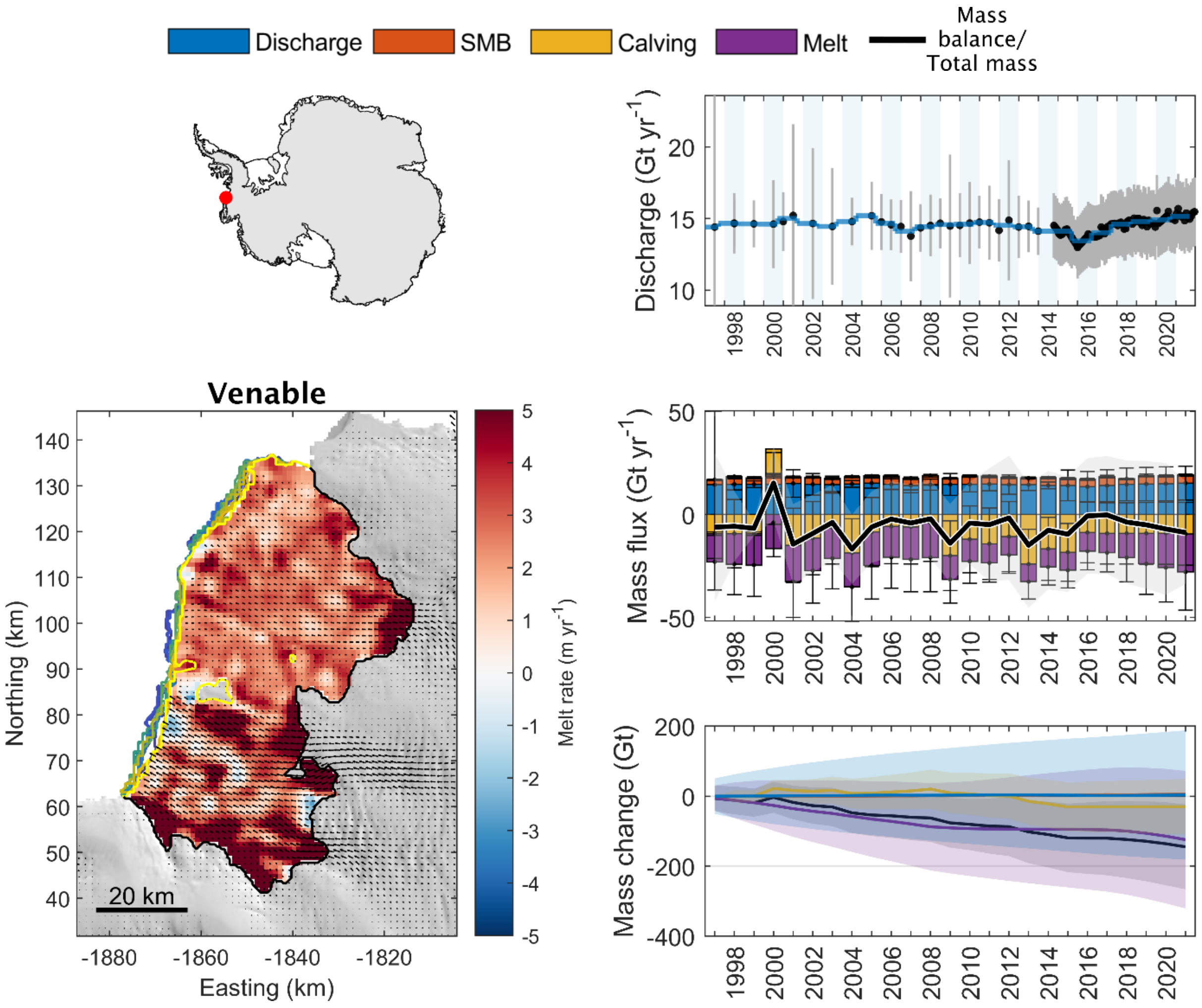
Discharge
SMB
Calving
Melt
Mass balance/ Total mass
Venable
Northing (km)
Easting (km)
20 km
Melt rate (m yr^{-1})
Discharge (Gt yr^{-1})
Mass flux (Gt yr^{-1})
Mass change (Gt)

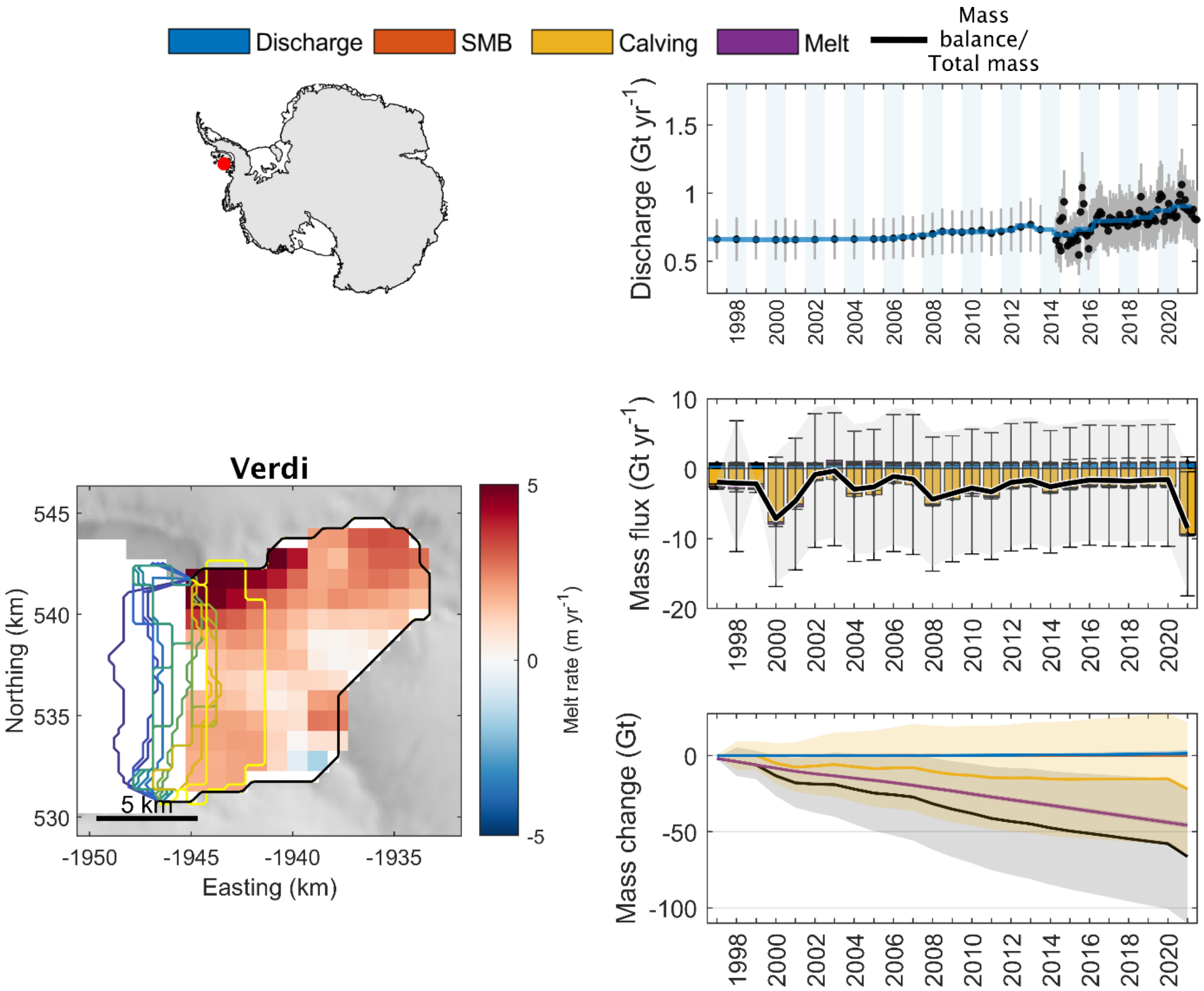

Discharge
SMB
Calving
Melt
Mass balance/ Total mass
Verdi
Northing (km)
Easting (km)
5 km
Melt rate (m yr^{-1})
Discharge (Gt yr^{-1})
Mass flux (Gt yr^{-1})
Mass change (Gt)

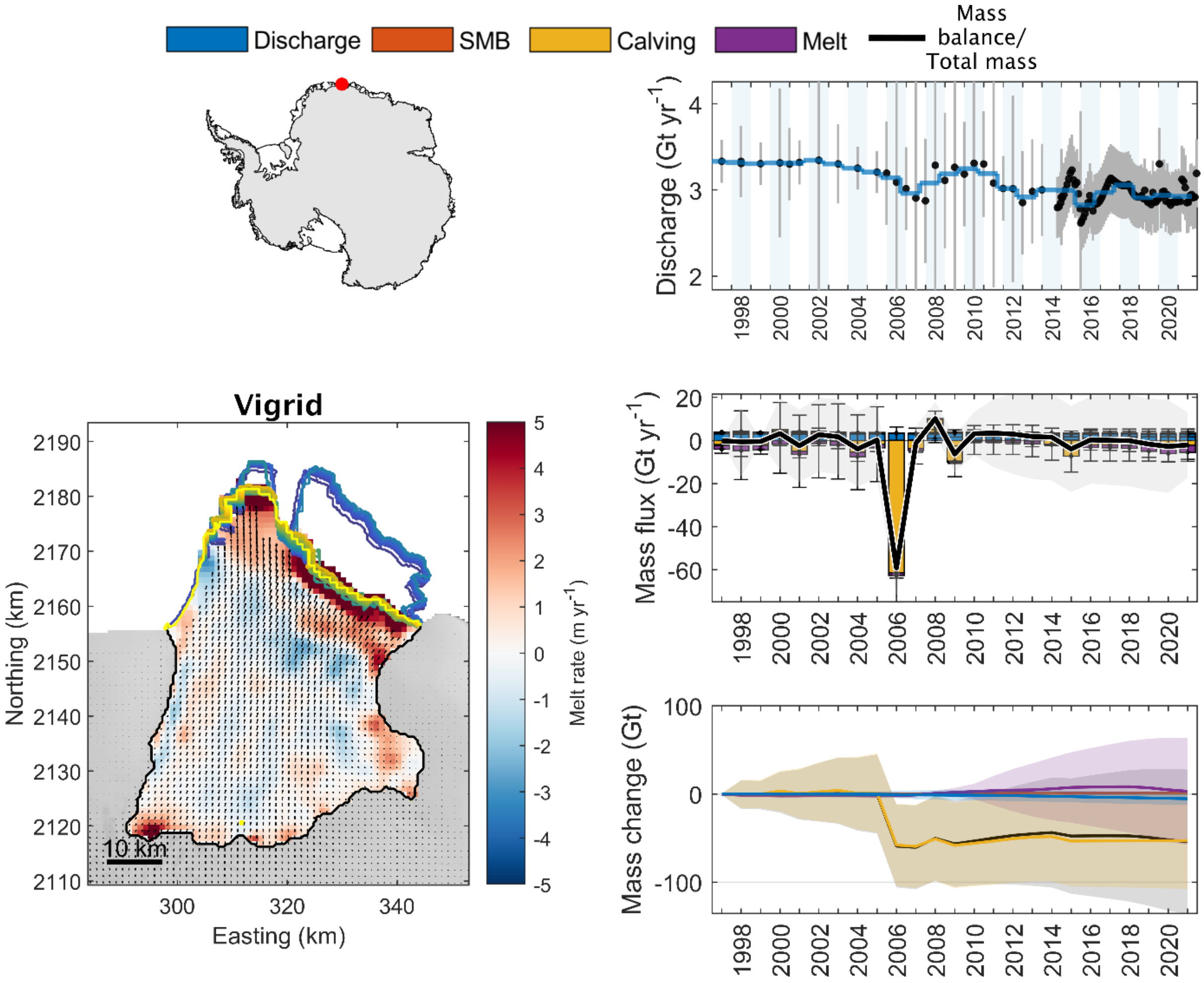
Discharge
SMB
Calving
Melt
Mass balance/ Total mass
Discharge (Gt yr^{-1})
Vigrid
Northing (km)
Easting (km)
10 km
Melt rate (m yr^{-1})
Mass flux (Gt yr^{-1})
Mass change (Gt)

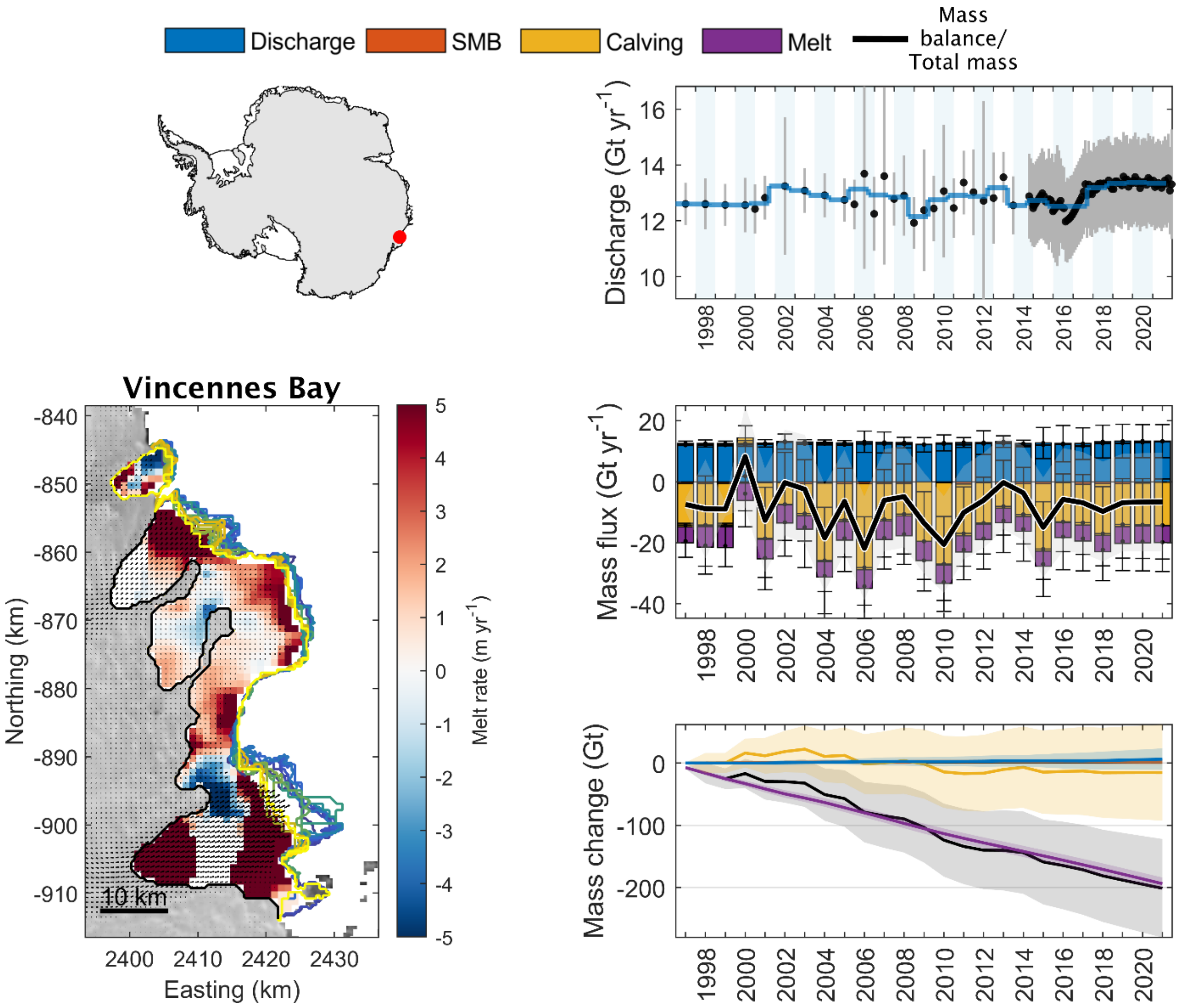
Discharge
SMB
Calving
Melt
Mass balance/ Total mass
Vincennes Bay
Discharge (Gt yr^{-1})
Mass flux (Gt yr^{-1})
Mass change (Gt)
Northing (km)
Easting (km)
Melt rate (m yr^{-1})
10 km

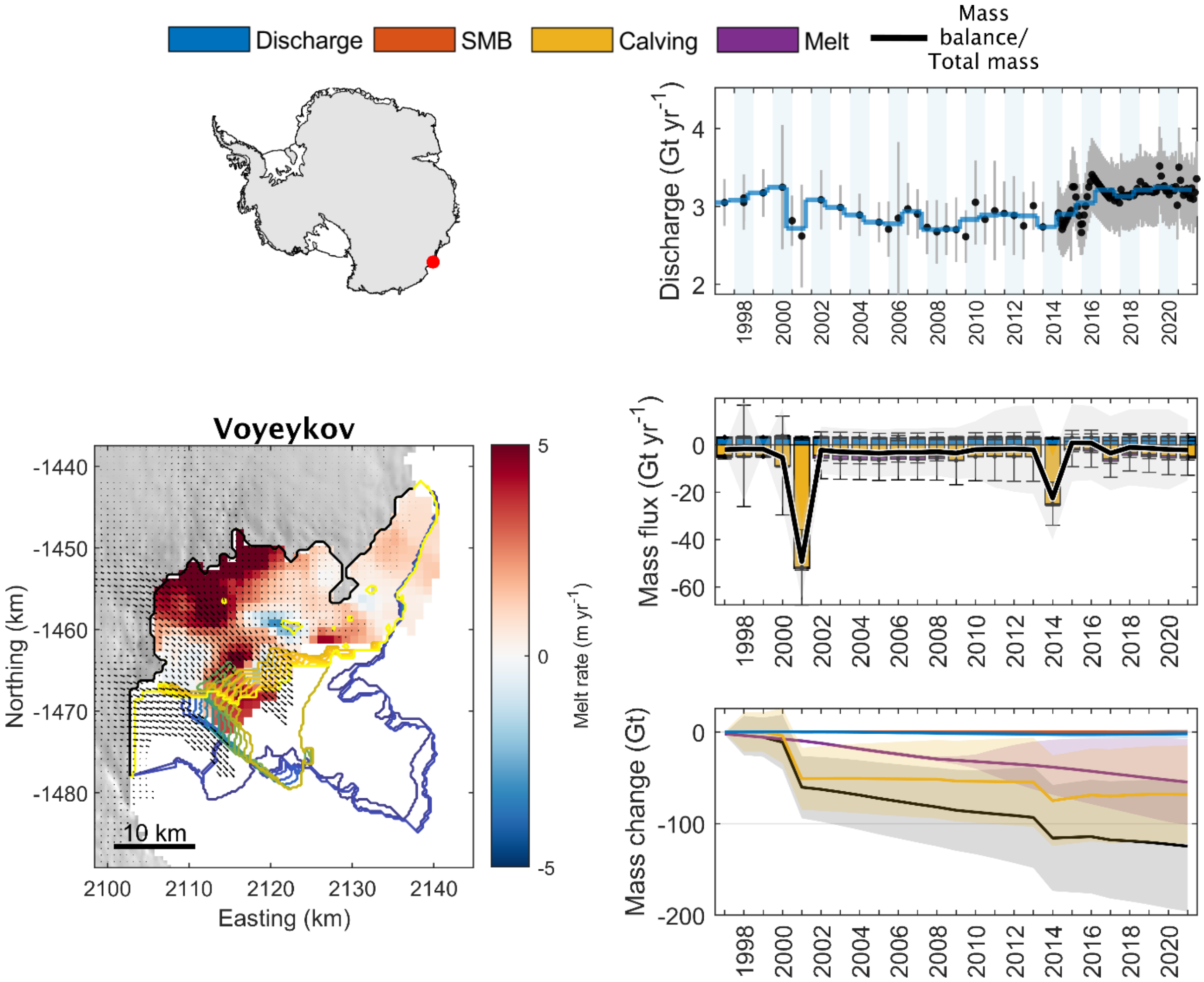
Discharge
SMB
Calving
Melt
Mass balance/ Total mass
Discharge (Gt yr-1)
Voyeykov
Northing (km)
Easting (km)
10 km
Melt rate (m yr-1)
Mass flux (Gt yr-1)
Mass change (Gt)

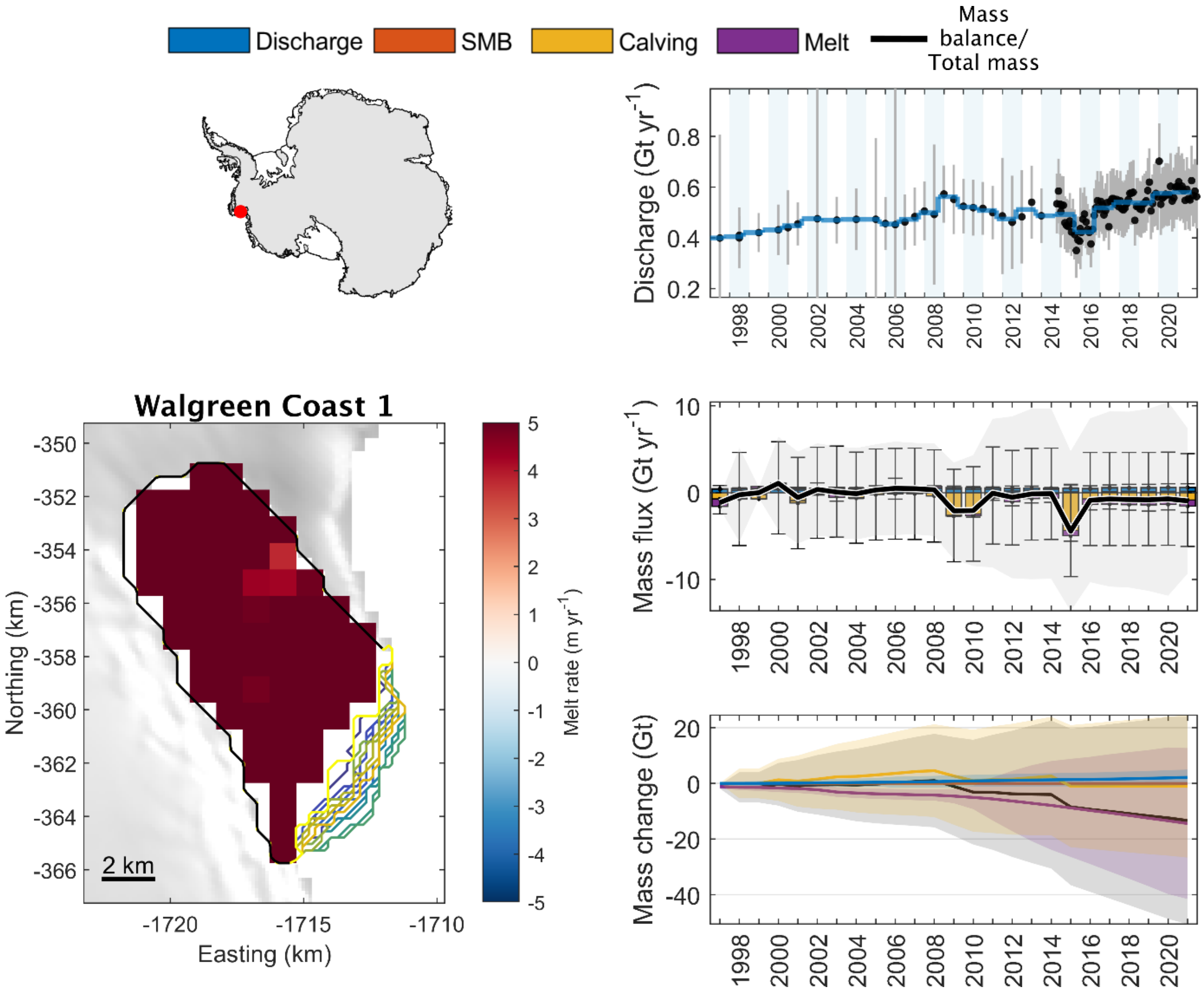

Discharge
SMB
Calving
Melt
Mass balance/ Total mass
Walgreen Coast 1
Northing (km)
Easting (km)
2 km
Melt rate (m yr-1)
Discharge (Gt yr-1)
Mass flux (Gt yr-1)
Mass change (Gt)

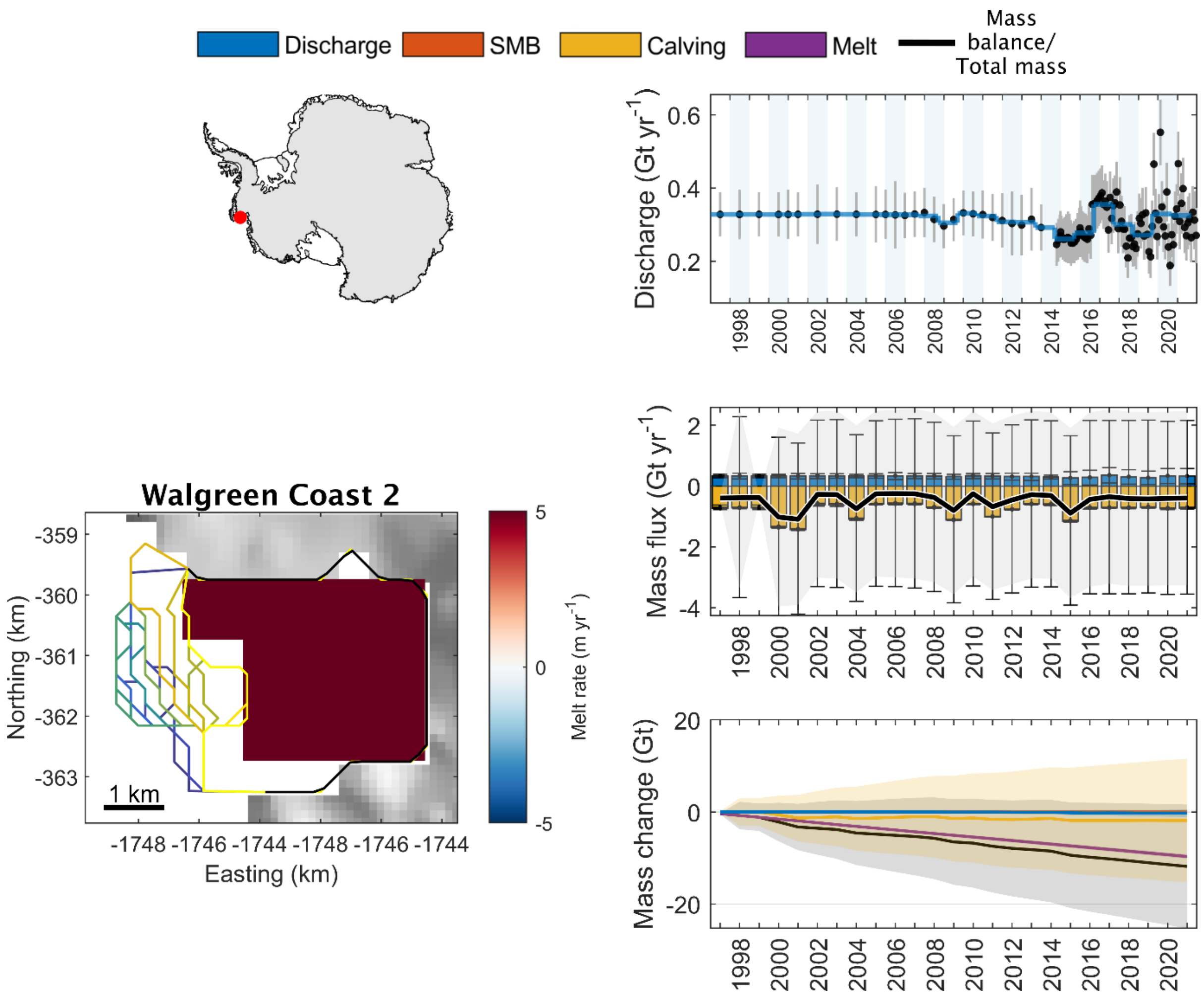
Discharge
SMB
Calving
Melt
Mass balance/ Total mass
Walgreen Coast 2
Northing (km)
-359
-360
-361
-362
-363
1 km
-1748 -1746 -1744 -1748 -1746 -1744
Easting (km)
Melt rate (m yr-1)
5
0
-5
Discharge (Gt yr-1)
0.6
0.4
0.2
Mass flux (Gt yr-1)
2
0
-2
-4
Mass change (Gt)
20
0
-20
1998
2000
2002
2004
2006
2008
2010
2012
2014
2016
2018
2020

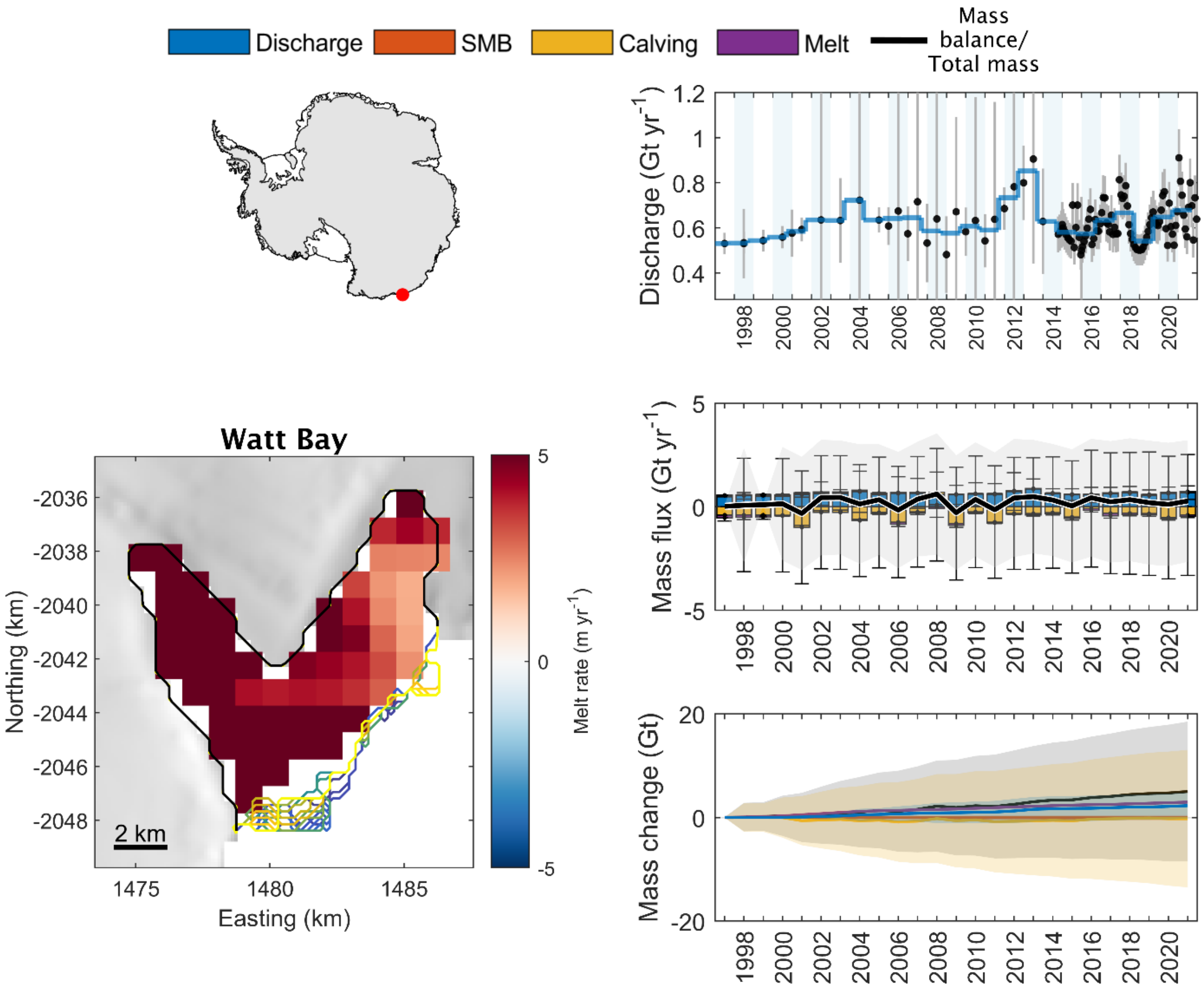

Discharge
SMB
Calving
Melt
Mass balance/ Total mass
Discharge (Gt yr-1)
Watt Bay
Northing (km)
Easting (km)
2 km
Melt rate (m yr-1)
Mass flux (Gt yr-1)
Mass change (Gt)

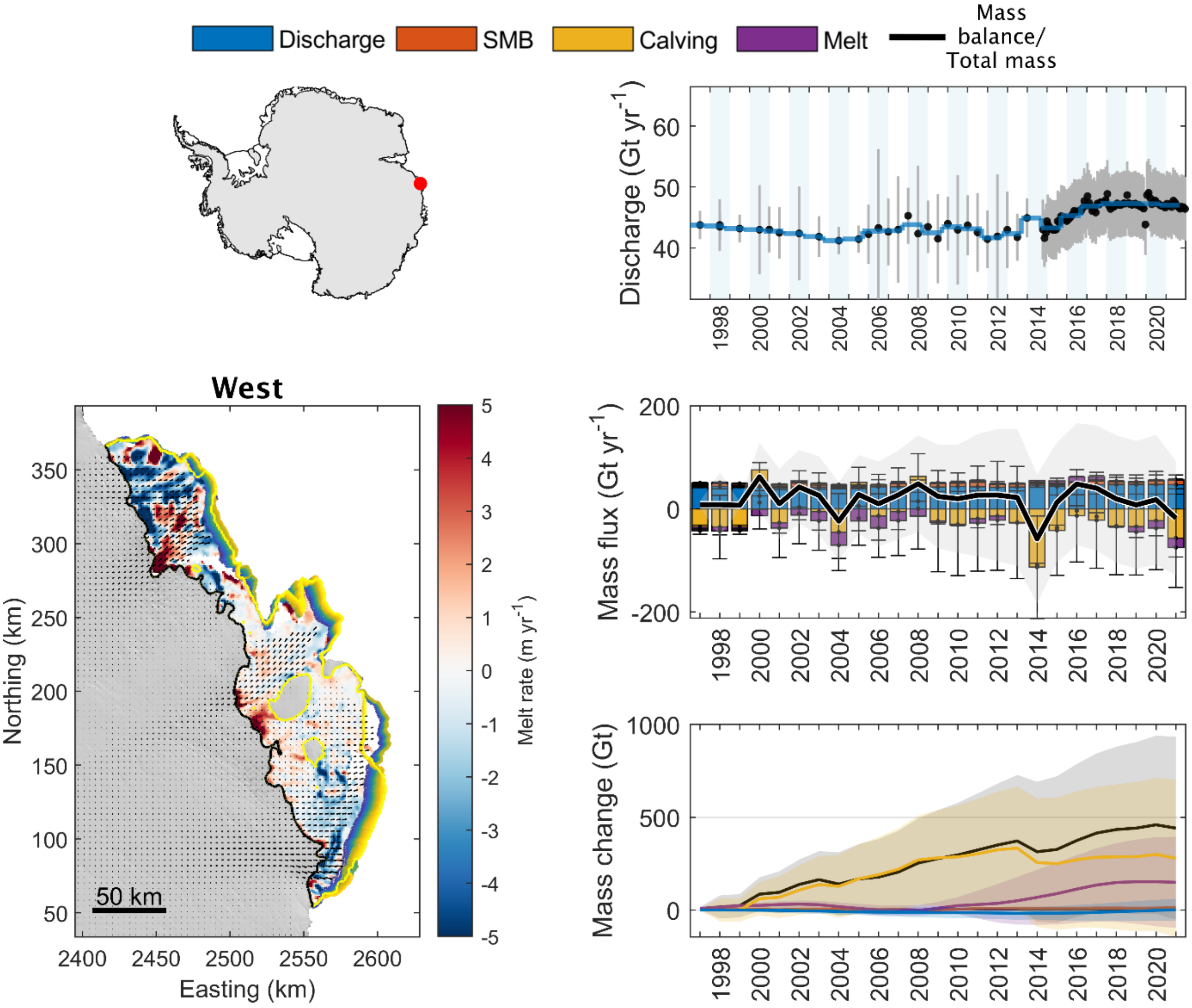
Discharge
SMB
Calving
Melt
Mass balance/ Total mass
Discharge (Gt yr^{-1})
West
Melt rate (m yr^{-1})
Northing (km)
Easting (km)
50 km
Mass flux (Gt yr^{-1})
Mass change (Gt)

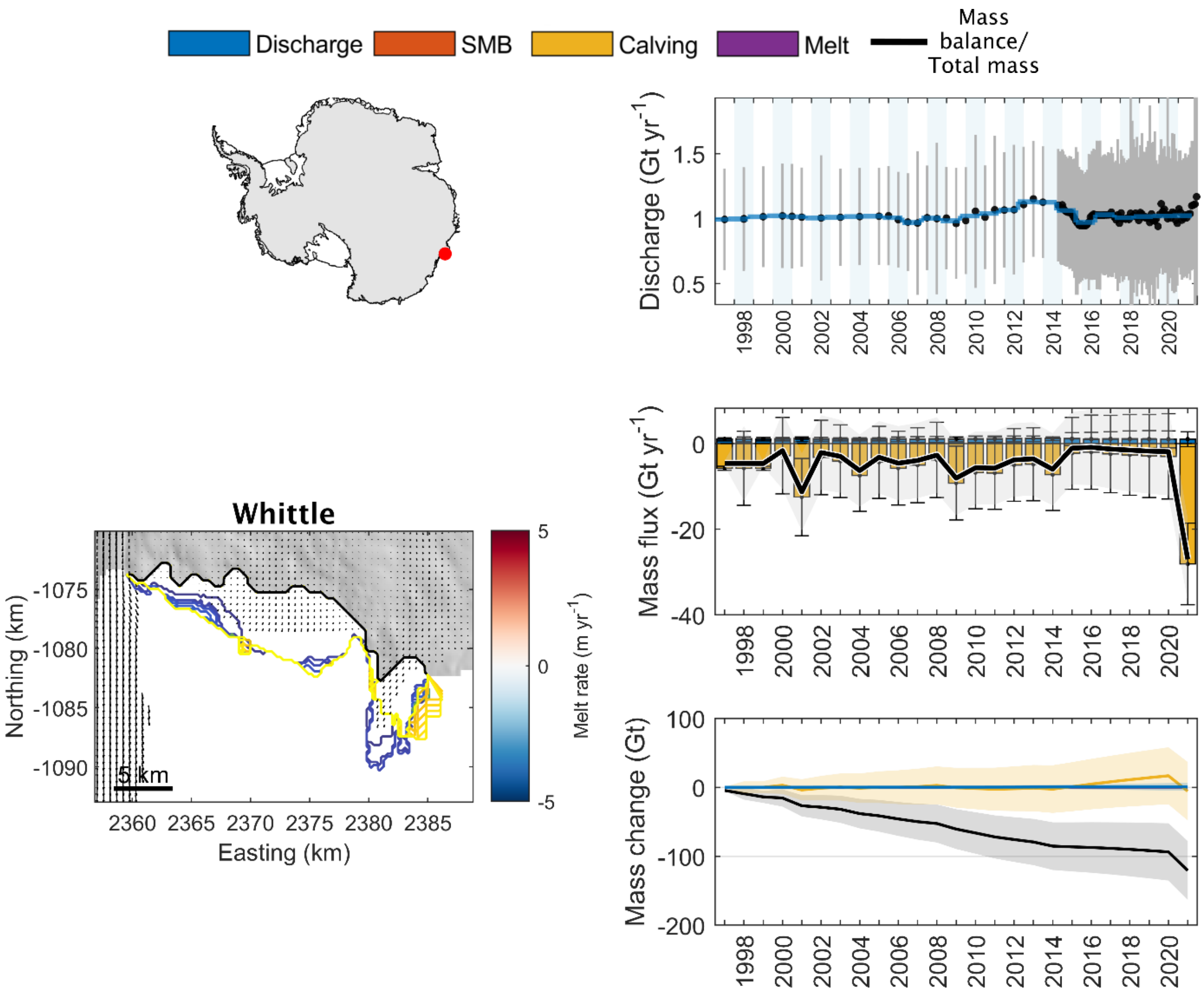
Discharge
SMB
Calving
Melt
Mass balance/ Total mass
Discharge (Gt yr-1)
Whittle
Northing (km)
Easting (km)
5 km
Melt rate (m yr-1)
Mass flux (Gt yr-1)
Mass change (Gt)

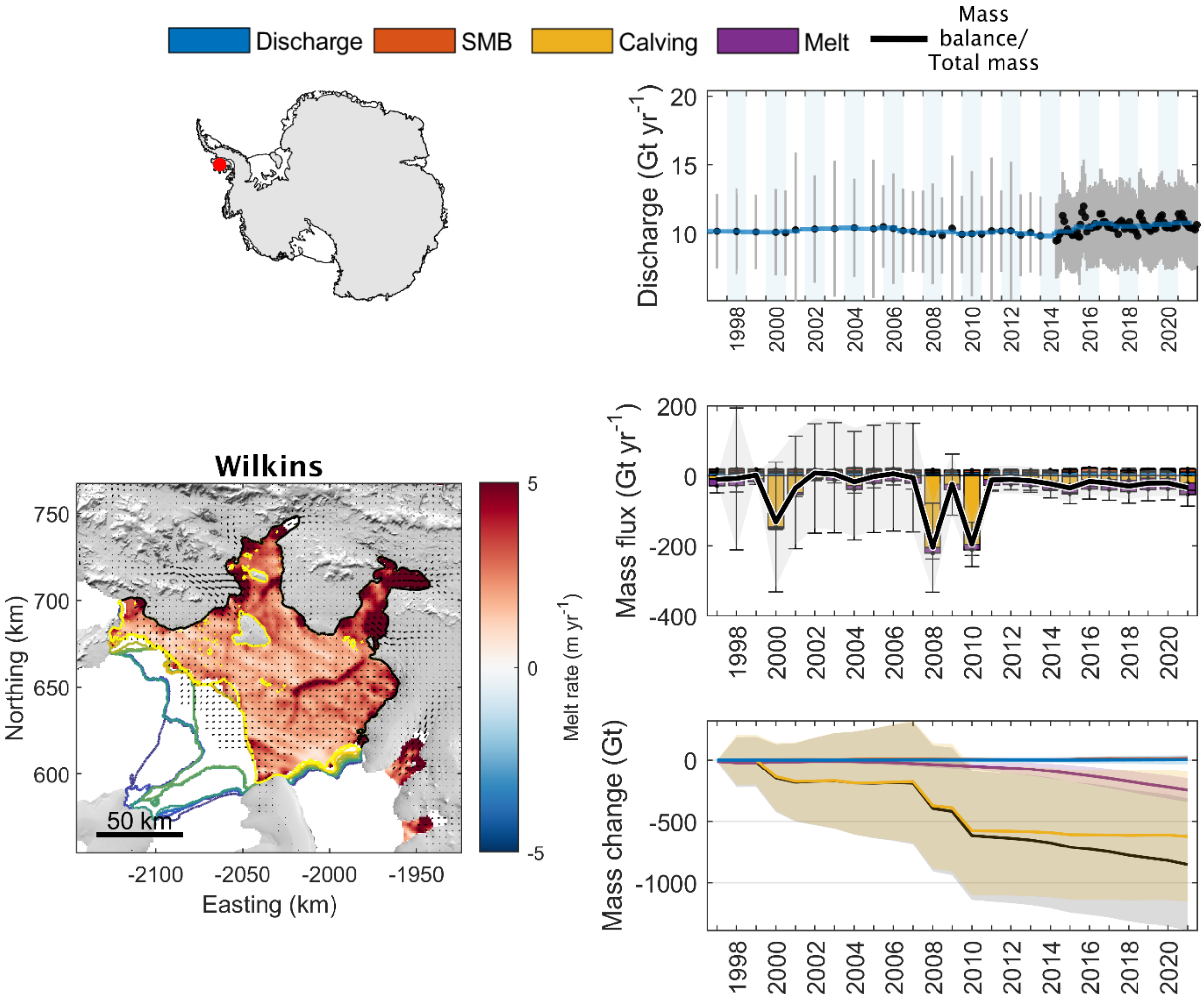
Discharge
SMB
Calving
Melt
Mass balance/ Total mass
Wilkins
50 km
Northing (km)
Easting (km)
Melt rate (m yr-1)
Discharge (Gt yr-1)
Mass flux (Gt yr-1)
Mass change (Gt)

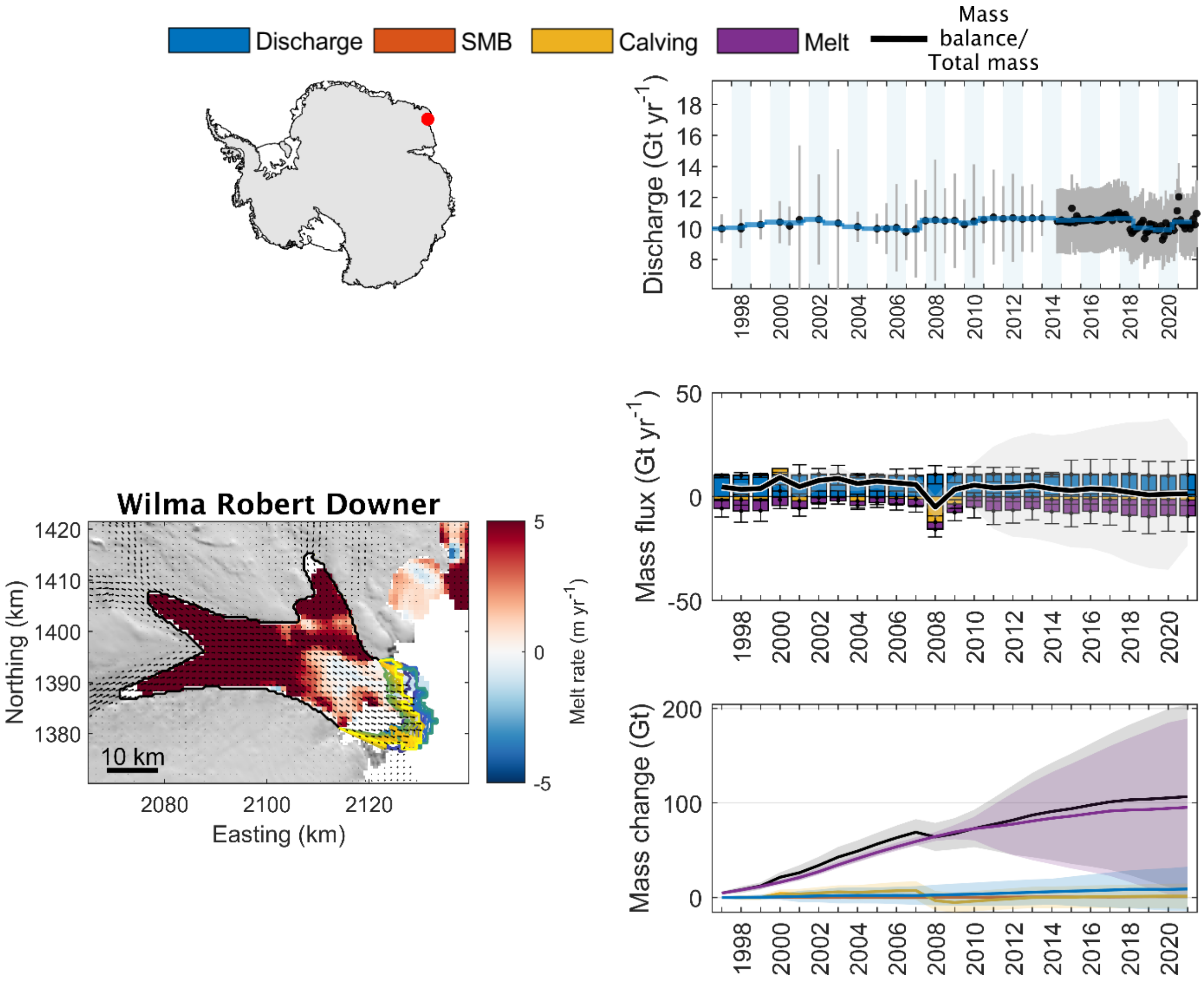
Discharge
SMB
Calving
Melt
Mass balance/ Total mass
Discharge (Gt yr^{-1})
Mass flux (Gt yr^{-1})
Mass change (Gt)
Wilma Robert Downer
Northing (km)
Easting (km)
Melt rate (m yr^{-1})
10 km

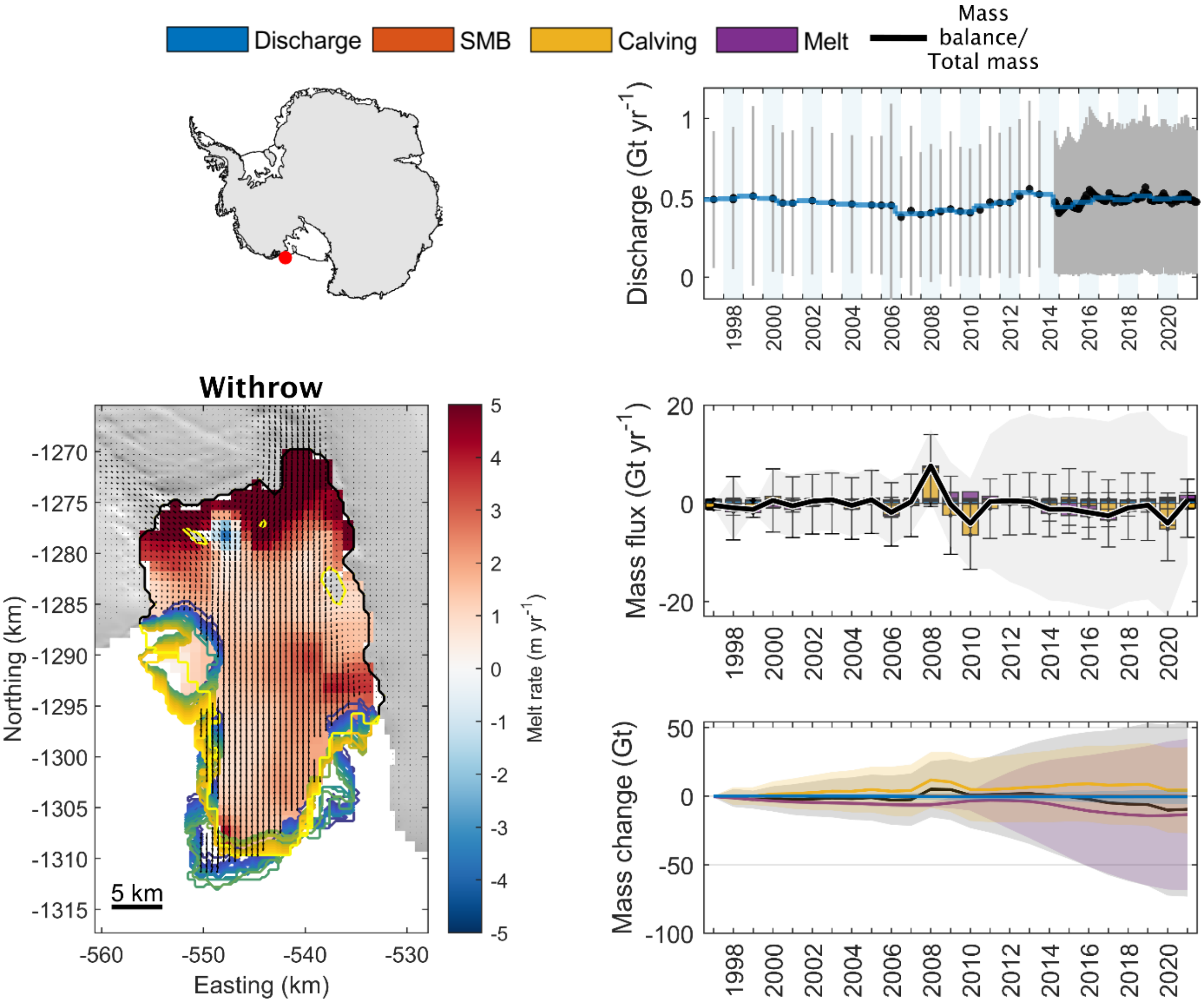
Discharge
SMB
Calving
Melt
Mass balance/ Total mass
Withrow
5 km
Northing (km)
Easting (km)
Melt rate (m yr-1)
Discharge (Gt yr-1)
Mass flux (Gt yr-1)
Mass change (Gt)

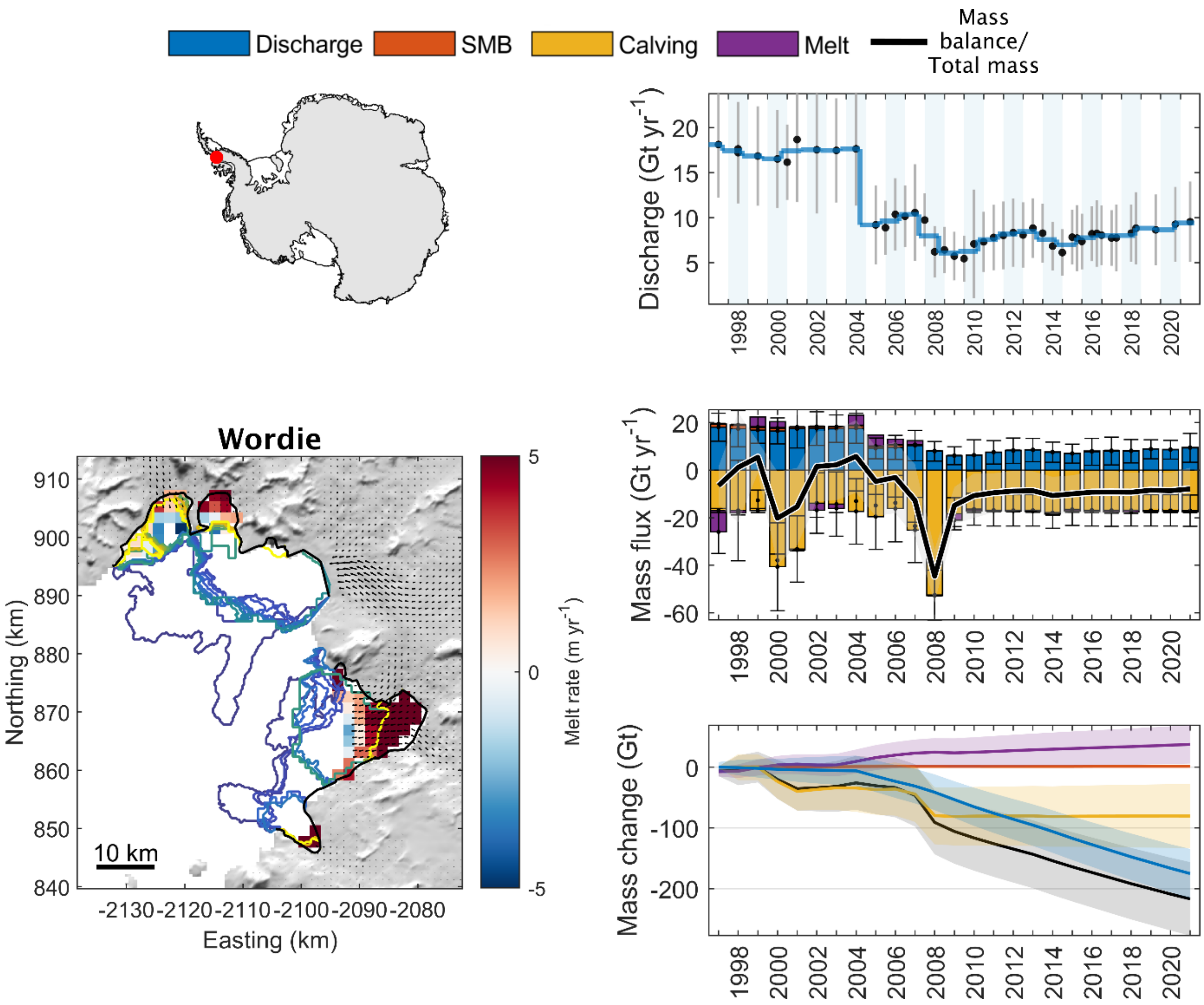
Discharge
SMB
Calving
Melt
Mass balance/ Total mass
Wordie
Northing (km)
Easting (km)
10 km
Melt rate (m yr^{-1})
Discharge (Gt yr^{-1})
Mass flux (Gt yr^{-1})
Mass change (Gt)

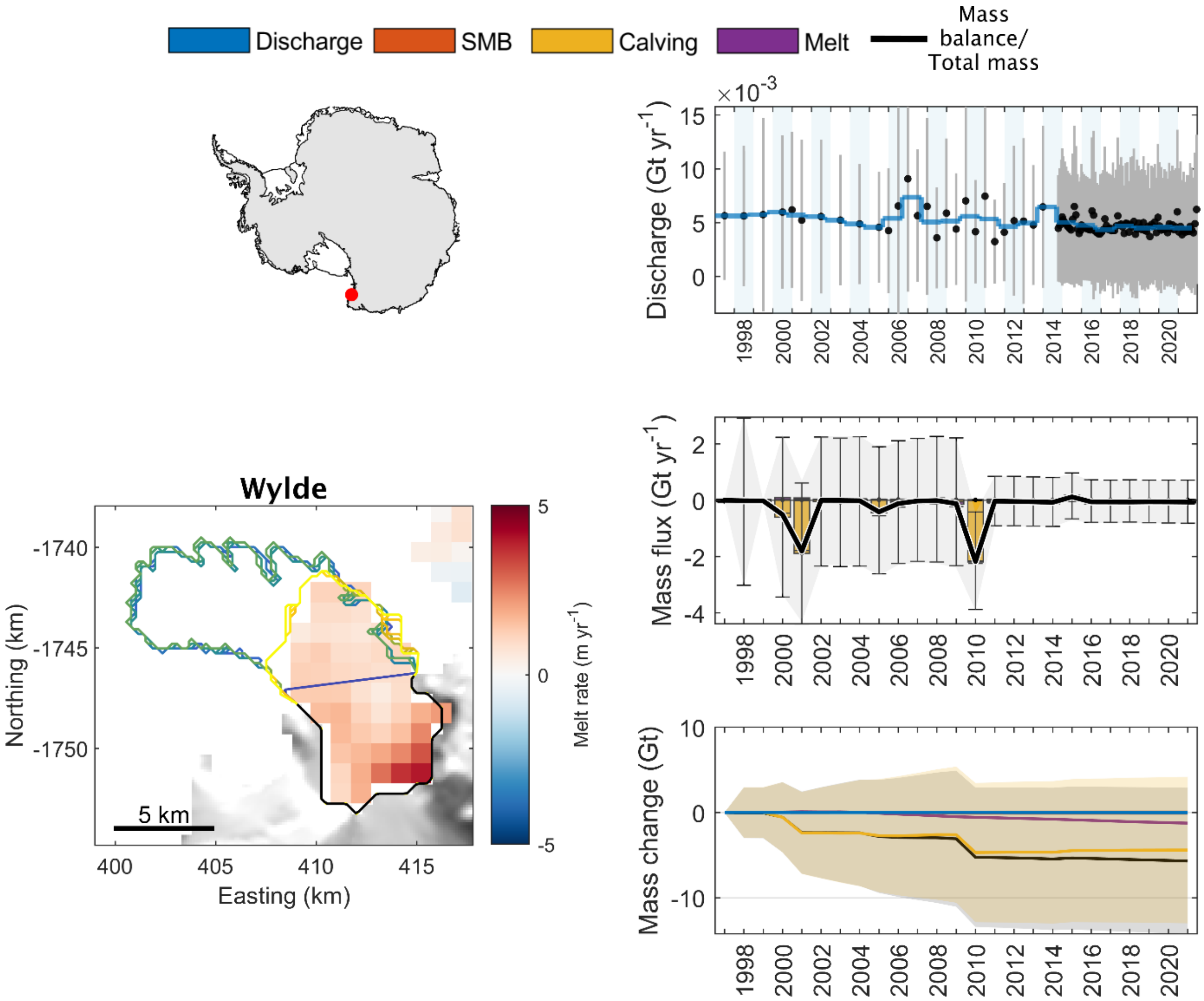

Discharge
SMB
Calving
Melt
Mass balance/ Total mass
Wylde
Northing (km)
Easting (km)
5 km
Melt rate (m yr^{-1})
Discharge (Gt yr^{-1})
Mass flux (Gt yr^{-1})
Mass change (Gt)

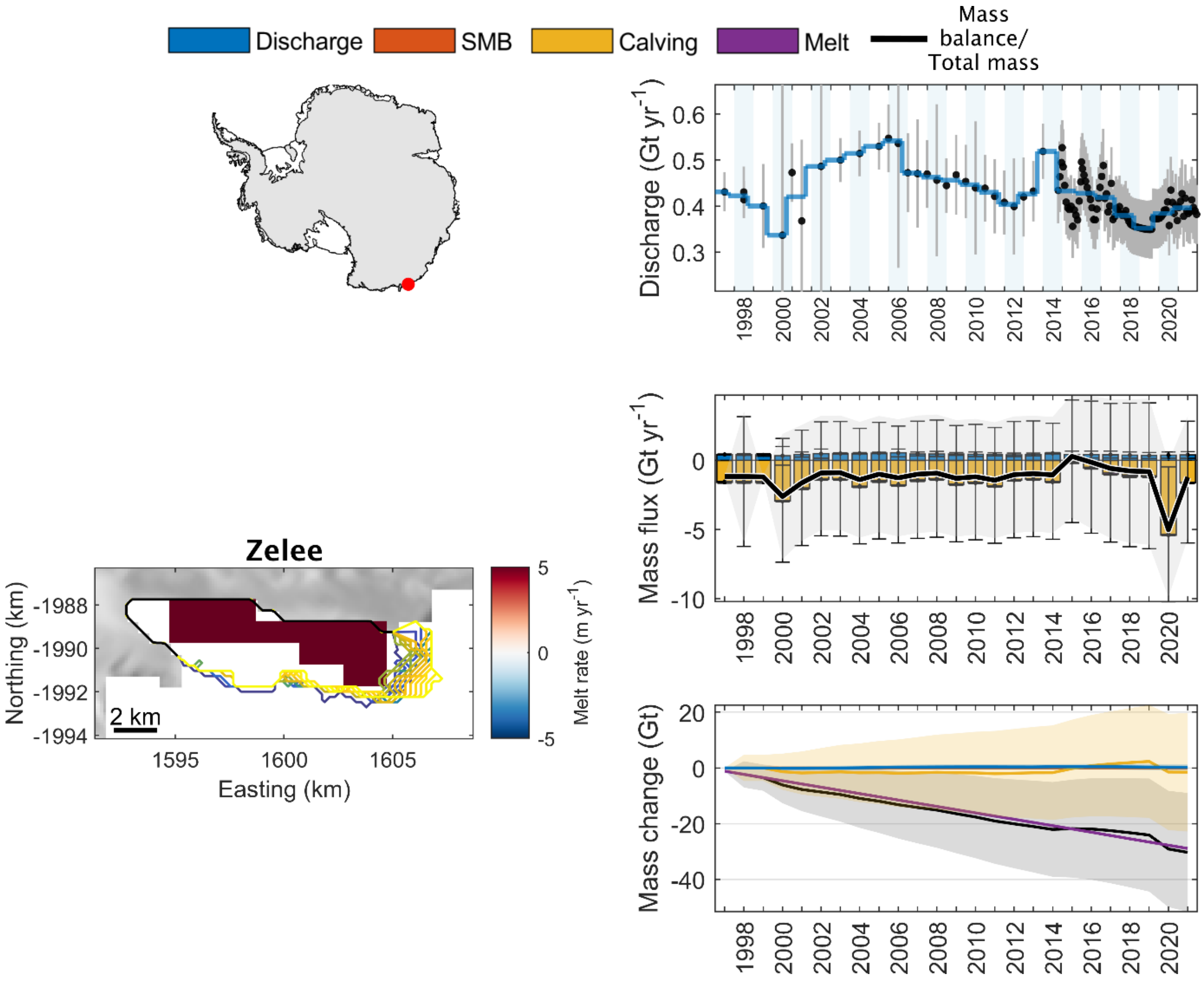
Discharge
SMB
Calving
Melt
Mass balance/ Total mass
Zelee
Northing (km)
-1988
-1990
-1992
-1994
2 km
1595
1600
1605
Easting (km)
5
0
-5
Melt rate (m yr^{-1})
Discharge (Gt yr^{-1})
0.6
0.5
0.4
0.3
Mass flux (Gt yr^{-1})
0
-5
-10
Mass change (Gt)
20
0
-20
-40
1998
2000
2002
2004
2006
2008
2010
2012
2014
2016
2018
2020

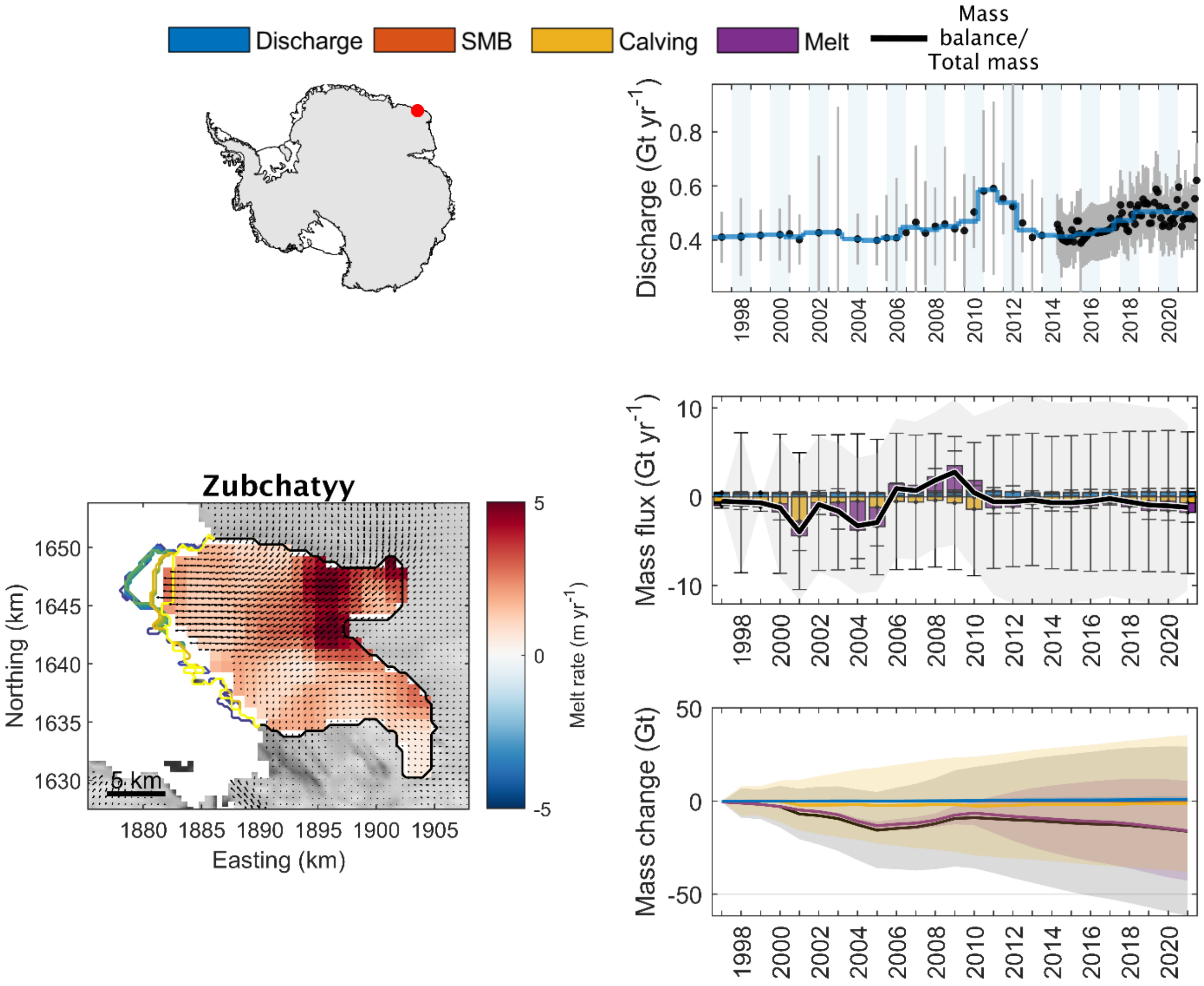
Discharge
SMB
Calving
Melt
Mass balance/ Total mass
Zubchatyy
Northing (km)
Easting (km)
5 km
Melt rate (m yr^{-1})
Discharge (Gt yr^{-1})
Mass flux (Gt yr^{-1})
Mass change (Gt)

Supplementary Table 2. Contains our annual mass budget component data for all ice shelves individually and combined.

REFERENCES AND NOTES

1. E. Rignot, S. Jacobs, J. Mouginot, B. Scheuchl, Ice-shelf melting around Antarctica. *Science* **341**, 266–270 (2013).

2. S. S. Pegler, Marine ice sheet dynamics: The impacts of ice-shelf buttressing. *J. Fluid Mech.* **857**, 605–647 (2018).

3. M. Haseloff, O. V. Sergienko, The effect of buttressing on grounding line dynamics. *J. Glaciol.* **64**, 417–431 (2018).

4. G. H. Gudmundsson, F. S. Paolo, S. Adusumilli, H. A. Fricker, Instantaneous Antarctic ice sheet mass loss driven by thinning ice shelves. *Geophys. Res. Lett.* **46**, 13903–13909 (2019).

5. R. Reese, G. H. Gudmundsson, A. Levermann, R. Winkelmann, The far reach of ice-shelf thinning in Antarctica. *Nat. Clim. Chang.* **8**, 53–57 (2018).

6. T. Mitcham, G. H. Gudmundsson, J. L. Bamber, The instantaneous impact of calving and thinning on the Larsen C Ice Shelf. *Cryosphere* **16**, 883–901 (2022).

7. C. Greene, A. S. Gardner, N.-J. Schlegel, A. D. Fraser, Antarctic calving loss rivals ice-shelf thinning. *Nature* **609**, 948–953 (2022).

8. I. Joughin, D. Shapero, B. Smith, P. Dutrieux, M. Barham, Ice-shelf retreat drives recent Pine Island Glacier speedup. *Sci. Adv.* **7**, eabg3080 (2021).

9. H. Rott, P. Skvarca, T. Nagler, Rapid collapse of northern larsen ice shelf, Antarctica. *Science* **271**, 788–792 (1996).

10. H. Rott, W. Abdel Jaber, J. Wuite, S. Scheiblauer, D. Floricioiu, J. M. Van Wessem, T. Nagler, N. Miranda, M. R. Van Den Broeke, Changing pattern of ice flow and mass balance for glaciers discharging into the Larsen A and B embayments, Antarctic Peninsula, 2011 to 2016. *Cryosphere* **12**, 1273–1291 (2018).

11. A. Jenkins, D. Shoosmith, P. Dutrieux, S. Jacobs, T. W. Kim, S. H. Lee, H. K. Ha, S. Stammerjohn, West Antarctic ice sheet retreat in the Amundsen Sea driven by decadal oceanic variability. *Nat. Geosci.* **11**, 733–738 (2018).

12. P. R. Holland, T. J. Bracegirdle, P. Dutrieux, A. Jenkins, E. J. Steig, West Antarctic ice loss influenced by internal climate variability and anthropogenic forcing. *Nat. Geosci.* **12**, 718–724 (2019).

13. J. Mouginot, E. Rignot, B. Scheuchl, Sustained increase in ice discharge from the Amundsen Sea Embayment, West Antarctica, from 1973 to 2013. *Geophys. Res. Lett.* **41**, 1576–1584 (2014).

14. H. Konrad, L. Gilbert, S. L. Cornford, A. Payne, A. E. Hogg, A. Muir, A. Shepherd, Uneven onset and pace of ice-dynamical imbalance in the Amundsen Sea Embayment, West Antarctica. *Geophys. Res. Lett.* **44**, 910–918 (2017).

15. F. S. Paolo, L. Padman, H. A. Fricker, S. Adusumilli, S. Howard, M. R. Siegfried, Response of Pacific-sector Antarctic ice shelves to the El Niño/Southern Oscillation. *Nat. Geosci.* **11**, 121–126 (2018).

16. C. L. Hulbe, T. A. Scambos, T. Youngberg, A. K. Lamb, Patterns of glacier response to disintegration of the Larsen B ice shelf, Antarctic Peninsula. *Glob. Planet. Change* **63**, 1–8 (2008).

17. E. Rignot, G. Casassa, P. Gogineni, W. Krabill, A. Rivera, R. Thomas, Accelerated ice discharge from the Antarctic Peninsula following the collapse of Larsen B ice shelf. *Geophys. Res. Lett.* **31**, 2–5 (2004).

18. T. A. Scambos, J. A. Bohlander, C. A. Shuman, P. Skvarca, Glacier acceleration and thinning after ice shelf collapse in the Larsen B embayment Antarctica. *Geophys. Res. Lett.* **31**, 10.1029/2004GL020670 (2004).

19. H. Rott, D. Floricioiu, J. Wuite, S. Scheiblauer, T. Nagler, M. Kern, Mass changes of outlet glaciers along the Nordensjköld Coast, northern Antarctic Peninsula, based on TanDEM-X satellite measurements. *Geophys. Res. Lett.* **41**, 8123–8129 (2014).

20. R. M. DeConto, D. Pollard, Contribution of Antarctica to past and future sea-level rise. *Nature* **531**, 591–597 (2016).

21. S. Sun, F. Pattyn, E. G. Simon, T. Albrecht, S. Cornford, R. Calov, C. Dumas, F. Gillet-Chaulet, H. Goelzer, N. R. Golledge, R. Greve, M. J. Hoffman, A. Humbert, E. Kazmierczak, T. Kleiner, G. R. Leguy, W. H. Lipscomb, D. Martin, M. Morlighem, S. Nowicki, D. Pollard, S. Price, A. Quiquet, H. Seroussi, T. Schlemm, J. Sutter, R. S. W. Van De Wal, R. Winkelmann, T. Zhang, Antarctic ice sheet response to sudden and sustained ice-shelf collapse (ABUMIP). *J. Glaciol.* **66**, 891–904 (2020).

22. R. E. Bell, A. F. Banwell, L. D. Trusel, J. Kingslake, Antarctic surface hydrology and impacts on ice-sheet mass balance. *Nat. Clim. Chang.* **8**, 1044–1052 (2018).

23. C. R. Stokes, J. E. Sanderson, B. W. J. Miles, S. S. R. Jamieson, A. A. Leeson, Widespread distribution of supraglacial lakes around the margin of the East Antarctic Ice Sheet. *Sci. Rep.* **9**, 13823 (2019).

24. J. Kingslake, J. C. Ely, I. Das, R. E. Bell, Widespread movement of meltwater onto and across Antarctic ice shelves. *Nature* **544**, 349–352 (2017).

25. P. A. Tuckett, J. C. Ely, A. J. Sole, J. M. Lea, S. J. Livingstone, J. M. Jones, J. M. Van Wessem, Automated mapping of the seasonal evolution of surface meltwater and its links to climate on the Amery Ice Shelf, Antarctica. *Cryosphere* **15**, 5785–5804 (2021).

26. R. L. Dell, A. F. Banwell, I. C. Willis, N. S. Arnold, A. R. W. Halberstadt, T. R. Chudley, H. D. Pritchard, Supervised classification of slush and ponded water on Antarctic ice shelves using Landsat 8 imagery. *J. Glaciol.* **68**, 401–414 (2022).

27. A. F. Banwell, I. C. Willis, G. J. Macdonald, B. Goodsell, D. R. MacAyeal, Direct measurements of ice-shelf flexure caused by surface meltwater ponding and drainage. *Nat. Commun.* **10**, 730 (2019).

28. T. Scambos, H. A. Fricker, C. C. Liu, J. Bohlander, J. Fastook, A. Sargent, R. Massom, A. M. Wu, Ice shelf disintegration by plate bending and hydro-fracture: Satellite observations and model results of the 2008 Wilkins ice shelf break-ups. *Earth Planet. Sci. Lett.* **280**, 51–60 (2009).

29. W. Rack, H. Rott, Pattern of retreat and disintegration of the Larsen B ice shelf, Antarctic Peninsula. *Ann. Glaciol.* **39**, 505–510 (2004).

30. L. D. Trusel, K. E. Frey, S. B. Das, K. B. Karnauskas, P. Kuipers Munneke, E. Van Meijgaard, M. R. Van Den Broeke, Divergent trajectories of Antarctic surface melt under two twenty-first-century climate scenarios. *Nat. Geosci.* **8**, 927–932 (2015).

31. C. Y. Lai, J. Kingslake, M. G. Wearing, P. H. C. Chen, P. Gentine, H. Li, J. J. Spergel, J. M. van Wessem, Vulnerability of Antarctica's ice shelves to meltwater-driven fracture. *Nature* **584**, 574–578 (2020).

32. S. Adusumilli, H. A. Fricker, B. Medley, L. Padman, M. R. Siegfried, Interannual variations in meltwater input to the Southern Ocean from Antarctic ice shelves. *Nat. Geosci.* **13**, 616–620 (2020).

33. Y. Liu, J. C. Moore, X. Cheng, R. M. Gladstone, J. N. Bassis, H. Liu, J. Wen, F. Hui, Ocean-driven thinning enhances iceberg calving and retreat of Antarctic ice shelves. *Proc. Natl. Acad. Sci. U.S.A.* **112**, 3263–3268 (2015).

34. K. E. Alley, T. A. Scambos, R. B. Alley, N. Holschuh, Troughs developed in ice-stream shear margins precondition ice shelves for ocean-driven breakup. *Sci. Adv.* **5**, eaax2215 (2019).

35. R. A. Massom, T. A. Scambos, L. G. Bennetts, P. Reid, V. A. Squire, S. E. Stammerjohn, Antarctic ice shelf disintegration triggered by sea ice loss and ocean swell. *Nature* **558**, 383–389 (2018).

36. F. D. W. Christie, T. J. Benham, C. L. Batchelor, W. Rack, A. Montelli, J. A. Dowdeswell, Antarctic ice-shelf advance driven by anomalous atmospheric and sea-ice circulation. *Nat. Geosci.* **15**, 356–362 (2022).

37. Y. Nakayama, R. Timmermann, H. H. Hellmer, Impact of West Antarctic ice shelf melting on Southern Ocean hydrography. *Cryosphere* **14**, 2205–2216 (2020).

38. M. R. England, T. J. W. Wagner, I. Eisenman, Modeling the breakup of tabular icebergs. *Sci. Adv.* **6**, eabd1273 (2020).

39. R. Bintanja, G. J. Van Oldenborgh, S. S. Drijfhout, B. Wouters, C. A. Katsman, Important role for ocean warming and increased ice-shelf melt in Antarctic sea-ice expansion. *Nat. Geosci.* **6**, 376–379 (2013).

40. G. D. Williams, L. Herraiz-Borreguero, F. Roquet, T. Tamura, K. I. Ohshima, Y. Fukamachi, A. D. Fraser, L. Gao, H. Chen, C. R. McMahon, R. Harcourt, M. Hindell, The suppression of Antarctic bottom water formation by melting ice shelves in Prydz Bay. *Nat. Commun.* **7**, 12577 (2016).

41. Q. Li, M. H. England, A. M. Hogg, S. R. Rintoul, A. K. Morrison, Abyssal ocean overturning slowdown and warming driven by Antarctic meltwater. *Nature* **615**, 841–847 (2023).

42. A. T. Bradley, D. T. Bett, P. Dutrieux, J. De Rydt, P. R. Holland, The influence of pine island ice shelf calving on basal melting. *J. Geophys. Res. Ocean.* **127**, e2022JC018621 (2022).

43. M. A. Depoorter, J. L. Bamber, J. A. Griggs, J. T. M. Lenaerts, S. R. M. Ligtenberg, M. R. Van Den Broeke, G. Moholdt, Calving fluxes and basal melt rates of Antarctic ice shelves. *Nature* **502**, 89–92 (2013).

44. A. G. Pauling, C. M. Bitz, I. J. Smith, P. J. Langhorne, The response of the Southern Ocean and Antarctic sea ice to freshwater from ice shelves in an earth system model. *J. Climate* **29**, 1655–1672 (2016).

45. F. S. Paolo, H. A. Fricker, L. Padman, Volume loss from Antarctic ice shelves is accelerating. *Science* **348**, 327–331 (2015).

46. F. S. Paolo, A. S. Gardner, C. A. Greene, J. N. Nilsson, M. P. Schodlok, J. Schlegel, H. A. Fricker, Widespread slowdown in thinning rates of West Antarctic Ice Shelves. *Cryosph. Discuss.* (2022).

47. T. C. Sutterley, T. Markus, T. A. Neumann, M. Van Den Broeke, J. M. Van Wessem, S. R. M. Ligtenberg, Antarctic ice shelf thickness change from multimission lidar mapping. *Cryosphere* **13**, 1801–1817 (2019).

48. A. E. Hogg, L. Gilbert, A. Shepherd, A. S. Muir, M. McMillan, Extending the record of Antarctic ice shelf thickness change, from 1992 to 2017. *Adv. Sp. Res.* **68**, 724–731 (2021).

49. H. D. Pritchard, S. R. M. Ligtenberg, H. A. Fricker, D. G. Vaughan, M. R. Van Den Broeke, L. Padman, Antarctic ice-sheet loss driven by basal melting of ice shelves. *Nature* **484**, 502–505 (2012).

50. M. Qi, Y. Liu, J. Liu, X. Cheng, Y. Lin, Q. Feng, A 15-year circum-Antarctic iceberg calving dataset derived from continuous satellite observations. *Earth Syst. Sci. Data* **13**, 4583–4601 (2021).

51. H. L. Andreasen, J. Hogg, A. E. Selley, Change in area of Antarctic Ice Shelves from 2009 to 2019. *Cryosph. Discuss.* (2022).

52. C. A. Baumhoer, A. J. Dietz, C. Kneisel, H. Paeth, C. Kuenzer, Environmental drivers of circum-Antarctic glacier and ice shelf front retreat over the last two decades. *Cryosphere* **15**, 2357–2381 (2021).

53. B. W. J. Miles, C. R. Stokes, S. S. R. Jamieson, J. R. Jordan, G. H. Gudmundsson, A. Jenkins, High spatial and temporal variability in Antarctic ice discharge linked to ice shelf buttressing and bed geometry. *Sci. Rep.* **12**, 10968 (2022).

54. A. S. Gardner, G. Moholdt, T. Scambos, M. Fahnstock, S. Ligtenberg, M. Van Den Broeke, J. Nilsson, Increased West Antarctic and unchanged East Antarctic ice discharge over the last 7 years. *Cryosphere* **12**, 521–547 (2018).

55. P. Milillo, E. Rignot, P. Rizzoli, B. Scheuchl, J. Mouginot, J. L. Bueso-Bello, P. Prats-Iraola, L. Dini, Rapid glacier retreat rates observed in West Antarctica. *Nat. Geosci.* **15**, 48–53 (2022).

56. E. Rignot, J. Mouginot, M. Morlighem, H. Seroussi, B. Scheuchl, Widespread, rapid grounding line retreat of Pine Island, Thwaites, Smith, and Kohler glaciers, West Antarctica, from 1992 to 2011. *Geophys. Res. Lett.* **41**, 3502–3509 (2014).

57. D. I. Benn, J. A. Åström, Calving glaciers and ice shelves. *Adv. Phys. X* **3**, 1513819 (2018).

58. N. C. Jourdain, X. Asay-davis, T. Hattermann, F. Straneo, H. Seroussi, A protocol for calculating basal melt rates in the ISMIP6 Antarctic ice sheet projections. *Cryosph.* **14**, 3111–3134 (2020).

59. F. S. Paolo, A. S. Gardner, C. A. Greene, N. J. Schlegel, *MEaSUREs ITS_LIVE Antarctic Ice Shelf Height Change and Basal Melt Rates, Version 1* (National Aeronautics and Space Administration, 2022); https://doi.org/10.5067/SE3XH9RXQWAM.

60. N. Gourmelen, D. N. Goldberg, K. Snow, S. F. Henley, R. G. Bingham, S. Kimura, A. E. Hogg, A. Shepherd, J. Mouginot, J. T. M. Lenaerts, S. R. M. Ligtenberg, W. J. van de Berg, Channelized melting drives thinning under a rapidly melting antarctic ice shelf. *Geophys. Res. Lett.* **44**, 9796–9804 (2017).

61. N. Gourmelen, M. J. Escorihuela, A. Shepherd, L. Foresta, A. Muir, A. Garcia-Mondéjar, M. Roca, S. G. Baker, M. R. Drinkwater, CryoSat-2 swath interferometric altimetry for mapping ice elevation and elevation change. *Adv. Sp. Res.* **62**, 1226–1242 (2018).

62. J. M. van Wessem, W. J. van de Berg, B. P. Y. Noël, E. van Meijgaard, G. Birnbaum, C. L. Jakobs, K. Krüger, J. T. M. Lenaerts, S. Lhermitte, S. R. M. Ligtenberg, B. Medley, C. H. Reijmer, K. van Tricht, L. D. Trusel, L. H. van Ulft, B. Wouters, J. Wuite, M. R. van den Broeke, Modelling the climate and surface mass balance of polar ice sheets using RACMO2 – Part 2: Antarctica (1979-2016). *Cryosphere* **12**, 1479–1498 (2018).

63. C. Agosta, C. Amory, C. Kittel, A. Orsi, V. Favier, H. Gallée, M. R. Van Den Broeke, J. T. M. Lenaerts, J. M. Van Wessem, W. J. Van De Berg, X. Fettweis, Estimation of the Antarctic surface mass balance using the regional climate model MAR (1979-2015) and identification of dominant processes. *Cryosphere* **13**, 281–296 (2019).

64. C. Kittel, C. Amory, C. Agosta, A. Delhasse, S. Doutreloup, P. V. Huot, C. Wyard, T. Fichefet, X. Fettweis, Sensitivity of the current Antarctic surface mass balance to sea surface conditions using MAR. *Cryosphere* **12**, 3827–3839 (2018).

65. N. Hansen, P. L. Langen, F. Boberg, R. Forsberg, S. B. Simonsen, P. Thejll, B. Vandecrux, R. Mottram, Downscaled surface mass balance in Antarctica: Impacts of subsurface processes and large-scale atmospheric circulation. *Cryosphere* **15**, 4315–4333 (2021).

66. L. Libert, J. Wuite, T. Nagler, Automatic delineation of cracks with Sentinel-1 interferometry for monitoring ice shelf damage and calving. *Cryosphere* **16**, 1523–1542 (2022).

67. J. Wuite, T. Nagler, N. Gourmelen, M. J. Escorihuela, A. E. Hogg, M. R. Drinkwater, Sub-annual calving front migration, area change and calving rates from swath mode CryoSat-2. *Remote Sens. (Basel)* **11**, 2761 (2019).

68. D. R. Macayeal, L. Padman, M. R. Drinkwater, M. Fahnestock, T. T. Gotis, L. *Gray, Effects of Rigid Body Collisions and Tide-Forced Drift on Large Tabular Icebergs of the Antarctic* (2002); http//geosci.uchicago.edu/~drm7/research/Icebergs_of_Y2k.pdf [accessed 23 July 2019].

69. B. W. J. Miles, C. R. Stokes, A. Jenkins, J. R. Jordan, S. S. R. Jamieson, G. H. Gudmundsson, Intermittent structural weakening and acceleration of the Thwaites Glacier Tongue between 2000 and 2018. *J. Glaciol.* **66**, 485–495 (2020).

70. R. A. Massom, A. Giles, R. C. Warner, H. A. Fricker, B. Legrésy, G. Hyland, L. Lescarmontier, N. Young, External influences on the Mertz Glacier Tongue (East Antarctica) in the decade leading up to its calving in 2010. *J. Geophys. Res. Earth Surf.* **120**, 490–506 (2015).

71. A. E. Hogg, G. H. Gudmundsson, Impacts of the Larsen-C ice shelf calving event. *Nat. Clim. Chang.* **7**, 540–542 (2017).

72. J. J. Fürst, G. Durand, F. Gillet-Chaulet, L. Tavard, M. Rankl, M. Braun, O. Gagliardini, The safety band of Antarctic ice shelves. *Nat. Clim. Chang.* **6**, 479–482 (2016).

73. J. De Rydt, R. Reese, F. S. Paolo, G. H. Gudmundsson, Drivers of pine island glacier speed-up between 1996 and 2016. *Cryosphere* **15**, 113–132 (2021).

74. D. N. Goldberg, P. Heimbach, I. Joughin, B. Smith, Committed retreat of Smith, Pope, and Kohler Glaciers over the next 30 years inferred by transient model calibration. *Cryosphere* **9**, 2429–2446 (2015).

75. F. Pattyn, A. Huyghe, S. De Brabander, B. De Smedt, Role of transition zones in marine ice sheet dynamics. *J. Geophys. Res. Earth Surf.* **111**, (2006).

76. D. Goldberg, D. M. Holland, C. Schoof, Grounding line movement and ice shelf buttressing in marine ice sheets. *J. Geophys. Res. Earth Surf.* **114**, (2009).

77. M. Donat-Magnin, N. C. Jourdain, P. Spence, J. Le Sommer, H. Gallée, G. Durand, Ice-shelf melt response to changing winds and glacier dynamics in the amundsen sea sector, Antarctica. *J. Geophys. Res. Ocean.* **122**, 10206–10224 (2017).

78. A. Aschwanden, T. C. Bartholomaus, D. J. Brinkerhoff, M. Truffer, Brief communication: A roadmap towards credible projections of ice sheet contribution to sea level. *Cryosphere* **15**, 5705–5715 (2021).

79. M. Morlighem, E. Rignot, T. Binder, D. Blankenship, R. Drews, G. Eagles, O. Eisen, F. Ferraccioli, R. Forsberg, P. Fretwell, V. Goel, J. S. Greenbaum, H. Gudmundsson, J. Guo, V. Helm, C. Hofstede, I. Howat, A. Humbert, W. Jokat, N. B. Karlsson, W. S. Lee, K. Matsuoka, R. Millan, J. Mouginot, J. Paden, F. Pattyn, J. Roberts, S. Rosier, A. Ruppel, H. Seroussi, E. C. Smith, D. Steinhage, B. Sun, M. R. van den Broeke, T. D. van Ommen, M. van Wessem, D. A. Young, Deep glacial troughs and stabilizing ridges unveiled beneath the margins of the Antarctic ice sheet. *Nat. Geosci.* **13**, 132–137 (2020).

80. J. Mouginot, B. Scheuchl, E. Rignot, *MEaSUREs Antarctic Boundaries for IPY 2007–2009 from Satellite Radar, Version 2* (NASA National Snow and Ice Data Center Distributed Active Archive Center, 2017); https://doi.org/10.5067/AXE4121732AD.

81. S. Lhermitte, S. Sun, C. Shuman, B. Wouters, F. Pattyn, J. Wuite, E. Berthier, T. Nagler, Damage accelerates ice shelf instability and mass loss in Amundsen Sea Embayment. *Proc. Natl. Acad. Sci. U.S.A.* **117**, 24735–24741 (2020).

82. J. A. MacGregor, G. A. Catania, M. S. Markowski, A. G. Andrews, Widespread rifting and retreat of ice-shelf margins in the eastern Amundsen Sea Embayment between 1972 and 2011. *J. Glaciol.* **58**, 458–466 (2012).

83. J. Mouginot, E. Rignot, B. Scheuchl, R. Millan, Comprehensive annual ice sheet velocity mapping using Landsat-8, Sentinel-1, and RADARSAT-2 data. *Remote Sens.* **9**, 364 (2017).

84. J. Mouginot, B. Scheuchl, E. Rignot, *MEaSUREs Annual Antarctic Ice Velocity Maps, Version 1* (NASA National Snow and Ice Data Center Distributed Active Archive Center, 2017); https://doi.org/10.5067/9T4EPQXTJYW9.

85. E. Rignot, B. Scheuchl, J. Mouginot, *MEaSUREs Multi-year Reference Velocity Maps of the Antarctic Ice Sheet, Version 1.* (NASA National Snow and Ice Data Center Distributed Active Archive Center, 2022); https://doi.org/10.5067/FB851ZIZYX5O.

86. ENVEO, J. Wuite, M. Hetzenecker, T. Nagler, S. Scheiblauer, *ESA Antarctic Ice Sheet Climate Change Initiative (Antarctic_Ice_Sheet_cci): Antarctic Ice Sheet Monthly Velocity from 2017 to 2020, Derived from Sentinel-1, v1* (NERC EDS Centre for Environmental Data Analysis, 2021); https://dx.doi.org/10.5285/00fe090efc58446e8980992a617f632f.

87. E. Rignot, J. Mouginot, B. Scheuchl, *MEaSUREs InSAR-Based Ice Velocity of the Amundsen Sea Embayment, Antarctica, Version 1* (NASA National Snow and Ice Data Center Distributed Active Archive Center, 2014); https://doi.org/10.5067/MEASURES/CRYOSPHERE/nsidc-0545.001.

88. H. L. Selley, A. E. Hogg, S. Cornford, P. Dutrieux, A. Shepherd, J. Wuite, D. Floricioiu, A. Kusk, T. Nagler, L. Gilbert, T. Slater, T.-W. Kim, Widespread increase in dynamic imbalance in the Getz region of Antarctica from 1994 to 2018. *Nat. Commun.* **12**, 1133 (2021).

89. K. D. Mankoff, A. Solgaard, W. Colgan, A. P. Ahlstrøm, S. Abbas Khan, R. S. Fausto, Greenland Ice Sheet solid ice discharge from 1986 through March 2020. *Earth Syst. Sci. Data* **12**, 1367–1383 (2019).

90. M. Morlighem, *MEaSUREs BedMachine Antarctica, Version 2* (NASA National Snow and Ice Data Center Distributed Active Archive Center, 2020); https://doi.org/10.5067/E1QL9HFQ7A8M

91. X. Cui, H. Jeofry, J. S. Greenbaum, J. Guo, L. Li, L. E. Lindzey, F. A. Habbal, W. Wei, D. A. Young, N. Ross, M. Morlighem, L. M. Jong, J. L. Roberts, D. D. Blankenship, S. Bo, M. J. Siegert, Bed topography of Princess Elizabeth Land in East Antarctica. *Earth Syst. Sci. Data* **12**, 2765–2774 (2020).

92. M. Huss, D. Farinotti, A high-resolution bedrock map for the Antarctic Peninsula. *Cryosphere* **8**, 1261–1273 (2014).

93. I. M. Howat, C. Porter, B. E. Smith, M. J. Noh, P. Morin, The reference elevation model of antarctica. *Cryosphere* **13**, 665–674 (2019).

94. J. Nilsson, A. S. Gardner, F. S. Paolo, Elevation change of the Antarctic Ice Sheet: 1985 to 2020. *Earth Syst. Sci. Data* **14**, 3573–3598 (2022).

95. S. R. M. Ligtenberg, M. M. Helsen, M. R. Van Den Broeke, An improved semi-empirical model for the densification of Antarctic firn. *Cryosphere* **5**, 809–819 (2011).

96. S. B. M. Veldhuijsen, W. J. van de Berg, M. Brils, P. K. Munneke, M. R. van den Broeke, Characteristics of the contemporary Antarctic firn layer simulated with IMAU-FDM v1.2A (1979-2020). *Cryosphere Discuss.* (2022).

97. B. Smith, H. A. Fricker, A. S. Gardner, B. Medley, J. Nilsson, F. S. Paolo, N. Holschuh, S. Adusumilli, K. Brunt, B. Csatho, K. Harbeck, T. Markus, T. Neumann, M. R. Siegfried, H. J. Zwally, Pervasive ice sheet mass loss reflects competing ocean and atmosphere processes. *Science* **368**, 1239–1242 (2020).

98. E. Rignot, J. Mouginot, B. Scheuchl, M. Van Den Broeke, M. J. Van Wessem, M. Morlighem, Four decades of Antarctic ice sheet mass balance from 1979–2017. *Proc. Natl. Acad. Sci. U.S.A.* **116**, 1095–1103 (2019).

99. A. S. Gardner, M. A. Fahnestock, T. A. Scambos, MEaSURes *ITS_LIVE Regional Glacier and Ice Sheet Surface Velocities, Version 1* (NASA National Snow and Ice Data Center Distributed Active Archive Center, 2019); https://doi.org/10.5067/6II6VW8LLWJ7.

100. T. Slater, I. R. Lawrence, I. N. Otosaka, A. Shepherd, N. Gourmelen, L. Jakob, P. Tepes, L. Gilbert, P. Nienow, Review article: Earth's ice imbalance. *Cryosphere* **15**, 233–246 (2021).

101. M. van den Broeke, J. Bamber, J. Ettema, E. Rignot, E. Schrama, W. J. van de Berg, E. van Meijgaard, I. Velicogna, B. Wouters, Partitioning recent Greenland mass loss. *Science* **326**, 984–986 (2009).

102. T. Haran, M. Klinger, J. Bohlander, M. Fahnestock, T. Painter, T. Scambos, *MEaSUREs MODIS Mosaic of Antarctica 2013-2014 (MOA2014) Image Map, Version 1* (NASA National Snow and Ice Data Center Distributed Active Archive Center, 2018); https://doi.org/10.5067/RNF17BP824UM.

103. E. Rignot, J. Mouginot, B. Scheuchl, *MEaSUREs InSAR-Based Antarctica Ice Velocity Map, Version 2*. ((NASA National Snow and Ice Data Center Distributed Active Archive Center, 2017); https://doi.org/10.5067/D7GK8F5J8M8R.

104. A. Shepherd, D. Wingham, E. Rignot, Warm ocean is eroding West Antarctic Ice Sheet. *Geophys. Res. Lett.* **31**, L23402 (2004).

105. A. Shepherd, D. Wingham, D. Wallis, K. Giles, S. Laxon, A. V. Sundal, Recent loss of floating ice and the consequent sea level contribution. *Geophys. Res. Lett.* **37**, (2010).

106. C. A. Greene, D. E. Gwyther, D. D. Blankenship, Antarctic mapping tools for MATLAB. *Comput. Geosci.* **104**, 151–157 (2017).
